# Efficacy of Pneumococcal Nontypable *Haemophilus influenzae* Protein D Conjugate Vaccine (PHiD-CV) in Young Latin American Children: A Double-Blind Randomized Controlled Trial

**DOI:** 10.1371/journal.pmed.1001657

**Published:** 2014-06-03

**Authors:** Miguel W. Tregnaghi, Xavier Sáez-Llorens, Pio López, Hector Abate, Enrique Smith, Adriana Pósleman, Arlene Calvo, Digna Wong, Carlos Cortes-Barbosa, Ana Ceballos, Marcelo Tregnaghi, Alexandra Sierra, Mirna Rodriguez, Marisol Troitiño, Carlos Carabajal, Andrea Falaschi, Ana Leandro, Maria Mercedes Castrejón, Alejandro Lepetic, Patricia Lommel, William P. Hausdorff, Dorota Borys, Javier Ruiz Guiñazú, Eduardo Ortega-Barría, Juan P. Yarzábal, Lode Schuerman, on behalf of the COMPAS Group

**Affiliations:** 1 Centro de Desarrollo del Proyectos Avanzados en Pediatría, Córdoba, Argentina; 2 Department of Infectious Diseases, Hospital del Niño, Panama City, Panama; 3 Centro de Estudios en Infectología Pediátrica, Cali, Colombia; 4 Department of Infectious Diseases, Hospital Notti, Mendoza, Argentina; 5 Centro de Desarrollo del Proyectos Avanzados en Pediatría, Santiago del Estero, Argentina; 6 Centro de Desarrollo del Proyectos Avanzados en Pediatría, San Juan, Argentina; 7 Health Research International, Panama City, Panama; 8 Instituto de Investigaciones Científicas y Servicios de Alta Tecnología, Panama City, Panama; 9 Department of Pediatrics, Hospital del Niño, Panama City, Panama; 10 GlaxoSmithKline Vaccines, Panama City, Panama; 11 GlaxoSmithKline Vaccines, Buenos Aires, Argentina; 12 GlaxoSmithKline Vaccines, Wavre, Belgium; Public Health England, United Kingdom

## Abstract

**Background:**

The relationship between pneumococcal conjugate vaccine–induced antibody responses and protection against community-acquired pneumonia (CAP) and acute otitis media (AOM) is unclear. This study assessed the impact of the ten-valent pneumococcal nontypable *Haemophilus influenzae* protein D conjugate vaccine (PHiD-CV) on these end points. The primary objective was to demonstrate vaccine efficacy (VE) in a per-protocol analysis against likely bacterial CAP (B-CAP: radiologically confirmed CAP with alveolar consolidation/pleural effusion on chest X-ray, or non-alveolar infiltrates and C-reactive protein ≥ 40 µg/ml); other protocol-specified outcomes were also assessed.

**Methods and Findings:**

This phase III double-blind randomized controlled study was conducted between 28 June 2007 and 28 July 2011 in Argentine, Panamanian, and Colombian populations with good access to health care. Approximately 24,000 infants received PHiD-CV or hepatitis control vaccine (hepatitis B for primary vaccination, hepatitis A at booster) at 2, 4, 6, and 15–18 mo of age. Interim analysis of the primary end point was planned when 535 first B-CAP episodes, occurring ≥2 wk after dose 3, were identified in the per-protocol cohort. After a mean follow-up of 23 mo (PHiD-CV, *n* = 10,295; control, *n* = 10,201), per-protocol VE was 22.0% (95% CI: 7.7, 34.2; one-sided *p* = 0.002) against B-CAP (conclusive for primary objective) and 25.7% (95% CI: 8.4%, 39.6%) against World Health Organization–defined consolidated CAP. Intent-to-treat VE was 18.2% (95% CI: 5.5%, 29.1%) against B-CAP and 23.4% (95% CI: 8.8%, 35.7%) against consolidated CAP. End-of-study per-protocol analyses were performed after a mean follow-up of 28–30 mo for CAP and invasive pneumococcal disease (IPD) (PHiD-CV, *n* = 10,211; control, *n* = 10,140) and AOM (*n* = 3,010 and 2,979, respectively). Per-protocol VE was 16.1% (95% CI: −1.1%, 30.4%; one-sided *p* = 0.032) against clinically confirmed AOM, 67.1% (95% CI: 17.0%, 86.9%) against vaccine serotype clinically confirmed AOM, 100% (95% CI: 74.3%, 100%) against vaccine serotype IPD, and 65.0% (95% CI: 11.1%, 86.2%) against any IPD. Results were consistent between intent-to-treat and per-protocol analyses. Serious adverse events were reported for 21.5% (95% CI: 20.7%, 22.2%) and 22.6% (95% CI: 21.9%, 23.4%) of PHiD-CV and control recipients, respectively. There were 19 deaths (*n* = 11,798; 0.16%) in the PHiD-CV group and 26 deaths (*n* = 11,799; 0.22%) in the control group. A significant study limitation was the lower than expected number of captured AOM cases.

**Conclusions:**

Efficacy was demonstrated against a broad range of pneumococcal diseases commonly encountered in young children in clinical practice.

**Trial registration:**

www.ClinicalTrials.gov NCT00466947

*Please see later in the article for the Editors' Summary*

## Abstract 2

In a double-blind randomized controlled trial, Xavier Saez-Llorens and colleagues examine the vaccine efficacy of PHiD-CV against community-acquired pneumonia in young children in Panama, Argentina, and Columbia.

*Please see later in the article for the Editors' Summary*

## Editors' Summary

**Background:**

Pneumococcal diseases are illnesses caused by *Streptococcus pneumonia*e bacteria, pathogens (disease-causing organisms) that are transmitted through contact with infected respiratory secretions. *S. pneumoniae* causes mucosal diseases–infections of the lining of the body cavities that are connected to the outside world–such as community-acquired pneumonia (CAP; lung infection) and acute otitis media (AOM; middle-ear infection). It also causes invasive pneumococcal diseases (IPDs) such as septicemia and meningitis (infections of the bloodstream and the covering of the brain, respectively). Although pneumococcal diseases can sometimes be treated with antibiotics, CAP and IPDs are leading global causes of childhood deaths, particularly in developing countries. It is best therefore to avoid *S. pneumoniae* infections through vaccination. Vaccination primes the immune system to recognize and attack pathogens rapidly and effectively by exposing it to weakened or dead pathogens or to pathogen molecules that it recognizes as foreign (antigens). Because there are more than 90 *S. pneumoniae* variants (“serotypes”), each characterized by a different antigenic polysaccharide (complex sugar) coat, *S. pneumoniae* vaccines have to include antigens from multiple serotypes. For example, the PHiD-CV vaccine contains polysaccharides from ten *S. pneumoniae* serotypes.

**Why Was This Study Done?:**

Although in most countries PHiD-CV has been licensed for protection against IPD and pneumococcal AOM, at the time of study, it was not known how well it protected against CAP and overall AOM, which are important public health problems. In this double-blind randomized controlled trial (the Clinical Otitis Media and Pneumonia Study; COMPAS), the researchers investigate the efficacy of PHiD-CV against CAP and AOM and assess other clinical end points, such as IPD, in Latin American infants. Double-blind randomized controlled trials compare the effects of interventions by assigning study participants to different interventions randomly and measuring predefined outcomes without the study participants or researchers knowing who has received which intervention until the trial is completed. Vaccine efficacy is the reduction in the incidence of a disease (the number of new cases that occur in a population in a given time) among trial participants who receive the vaccine compared to the incidence among participants who do not receive the vaccine.

**What Did the Researchers Do and Find?:**

The researchers enrolled around 24,000 infants living in urban areas of Argentina, Panama, and Colombia. Half the infants were given PHiD-CV at 2, 4, and 6 months of age and a booster dose at age 15–18 months. The remaining infants were given a hepatitis control vaccine at the same intervals. The trial's primary end point was likely bacterial CAP (B-CAP) –radiologically confirmed CAP, with the airspaces (alveoli) in the lungs filled with liquid instead of gas (alveolar consolidation) or with non-alveolar infiltrates and raised blood levels of C-reactive protein (a marker of inflammation). In a planned interim analysis, which was undertaken after an average follow-up of 23 months, the vaccine efficacy in the per-protocol cohort (the group of participants who actually received their assigned intervention) was 22% against B-CAP. Intent-to-treat vaccine efficacy in the interim analysis (which considered all the trial participants regardless of whether they received their assigned intervention) was 18.2%. At the end of the study (average follow up 30 months), the vaccine efficacy against B-CAP was 18.2% and 16.7% in the per-protocol and intent-to-treat cohorts, respectively. Per-protocol vaccine efficacies against clinically confirmed AOM and vaccine serotype AOM were 16.1% and 67.1%, respectively. Against any IPD and against vaccine serotype IPD, the respective vaccine efficacies were 65% and 100%. Finally, about one-fifth of children who received PHiD-CV and a similar proportion who received the control vaccine experienced a serious adverse event (for example, gastroenteritis); 19 children who received PHiD-CV died compared to 26 children who received the control vaccine.

**What Do These Findings Mean?:**

These findings indicate that in Latin America, a region with an intermediate burden of pneumococcal disease, PHiD-CV is efficacious against a broad range of pneumococcal diseases that often affect young children. The accuracy of these findings may be limited by the withdrawal of 14% of participants from the trial because of adverse media coverage and by the low number of reported cases of AOM. Moreover, because most study participants lived in urban areas, these findings may not be generalizable to rural settings. Despite these and other study limitations, these findings provide new information about the magnitude of the effect of PHiD-CV vaccination against CAP and AOM, two mucosal pneumococcal diseases of global public health importance.

**Additional Information:**

Please access these websites via the online version of this summary at http://dx.doi.org/10.1371/journal.pmed.1001657.

- The US Centers for Disease Control and Prevention provides information for patients and health professionals on all aspects of pneumococcal disease and pneumococcal vaccination, including personal stories
- Public Health England provides information on pneumococcal disease and on pneumococcal vaccines
- The not-for-profit Immunization Action Coalition has information on pneumococcal disease, including personal stories
- The GAVI Alliance provides information about pneumococcal disease and the importance of vaccination
- MedlinePlus has links to further information about pneumococcal infections, including pneumonia and otitis media (in English and Spanish)
- More information about COMPAS is available
- The European Medicines Agency provides information about PHiD-CV (Synflorix)

## Introduction

*Streptococcus pneumoniae* is a major cause of various diseases, ranging from septicemia and meningitis to pneumonia and acute otitis media (AOM). As community-acquired pneumonia (CAP) is a leading cause of childhood mortality [1], the World Health Organization (WHO) recommends inclusion of pneumococcal conjugate vaccines (PCVs) in childhood immunization programs [2].

In Latin America, pneumococcal disease rates among young children are intermediate in comparison with other global areas [3], but the impact of PCVs in diminishing this burden has not been assessed in this region. Pneumococcal serotypes included in the ten-valent pneumococcal nontypable *Haemophilus influenzae* protein D conjugate vaccine (PHiD-CV) represent 70%–80% of those that cause invasive pneumococcal disease (IPD) and AOM in young children in Latin America [4],[5]. PHiD-CV was licensed for protection against IPD based on demonstration of immunological non-inferiority to the seven-valent pneumococcal CRM-conjugate vaccine (7vCRM; Prevenar/Prevnar, Pfizer) [6], using criteria proposed by the WHO [7].

In contrast, for mucosal diseases–e.g., pneumonia and AOM–no such licensing criteria are defined. Furthermore, antibody levels for most of the pneumococcal serotypes contained in both vaccines, when expressed as geometric mean concentrations, tended to be higher with 7vCRM than with PHiD-CV [6]. This has unknown implications for the magnitude of protection against mucosal diseases of importance to public health. Also, the etiology of the mucosal diseases involves many bacterial and viral pathogens [5],[8]–[10] and can be affected by factors such as variations in pneumococcal serotype incidence [11]–[13], care-seeking behavior, and antibiotic prescription practices [14]. Since double-blind randomized controlled trials are the gold standard for establishing vaccine efficacy (VE), the Clinical Otitis Media and Pneumonia Study (COMPAS) was designed to demonstrate the efficacy of PHiD-CV against CAP and AOM, and to assess other clinical end points, such as IPD, in young Latin American children.

## Methods

### Ethics Statement

The trial was sponsored by GlaxoSmithKline Biologicals. An independent data monitoring committee (IDMC), composed of seven independent experts in infectious diseases and/or statistics, provided oversight by reviewing serious adverse events (SAEs) and assessing potential treatment harm. The IDMC also made recommendations to the sponsor regarding safety measures, study design, and analysis and reporting plans. Written informed consent was obtained from children's parents/guardians, and the study was conducted in accordance with good clinical practice, all applicable regulatory requirements, and the Declaration of Helsinki. When deviations from these guidelines/regulatory requirements were detected (Text S1), corrective actions were implemented and reported to the ethics committees, IDMC, and regulatory authorities. The trial protocol (Text S2) was approved by national public health authorities and the ethical review committees for each study site (Table S1). Major amendments made to the protocol, including changes to the planned interim analysis, are listed in Text S3.

### Study Setting, and Socioeconomic and Public Health Indicators

This study was conducted at five sites: three in Argentina (Mendoza, San Juan, and Santiago del Estero), one in Colombia (Cali), and one in Panama (Panama City). The study locations were chosen partly because local investigators and public health authorities from the three countries were experienced in collaborative epidemiological surveillance research on pneumococcal diseases, including an international surveillance study of invasive bacterial isolates (SIREVA [Sistema Regional de Vacunas] and SIREVA II [Sistema de Redes de Vigilancia de los Agentes Bacterianos Responsables de Neumonías y Meningitis]) [15],[16]. Investigators in Panama were also experienced in the conduct of AOM studies [17]–[20].

The study areas were mainly urban, and local climates have either clear seasonality (Argentina) or are tropical or subtropical (Panama and Colombia). Populations in these countries generally have good access to health care and drug treatments, including antibiotics (upon prescription in Argentina and Panama, but freely available in Colombia). Immunization coverage for routine childhood vaccines is high in each country (in 2011, approximately 80% to 99% for recommended vaccines) [21]. Seasonal influenza vaccination has been recommended since 2010 in Argentina and since 2005 in Colombia and Panama for 6- to 24-mo-old children [22],[23], for whom coverage is at least 70% in Argentina and Panama.

All three countries are classified by the World Bank, based on gross national income per capita, as upper middle income economies, a classification shared by many other countries in Latin America, Eastern Europe, Asia, the Middle East, and North Africa [24]. Gross domestic product per capita in 2011 was US$10,942 for Argentina, US$7,498 for Panama, and US$7,104 for Colombia. Adult life expectancy is similar among the countries (76 to 78 y), as is the adult literacy rate (93% to 98%) [25]. In comparison to the global infant mortality rate (37 deaths per 1,000 live births) [26], infant mortality rates are low (13, 17, and 15 per 1,000 live births in Argentina, Panama, and Colombia, respectively) and similar to or lower than rates in other countries with upper middle income economies (average 16 per 1,000 live births) [24] but higher than the average rate in the European Union (4 per 1,000 live births) [27]. Each study area had health care centers and at least one public pediatric hospital that were readily accessible to participants by public transport.

### Study Design

This was a phase III double-blind randomized controlled study. The primary confirmatory objective of the study was to demonstrate the VE of PHiD-CV (three doses in children aged 18 mo or less, or four doses in children aged over 18 mo) against first episodes of likely bacterial CAP (B-CAP) occurring at least 2 wk after administration of the third dose of study vaccine in the per-protocol cohort for efficacy. Efficacy against clinically confirmed AOM (C-AOM) was evaluated as the first secondary confirmatory objective; efficacy against IPD and other CAP and AOM end points were assessed as secondary objectives (Table 1).

**Table 1:** Study objectives.

Objective	Study Cohort
**Primary objective**	
To demonstrate the efficacy of PHiD-CV against B-CAP	All children
**Secondary objectives**	
To demonstrate the efficacy of PHiD-CV against C-AOM	7,000 children enrolled in Panama
To assess the efficacy of PHiD-CV against CAP with alveolar consolidation or pleural effusion on chest X-ray	All children
To assess the efficacy of PHiD-CV in preventing bacteriologically confirmed AOM cases caused by: Any bacterial pathogen Vaccine, cross-reactive, and other *S. pneumoniae* serotypes *H. influenzae* Nontypable *H. influenzae* Other AOM pathogens (e.g., *Moraxella catarrhalis*, Group A streptococci, *Staphylococcus aureus*)	7,000 children enrolled in Panama
To document the impact of PHiD-CV against: CAP cases with alveolar consolidation or pleural effusion on chest X-ray and positive respiratory viral test CAP cases with any abnormal chest X-ray with positive respiratory viral test B-CAP cases with positive respiratory viral test	All children
To document the impact of PHiD-CV against: Suspected CAP cases CAP cases with any abnormal chest X-ray Suspected CAP cases with CRP ≥ 40/80/120 µg/ml, regardless of chest X-ray reading CAP cases with either alveolar consolidation/pleural effusion on chest X-ray or with non-alveolar infiltrates and CRP ≥ 80/120 µg/ml	All children
To document the impact of PHiD-CV against: Bacteriologically culture-confirmed IPD cases caused by any of the ten pneumococcal VTs VT IPD identified through positive culture or nonculture pneumococcal diagnosis with additional nonculture VT serotyping Invasive disease caused by cross-reactive pneumococcal serotypes, other pneumococcal serotypes, and *H. influenzae*	All children
To document the impact of PHiD-CV on reducing nasopharyngeal carriage of *S. pneumoniae* (VTs and others) and *H. influenzae*	Subset of 2,000 children in Panama
To document the impact of PHiD-CV on antibiotic prescriptions	Subset of 2,000 children in Panama (same as carriage subset)
To assess the immune response to PHiD-CV	Subset of 1,000 children in Argentina and Panama
To assess the reactogenicity of PHiD-CV in terms of solicited general and local symptoms	Subset of 1,000 children in Argentina and Panama (same as immunogenicity subset)
To assess the safety of PHiD-CV in terms of unsolicited adverse events	7,000 children enrolled in Panama
To assess the safety of PHiD-CV in terms of SAEs occurring during the entire study period	All children

AOM, acute otitis media; B-CAP, likely bacterial community-acquired pneumonia; C-AOM, clinically confirmed acute otitis media; CAP, community-acquired pneumonia; CRP, C-reactive protein; IPD, invasive pneumococcal disease; PHiD-CV, pneumococcal nontypable *Haemophilus influenzae* protein D conjugate vaccine; SAE, serious adverse event; VT, vaccine serotype.

**Figure 1:**
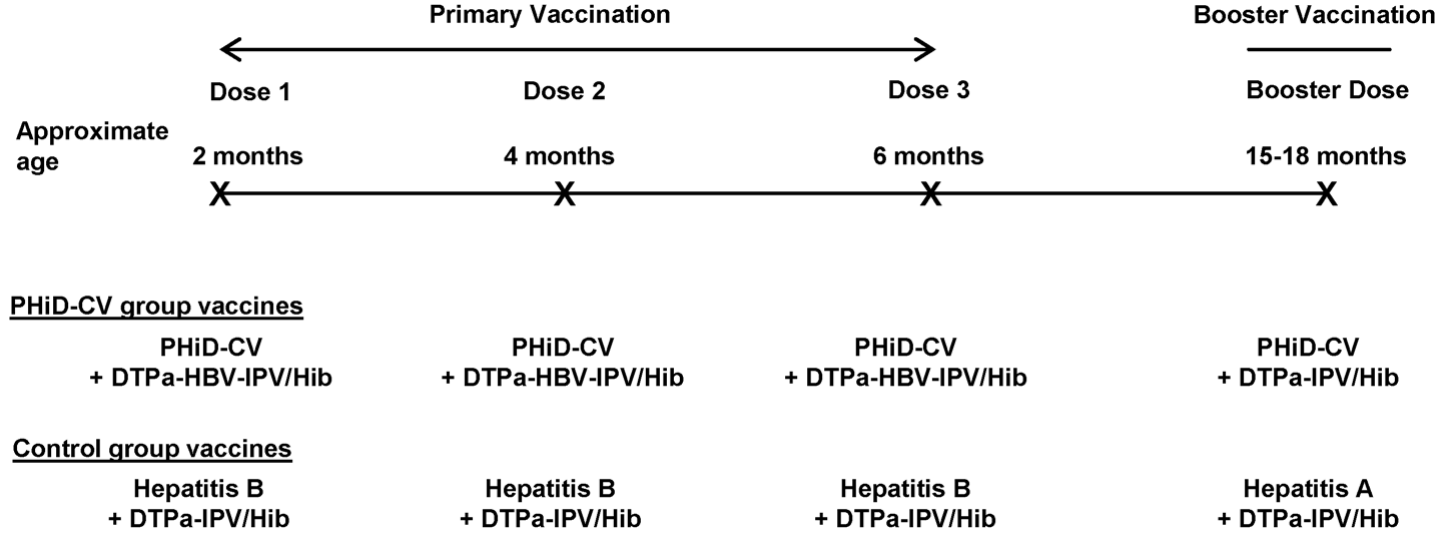
Vaccination schedule. The following vaccines were used: PHiD-CV, Synflorix; diphtheria–tetanus–acellular pertussis–hepatitis B–inactivated poliovirus–*Haemophilus influenzae* type b vaccine (DTPa-HBV-IPV/Hib), Infanrix hexa; DTPa-IPV/Hib, Infanrix-IPV/Hib; hepatitis B, Engerix-B; hepatitis A, Havrix (all by GlaxoSmithKline Vaccines). In addition to these blinded study vaccines, the following vaccines were administered or were recommended: measles–mumps–rubella vaccine at 12 mo of age, hepatitis B vaccination at birth, and hepatitis A vaccination at 12 and 18–21 mo of age, with the second dose given at least 28 days after the study vaccine booster dose. In Argentina, *Neisseria meningitidis* group C conjugate vaccine (NeisVac-C, Baxter International) was offered at 12 mo of age; in Colombia and Panama, varicella vaccine (Varilrix, GlaxoSmithKline Vaccines) was offered at 12 mo of age; in Colombia, two doses of oral rotavirus vaccine (Rotarix, GlaxoSmithKline Vaccines) were offered within the first 6 mo of life.

Healthy infants aged 6–16 wk were enrolled by pediatricians during well-baby clinic visits. Enrollment took place from 28 June 2007 until 30 December 2008. The inclusion and exclusion criteria for participation in the trial are listed in Table 2. Infants were randomized (1∶1 ratio) to receive either PHiD-CV (Synflorix) and diphtheria–tetanus–acellular pertussis–hepatitis B–inactivated poliovirus–*Haemophilus influenzae* type b vaccine (Infanrix hexa) (PHiD-CV group) or hepatitis B vaccine (Engerix-B) and diphtheria–tetanus–acellular pertussis–inactivated poliovirus–*Haemophilus influenzae* type b vaccine (DTPa-IPV/Hib; Infanrix-IPV/Hib) (control group) at approximately 2, 4, and 6 mo of age. This was followed by one dose of PHiD-CV or hepatitis A vaccine (Havrix), respectively, at 15–18 mo of age, both coadministered with DTPa-IPV/Hib (Figure 1).

**Table 2:** Inclusion and exclusion criteria.

Inclusion Criteria	Exclusion Criteria
A male or female between, and including, 6 and 16 wk of age (between 42 and 118 d) at the time of the first vaccination. Infants born preterm (after a gestation period of <37 wk) can be included in the study starting from 8 wk of chronological age at the time of first vaccination and up to 16 wk of chronological age (between 56 and 118 d). Residence in the prespecified disease surveillance area. Written informed consent obtained from the parent or guardian of the child. Free of any known or suspected health problems (as established by medical history and clinical examination before entering into the study) that would contraindicate the initiation of routine immunizations outside a clinical trial context. Children for whom the investigator believes that their parents/guardians can and will comply with the requirements of the protocol (e.g., completion of the diary cards, return for follow-up visits).	Use or planned use of any investigational or unregistered drug or vaccine other than the study vaccines. If seven-valent pneumococcal conjugate vaccine immunization needs to be initiated because of the presence of a high risk for pneumococcal infections for which the seven-valent pneumococcal conjugate vaccine is made locally available, the child cannot be enrolled. Previous vaccination against diphtheria, tetanus, pertussis, *H. influenzae* type b, hepatitis A, and/or *S. pneumoniae*. Locally recommended Expanded Program on Immunization vaccines given at birth (such as BCG [Bacillus Calmette–Guérin], hepatitis B, oral poliovirus) are allowed, but should be administered at least 1 mo before the first dose of the study vaccine. Other locally recommended vaccines are allowed, even if concomitantly administered with the study vaccines. History of allergic disease or reactions likely to be exacerbated by any component of the vaccines. History of any neurologic disorders or seizures. Acute disease at the time of enrollment, defined as the presence of a moderate or severe illness with or without fever. Presence of acute disease and/or temperature (rectal temperature ≥ 38.0 °C or ≥ 37.5 °C for any other route) warrants delay of enrollment until the illness has improved. Low birth weight (<2,500 g) not permitted for Colombia.

Vaccine allocation at each site was performed using a central randomization system on the Internet (SBIR, GlaxoSmithKline Vaccines), and treatment was concealed from all study personnel. Information on individual randomization could be obtained only through SBIR by the study safety physician if unblinding was needed, for example, because of an individual safety issue. Twenty-three participants were unblinded by the study safety physician as part of the emergency unblinding process or because the study participant was at risk for pneumococcal disease because of a medical condition diagnosed during the study. As a consequence they were censored at the time of unblinding, as defined in the statistical analysis plan. Three additional participants were unblinded by the study safety physician because of a suspected unexpected serious adverse reaction but remained in the per-protocol cohort for efficacy analysis. The randomization list was generated by the sponsor using a standard SAS (SAS Institute) program and was used to number the vaccines. A randomization blocking scheme was used to ensure that balance between treatment groups was maintained.

Since there were minor differences in vaccine appearance, an observer-blind methodology was used to maintain double-blinding and avoid possible observer bias. This means that all vaccines were prepared and administered by dedicated personnel who took no further part in the study. The parents/guardians of children enrolled in the study and personnel involved in data gathering, processing, and analysis and safety assessment were blind to vaccine allocation.

Exclusion/elimination criteria for further study participation were checked for all children at each scheduled and unscheduled visit after the first visit, including high-risk conditions for pneumococcal infections as defined by the American Academy of Pediatrics and the Argentinean Pediatric Society (Table S2). The diagnosis of high-risk conditions for pneumococcal infection was made according to medical judgment (as established by medical history and clinical examination) and confirmed by appropriate tests, if clinically required. If a high-risk condition for pneumococcal infection requiring pneumococcal conjugate vaccination was diagnosed, the child was unblinded to ensure receipt of a licensed PCV according to the age-appropriate immunization schedule. If a licensed PCV was available through a targeted national immunization program for high-risk groups, the investigator ensured that children belonging to high-risk groups received that vaccine. If no locally licensed PCV was available, it was provided to the investigator by the study sponsor. The date of unblinding for these children was recorded in the electronic case report form, and efficacy follow-up data collected after this date no longer contributed to the primary efficacy analysis.

### Study Vaccines and Vaccination Schedule

All study vaccines were manufactured by GlaxoSmithKline Vaccines. PHiD-CV contained 1 µg of each capsular polysaccharide for pneumococcal serotypes 1, 5, 6B, 7F, 9V, 14, and 23F and 3 µg for serotype 4 conjugated individually to protein D (a novel carrier protein derived from nontypable *H. influenzae*); 3 µg of serotype 18C capsular polysaccharide conjugated to tetanus toxoid; and 3 µg of serotype 19F capsular polysaccharide conjugated to diphtheria toxoid [6]. The hepatitis A vaccine contained inactivated hepatitis A virus antigen (720 ELISA units, strain HM 175), and the hepatitis B vaccine contained hepatitis B surface antigen (10 µg). All study vaccines were administered intramuscularly into the thigh or deltoid. The vaccination schedule and vaccines permitted for concomitant administration are listed in Figure 1.

### Choice of Control Vaccines

COMPAS was designed to assess the impact of PHiD-CV compared to the standard of care in place in the participating countries at the time of study initiation. When the study began in 2007 and until the end of primary vaccination, 7vCRM was not included in the Expanded Program on Immunization of the participating countries, and uptake via prescription by individual physicians was limited (less than 5%). Inclusion of a 7vCRM control group in our study was therefore deemed not mandatory [28]. In fact, assessment of the relative efficacy of a new vaccine compared to a licensed vaccine for which the impact in Latin America had not been previously determined would not permit understanding of the vaccine's real public health value in that region. The ethical acceptability of a pneumonia efficacy trial in Latin America with a 7vCRM control group that does not allow meaningful conclusions to be drawn would therefore be questionable. Thus, the choice of control vaccines maintained the standard of care in the three countries and was approved by national public health and regulatory authorities and ethical review committees for each study site.

### Disease Surveillance and Case Definitions

Disease surveillance involved the major local pediatric hospitals and primary health care centers. Parents were advised to (1) seek medical attention if their child had any health problem, including fever or respiratory symptoms, (2) highlight their child's participation in COMPAS when seeking medical care, and (3) inform study personnel as soon as possible. Parents were asked at each study visit about symptoms of illness experienced by their child since the previous visit. Disease episodes were also captured by study personnel blinded to vaccine allocation via screenings of emergency room, hospital admissions, radiology, and outpatient records, and mortuary lists.

#### Pneumonia surveillance

Suspected pneumonia cases were identified by pediatricians based on medical history and clinical symptoms. Chest X-ray and laboratory tests to guide diagnosis and treatment were performed according to local routine clinical practice and the judgment of the treating physician. A child with an acute respiratory tract infection, referred by the treating pediatrician for a chest X-ray as part of the clinical assessment, was considered for suspected CAP study end points. Children with only symptoms of wheezing diseases (such as bronchiolitis and hyperreactive bronchial syndromes) were not considered for suspected CAP end points. Serum samples for C-reactive protein (CRP) measurement were obtained from suspected CAP cases and stored at −20 °C until analyzed at the central laboratory.

**Figure 2:**
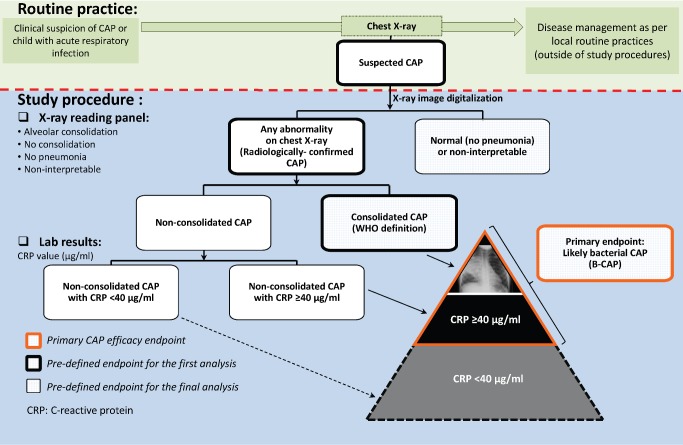
Chest X-ray classification and CAP end point definitions.

A group composed of representatives of participating radiology departments was established to install a quality assurance system in all centers where chest X-rays were performed. Analog X-ray films were digitalized using a Sierra Euro scanner (Vidar Systems Corporation), while digital X-ray images were used as such. Interpretation and classification of the digital images for study end point assessment was performed by an independent panel of external readers trained in WHO definitions before the start of image evaluation and regularly thereafter [29]. First, X-rays were independently classified by two of the external readers (a pediatric radiologist and a pediatrician) as normal or radiologically confirmed CAP (Figure 2). The latter category included any suspected CAP with pulmonary infiltrates on chest X-ray (including perihilar infiltrates and other abnormal findings). Radiologically confirmed CAP cases were further classified as either consolidated CAP (defined as radiologically confirmed CAP with alveolar consolidation/pleural effusion on the chest X-ray according to the WHO definition [29]) or nonconsolidated CAP. X-rays were categorized as non-interpretable if the quality of the chest X-ray did not allow conclusions to be made regarding the presence or absence of abnormalities on the pulmonary parenchyma. If there was disagreement in the classification of individual images between the two initial X-ray reviewers, another panel of two pediatric radiologists examined the image to reach a consensus.

The CAP case definitions used in COMPAS overlap, and as they become more specific for CAP with bacterial origin, they also tend to detect fewer cases (i.e., lose some sensitivity). The suspected CAP case definition, being the broadest, encompassed all other CAP case definitions and was selected because it reflects the first step in the clinical assessment of a sick child by the treating physician: to identify potential pneumonia cases that require further evaluation. The radiologically confirmed CAP case definition represents the next step in the clinical reasoning process, where the chest X-ray is examined in an attempt to confirm the presence or absence of pneumonia. However, for study purposes and end point evaluation, as described above, assessment of X-rays was performed by external readers, independently of disease management procedures. The consolidated CAP case definition was proposed by the WHO to facilitate comparisons between pneumonia efficacy trials [29]. It is the most stringent case definition and therefore captures the smallest number of CAP cases. However, in previous PCV efficacy trials, it was observed that PCVs prevented more CAP cases than those captured by the WHO consolidated CAP case definition [30], and the investigators proposed an expanded case definition that included, in addition, all X-ray-confirmed CAP cases that had elevated CRP levels (≥40 µg/ml).

In COMPAS, B-CAP was therefore defined as radiologically confirmed CAP with either alveolar consolidation/pleural effusion or non-alveolar infiltrates and CRP ≥ 40 µg/ml (Figure 2). The B-CAP case definition was chosen as the primary end point, as it fully encompasses the consolidated CAP case definition and adds additional B-CAP cases to that definition.

#### AOM surveillance

The AOM outcome was studied in Panama only. Training in order to standardize assessment of AOM cases was provided before the start of the study and regularly thereafter to the physicians involved with the study, which followed recommendations provided by an external consultant with expertise in AOM. Initially, cases of AOM were captured only when parents sought medical attention for children with symptoms of AOM. However, approximately 2 y into the study (July 2009), because of a lower than expected AOM rate, the surveillance was enhanced through regular telephone calls or home visits by study personnel, who advised parents to visit the clinic if their child had symptoms suggestive of AOM. If AOM was suspected by the physician, the child was referred to one of the ear, nose, and throat (ENT) specialists involved in the trial. Study pediatricians and physicians on duty in emergency departments were asked not to initiate antibiotic treatment before referral to the ENT specialist.

A C-AOM diagnosis required either altered visual appearance of the tympanic membrane (e.g., redness, bulging, loss of light reflex) or the presence of middle-ear effusion (as demonstrated by simple or pneumatic otoscopy or by otomicroscopy). At least two of the following clinical symptoms, which started within the previous 5 d, were also required: ear pain, ear discharge, hearing loss, fever, lethargy, irritability, anorexia, vomiting, or diarrhea.

The severity of each suspected AOM case was assessed, combining objective elements of the Friedman scale (the Ear Treatment Group–five items [ETG-5] and otoscopy scale with eight grades of severity [OS-8]) [31] with subjective elements in the clinical/otologic scale developed by Dagan et al. [32]. This scale is based on five symptoms: temperature, irritability, and ear tugging on the day of the visit, as reported by parents, and redness and bulging of the tympanic membrane, as observed by otoscopy. Scores of 0 to 4, 5 to 7, and 8 to 15 were considered indicative of mild, moderate, and severe AOM, respectively. Clinical data were collected by the pediatrician or general physician who referred the child to the ENT specialist. Objective data related to observation of the ear drum were collected by the ENT specialist. When fluid was suspected to be in the middle ear, following specific informed consent, tympanocentesis was performed using a Channel Directed Tympanocentesis speculum (Walls Precision Instruments). Bacterial AOM was determined if the fluid, assessed via standard microbiological culture methods, contained a bacterial pathogen recognized as causative of AOM.

#### Invasive disease surveillance

Invasive disease was defined as any disease where *S. pneumoniae* or *H. influenzae* was identified in normally sterile body fluids such as blood, cerebrospinal fluid (CSF), pleural effusion, synovial fluid, or peritoneal fluid.

Various clinical syndromes of focal invasive disease were recorded. These syndromes included purulent meningitis (CSF with >10 polymorphonuclear cells/mm^3^, >100 leukocytes/mm^3^, or 10–99 leukocytes/mm^3^ and either glucose <30 mg/dl or protein >100 mg/dl), confirmed meningitis due to *S. pneumoniae* or *H. influenzae* (presence of *S. pneumoniae* or *H. influenzae* in CSF culture or purulent meningitis with blood culture positive for *S. pneumoniae* or *H. influenzae*), and probable bacterial meningitis due to *S. pneumoniae* or *H. influenzae* (purulent meningitis with negative result for CSF and blood culture but with antigen testing [Latex or BinaxNOW] positive for *S. pneumoniae* or *H. influenzae* in CSF, or meningitis with blood culture positive for *S. pneumoniae* or *H. influenzae* and with negative result for CSF culture but antigen testing [Latex or BinaxNOW] positive for *S. pneumoniae* or *H. influenzae* in CSF). Other clinical syndromes of focal invasive disease that were recorded included the following: bacteremic pneumonia (pneumonia confirmed by radiological evidence of any lung infiltrate and the presence of *S. pneumoniae* or *H. influenzae* identified by blood culture), empyema (isolation of *S. pneumoniae* or *H. influenzae* from pleural fluid obtained by thoracentesis, or pleural fluid with polymorphonuclear cells and isolation of *S. pneumoniae* or *H. influenzae* from blood culture), peritonitis (isolation of *S. pneumoniae* or *H. influenzae* from peritoneal fluid obtained by laparocentesis, paracentesis, or laparotomy), osteomyelitis (isolation of *S. pneumoniae* or *H. influenzae* from bone aspirate, or clinical symptoms and imaging compatible with osteomyelitis and isolation of *S. pneumoniae* or *H. influenzae* from blood culture), soft tissue infection (positive blood culture for *S. pneumoniae* or *H. influenzae* and clinical signs of cellulitis or abscesses, or isolation of *S. pneumoniae* or *H. influenzae* from abscess-aspirated material), septic arthritis (clinical and radiological presentation compatible with acute arthritis and positive blood culture for *S. pneumoniae* or *H. influenzae*, or isolation of *S. pneumoniae* or *H. influenzae* from material obtained by arthocentesis or arthrotomy), pericarditis (isolation of *S. pneumoniae* or *H. influenzae* from fluid obtained by pericardiocentesis or pericardiotomy, or presence of pleural friction rubs, muted heart sounds, or alterations indicative of a pericardial effusion in thoracic radiology, echocardiogram, or electrocardiogram and isolation of *S. pneumoniae* or *H. influenzae* from blood culture).

Invasive disease without focal infection, such as bacteremia (positive blood culture for *S. pneumoniae* or *H. influenzae* indicating viable bacteria in blood without an obvious focus of infection), was also reported. In addition, generalized infections were reported as sepsis (defined as the presence of a systemic inflammatory response syndrome as a result of suspected or proven infection [33]), severe sepsis (sepsis plus cardiovascular organ dysfunction or acute respiratory distress syndrome or two or more other organ dysfunctions), or septic shock (sepsis and cardiovascular organ dysfunction).

Identification of cases of invasive disease was based on clinical suspicion of the treating physician followed by a positive culture. Blood culture was recommended in all cases of febrile illness (axillary fever≥39.0°C or rectal temperature≥39.5°C) without overt focus of infection or with suspicion of meningitis, pneumonia, empyema, septic arthritis, peritonitis, osteomyelitis, pericarditis, or bacterial infection of soft tissue. In addition, a blood culture was to be seriously considered in children with a recent (within 24 h) history of febrile illness, especially if antipyretic treatment could have interfered with the temperature measurement at the time of physical examination of the child. In Panama, where dengue was endemic, it was recommended to perform dengue testing, according to local practices.

In addition, study personnel at each site reviewed the records of the microbiology laboratories to identify study participants for whom *S. pneumoniae* or *H. influenzae* was isolated from blood cultures or other normally sterile body fluids and/or CSF, and in which testing for *S. pneumoniae* or *H. influenzae* antigens (BinaxNOW or Latex) was positive.

### Immunogenicity Assessment

Immune responses against PHiD-CV antigens were planned to be assessed in approximately 1,000 infants (first 500 enrolled in selected centers in Argentina and Panama) and are reported from blood samples taken 1 mo after dose 3, just before booster, and 1 mo after booster vaccination. Serum anti-pneumococcal, serotype-specific IgG antibodies were measured using GlaxoSmithKline's 22F-inhibition ELISA with a cutoff value of 0.05 µg/ml, as described before [34],[35]. An antibody concentration of 0.2 µg/ml measured by this 22F-inhibition ELISA correspond to a concentration of 0.35 µg/ml in the WHO reference laboratory non-inhibition assay [36]. Opsonophagocytic activity (OPA) was measured using a pneumococcal killing assay with HL60 phagocytes, as described previously [37], with a cutoff titer of eight [38]. Antibody concentrations and OPA titers were also determined for cross-reactive serotypes 6A and 19A. Antibodies against nontypable *H. influenzae* protein D were measured by ELISA with an assay cutoff of 100 ELISA units (ELU)/ml.

### Serious Adverse Events

SAEs are reported in this paper. Investigators were requested to report any SAE, defined as any medical event that resulted in death, was life-threatening, caused disability, or required hospitalization or prolongation of hospitalization, within 24 h of identification.

### Laboratory Analyses

Laboratory procedures are described in Text S4.

### Roles of Investigators and Sponsor

The study was sponsored by GlaxoSmithKline Biologicals, the vaccine developer and manufacturer. The data generated in the trial are subject to a confidentiality agreement between the investigators and sponsor that allowed the investigators full access to the study data at the end of the study and included an obligation for GlaxoSmithKline Biologicals to permit publication without excessive delay.

### Data Collection and Management

The contract research organizations I3 and Progenitor (currently Encorium) were contracted to perform monitoring, Eurofins Medinet to perform biological sample processing, and S-Clinica to perform blinded statistical analyses. At each study center, data were remotely entered on electronic case report forms and transferred to GlaxoSmithKline for data management. All data cleaning processes were blinded to study group, and analyses before the end of the study were conducted by an external statistician (from S-Clinica, Belgium) who performed the analyses using SAS and the StatXact 8.0 procedure in SAS based on cleaned datasets and quality control programs provided by GlaxoSmithKline.

### Statistical Analysis

The per-protocol cohort for efficacy comprised participants who had complied with vaccination and did not fulfill elimination criteria. In the per-protocol analysis, follow-up was censored at whatever came first: data lock point, time of last contact (in case of withdrawal), time of unblinding, time of booster vaccination (if the booster dose was not given correctly), or 18 mo of age (if the booster dose was not given by this age). The intent-to-treat analysis assessed efficacy against disease episodes from administration of the first vaccine dose.

The incidence (percentage) of first disease episodes was calculated as the number of first episodes during the respective follow-up period divided by *n*, multiplied by 100. VE was estimated as one minus the hazard ratio and obtained, with its 95% CI, from a Cox regression model based on time to first disease episode. The Cox model was applied for VE and associated 95% CI and *p*-value computations when at least one event was observed in each group. Where zero cases were observed in one or more groups, the Poisson model, conditional to the number of cases, was applied without specific correction.

#### Primary outcome

The primary confirmatory objective was to demonstrate VE of PHiD-CV vaccination (three doses in children aged ≤18 mo or four doses if aged >18 mo) against first episodes of B-CAP occurring at least 2 wk after administration of the third dose of PHiD-CV in the per-protocol cohort for efficacy. The study was designed to detect a VE of 20% at study end, but allowed an earlier efficacy conclusion if a higher VE was achieved at the time of the interim analysis. As defined in the study protocol, the interim analysis was planned to occur at least 18 mo after study start, once 535 first B-CAP episodes were identified.

A sample size of 24,000 healthy infants was planned to allow 21,600 evaluable children for the per-protocol CAP efficacy analysis. Power for B-CAP efficacy at the interim analysis and at the final analysis was calculated according to various VE assumptions and numbers of first B-CAP cases. With 535 first B-CAP cases and assuming a true VE of at least 25%, the study had 87.6% power to be conclusive at the interim analysis considering an adjusted one-sided alpha level of 1.75%.

At the interim analysis, the primary objective was considered to be conclusive and final if the one-sided nominal *p*-value for the null hypothesis (B-CAP VE ≤ 0%) was lower than the adjusted (Pocock adjustment) one-sided significance level of 1.75% (*p*<0.0175), a value accounting for the possibility of a second analysis of the primary end point if the first was inconclusive (East, version 2.0, survival module: one-sided tests with early rejection of null hypothesis only; Cytel). The interim analysis was performed by an independent statistician to maintain blinding for the sponsor. The results were first shared with the IDMC, which, following agreement on the conclusions, sent them to the study sponsor.

After the interim analysis was performed, issues were discovered in Panama in relation to informed consent for some children (see below and Text S1). Corrective actions were implemented, with notification of the ethics committees and IDMC. Deficiencies in the informed consent process led to exclusion of some children from analyses, or where analyses had already been performed without awareness of the deficiencies, these deficiencies were addressed by performing a sensitivity analysis to investigate the impact on study outcomes of the use of data from children for whom deficiencies were noted and could not be corrected. Specifically, for 60 children, the parent was a minor at the time of providing initial informed consent, and re-consent once the parent was aged 18 y could not be obtained, and for 53 children parental age was unknown. Therefore, in line with guidance from the European Medicines Agency [39],[40], a descriptive sensitivity analysis was conducted on the primary objective in which children with informed consent issues were excluded to confirm the validity of the interim analysis. Children for whom the original signed informed consent form was missing (*n* = 31) were also excluded from this sensitivity analysis, which was performed by an independent statistician on the database used for the interim analysis.

#### First secondary outcome

For the sequential secondary confirmatory objective (efficacy against C-AOM at the end of the study; not part of interim analysis), the number of first C-AOM episodes required to show VE>0% with 80% and 90% power (non-adjusted one-sided test, nominal type I error of 2.5%) was calculated according to various true VE assumptions. If VE was 20%, 662 and 880 first C-AOM episodes were required to demonstrate positive VE with 80% and 90% power, respectively. If VE was 15%, 1,226 and 1,637 first C-AOM episodes were required for 80% and 90% power, respectively. Since the objective of the trial was to demonstrate VE in terms of a lower incidence of B-CAP or C-AOM in the vaccine group compared to the control group, only comparison in one direction was deemed necessary, hence the use of a one-sided statistical hypothesis linked to VE (with associated one-sided alpha level).

The first secondary objective (to demonstrate VE of PHiD-CV against first C-AOM episodes) was considered significant if the primary objective was significant and the one-sided *p*-value for VE against C-AOM was <0.025, allowing for control of the primary confirmatory B-CAP objective and the sequential secondary confirmatory C-AOM objective.

#### Other secondary outcomes and safety

Other analyses, including VE against first IPD episodes, were descriptive, and therefore there was no adjustment for multiplicity for the associated informative *p*-values. Interpretations of nonoverlapping 95% CI boundaries should therefore be made with caution, as no predefined criteria were fixed, with no formal control of the alpha level. A two-sided Schoenfeld residual test for the null hypothesis that the VE is homogeneous over the age/time range was performed in order to assess the proportionality assumption associated with the Cox model.

An analysis of VE against consolidated CAP was conducted among children who received the booster vaccine dose; VE was calculated for cases reported before the booster dose, given at 15–18 mo of age, and for cases reported after the booster dose. In another subanalysis, AOM severity was categorized according to the five symptoms on the Dagan scale. Cases were analyzed for which the presence or absence of each of the five symptoms had been recorded. In addition, a second complementary analysis of severity was conducted, considering cases that had at least one symptom score recorded, with the assumption that unrecorded symptoms were not observed.

Immunogenicity analyses are reported for the per-protocol cohort for immunogenicity, defined as vaccinated children who met all eligibility criteria, complied with protocol-defined procedures, and had at least one antibody assay result available. An incorrect version of the informed consent form was used to obtain consent for children in the immunogenicity group in Panama (see Text S1). When the error was detected, in agreement with the independent ethics committee, 501 parents/guardians were recontacted to confirm their agreement to the use of the immunogenicity data of their child/ward. For 262 children, parents/guardians could not be contacted to provide consent or did not agree to the use of immunogenicity data. In addition, two children were excluded because the original informed consent forms were lost during the re-monitoring activities. Therefore, 264 children were excluded from the intent-to-treat cohort for immunogenicity (Figure S1). Seropositivity rates, ELISA geometric mean antibody concentrations, and geometric mean OPA titers were calculated with 95% CIs. Safety analyses (including mortality) were performed on the intent-to-treat cohort.

## Results

### Trial Profile

**Figure 3:**
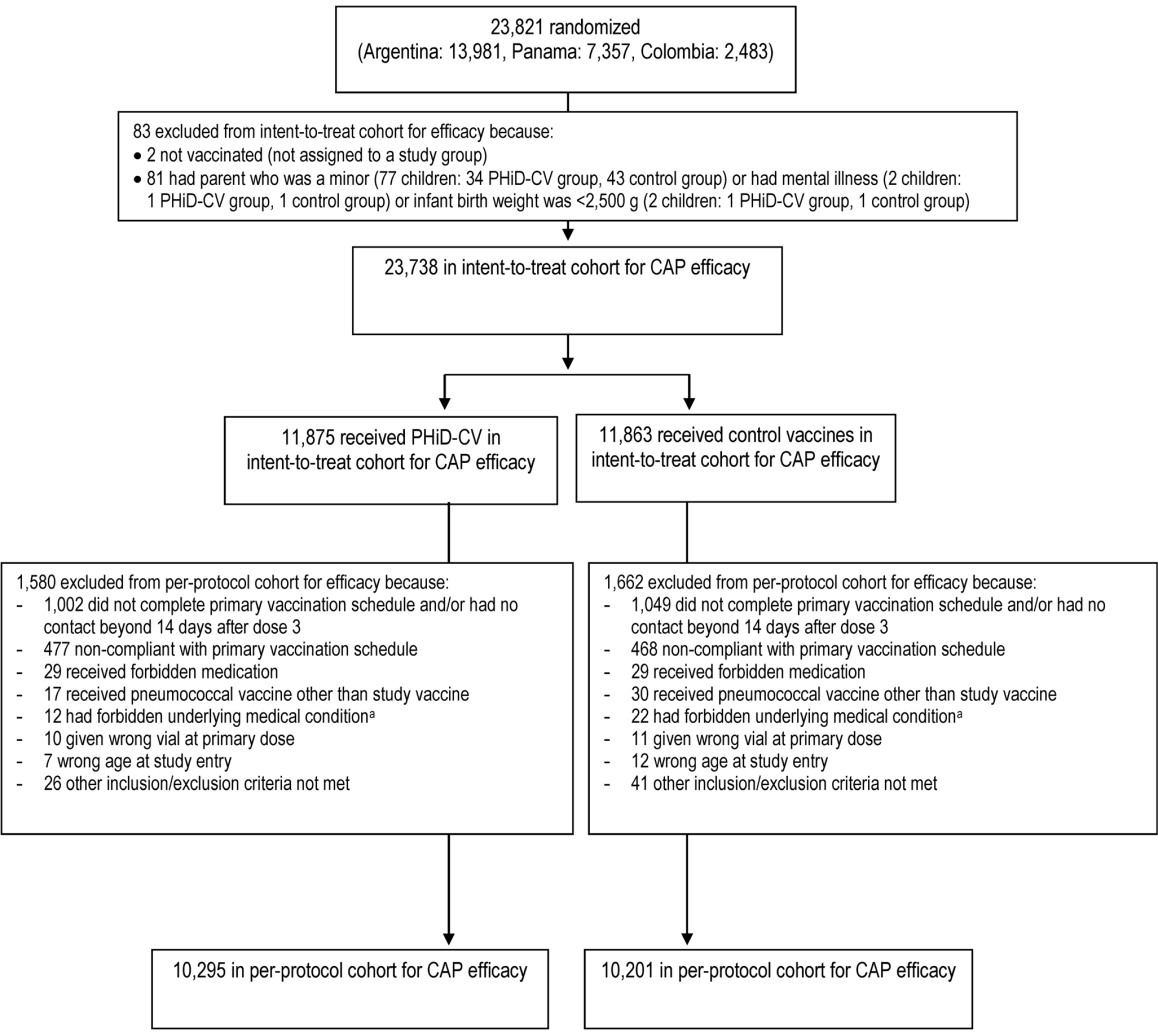
Trial profile for children included in the analysis of the primary study end point. Elimination criteria shown for one reason only, although more than one reason for elimination could apply per child. ^a^Forbidden underlying medical conditions included, but were not limited to, major congenital defects, serious chronic illness, or confirmed or suspected immunosuppressive or immunodeficient conditions.

**Figure 4:**
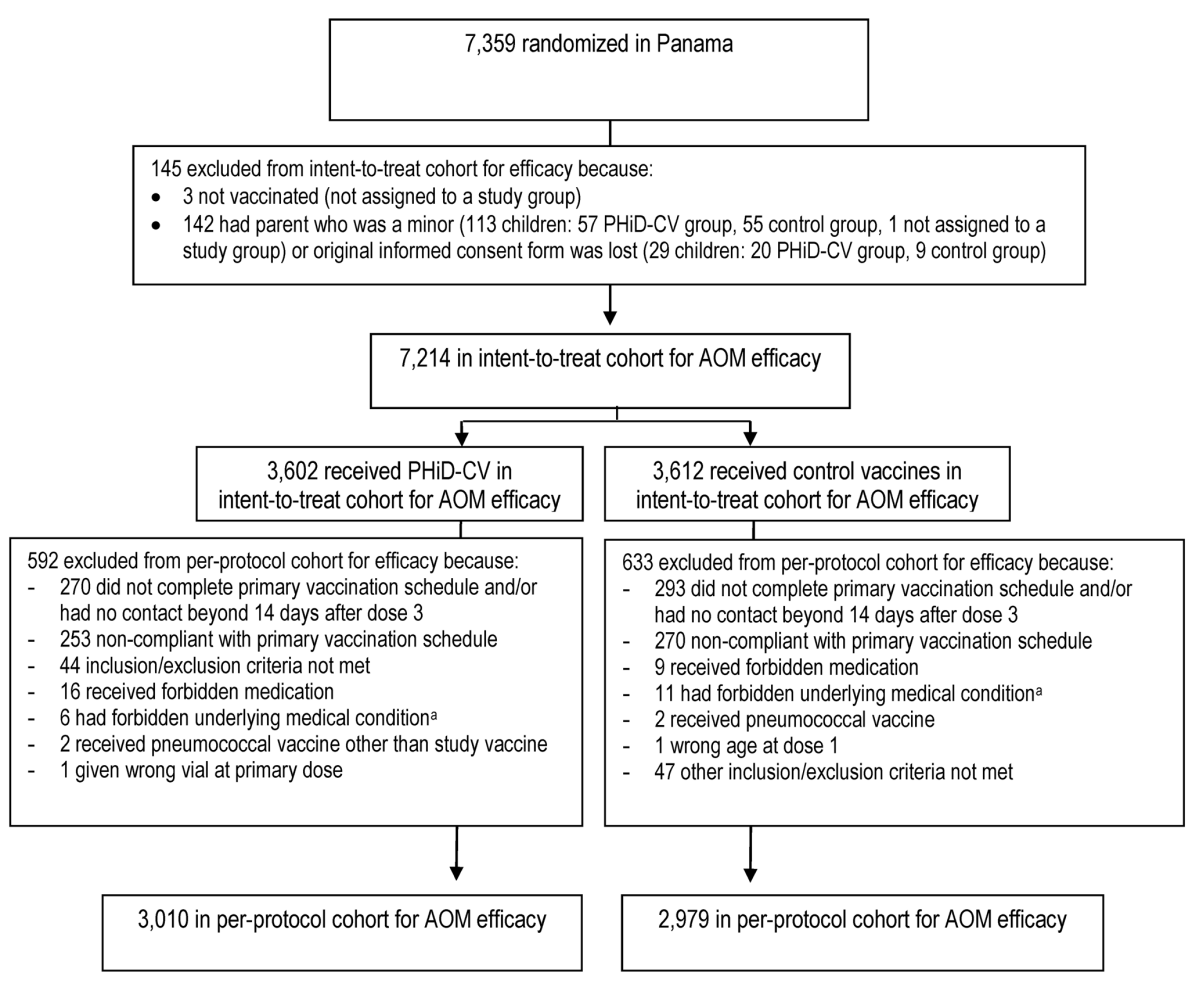
Trial profile for children included in the end-of-study analysis of acute otitis media. Elimination criteria shown for one reason only, although more than one reason for elimination could apply per child. ^a^Forbidden underlying medical conditions included, but were not limited to, major congenital defects, serious chronic illness, or confirmed or suspected immunosuppressive or immunodeficient conditions.

The numbers of children enrolled, randomized, and eligible for primary end point analysis are shown in Figure 3, and end-of-study cohort numbers are provided in Figures 4 and S2. Demographic characteristics of the groups were well balanced (Table 3). As shown in the study timeline (Figure S3), the data lock point for the interim CAP efficacy analysis was August 31, 2010, and the study ended on July 28, 2011 (last child, last contact).

**Table 3:** Characteristics of the trial participants included in the analysis of the primary study end point (CAP efficacy cohort; interim analysis), CAP and IPD end-of-study analysis (CAP/IPD efficacy cohort), and AOM end-of-study analysis (AOM efficacy cohort).

Efficacy Analysis Population	Characteristic	Category	Intent-to-Treat Cohort		Per-Protocol Cohort	
			PHiD-CV Group	Control Group	PHiD-CV Group	Control Group
**CAP interim**			*n* = 11,875	*n* = 11,863	*n* = 10,295	*n* = 10,201
	**Mean age ± SD**	At dose 1 (weeks)	9.2±1.9	9.2±1.9	9.2±1.9	9.2±1.9
		At booster dose (months)	16.1±1.6	16.1±1.6	16.1±1.6	16.1±1.6
	**Sex, ** ***n*** ** (percent)**	Female	5,826 (49.1)	5,801 (48.9)	5,072 (49.3)	4,987 (48.9)
		Male	6,049 (50.9)	6,062 (51.1)	5,223 (50.7)	5,214 (51.1)
	**Race** a **, ** ***n*** ** (percent)**	White	6,757 (56.9)	6,751 (56.9)	5,958 (57.9)	5,918 (58.0)
		Other or mixed race	5,118 (43.1)	5,112 (43.1)	4,337 (42.1)	4,283 (42.0)
	**Follow-up time** b	Sum of time to first B-CAP (years)	25,516	25,329	19,513	19,260
**CAP/IPD end of study**			*n* = 11,798	*n* = 11,799	*n* = 10,211	*n* = 10,140
	**Mean age ± SD**	At dose 1 (weeks)	9.2±1.9	9.2±1.9	9.2±1.9	9.2±1.9
		At booster dose (months)	16.1±1.6	16.1±1.6	16.1±1.6	16.1±1.6
	**Sex, ** ***n*** ** (percent)**	Female	5,796 (49.1)	5,767 (48.9)	5,040 (49.4)	4,947 (48.8)
		Male	6,002 (50.9)	6,032 (51.1)	5,171 (50.6)	5,193 (51.2)
	**Race** a **, ** ***n*** ** (percent)**	White	6,756 (57.3)	6,751 (57.2)	5,950 (58.3)	5,909 (58.3)
		Other or mixed race	5,042 (42.7)	5,048 (42.8)	4,261 (41.7)	4,231 (41.7)
	**Follow-up time** b	Sum of time to first B-CAP (years)	31,480	31,265	24,821	24,545
		Sum of time to first IPD (years)	32,117	32,023	25,244	25,043
**AOM end of study** c			*n* = 3,602	*n* = 3,612	*n* = 3,010	*n* = 2,979
	**Mean age ± SD**	At dose 1 (weeks)	9.0±1.3	9.0±1.3	9.0±1.3	9.0±1.3
		At booster dose (months)	15.8±1.8	15.8±2.0	15.7±1.7	15.7±1.8
	**Sex, ** ***n*** ** (percent)**	Female	1,762 (48.9)	1,775 (49.1)	1,478 (49.1)	1,464 (49.1)
		Male	1,840 (51.1)	1,837 (50.9)	1,532 (50.9)	1,515 (50.9)
	**Race, ** ***n*** ** (percent)**	Other or mixed race	3,588 (99.6)	3,597 (99.6)	2,998 (99.6)	2,966 (99.6)
		White	14 (0.4)	15 (0.4)	12 (0.4)	13 (0.4)
	**Follow-up time** b	Sum of time to first C-AOM (years)	9,018	8,835	6,720	6,605

a 59% of participants were recruited in Argentina (race predominantly white or with European heritage), and the remaining were recruited in Colombia and Panama (participants predominantly mixed race).

b Follow-up time calculated as sum of follow-up periods of each child, expressed in years, censored at the first occurrence of a respective end point event.

c Recruited in Panama only.

SD, standard deviation.

The sum of the follow-up period, censored at the first occurrence of a respective end point event in each group, is provided in Table 3. For the intent-to-treat analysis, mean duration of follow-up was 26 mo for the interim CAP analysis, 33 mo for the end-of-study CAP/IPD analysis, and 31 mo for the end-of-study AOM analysis. For the per-protocol analysis, mean duration of follow-up was 23, 30, and 28 mo, respectively.

### Disease Reporting and Vaccine Efficacy

#### Primary outcome and other CAP outcomes

Pneumonia rates for the two groups are presented in Table 4. Fewer than 1.5% of X-rays in the study were categorized as non-interpretable. At least one suspected CAP episode was reported in approximately 20% of all children. Around one-third of suspected CAP cases were radiologically confirmed (including perihilar infiltrates and other abnormal findings), of which approximately 30% were WHO-defined consolidated CAP and 40% were B-CAP (WHO-defined consolidated CAP, or nonconsolidated CAP with CRP ≥ 40 µg/ml).

**Table 4:** Efficacy of PHiD-CV against first community-acquired pneumonia and invasive pneumococcal disease episodes.

Cohort/Case Definition	Intent-to-Treat Analysis					Per-Protocol Analysis				
	PHiD-CV Group		Control Group		VE, Percent (95% CI)	PHiD-CV Group		Control Group		VE, Percent (95% CI)
	Number of First Episodes	Incidence, Percent (95% CI)^a^	Number of First Episodes	Incidence, Percent (95% CI)^a^		Number of First Episodes	Incidence, Percent (95% CI)^a^	Number of First Episodes	Incidence, Percent (95% CI)^a^	
**CAP interim**	*n* = 11,875		*n* = 11,863			*n* = 10,295		*n* = 10,201		
Consolidated CAP	223	1.9 (1.6, 2.1)	289	2.4 (2.2, 2.7)	23.4 (8.8, 35.7)	155	1.5 (1.3, 1.8)	206	2.0 (1.8, 2.3)	25.7 (8.4, 39.6)
B-CAP	341	2.9 (2.6, 3.2)	414	3.5 (3.2, 3.8)	18.2 (5.5, 29.1)	240	2.3 (2.0, 2.6)	304	3.0 (2.7, 3.3)	22.0 (7.7, 34.2)^b^
Radiologically confirmed CAP	854	7.2 (6.7, 7.7)	947	8.0 (7.5, 8.5)	10.5 (1.8, 18.4)	625	6.1 (5.6, 6.5)	711	7.0 (6.5, 7.5)	13.3 (3.4, 22.1)
Suspected CAP	2,455	20.7 (19.9, 21.4)	2,616	22.1 (21.3, 22.8)	7.3 (2.1, 12.3)	1,916	18.6 (17.9, 19.4)	2,019	19.8 (19.0, 20.6)	6.7 (0.7, 12.3)
**CAP/IPD end of study**	*n* = 11,798		*n* = 11,799			*n* = 10,211		*n* = 10,140		
**CAP**										
Consolidated CAP	251	2.1 (1.9, 2.4)	319	2.7 (2.4, 3.0)	21.8 (7.7, 33.7)	181	1.8 (1.5, 2.0)	231	2.3 (2.0, 2.6)	22.4 (5.7, 36.1)
B-CAP	377	3.2 (2.9, 3.5)	450	3.8 (3.5, 4.2)	16.7 (4.5, 27.4)	275	2.7 (2.4, 3.0)	333	3.3 (2.9, 3.6)	18.2 (4.1, 30.3)
Radiologically confirmed CAP	919	7.8 (7.3, 8.3)	1,015	8.6 (8.1, 9.1)	10.0 (1.7, 17.7)	681	6.7 (6.2, 7.2)	764	7.5 (7.0, 8.1)	11.9 (2.3, 20.5)
Suspected CAP	2,667	22.6 (21.9, 23.4)	2,880	24.4 (23.6, 25.2)	8.7 (3.8, 13.4)	2,108	20.6 (19.9, 21.4)	2,237	22.1 (21.3, 22.9)	7.3 (1.6, 12.6)
**IPD** c										
Culture-confirmed	7	0.1 (0.0, 0.1)	21	0.2 (0.1, 0.3)	66.7 (21.8, 85.9)	6	0.1 (0.0, 0.1)	17	0.2 (0.1, 0.3)	65.0 (11.1, 86.2)
Vaccine serotypes	0	0.0 (0.0, 0.0)	18	0.1 (0.1, 0.2)	100 (77.3, 100)	0	0.0 (0.0, 0.0)	16	0.2 (0.1, 0.3)	100 (74.3, 100)
Cross-reactive serotypes^d^	2	0.0 (0.0, 0.1)	1	0.0 (0.0, 0.0)	−99.5 (−2,100.2, 81.9)	2	0.0 (0.0, 0.1)	1	0.0 (0.0, 0.1)	−98.6 (−2,089.5, 82.0)
Other serotypes	4	0.0 (0.0, 0.1)	2	0.0 (0.0, 0.1)	−99.5 (−989.2, 63.5)	3	0.0 (0.0, 0.1)	0	0.0 (0.0, 0.0)	NC
Not serotyped	1	0.0 (0.0, 0.0)	0	0.0 (0.0, 0.0)	NC	1	0.0 (0.0, 0.1)	0	0.0 (0.0, 0.0)	NC

VE estimated as one minus the hazard ratio and obtained, with its 95% CI, from a Cox regression model based on time to first episode when at least one event was observed in each group and conditional on number of cases when no case in at least one group (i.e., VE equal to zero or −infinite (VE, 1 − [*x*/0]).

a Number of first episodes during the respective follow-up period divided by *n*, multiplied by 100.

b Significant *p*-value (*p* = 0.002); one-sided *p*-value from Cox regression model to test null hypothesis VE ≤ 0% with one-sided alpha of 1.75%.

c *S. pneumoniae* was isolated from all culture-confirmed invasive disease cases.

d Pneumococcal serotype 6A, 9N, or 19A.

NC, not calculable.

VE against first B-CAP episodes at interim analysis was 22.0% (95% CI: 7.7%, 34.2%) and met prespecified criteria for conclusive results of the primary objective (*p* = 0.002). VE was also observed against the secondary CAP end points, with consistent results between the intent-to-treat and per-protocol analyses for the interim and end-of-study time points (Table 4). VE against WHO-defined consolidated CAP at end of study was 21.8% (95% CI: 7.7%, 33.7%) and 22.4% (95% CI: 5.7%, 36.1%) in the intent-to-treat and per-protocol analyses, respectively (Table 4).

In the sensitivity analysis of the primary objective (see Methods), 144 children were excluded out of 20,496 children. The results of this analysis showed 239 first B-CAP cases in the PHiD-CV group and 304 in the control group. VE against first B-CAP episodes was 22.3% (95% CI: 7.9%, 34.4%; *p* = 0.002), which was consistent with the final per-protocol CAP efficacy analysis result.

Schoenfeld residual analysis did not indicate heterogeneity of VE over the entire follow-up period. In line with this test, in the intent-to-treat analysis, VE against consolidated CAP was 21.6% (95% CI: −1.4%, 39.5%) among children aged less than 12 mo, 26.7% (95% CI: 2.3%, 45.0%) in children aged between 12 and 24 mo, and 21.3% (95% CI: −12.9%, 45.1%) in children aged between 24 and 36 mo. In the per-protocol analysis these percentages were 15.1% (95% CI: −22.5%, 41.2%), 31.8% (95% CI: 7.2%, 49.9%), and 20.7% (95% CI: −15.9%, 45.8%), respectively. VE against consolidated CAP was 21.7% (95% CI: −0.5%, 38.9%) prior to the booster dose and 26.3% (95% CI: 5.9%, 42.3%) after booster vaccination in the intent-to-treat analysis. In the per-protocol analysis, VE was 15.1% (95% CI: −15.6%, 37.6%) and 26.3% (95% CI: 4.4%, 43.2%) pre- and post-booster, respectively.

#### First secondary outcome and other AOM outcomes

At least one C-AOM episode was reported in approximately 7% of children (Table 5). VE against C-AOM was 16.1% (95% CI: −1.1%, 30.4%) in the per-protocol analysis (*p* = 0.032) and 19.0% (95% CI: 4.4%, 31.4%) in the intent-to-treat analysis (*p* = 0.007). VE against pneumococcal AOM and vaccine serotype AOM was 56.1% (95% CI: 13.4%, 77.8%) and 67.1% (95% CI: 17.0%, 86.9%), respectively, in the per-protocol analysis, with consistent results in the intent-to-treat analysis (Table 5). Among cases for whom the presence or absence of all five symptoms on the Dagan scale was recorded (approximately 80% of all C-AOM cases), 46% were categorized as mild, with few (9%) severe cases in both the intent-to-treat and per-protocol analyses. Among all C-AOM cases that had at least one symptom recorded as present or absent on the Dagan scale, 51% and 52% were mild in the intent-to-treat and per-protocol analyses, respectively, and 8% and 7%, respectively, were severe. Serotype 19F was the most common vaccine serotype isolated from middle ear fluid (Tables S3 and S4). Among the cases of AOM with *H. influenzae*, all but one were nontypable *H. influenzae* (Table 5).

**Table 5:** Efficacy of PHiD-CV against first acute otitis media episodes (end-of-study analysis).

Case Definition	Intent-to-Treat Analysis					Per-Protocol Analysis				
	PHiD-CV Group (*n* = 3,602)		Control Group (*n* = 3,612)		VE, Percent (95% CI)	PHiD-CV Group (*n* = 3,010)		Control Group (*n* = 2,979)		VE, Percent (95% CI)
	Number of First Episodes	Incidence, Percent (95% CI)^a^	Number of First Episodes	Incidence, Percent (95% CI)^a^		Number of First Episodes	Incidence, Percent (95% CI)^a^	Number of First Episodes	Incidence, Percent (95% CI)^a^	
C-AOM	254	7.1 (6.2, 7.9)	308	8.5 (7.6, 9.5)	19.0 (4.4, 31.4)	204	6.8 (5.9, 7.7)	239	8.0 (7.1, 9.1)	16.1 (−1.1, 30.4)
Culture-confirmed C-AOM	45	1.3 (0.9, 1.7)	67	1.9 (1.4, 2.4)	33.6 (3.2, 54.5)	32	1.1 (0.7, 1.5)	45	1.5 (1.1, 2.0)	29.9 (−10.4, 55.4)
Pneumococcal C-AOM	17	0.5 (0.3, 0.8)	38	1.1 (0.8, 1.4)	55.7 (21.5, 75.0)	12	0.4 (0.2, 0.7)	27	0.9 (0.6, 1.3)	56.1 (13.4, 77.8)
Vaccine serotype C-AOM	7	0.2 (0.1, 0.4)	23	0.6 (0.4, 1.0)	69.9 (29.8, 87.1)	6	0.2 (0.1, 0.4)	18	0.6 (0.4, 1.0)	67.1 (17.0, 86.9)
Cross-reactive serotype AOM^b^	5	0.1 (0.1, 0.3)	7	0.2 (0.1, 0.4)	29.0 (−123.7, 77.5)	3	0.1 (0.0, 0.3)	4	0.1 (0.0, 0.3)	25.7 (−232.2, 83.4)
Other serotype C-AOM	6	0.2 (0.1, 0.4)	7	0.2 (0.1, 0.4)	14.8 (−153.7, 71.4)	3	0.1 (0.0, 0.3)	4	0.1 (0.0, 0.3)	25.7 (−231.9, 83.4)
*H. influenzae* C-AOM	20	0.6 (0.3, 0.9)	24	0.7 (0.4, 1.0)	17.3 (−49.8, 54.3)	12	0.4 (0.2, 0.7)	14	0.5 (0.3, 0.8)	15.0 (−83.8, 60.7)
Nontypable *H. influenzae* C-AOM	19	0.5 (0.3, 0.8)	24	0.7 (0.4, 1.0)	21.5 (−43.4, 57.0)	12	0.4 (0.2, 0.7)	14	0.5 (0.3, 0.8)	15.0 (−83.8, 60.7)

VE estimated as one minus the hazard ratio and obtained, with its 95% CI, from a Cox regression model based on time to first episode.

a Number of first episodes during the respective follow-up period divided by *n*, multiplied by 100.

b Pneumococcal serotype 6A, 18B, 19A, or 23A.

#### IPD outcomes

Most of the culture-confirmed IPD cases were caused by vaccine serotypes (Table 4). In the per-protocol analysis, VE was 100% (95% CI: 74.3%, 100%) against IPD caused by vaccine serotypes and 65.0% (95% CI: 11.1%, 86.2%) against any IPD. Pneumococcal serotype 14 was the most commonly isolated IPD serotype (Tables S5 and S6).

### Immunogenicity

The per-protocol immunogenicity cohort included 334 and 331 children in the PHiD-CV and control groups for primary vaccination, respectively, and 232 and 215 children, respectively, for booster vaccination. As shown in Figure S1, the difference in the number of children between the primary and booster vaccination time points was mainly due to noncompliance with booster vaccination or blood sampling schedules (visits outside of scheduled intervals in the protocol, predominantly as a result of social disruptions caused by the H1N1 influenza pandemic). In the per-protocol immunogenicity cohort, for each of the ten vaccine pneumococcal serotypes, the percentage of children with antibody concentration ≥ 0.2 µg/ml after PHiD-CV primary vaccination was at least 93.1%, and the percentage with OPA titer ≥ 8 was at least 90.8% (Table S7). Robust immune responses were observed after booster vaccination (Table S7). For the cross-reactive serotypes 6A and 19A, at least 85.4% of children had an antibody concentration ≥ 0.2 µg/ml, and at least 80.5% had an OPA titer ≥ 8 after booster vaccination. Anti–protein D antibody geometric mean antibody concentrations following PHiD-CV vaccination were 2,455 ELU/ml (95% CI: 2,248, 2,681) after primary vaccination and 2,787 ELU/ml (95% CI: 2,436, 3,189) after booster vaccination, compared to 101 ELU/ml (95% CI: 91, 112) and 92 ELU/ml (95% CI: 83, 103), respectively, in the control group. The immunogenicity results of the intent-to-treat cohort for immunogenicity were consistent with those of the primary and booster per-protocol cohorts for immunogenicity (data not shown).

### Serious Adverse Events

SAEs were reported for 21.5% (95% CI: 20.7%, 22.2%) of PHiD-CV recipients and 22.6% (95% CI: 21.9%, 23.4%) of children in the control group (Tables 6 and S8). One SAE, an apparent life-threatening event in the control group, was considered by the investigator to be causally related to vaccination. It occurred on the day of the second hepatitis B vaccine and DTPa-IPV/Hib vaccine dose administration, and resolved without sequelae.

**Table 6:** Serious adverse events reported from study start and administration of the first vaccine dose up to study end in at least 1.0% of children (intent-to-treat cohort: all children).

SAEs Reported in ≥1.0% of Children, *n* (Percent)	PHiD-CV Group (*n* = 11,798)	Control Group (*n* = 11,799)
Gastroenteritis	553 (4.7)	497 (4.2)
Pneumonia	478 (4.1)	557 (4.7)
Bronchiolitis	473 (4.0)	518 (4.4)
Dehydration	463 (3.9)	438 (3.7)
Asthmatic crisis	192 (1.6)	210 (1.8)
Bronchial obstruction	127 (1.1)	141 (1.2)
Bronchitis	124 (1.1)	129 (1.1)
Febrile convulsion	95 (0.8)	135 (1.1)
Any SAE(s)	2,534 (21.5)	2,668 (22.6)

A full listing of SAEs is provided in Table S8.

### Mortality

Nineteen deaths among 11,798 children (0.16%; 95% CI: 0.10%, 0.25%) were reported in the PHiD-CV group, and 26 deaths among 11,799 children (0.22%; 95% CI: 0.14%, 0.32%) were reported in the control group. This suggested that PHiD-CV vaccination reduced all-cause mortality by 27.0% (95% CI: −31.8%, 59.6%), although this difference was not statistically significant. The majority of the reported fatal cases (nine in the PHiD-CV group, 16 in the control group) involved children aged less than 1 y. None of the deaths were considered by the investigator to be causally related to vaccination.

## Discussion

Efficacy of PHiD-CV was shown against a variety of CAP, AOM, and IPD end points, with consistent results between the per-protocol and intent-to-treat analyses. In the interim analysis, VE against first episodes of B-CAP was 22.0% and therefore (as predefined in the protocol) was considered conclusive for the primary objective. In this analysis, efficacy against WHO-defined consolidated CAP was 25.7%. In the end-of-study per-protocol analyses, VE was 16.1% against C-AOM, 67.1% against vaccine pneumococcal serotype C-AOM, 100% against vaccine serotype IPD, and 65.0% against IPD of any serotype.

This study thereby provides a comprehensive assessment of clinical protection of PHiD-CV against invasive and mucosal infections in Latin American children. In addition, despite the lower immunogenicity of PHiD-CV compared to 7vCRM, the antibody levels achieved were sufficient to provide a level of protection against WHO-defined CAP with alveolar consolidation (22–26%) that was consistent with the 20–37% efficacy reported in previous studies with other PCVs, including 7vCRM [41]–[45]. It should be noted that these studies were conducted with vaccine candidates of different serotype valencies and carrier proteins (seven- and nine-valent CRM_197_-conjugated vaccines, and 11-valent vaccine containing tetanus toxoid and diphtheria toxoid carrier proteins), and took place in a variety of geographic and socioeconomic settings.

In our study, VE against WHO-defined consolidated CAP among children aged 12–24 mo and 24–36 mo remained above 20%, which was consistent with results obtained with 9vCRM in the Gambia [42] and South Africa [46] but differed from studies of 7vCRM in the US [41] and an 11-valent vaccine in the Philippines [44]. In the latter two studies there was evidence of diminished efficacy already in the second year of life. The reasons for waning VE in some studies but not others are unknown but might be associated with inconsistent use of booster vaccination between studies, differences in strength of natural boosting between populations, or proportional increases in pneumonia caused by pathogens other than *S. pneumoniae* or by non-vaccine serotypes [41]. It should be noted that, with the descriptive analyses of VE per age subgroup and pre-/post-booster dose, it is difficult to disentangle the effect of age from the effect of the booster dose.

The number of AOM cases identified in Panama was lower than expected, and various factors might have contributed to this outcome. Case detection was especially low during the first 2 y of the study, and by the time it was enhanced, the age period of expected peak in AOM incidence [47] had likely passed for most participants. The requirement for evaluation by a pediatrician followed by further assessment by the ENT specialist and exclusion of children at high risk for pneumococcal infection could also have contributed. Some parents might have been reluctant to seek medical advice because of perceptions of AOM as a usually mild disease or might have obtained care from nearby pharmacies or clinics not involved in the study.

Regardless of the factors responsible, the relatively low incidence of AOM may help with interpretation of the C-AOM protection results since, in the prespecified primary outcome (the per-protocol analysis), the lower limit of the 95% CI around the 16.1% point estimate was slightly below zero (95% CI: −1.1%, 30.4%). Analysis of the intent-to-treat cohort gave a similar point estimate (19.0%) but with a 95% CI lower limit above zero (95% CI: 4.4%, 31.4%). This suggests that the lack of statistical significance in the per-protocol analysis was due to a lack of cases rather than to true lack of efficacy.

Nevertheless, we consider these results as highly relevant to other settings, as the AOM cases had both pediatrician and ENT confirmation, and around half of the C-AOM episodes were mild, with the remainder being moderate or severe, thus representing the broad spectrum of clinical AOM. In any case, differences in case definitions contribute little to differences in PCV efficacy estimates against AOM [48]. Notably, VE against AOM episodes caused by vaccine serotypes in our study was similar to that observed in two European studies, the Finnish Otitis Media (FinOM) trial of two seven-valent PCVs (one investigational) and the Pneumococcal Otitis Efficacy Trial (POET) of the 11-valent predecessor to PHiD-CV conducted in the Czech Republic and Slovakia [49],[50]. We note that the most common serotypes causing AOM in both Latin America and Europe are members of serogroups 6, 14, 19, and 23 [5],[51].

Similar to the Pneumococcal Otitis Efficacy Trial and in contrast to the Finnish Otitis Media trial, VE against AOM caused by nontypable *H. influenzae* in COMPAS suggested a beneficial effect, although the result was not significant (95% CI included zero). It should be noted that the COMPAS study was not powered to assess the nontypable *H. influenzae* end point. Also similar to the Pneumococcal Otitis Efficacy Trial, there was no evidence for replacement of vaccine serotypes with non-vaccine pneumococcal serotypes or other otopathogens during 31 mo of intent-to-treat follow-up for AOM analysis; in contrast, evidence of replacement disease was found early in the follow-up of the Finnish Otitis Media trial with 7vCRM, as of 2 mo of age [52]. Overall, as AOM is a common disease [47], the 16%–19% VE observed against C-AOM represents substantial reductions in AOM case numbers, from both public health and health care cost perspectives. In previous double-blind randomized trials of PCVs (all seven-valent) administered in infancy, VE estimates against clinical AOM were 0%–7% [53] and appeared unaffected by population variability [54]. With the 11-valent protein D conjugate vaccine, VE against clinical AOM was 34% [50]. However, comparisons of C-AOM results among trials are complicated by differences in care-seeking and case detection methods, as well as disease burden.

All invasive disease cases were first episodes and pneumococcal, and, as anticipated from the regional epidemiology of pneumococcal serotypes [4], most were caused by vaccine serotypes. The results presented here are similar to those obtained in a recently published cluster-randomized double-blind trial conducted in a different socioeconomic setting, Finland. There, PHiD-CV was highly effective against IPD when given either in a 3+1 or 2+1 vaccination schedule to infants or as a two-dose catch-up to children in the second year of life [55]. As in COMPAS, following the 3+1 PHiD-CV schedule, VE against IPD caused by any vaccine serotype was 100% in the Finnish study. This point estimate is similar to or slightly higher than those observed in studies of other PCVs against IPD [42],[43],[56],[57].

One limitation of this study, as noted earlier, lies in the low number of reported AOM cases, despite efforts to improve surveillance during the study. Another limitation was the study setting being in mostly urban areas, potentially affecting its generalizability to rural populations. This setting was chosen to aid recruitment and to improve the likelihood of capturing all pneumonia cases, as that might have been difficult in rural areas without easy access to health care centers and pediatric hospitals. An additional study limitation was that approximately 14% of participants were excluded from the per-protocol efficacy analyses, mainly because of early withdrawal and noncompliance with the primary vaccination schedule in both groups. This was predominantly as a result of adverse media coverage of the study in 2007/2008 linked to unfounded rumors of a causal relationship between PHiD-CV vaccination and infant mortality [58]. Subsequent investigations of the blinded safety results were conducted by the IDMC and local regulatory authorities. Although these concluded that the study could continue as planned, many parents became unwilling to allow their children to continue to participate.

Nonetheless, these exclusions do not appear to have affected the robustness of the study, as VE results were consistent between the intent-to-treat and per-protocol analyses. Importantly, a 27% reduction (not statistically significant) in overall mortality in PHiD-CV recipients was observed, which was most evident in children in the first year of life, when the risk of severe pneumococcal disease is highest [2]. Reductions in all-cause mortality reported in other pneumococcal conjugate VE studies in young children, both with a nine-valent vaccine, were 14%–16% in the Gambia [42] and 5% in South Africa [43].

In conclusion, COMPAS is, to our knowledge, the first double-blind randomized controlled trial to assess pneumococcal conjugate VE in Latin America, a region with an intermediate pneumococcal disease burden. The results of this study demonstrate the efficacy of PHiD-CV and document the magnitude of its impact against CAP and AOM, which are mucosal diseases of public health importance, commonly encountered in young children in clinical practice.

## Supporting Information

Figure S1 **Number of children in the primary or booster vaccination intent-to-treat and per-protocol cohorts for immunogenicity.**
(DOCX)

Figure S2 **Trial profile for children included in the end-of-study analyses of community-acquired pneumonia and invasive pneumococcal disease.**
(DOCX)

Figure S3 **Clinical Otitis Media and Pneumonia Study timeline.**
(DOCX)

Table S1 **National public health authorities and ethical review committees.**
(DOCX)

Table S2 **Children at high risk of invasive pneumococcal infection.**
(DOCX)

Table S3 **Occurrence of first clinically confirmed acute otitis media episodes (per-protocol cohort for acute otitis media vaccine efficacy analysis).**
(DOCX)

Table S4 **Occurrence of first clinically confirmed acute otitis media episodes (intent-to-treat cohort for acute otitis media vaccine efficacy analysis).**
(DOCX)

Table S5 **Occurrence of invasive pneumococcal infection episodes (per-protocol cohort for community-acquired pneumonia/invasive pneumococcal infection vaccine efficacy analysis).**
(DOCX)

Table S6 **Occurrence of invasive pneumococcal infection episodes (intent-to-treat cohort for community-acquired pneumonia/invasive pneumococcal infection vaccine efficacy analysis).**
(DOCX)

Table S7 **Pneumococcal antibody concentration and opsonophagocytic activity in PHiD-CV group (per-protocol immunogenicity cohort for primary or booster vaccination).**
(DOCX)

Table S8 **Serious adverse events reported from study start and administration of the first vaccine dose up to study end (intent-to-treat cohort: all children).**
(DOCX)

Text S1 **Ethical considerations and informed consent.**
(DOCX)

Text S2 **Trial protocol.**
(PDF)

Text S3 **Major protocol changes.**
(DOCX)

Text S4 **Laboratory analyses.**
(DOCX)

Text S5 **CONSORT checklist.**
(DOCX)

## Abbreviations

7vCRM: seven-valent pneumococcal CRM-conjugate vaccine
AOM: acute otitis media
B-CAP: likely bacterial community-acquired pneumonia
C-AOM: clinically confirmed acute otitis media
CAP: community-acquired pneumonia
COMPAS: Clinical Otitis Media and Pneumonia Study
CRP: C-reactive protein
CSF: cerebrospinal fluid
DTPa-IPV/Hib: diphtheria–tetanus–acellular pertussis–inactivated poliovirus–*Haemophilus influenzae* type b vaccine; ELU, ELISA units
ENT: ear, nose, and throat
IDMC: independent data monitoring committee
IPD: invasive pneumococcal disease
OPA: opsonophagocytic activity
PCV: pneumococcal conjugate vaccine
PHiD-CV: pneumococcal nontypable *Haemophilus influenzae* protein D conjugate vaccine
SAE: serious adverse event
VE: vaccine efficacy
WHO: World Health Organization
