# Correction: Efficacy of Pneumococcal Nontypable *Haemophilus influenzae* Protein D Conjugate Vaccine (PHiD-CV) in Young Latin American Children: A Double-Blind Randomized Controlled Trial

**DOI:** 10.1371/journal.pmed.1001850

**Published:** 2015-06-26

**Authors:** Miguel W. Tregnaghi, Xavier Sáez-Llorens, Pio López, Hector Abate, Enrique Smith, Adriana Pósleman, Arlene Calvo, Digna Wong, Carlos Cortes-Barbosa, Ana Ceballos, Marcelo Tregnaghi, Alexandra Sierra, Mirna Rodriguez, Marisol Troitiño, Carlos Carabajal, Andrea Falaschi, Ana Leandro, Maria Mercedes Castrejón, Alejandro Lepetic, Patricia Lommel, William P. Hausdorff, Dorota Borys, Javier Ruiz Guiñazú, Eduardo Ortega-Barría, Juan P. Yarzábal, Lode Schuerman, COMPAS Group

There is information missing from the Competing Interests statement. While the authors have already declared in the Funding statement that GlaxoSmithKline (GSK) Biologicals SA was the funding source and was involved in all stages of the study conduct and analysis, they did not disclose all potential financial competing interests for each individual co-author related to GSK funding in the original competing interests statement due to a misunderstanding in what was expected from them.

The authors disclose the following additional competing interests:

In relation with the COMPAS study, presented in the article:

Personal fees from GSK for the conduct of the COMPAS study: M.W.T. (as Principal Investigator); M.T., E.S., C.C., H.A., A.P. (Investigators); A.Ce., A.S. (coordinating investigators) and A.F. (sub-investigator). Personal fees from GSK for support to travel: M.W.T., M.T., E.S., C.C., H.A., A.P., D.W., C.C.B., A.Ce., A.S., A.F. Personal fees from GSK for advisory board attendance: M.W.T., H.A., A.Ce. Personal or consulting fees from GSK, through the indicated institution: A.L. (Sub-investigator, INDICASAT); M.R., D.W. (INDICASAT); C.C.B. (CEIP); M.Tro. (INDICASAT/Health research international [HRI]); X.S-L. (Principal Investigator, HRI/Hospital del Nino); A.Ca. (HRI). Support for travel from GSK through the indicated institution: X.S-L. and M.R. A.Ca declares her institution (HRI) has received grant from GSK for the study conduct.

Outside of the work presented in the article:

Personal fees from GSK for the conduct of other study(ies): M.W.T., M.T., E.S., C.C., H.A., A.Ce. Payment from GSK for lectures in meeting and symposia: M.W.T., A.Ce. Payment from GSK for speaker activity: X.S-L., C.C.B. Grants from GSK for the conduct of other studies: P.L. Personal fees from HRI: A.Ca. Personal fees from other pharmaceutical companies: M.W.T. (Novartis, as Principal Investigator); M.T. (Novartis, as Investigator); H.A., A.Ce. (Novartis, for lecture at meeting/symposia); A.S. (MSD, as speaker); A.F. (Thallion, as Principal Investigator).

Employed by and owning stock/stock options of the GSK group of companies: M.M.C., A.Le, P.Lom., W.P.H., D.B., J.R.G., E.O.B., J.P.Y. and L.S. Patent: W.P.H. is a patent co-holder for PCV13 (no royalties).
