# Supplementary material for: Efficacy of Pneumococcal Nontypable Haemophilus influenzae Protein D Conjugate Vaccine (PHiD-CV) in Young Latin American Children: A Double-Blind Randomized Controlled Trial
Source: PLoS Med. 2014 Jun 3;11(6):e1001657. doi: 10.1371/journal.pmed.1001657 (PMC4043495; doi:10.1371/journal.pmed.1001657)
Supplement: Figure S1 — Number of children in the primary or booster vaccination intent-to-treat and per-protocol cohorts for immunogenicity. (DOCX) [file pmed.1001657.s001.docx]

**Figure S1 Number of children in the primary or booster vaccination intent-to-treat (ITT) and per-protocol cohorts for immunogenicity**

**23,823 children randomized**

**Control Group, N = 500**

**Total enrolled immunogenicity cohort**

**136 children excluded (Panama)** because of incorrect signed immunogenicity ICF or original ICF lost

**Primary per-protocol immunogenicity cohort, N = 331**

**33 children excluded because:**

- 12 non-compliant with blood sampling schedule
- 16 essential serological data missing
- 1 non-compliant with vaccination schedule
- 4 other protocol violations

**Primary ITT (or vaccinated) immunogenicity cohort, N = 364**

**Control Group, N = 500**

**Total enrolled immunogenicity cohort**

**136 children excluded (Panama)** because of incorrect signed immunogenicity ICF or original ICF lost

**Primary per-protocol immunogenicity cohort, N = 331**

**33 children excluded because:**

- 12 non-compliant with blood sampling schedule
- 16 essential serological data missing
- 1 non-compliant with vaccination schedule
- 4 other protocol violations

**Primary ITT (or vaccinated) immunogenicity cohort, N = 364**

**PHiD-CV Group, N = 501**

**Total enrolled immunogenicity cohort**

**128 children excluded (Panama)** because of incorrect signed immunogenicity informed consent form (ICF) or original ICF lost

**Primary per-protocol immunogenicity cohort,** **N = 334**

**39 children excluded because:**

- 19 non-compliant with blood sampling schedule
- 13 essential serological data missing
- 4 non-compliant with vaccination schedule
- 3 other protocol violations

**Primary ITT (or vaccinated) immunogenicity cohort, N = 373**

**Immunogenicity cohort**

First 500 children enrolled in selected centers in Argentina and first 501 children enrolled in selected centers in Panama

**23,821 children randomized**

**34 children excluded because not administered booster dose**

**115 children excluded because:**

- 77 non-compliant with blood sampling schedule
- 35 violation inclusion/exclusion criteria (wrong age at booster dose)
- 3 other protocol violations

**Booster ITT (or vaccinated) immunogenicity cohort, N = 330**

**Booster per-protocol immunogenicity cohort,** **N = 215**

**28 children excluded because not administered booster dose**

**113 children excluded because:**

- 88 non-compliant with blood sampling schedule
- 23 violation inclusion/exclusion criteria (wrong age at booster dose)
- 2 other protocol violations

**Booster ITT (or vaccinated) immunogenicity cohort, N = 345**

**Booster per-protocol immunogenicity cohort,** **N = 232**

**113 children excluded because:**

- 88 non-compliant with blood sampling schedule
- 23 violation inclusion/exclusion criteria (wrong age at booster dose)
- 2 other protocol violations

**Booster ITT (or vaccinated) immunogenicity cohort, N = 345**

**Booster per-protocol immunogenicity cohort,** **N = 232**
