# Supplementary material for: Efficacy of Pneumococcal Nontypable Haemophilus influenzae Protein D Conjugate Vaccine (PHiD-CV) in Young Latin American Children: A Double-Blind Randomized Controlled Trial
Source: PLoS Med. 2014 Jun 3;11(6):e1001657. doi: 10.1371/journal.pmed.1001657 (PMC4043495; doi:10.1371/journal.pmed.1001657)
Supplement: Figure S2 — Trial profile for children included in the end-of-study analyses of community-acquired pneumonia and invasive pneumococcal disease. (DOCX) [file pmed.1001657.s002.docx]

**Figure S2 Trial profile for children included in the end-of-study analyses of community-acquired pneumonia (CAP) and invasive pneumococcal disease (IPD)**

23,823 randomized

(Argentina: 13,981, Panama: 7,359^a^, Colombia: 2,483)

226 excluded from intent-to-treat cohort for efficacy because:

- 3 not vaccinated (not assigned to a study vaccine group)
- 223 had parents who were minor (77 in Colombia [34 PHiD-CV group, 43 control group], 113 in Panama [57 PHiD-CV group, 55 control group, 1 not assigned to a study group]) or original informed consent form was lost (29 children: 20 PHiD-CV group, 9 control group) or had mental illness (2 children: 1 PHiD-CV group, 1 control group) or infant birth weight was <2,500 g (2 children: 1 PHiD-CV group, 1 control group)

23,597 in intent-to-treat cohort for CAP/IPD efficacy

11,799 received control vaccines in
intent-to-treat cohort for CAP/IPD efficacy

11,798 received PHiD-CV in
intent-to-treat cohort for CAP/IPD efficacy

1,587 excluded from per-protocol cohort for efficacy because:

971 did not complete primary vaccination schedule and/or had no contact beyond 14 days after dose 3

461 non-compliant with primary vaccination schedule

39 received forbidden medication

26 received pneumococcal vaccine other than study vaccine

20 had forbidden underlying medical condition^b^

10 given wrong vial at primary dose

7 wrong age at study entry

52 other inclusion/exclusion criteria not met

1 received vaccine after its expiry date

1,659 excluded from per-protocol cohort for efficacy because:

1,010 did not complete primary vaccination schedule and/or had no contact beyond 14 days after dose 3

460 non-compliant with primary vaccination schedule

37 received forbidden medication

46 received pneumococcal vaccine

24 had forbidden underlying medical condition^b^

12 wrong age at study entry

59 other inclusion/exclusion criteria not met

11 given wrong vial at primary dose

10,140 in per-protocol cohort for CAP/IPD efficacy

10,211 in per-protocol cohort for CAP/IPD efficacy

^a^ During re-monitoring after the interim analysis was performed, two children were detected as enrolled. Both had discontinued the study (consent withdrawn) before receiving vaccination. The children were given subject numbers but as having received no dose, they were not considered for any analysis based on vaccinated children.

^b^ Forbidden underlying medical conditions included, but were not limited to:

- Major congenital defects or serious chronic illness

- Confirmed or suspected immunosuppressive or immunodeficient condition
