# Supplementary material for: Efficacy of Pneumococcal Nontypable Haemophilus influenzae Protein D Conjugate Vaccine (PHiD-CV) in Young Latin American Children: A Double-Blind Randomized Controlled Trial
Source: PLoS Med. 2014 Jun 3;11(6):e1001657. doi: 10.1371/journal.pmed.1001657 (PMC4043495; doi:10.1371/journal.pmed.1001657)
Supplement: Figure S3 — Clinical Otitis Media and Pneumonia Study timeline. (DOCX) [file pmed.1001657.s003.docx]

**Figure S3 Clinical Otitis Media and Pneumonia Study (COMPAS) timeline**
