# Supplementary material for: Efficacy of Pneumococcal Nontypable Haemophilus influenzae Protein D Conjugate Vaccine (PHiD-CV) in Young Latin American Children: A Double-Blind Randomized Controlled Trial
Source: PLoS Med. 2014 Jun 3;11(6):e1001657. doi: 10.1371/journal.pmed.1001657 (PMC4043495; doi:10.1371/journal.pmed.1001657)
Supplement: Table S1 — National public health authorities and ethical review committees. (DOCX) [file pmed.1001657.s004.docx]

**Table S1 National public health authorities and ethical review committees**

| **Argentina** | **Study sites**   - Ethics Committee of Public Health Secretary (Comité de Etica de la Secretaría de Estado de Salud Pública), San Juan - Ethics Institutional Committee of Health Research (Comité Institucional de Etica de Investigación en Salud), Santiago del Estero - Educational and Research Committee of the Hospital de Niños Eva Perón (Comité de Docencia e Investigación Hospital de Niños Eva Perón; name changed from July 2008 to Comité de Docencia, Capacitación e Investigación CePSI Eva Perón), Santiago del Estero - Educational and Research Committee of the Hospital de Niños “Francisco Viano” (Comité de Docencia e Investigación Hospital de Niños "Francisco Viano"), Santiago del Estero - Ethics Committee in Scientific Research (Comité de Etica de Investigación Científica) of the Hospital Dr. Humberto Notti, Mendoza - Independent Ethics Committee in Clinical Research (Comité Independiente de Etica) “Dr. Virgilio G. Foglia”. Name changed from June 2008 to Comité Independiente de Ética en Investigación Clínica “Dr. Carlos Barclay”, Buenos Aires   **National**   - National Administration for Medicines, Food and Medical Technology (ANMAT, Administración Nacional de Medicamentos, Alimentos y Tecnología Médica) - Minister of Justice |
| --- | --- |
| **Colombia** | **Study sites**   - Ethics Committee of the Hospital Carlos Holmes Trujillo (Comité de Ética en Investigación de la Red de Salud del Oriente E.S.E Hospital Carlos Holmes Trujillo), Santiago de Cali - Ethics Committee of Comfenalco (Comité de Ética en Investigación Clínica Comfenalco Valle), Santiago de Cali - Research Ethics Committee of the Medical and Health Science School of El Rosario University (Comité de Ética en Investigación Universidad del Rosario), Bogotá - Biomedical Research Ethics Committee, Pediatric Scientific Corporation (Comité de Ética en Investigación Biomédica, Corporación Científica Pediátrica), Santiago de Cali   **National**   - National Institute for Drug and Food Surveillance (INVIMA, Instituto Nacional de Vigilancia de Medicamentos y Alimentos) |
| **Panama** | **Study sites**   - Committee of Bioethics in Research of the Pediatric Specialties Hospital, Panama City - Committee of Bioethics in Health Research of the Hospital del Niño, Panama City   **National**   - National Committee of Bioethics in Health Research of the Gorgas Memorial Institute for Health Studies |
