# Supplementary material for: Efficacy of Pneumococcal Nontypable Haemophilus influenzae Protein D Conjugate Vaccine (PHiD-CV) in Young Latin American Children: A Double-Blind Randomized Controlled Trial
Source: PLoS Med. 2014 Jun 3;11(6):e1001657. doi: 10.1371/journal.pmed.1001657 (PMC4043495; doi:10.1371/journal.pmed.1001657)
Supplement: Table S2 — Children at high risk of invasive pneumococcal infection. (DOCX) [file pmed.1001657.s005.docx]

**Table S2 Children at high risk of invasive pneumococcal infection**

| **High risk** | |
| --- | --- |
| 1. | Sickle cell disease, congenital or acquired asplenia, or splenic dysfunction |
| 2. | Infection with HIV |
| **Presumed high risk (attack rate not calculated)** | |
| 1. | Congenital immune deficiency: some B- (humoral) or T-lymphocyte deficiencies, complement deficiencies (particularly C1, C2, C3, and C4 deficiencies), or phagocytic disorders (excluding chronic granulomatous disease) |
| 2. | Chronic cardiac disease (particularly cyanotic congenital heart disease and cardiac failure) |
| 3. | Chronic pulmonary disease (including cystic fibrosis, rheumatic pneumonia, tuberculosis, idiopathic interstitial diffuse fibrosis of the lung, pulmonary aspergillosis, and asthma treated with high-dose oral corticosteroid therapy defined as more than 14 days of prednisone or equivalent, ≥0.5 mg/kg/day. Inhaled and topical steroids are allowed) |
| 4. | Cerebrospinal fluid leaks |
| 5. | Chemotherapy |
| 6. | Chronic renal insufficiency, including nephrotic syndrome |
| 7. | Chronic hepatopathy |
| 8. | Diseases associated with immunosuppressive therapy or radiation therapy (including malignant neoplasms, leukemias, lymphomas, and Hodgkin’s disease) and solid organ transplantation |
| 9. | Diabetes mellitus |
| 10. | Multiple myeloma |
| 11. | Preterm infants (born after gestation period ≤32 weeks) and/or a birth weight ≤1,500 g (for Argentina only) |

Adapted from American Academy of Pediatrics Policy Statement [1] and Normas Nacionales de Vacunacion (Argentina) 2008 [2].

**References**

1. American Academy of Pediatrics. (2000) Policy statement: recommendations for the prevention of pneumococcal infections, including the use of pneumococcal conjugate vaccine (Prevnar), pneumococcal polysaccharide vaccine, and antibiotic prophylaxis. Pediatrics 106(2 Pt 1): 362-366.

2. Ministerio de Salud de la Nación del Argentina. (2008) Normas Nacionales de Vacunacion. Available: http://www.msal.gov.ar/images/stories/epidemiologia/inmunizaciones/Normas08.pdf. Accessed 3 June 2013.
