# Supplementary material for: Efficacy of Pneumococcal Nontypable Haemophilus influenzae Protein D Conjugate Vaccine (PHiD-CV) in Young Latin American Children: A Double-Blind Randomized Controlled Trial
Source: PLoS Med. 2014 Jun 3;11(6):e1001657. doi: 10.1371/journal.pmed.1001657 (PMC4043495; doi:10.1371/journal.pmed.1001657)
Supplement: Table S3 — Occurrence of first clinically confirmed acute otitis media episodes (per-protocol cohort for acute otitis media vaccine efficacy analysis). (DOCX) [file pmed.1001657.s006.docx]

**Table S3 Occurrence of first clinically-confirmed acute otitis media (AOM) episodes (per-protocol cohort for AOM vaccine efficacy analysis)**

|  | **PHiD-CV  N = 3010** | | | | **Control  N = 2979** | | | |
| --- | --- | --- | --- | --- | --- | --- | --- | --- |
|  |  | | **95% CI** | |  | | **95% CI** | |
| **Categories** | **n** | **%** | **LL** | **UL** | **n** | **%** | **LL** | **UL** |
| Clinically-confirmed AOM | 204 | 6.78 | 5.91 | 7.73 | 239 | 8.02 | 7.07 | 9.06 |
| Bacteriologically clinically-confirmed AOM | 32 | 1.06 | 0.73 | 1.50 | 45 | 1.51 | 1.10 | 2.02 |
| Pneumococcal clinically-confirmed AOM | 12 | 0.40 | 0.21 | 0.70 | 27 | 0.91 | 0.60 | 1.32 |
| Vaccine serotypes | 6 | 0.20 | 0.07 | 0.43 | 18 | 0.60 | 0.36 | 0.95 |
| 1 | 0 | 0 | 0.00 | 0.12 | 0 | 0 | 0.00 | 0.12 |
| 4 | 0 | 0 | 0.00 | 0.12 | 1 | 0.03 | 0.00 | 0.19 |
| 5 | 0 | 0 | 0.00 | 0.12 | 0 | 0 | 0.00 | 0.12 |
| 6B | 0 | 0 | 0.00 | 0.12 | 4 | 0.13 | 0.04 | 0.34 |
| 7F | 0 | 0 | 0.00 | 0.12 | 0 | 0 | 0.00 | 0.12 |
| 9V | 0 | 0 | 0.00 | 0.12 | 1 | 0.03 | 0.00 | 0.19 |
| 14 | 1 | 0.03 | 0.00 | 0.18 | 3 | 0.10 | 0.02 | 0.29 |
| 18C | 0 | 0 | 0.00 | 0.12 | 0 | 0 | 0.00 | 0.12 |
| 19F | 4 | 0.13 | 0.04 | 0.34 | 8 | 0.27 | 0.12 | 0.53 |
| 23F | 1 | 0.03 | 0.00 | 0.18 | 1 | 0.03 | 0.00 | 0.19 |
| Cross-reactive serotypes | 3 | 0.10 | 0.02 | 0.29 | 4 | 0.13 | 0.04 | 0.34 |
| 6A | 1 | 0.03 | 0.00 | 0.18 | 3 | 0.10 | 0.02 | 0.29 |
| 19A | 2 | 0.07 | 0.01 | 0.24 | 1 | 0.03 | 0.00 | 0.19 |
| Other pneumococcal serotypes | 3 | 0.10 | 0.02 | 0.29 | 4 | 0.13 | 0.04 | 0.34 |
| 3 | 1 | 0.03 | 0.00 | 0.18 | 2 | 0.07 | 0.01 | 0.24 |
| 15A | 1 | 0.03 | 0.00 | 0.18 | 0 | 0 | 0.00 | 0.12 |
| 15C | 1 | 0.03 | 0.00 | 0.18 | 0 | 0 | 0.00 | 0.12 |
| 21 | 0 | 0 | 0.00 | 0.12 | 1 | 0.03 | 0.00 | 0.19 |
| 35B | 0 | 0 | 0.00 | 0.12 | 1 | 0.03 | 0.00 | 0.19 |
| Uncapsulated | 0 | 0 | 0.00 | 0.12 | 1 | 0.03 | 0.00 | 0.19 |
| *H. influenzae* clinically-confirmed AOM | 12 | 0.40 | 0.21 | 0.70 | 14 | 0.47 | 0.26 | 0.79 |
| Nontypable *H. influenzae* | 12 | 0.40 | 0.21 | 0.70 | 14 | 0.47 | 0.26 | 0.79 |
| Other pathogen clinically-confirmed AOM | 8 | 0.27 | 0.11 | 0.52 | 7 | 0.23 | 0.09 | 0.48 |
| *Moraxella catarrhalis* | 1 | 0.03 | 0.00 | 0.18 | 0 | 0 | 0.00 | 0.12 |
| Group A *Streptococcus* | 4 | 0.13 | 0.04 | 0.34 | 4 | 0.13 | 0.04 | 0.34 |
| *Staphylococcus aureus* | 2 | 0.07 | 0.01 | 0.24 | 3 | 0.10 | 0.02 | 0.29 |
| Other bacteria | 1 | 0.03 | 0.00 | 0.18 | 0 | 0 | 0.00 | 0.12 |

N = total number of children

n/% = number/percentage of children reporting a first episode of defined AOM from two weeks after the administration of dose 3

95% CI = exact 95% confidence interval, LL = lower limit, UL = upper limit
