# Supplementary material for: Efficacy of Pneumococcal Nontypable Haemophilus influenzae Protein D Conjugate Vaccine (PHiD-CV) in Young Latin American Children: A Double-Blind Randomized Controlled Trial
Source: PLoS Med. 2014 Jun 3;11(6):e1001657. doi: 10.1371/journal.pmed.1001657 (PMC4043495; doi:10.1371/journal.pmed.1001657)
Supplement: Table S4 — Occurrence of first clinically confirmed acute otitis media episodes (intent-to-treat cohort for acute otitis media vaccine efficacy analysis). (DOCX) [file pmed.1001657.s007.docx]

**Table S4 Occurrence of first clinically-confirmed acute otitis media (AOM) episodes (intent-to-treat cohort for AOM vaccine efficacy analysis)**

|  | **PHiD-CV  N = 3602** | | | | **Control  N = 3612** | | | |
| --- | --- | --- | --- | --- | --- | --- | --- | --- |
|  |  | | **95% CI** | |  | | **95% CI** | |
| **Categories** | **n** | **%** | **LL** | **UL** | **n** | **%** | **LL** | **UL** |
| Clinically-confirmed AOM | 254 | 7.05 | 6.24 | 7.94 | 308 | 8.53 | 7.64 | 9.49 |
| Bacteriologically clinically-confirmed AOM | 45 | 1.25 | 0.91 | 1.67 | 67 | 1.85 | 1.44 | 2.35 |
| Pneumococcal clinically-confirmed AOM | 17 | 0.47 | 0.28 | 0.75 | 38 | 1.05 | 0.75 | 1.44 |
| Vaccine serotypes | 7 | 0.19 | 0.08 | 0.40 | 23 | 0.64 | 0.40 | 0.95 |
| 1 | 0 | 0 | 0.00 | 0.10 | 0 | 0 | 0.00 | 0.10 |
| 4 | 0 | 0 | 0.00 | 0.10 | 1 | 0.03 | 0.00 | 0.15 |
| 5 | 0 | 0 | 0.00 | 0.10 | 0 | 0 | 0.00 | 0.10 |
| 6B | 0 | 0 | 0.00 | 0.10 | 4 | 0.11 | 0.03 | 0.28 |
| 7F | 0 | 0 | 0.00 | 0.10 | 0 | 0 | 0.00 | 0.10 |
| 9V | 0 | 0 | 0.00 | 0.10 | 1 | 0.03 | 0.00 | 0.15 |
| 14 | 1 | 0.03 | 0.00 | 0.15 | 3 | 0.08 | 0.02 | 0.24 |
| 18C | 0 | 0 | 0.00 | 0.10 | 1 | 0.03 | 0.00 | 0.15 |
| 19F | 5 | 0.14 | 0.05 | 0.32 | 11 | 0.30 | 0.15 | 0.54 |
| 23F | 1 | 0.03 | 0.00 | 0.15 | 2 | 0.06 | 0.01 | 0.20 |
| Cross-reactive serotypes | 5 | 0.14 | 0.05 | 0.32 | 7 | 0.19 | 0.08 | 0.40 |
| 6A | 1 | 0.03 | 0.00 | 0.15 | 3 | 0.08 | 0.02 | 0.24 |
| 18B | 0 | 0 | 0.00 | 0.10 | 1 | 0.03 | 0.00 | 0.15 |
| 19A | 4 | 0.11 | 0.03 | 0.28 | 2 | 0.06 | 0.01 | 0.20 |
| 23A | 0 | 0 | 0.00 | 0.10 | 1 | 0.03 | 0.00 | 0.15 |
| Other pneumococcal serotypes | 6 | 0.17 | 0.06 | 0.36 | 7 | 0.19 | 0.08 | 0.40 |
| 3 | 2 | 0.06 | 0.01 | 0.20 | 5 | 0.14 | 0.04 | 0.32 |
| 10F | 1 | 0.03 | 0.00 | 0.15 | 0 | 0 | 0.00 | 0.10 |
| 11A | 1 | 0.03 | 0.00 | 0.15 | 0 | 0 | 0.00 | 0.10 |
| 15A | 1 | 0.03 | 0.00 | 0.15 | 0 | 0 | 0.00 | 0.10 |
| 15C | 1 | 0.03 | 0.00 | 0.15 | 0 | 0 | 0.00 | 0.10 |
| 21 | 0 | 0 | 0.00 | 0.10 | 1 | 0.03 | 0.00 | 0.15 |
| 35B | 0 | 0 | 0.00 | 0.10 | 1 | 0.03 | 0.00 | 0.15 |
| Uncapsulated | 0 | 0 | 0.00 | 0.10 | 1 | 0.03 | 0.00 | 0.15 |
| *H. influenzae* clinically-confirmed AOM | 20 | 0.56 | 0.34 | 0.86 | 24 | 0.66 | 0.43 | 0.99 |
| Any typed *H. influenzae* | 1 | 0.03 | 0.00 | 0.15 | 0 | 0 | 0.00 | 0.10 |
| Type a | 1 | 0.03 | 0.00 | 0.15 | 0 | 0 | 0.00 | 0.10 |
| Nontypable *H. influenzae* | 19 | 0.53 | 0.32 | 0.82 | 24 | 0.66 | 0.43 | 0.99 |
| Other pathogen clinically-confirmed AOM | 10 | 0.28 | 0.13 | 0.51 | 10 | 0.28 | 0.13 | 0.51 |
| *Moraxella catarrhalis* | 2 | 0.06 | 0.01 | 0.20 | 0 | 0 | 0.00 | 0.10 |
| Group A *Streptococcus* | 4 | 0.11 | 0.03 | 0.28 | 4 | 0.11 | 0.03 | 0.28 |
| *Staphylococcus aureus* | 3 | 0.08 | 0.02 | 0.24 | 6 | 0.17 | 0.06 | 0.36 |
| Other bacteria | 1 | 0.03 | 0.00 | 0.15 | 0 | 0 | 0.00 | 0.10 |

N = total number of children

n/% = number/percentage of children reporting a first episode of defined AOM from the administration of dose 1

95% CI = exact 95% confidence interval, LL = lower limit, UL = upper limit
