# Supplementary material for: Efficacy of Pneumococcal Nontypable Haemophilus influenzae Protein D Conjugate Vaccine (PHiD-CV) in Young Latin American Children: A Double-Blind Randomized Controlled Trial
Source: PLoS Med. 2014 Jun 3;11(6):e1001657. doi: 10.1371/journal.pmed.1001657 (PMC4043495; doi:10.1371/journal.pmed.1001657)
Supplement: Table S6 — Occurrence of invasive pneumococcal infection episodes (intent-to-treat cohort for community-acquired pneumonia/invasive pneumococcal infection vaccine efficacy analysis). (DOCX) [file pmed.1001657.s009.docx]

**Table S6 Occurrence of invasive pneumococcal disease (IPD) episodes (intent-to-treat cohort for CAP/IPD vaccine efficacy analysis)**

|  | **PHiD-CV N = 11798** | | | | **Control  N = 11799** | | | |
| --- | --- | --- | --- | --- | --- | --- | --- | --- |
|  |  | | **95% CI** | |  | | **95% CI** | |
| **Categories** | **n** | **%** | **LL** | **UL** | **n** | **%** | **LL** | **UL** |
| IPD | 7 | 0.059 | 0.024 | 0.122 | 21 | 0.178 | 0.110 | 0.272 |
| Vaccine serotypes | 0 | 0 | 0.000 | 0.031 | 18 | 0.153 | 0.090 | 0.241 |
| 1 | 0 | 0 | 0.000 | 0.031 | 0 | 0 | 0.000 | 0.031 |
| 4 | 0 | 0 | 0.000 | 0.031 | 0 | 0 | 0.000 | 0.031 |
| 5 | 0 | 0 | 0.000 | 0.031 | 2 | 0.017 | 0.002 | 0.061 |
| 6B | 0 | 0 | 0.000 | 0.031 | 2 | 0.017 | 0.002 | 0.061 |
| 7F | 0 | 0 | 0.000 | 0.031 | 0 | 0 | 0.000 | 0.031 |
| 9V | 0 | 0 | 0.000 | 0.031 | 0 | 0 | 0.000 | 0.031 |
| 14 | 0 | 0 | 0.000 | 0.031 | 9 | 0.076 | 0.035 | 0.145 |
| 18C | 0 | 0 | 0.000 | 0.031 | 4 | 0.034 | 0.009 | 0.087 |
| 19F | 0 | 0 | 0.000 | 0.031 | 0 | 0 | 0.000 | 0.031 |
| 23F | 0 | 0 | 0.000 | 0.031 | 1 | 0.008 | 0.000 | 0.047 |
| Cross-reactive serotypes | 2 | 0.017 | 0.002 | 0.061 | 1 | 0.008 | 0.000 | 0.047 |
| 19A | 1 | 0.008 | 0.000 | 0.047 | 0 | 0 | 0.000 | 0.031 |
| 6A | 1 | 0.008 | 0.000 | 0.047 | 0 | 0 | 0.000 | 0.031 |
| 9N | 0 | 0 | 0.000 | 0.031 | 1 | 0.008 | 0.000 | 0.047 |
| Other pneumococcal serotypes | 4 | 0.034 | 0.009 | 0.087 | 2 | 0.017 | 0.002 | 0.061 |
| 12F | 1 | 0.008 | 0.000 | 0.047 | 0 | 0 | 0.000 | 0.031 |
| 16F | 0 | 0 | 0.000 | 0.031 | 1 | 0.008 | 0.000 | 0.047 |
| 24F | 1 | 0.008 | 0.000 | 0.047 | 1 | 0.008 | 0.000 | 0.047 |
| 38 | 1 | 0.008 | 0.000 | 0.047 | 0 | 0 | 0.000 | 0.031 |
| 8 | 1 | 0.008 | 0.000 | 0.047 | 0 | 0 | 0.000 | 0.031 |
| Not able to be serotyped | 1 | 0.008 | 0.000 | 0.047 | 0 | 0 | 0.000 | 0.031 |

N = total number of children

n/% = number/percentage of children reporting an IPD episode from the administration of dose 1

95% CI = exact 95% confidence interval, LL = lower limit, UL = upper limit

Note: All IPD cases were first episodes
