# Supplementary material for: Efficacy of Pneumococcal Nontypable Haemophilus influenzae Protein D Conjugate Vaccine (PHiD-CV) in Young Latin American Children: A Double-Blind Randomized Controlled Trial
Source: PLoS Med. 2014 Jun 3;11(6):e1001657. doi: 10.1371/journal.pmed.1001657 (PMC4043495; doi:10.1371/journal.pmed.1001657)
Supplement: Table S7 — Pneumococcal antibody concentration and opsonophagocytic activity in PHiD-CV group (per-protocol immunogenicity cohort for primary or booster vaccination). (DOCX) [file pmed.1001657.s010.docx]

**Table S7 Pneumococcal antibody concentration and opsonophagocytic activity (OPA) in PHiD-CV group (per-protocol immunogenicity cohort for primary or booster vaccination)**

|  |  | **22F-ELISA (95% CI)** | | | **OPA (95% CI)** | | |
| --- | --- | --- | --- | --- | --- | --- | --- |
| **Serotype** | **Timing** | **N** | **% ≥0.2 µg/ml** | **GMC (µg/ml)** | **N** | **% ≥8** | **GMT** |
| 1 | **Post-primary** | 334 | 99.7 (98.3, 100) | 2.51 (2.29, 2.75) | 306 | 91.5 (87.8, 94.4) | 139.5 (116.8, 166.5) |
|  | **Pre-booster** | 231 | 71.0 (64.7, 76.8) | 0.35 (0.31, 0.39) | 221 | 26.2 (20.6, 32.6) | 9.0 (7.4, 10.9) |
|  | **Post-booster** | 219 | 100 (98.3, 100) | 3.58 (3.15, 4.07) | 209 | 93.3 (89.0, 96.3) | 357.5 (281.8, 453.5) |
| 4 | **Post-primary** | 334 | 99.4 (97.9, 99.9) | 3.26 (2.98, 3.56) | 310 | 98.7 (96.7, 99.6) | 771.7 (686.5, 867.6) |
|  | **Pre-booster** | 231 | 82.3 (76.7, 87.0) | 0.48 (0.42, 0.55) | 208 | 51.9 (44.9, 58.9) | 29.2 (21.2, 40.2) |
|  | **Post-booster** | 217 | 100 (98.3, 100) | 6.55 (5.81, 7.38) | 207 | 99.5 (97.3, 100) | 2853.5 (2365.5, 3442.1) |
| 5 | **Post-primary** | 334 | 99.7 (98.3, 100) | 4.20 (3.86, 4.57) | 313 | 96.8 (94.2, 98.5) | 224.8 (196.0, 257.8) |
|  | **Pre-booster** | 231 | 94.4 (90.6, 97.0) | 0.90 (0.80, 1.01) | 205 | 67.3 (60.4, 73.7) | 18.5 (15.4, 22.3) |
|  | **Post-booster** | 219 | 100 (98.3, 100) | 5.73 (5.04, 6.50) | 196 | 99.0 (96.4, 99.9) | 306.1 (252.0, 371.8) |
| 6B | **Post-primary** | 334 | 93.1 (89.8, 95.6) | 1.34 (1.18, 1.52) | 315 | 90.8 (87.0, 93.7) | 689.7 (553.2, 859.7) |
|  | **Pre-booster** | 229 | 79.5 (73.7, 84.5) | 0.55 (0.47, 0.64) | 209 | 77.5 (71.2, 83.0) | 95.5 (70.1, 130.1) |
|  | **Post-booster** | 217 | 99.1 (96.7, 99.9) | 3.32 (2.92, 3.77) | 203 | 98.5 (95.7, 99.7) | 1123.4 (939.2, 1343.9) |
| 7F | **Post-primary** | 334 | 99.7 (98.3, 100) | 3.86 (3.56, 4.19) | 302 | 100 (98.8, 100) | 4656.7 (4175.6, 5193.3) |
|  | **Pre-booster** | 231 | 96.1 (92.7, 98.2) | 0.94 (0.84, 1.04) | 217 | 100 (98.3, 100) | 1522.8 (1338.5, 1732.6) |
|  | **Post-booster** | 218 | 100 (98.3, 100) | 5.77 (5.23, 6.36) | 205 | 99.0 (96.5, 99.9) | 4336.3 (3607.9, 5211.8) |
| 9V | **Post-primary** | 334 | 98.8 (97.0, 99.7) | 3.15 (2.84, 3.50) | 312 | 99.7 (98.2, 100) | 1690.4 (1489.4, 1918.7) |
|  | **Pre-booster** | 230 | 96.1 (92.7, 98.2) | 1.14 (1.00, 1.31) | 213 | 99.5 (97.4, 100) | 709.4 (601.7, 836.3) |
|  | **Post-booster** | 218 | 100 (98.3, 100) | 7.34 (6.51, 8.27) | 200 | 100 (98.2, 100) | 3763.0 (3317.5, 4268.3) |
| 14 | **Post-primary** | 334 | 98.2 (96.1, 99.3) | 4.55 (4.07, 5.10) | 308 | 99.4 (97.7, 99.9) | 908.5 (795.2, 1038.1) |
|  | **Pre-booster** | 231 | 93.9 (90.0, 96.6) | 1.15 (0.98, 1.35) | 205 | 77.1 (70.7, 82.6) | 121.2 (89.7, 163.8) |
|  | **Post-booster** | 219 | 100 (98.3, 100) | 9.31 (8.18, 10.60) | 209 | 99.5 (97.4, 100) | 2659.6 (2266.2, 3121.3) |
| 18C | **Post-primary** | 334 | 98.8 (97.0, 99.7) | 5.32 (4.73, 5.99) | 308 | 94.2 (90.9, 96.5) | 310.9 (259.4, 372.6) |
|  | **Pre-booster** | 231 | 93.9 (90.0, 96.6) | 0.90 (0.79, 1.03) | 204 | 42.2 (35.3, 49.3) | 15.0 (11.5, 19.6) |
|  | **Post-booster** | 219 | 100 (98.3, 100) | 16.38 (14.47, 18.55) | 188 | 99.5 (97.1, 100) | 2426.0 (2041.8, 2882.5) |
| 19F | **Post-primary** | 334 | 97.3 (94.9, 98.8) | 5.33 (4.70, 6.06) | 304 | 91.1 (87.3, 94.1) | 383.0 (308.5, 475.5) |
|  | **Pre-booster** | 231 | 95.7 (92.2, 97.9) | 1.27 (1.09, 1.48) | 218 | 68.8 (62.2, 74.9) | 24.3 (19.6, 30.1) |
|  | **Post-booster** | 219 | 99.5 (97.5, 100) | 9.40 (8.40, 10.52) | 199 | 93.5 (89.1, 96.5) | 657.5 (497.4, 869.1) |
| 23F | **Post-primary** | 334 | 96.1 (93.4, 97.9) | 1.99 (1.76, 2.26) | 317 | 98.1 (95.9, 99.3) | 2167.4 (1863.4, 2521.1) |
|  | **Pre-booster** | 229 | 86.5 (81.3, 90.6) | 0.62 (0.53, 0.72) | 215 | 88.4 (83.3, 92.3) | 679.3 (494.3, 933.6) |
|  | **Post-booster** | 218 | 99.1 (96.7, 99.9) | 4.02 (3.49, 4.62) | 206 | 99.5 (97.3, 100) | 4278.3 (3609.3, 5071.3) |
| Cross-reactive 6A | **Post-primary** | 334 | 64.4 (59.0, 69.5) | 0.32 (0.27, 0.37) | 297 | 78.1 (73.0, 82.7) | 156.9 (121.8, 202.1) |
|  | **Pre-booster** | 231 | 51.5 (44.9, 58.1) | 0.22 (0.18, 0.26) | 189 | 52.4 (45.0, 59.7) | 43.8 (31.0, 62.0) |
|  | **Post-booster** | 219 | 85.4 (80.0, 89.8) | 1.14 (0.93, 1.40) | 185 | 81.1 (74.7, 86.5) | 277.2 (198.7, 386.7) |
| Cross-reactive 19A | **Post-primary** | 334 | 61.1 (55.6, 66.3) | 0.29 (0.25, 0.33) | 302 | 40.1 (34.5, 45.8) | 18.2 (14.5, 22.9) |
|  | **Pre-booster** | 229 | 57.2 (50.5, 63.7) | 0.25 (0.21, 0.30) | 217 | 14.7 (10.3, 20.2) | 6.3 (5.3, 7.4) |
|  | **Post-booster** | 218 | 90.4 (85.7, 93.9) | 1.34 (1.11, 1.62) | 200 | 80.5 (74.3, 85.8) | 132.8 (97.5, 180.8) |

N, number of available children; GMC, geometric mean concentration; GMT, geometric mean titer; Post-primary, 1 month after primary vaccination; Pre-booster, before booster vaccination; Post-booster, 1 month after booster vaccination.
