# Supplementary material for: Efficacy of Pneumococcal Nontypable Haemophilus influenzae Protein D Conjugate Vaccine (PHiD-CV) in Young Latin American Children: A Double-Blind Randomized Controlled Trial
Source: PLoS Med. 2014 Jun 3;11(6):e1001657. doi: 10.1371/journal.pmed.1001657 (PMC4043495; doi:10.1371/journal.pmed.1001657)
Supplement: Table S8 — Serious adverse events reported from study start and administration of the first vaccine dose up to study end (intent-to-treat cohort: all children). (DOCX) [file pmed.1001657.s011.docx]

**Table S8 Serious adverse events (SAEs) reported from study start and administration of the first vaccine dose up to study end (intent-to-treat cohort: all children)***

| **All SAEs** | **PHiD-CV Group N = 11798** | **Control Group N = 11799** |
| --- | --- | --- |
| Children with any SAE(s), n (%) | 2534 (21.5) | 2668 (22.6) |
| Gastroenteritis | 553 (4.7) | 497 (4.2) |
| Pneumonia | 478 (4.1) | 557 (4.7) |
| Bronchiolitis | 473 (4.0) | 518 (4.4) |
| Dehydration | 463 (3.9) | 438 (3.7) |
| Asthmatic crisis | 192 (1.6) | 210 (1.8) |
| Bronchial obstruction | 127 (1.1) | 141 (1.2) |
| Bronchitis | 124 (1.1) | 129 (1.1) |
| Febrile convulsion | 95 (0.8) | 135 (1.1) |
| Bronchopneumonia | 106 (0.9) | 95 (0.8) |
| Urinary tract infection | 76 (0.6) | 93 (0.8) |
| Asthma | 82 (0.7) | 83 (0.7) |
| Head injury | 75 (0.6) | 74 (0.6) |
| Diarrhea | 63 (0.5) | 53 (0.4) |
| Cellulitis | 45 (0.4) | 46 (0.4) |
| Wheezing | 48 (0.4) | 42 (0.4) |
| Gastroenteritis rotavirus | 38 (0.3) | 49 (0.4) |
| Vomiting | 42 (0.4) | 37 (0.3) |
| Craniocerebral injury | 39 (0.3) | 36 (0.3) |
| Thermal burn | 25 (0.2) | 36 (0.3) |
| Multiple injuries | 26 (0.2) | 33 (0.3) |
| Ileus paralytic | 33 (0.3) | 23 (0.2) |
| Pyrexia | 24 (0.2) | 31 (0.3) |
| Convulsion | 28 (0.2) | 25 (0.2) |
| Chemical poisoning | 24 (0.2) | 21 (0.2) |
| Enteritis | 15 (0.1) | 20 (0.2) |
| Pertussis | 19 (0.2) | 16 (0.1) |
| Dengue fever | 18 (0.2) | 15 (0.1) |
| Otitis media acute | 16 (0.1) | 16 (0.1) |
| Foreign body | 16 (0.1) | 15 (0.1) |
| Lymphadenitis | 12 (0.1) | 19 (0.2) |
| Bronchial hyperreactivity | 16 (0.1) | 14 (0.1) |
| Periorbital cellulitis | 12 (0.1) | 16 (0.1) |
| Varicella | 10 (0.1) | 18 (0.2) |
| Abscess limb | 10 (0.1) | 16 (0.1) |
| Atypical pneumonia | 14 (0.1) | 12 (0.1) |
| Upper respiratory tract infection | 15 (0.1) | 11 (0.1) |
| Intussusception | 14 (0.1) | 11 (0.1) |
| Sepsis | 11 (0.1) | 14 (0.1) |
| Urticaria | 10 (0.1) | 15 (0.1) |
| Toxicity to various agents | 12 (0.1) | 12 (0.1) |
| Laryngitis | 9 (0.1) | 14 (0.1) |
| Viral infection | 12 (0.1) | 11 (0.1) |
| Subcutaneous abscess | 8 (0.1) | 14 (0.1) |
| Constipation | 11 (0.1) | 10 (0.1) |
| Abscess | 11 (0.1) | 9 (0.1) |
| Accidental exposure | 10 (0.1) | 8 (0.1) |
| Gastroenteritis viral | 9 (0.1) | 9 (0.1) |
| Pharyngitis | 5 (0.0) | 13 (0.1) |
| Croup infectious | 10 (0.1) | 7 (0.1) |
| Gastroenteritis bacterial | 8 (0.1) | 9 (0.1) |
| Pneumonia viral | 8 (0.1) | 9 (0.1) |
| Pyelonephritis | 11 (0.1) | 6 (0.1) |
| Gastroenteritis shigella | 8 (0.1) | 8 (0.1) |
| Viral pharyngitis | 9 (0.1) | 7 (0.1) |
| Atelectasis | 10 (0.1) | 5 (0.0) |
| Bronchospasm | 6 (0.1) | 9 (0.1) |
| Tracheitis | 8 (0.1) | 7 (0.1) |
| Anemia | 5 (0.0) | 9 (0.1) |
| Burns second degree | 6 (0.1) | 8 (0.1) |
| Impetigo | 10 (0.1) | 4 (0.0) |
| Sinusitis | 9 (0.1) | 5 (0.0) |
| Abdominal pain | 8 (0.1) | 5 (0.0) |
| Electrolyte imbalance | 8 (0.1) | 5 (0.0) |
| Oral herpes | 11 (0.1) | 2 (0.0) |
| Abscess neck | 4 (0.0) | 8 (0.1) |
| Kawasaki’s disease | 9 (0.1) | 3 (0.0) |
| Otitis media | 4 (0.0) | 8 (0.1) |
| Pyoderma | 4 (0.0) | 8 (0.1) |
| Rhinitis | 5 (0.0) | 7 (0.1) |
| Arthropod sting | 6 (0.1) | 5 (0.0) |
| Hypersensitivity | 5 (0.0) | 6 (0.1) |
| Pleural effusion | 8 (0.1) | 3 (0.0) |
| Respiratory syncytial virus bronchiolitis | 6 (0.1) | 5 (0.0) |
| Septic shock | 4 (0.0) | 7 (0.1) |
| Skull fracture | 6 (0.1) | 5 (0.0) |
| Bacteremia | 4 (0.0) | 6 (0.1) |
| Interstitial lung disease | 6 (0.1) | 4 (0.0) |
| Respiratory distress | 4 (0.0) | 6 (0.1) |
| Gastroesophageal reflux disease | 6 (0.1) | 3 (0.0) |
| Hypokalemia | 4 (0.0) | 5 (0.0) |
| Infectious mononucleosis | 5 (0.0) | 4 (0.0) |
| Nasopharyngitis | 6 (0.1) | 3 (0.0) |
| Near drowning | 6 (0.1) | 3 (0.0) |
| Respiratory syncytial virus bronchitis | 3 (0.0) | 6 (0.1) |
| Stomatitis | 3 (0.0) | 6 (0.1) |
| Breath holding | 2 (0.0) | 6 (0.1) |
| Exanthema subitum | 4 (0.0) | 4 (0.0) |
| Febrile neutropenia | 4 (0.0) | 4 (0.0) |
| Henoch-Schonlein purpura | 5 (0.0) | 3 (0.0) |
| Inguinal hernia, obstructive | 5 (0.0) | 3 (0.0) |
| Meningitis aseptic | 6 (0.1) | 2 (0.0) |
| Peritonitis | 4 (0.0) | 4 (0.0) |
| Animal bite | 5 (0.0) | 2 (0.0) |
| Apparent life threatening event | 4 (0.0) | 3 (0.0) |
| Femur fracture | 2 (0.0) | 5 (0.0) |
| Hematuria | 3 (0.0) | 4 (0.0) |
| Ileus | 4 (0.0) | 3 (0.0) |
| Malnutrition | 3 (0.0) | 4 (0.0) |
| Pneumonia respiratory syncytial viral | 2 (0.0) | 5 (0.0) |
| Pneumonitis | 3 (0.0) | 4 (0.0) |
| Tonsillitis | 4 (0.0) | 3 (0.0) |
| Upper limb fracture | 3 (0.0) | 4 (0.0) |
| Viral rash | 3 (0.0) | 4 (0.0) |
| Accidental poisoning | 3 (0.0) | 3 (0.0) |
| Appendicitis | 3 (0.0) | 3 (0.0) |
| Escherichia urinary tract infection | 4 (0.0) | 2 (0.0) |
| Food poisoning | 5 (0.0) | 1 (0.0) |
| Humerus fracture | 4 (0.0) | 2 (0.0) |
| Hypovolemic shock | 3 (0.0) | 3 (0.0) |
| Pharyngotonsillitis | 4 (0.0) | 2 (0.0) |
| Pneumonia aspiration | 3 (0.0) | 3 (0.0) |
| Shock | 2 (0.0) | 4 (0.0) |
| Acute sinusitis | 2 (0.0) | 3 (0.0) |
| Adenoiditis | 1 (0.0) | 4 (0.0) |
| Angioedema | 2 (0.0) | 3 (0.0) |
| Arthritis bacterial | 3 (0.0) | 2 (0.0) |
| Asphyxia | 2 (0.0) | 3 (0.0) |
| Aspiration bronchial | 2 (0.0) | 3 (0.0) |
| Electric shock | 4 (0.0) | 1 (0.0) |
| Fecaloma | 2 (0.0) | 3 (0.0) |
| Gastritis | 1 (0.0) | 4 (0.0) |
| Hand fracture | 2 (0.0) | 3 (0.0) |
| Idiopathic thrombocytopenic purpura | 3 (0.0) | 2 (0.0) |
| Myiasis | 1 (0.0) | 4 (0.0) |
| Nephrotic syndrome | 2 (0.0) | 3 (0.0) |
| Poisoning | 3 (0.0) | 2 (0.0) |
| Synovitis | 3 (0.0) | 2 (0.0) |
| Wound | 0 (0.0) | 5 (0.0) |
| Apnea | 3 (0.0) | 1 (0.0) |
| Ataxia | 2 (0.0) | 2 (0.0) |
| Bacterial diarrhea | 2 (0.0) | 2 (0.0) |
| Electrocution | 2 (0.0) | 2 (0.0) |
| Empyema | 2 (0.0) | 2 (0.0) |
| Epilepsy | 2 (0.0) | 2 (0.0) |
| Gastroenteritis adenovirus | 1 (0.0) | 3 (0.0) |
| Giardiasis | 1 (0.0) | 3 (0.0) |
| Herbal toxicity | 2 (0.0) | 2 (0.0) |
| Intestinal obstruction | 2 (0.0) | 2 (0.0) |
| Lymphadenopathy | 2 (0.0) | 2 (0.0) |
| Nosocomial infection | 1 (0.0) | 3 (0.0) |
| Pneumonitis chemical | 1 (0.0) | 3 (0.0) |
| Pneumothorax | 2 (0.0) | 2 (0.0) |
| Acute lymphocytic leukemia | 1 (0.0) | 2 (0.0) |
| Amoebic dysentery | 0 (0.0) | 3 (0.0) |
| Arthropod bite | 2 (0.0) | 1 (0.0) |
| Burn infection | 2 (0.0) | 1 (0.0) |
| Carbon monoxide poisoning | 2 (0.0) | 1 (0.0) |
| Cystic fibrosis | 3 (0.0) | 0 (0.0) |
| Dermatitis allergic | 2 (0.0) | 1 (0.0) |
| Ear infection | 1 (0.0) | 2 (0.0) |
| Food intolerance | 0 (0.0) | 3 (0.0) |
| Hemolytic uremic syndrome | 2 (0.0) | 1 (0.0) |
| Hypoglycemia | 3 (0.0) | 0 (0.0) |
| Influenza | 1 (0.0) | 2 (0.0) |
| Inguinal hernia | 0 (0.0) | 3 (0.0) |
| Meningitis | 1 (0.0) | 2 (0.0) |
| Mouth injury | 1 (0.0) | 2 (0.0) |
| Physical abuse | 1 (0.0) | 2 (0.0) |
| Pneumococcal sepsis | 1 (0.0) | 2 (0.0) |
| Postoperative wound infection | 2 (0.0) | 1 (0.0) |
| Pyelonephritis acute | 2 (0.0) | 1 (0.0) |
| Staphylococcal bacteremia | 2 (0.0) | 1 (0.0) |
| Staphylococcal scalded skin syndrome | 3 (0.0) | 0 (0.0) |
| Systemic candida | 2 (0.0) | 1 (0.0) |
| Thrombocytopenic purpura | 1 (0.0) | 2 (0.0) |
| Tibia fracture | 1 (0.0) | 2 (0.0) |
| Upper gastrointestinal hemorrhage | 2 (0.0) | 1 (0.0) |
| Wound infection | 0 (0.0) | 3 (0.0) |
| Acute abdomen | 2 (0.0) | 0 (0.0) |
| Acute myeloid leukemia | 1 (0.0) | 1 (0.0) |
| Acute pulmonary edema | 1 (0.0) | 1 (0.0) |
| Acute respiratory failure | 0 (0.0) | 2 (0.0) |
| Allergic bronchitis | 1 (0.0) | 1 (0.0) |
| Amoebiasis | 0 (0.0) | 2 (0.0) |
| Anal abscess | 2 (0.0) | 0 (0.0) |
| Appendix disorder | 2 (0.0) | 0 (0.0) |
| Bacterial infection | 0 (0.0) | 2 (0.0) |
| Cardiac failure | 1 (0.0) | 1 (0.0) |
| Cardio-respiratory arrest | 2 (0.0) | 0 (0.0) |
| Cellulitis of male external genital organ | 1 (0.0) | 1 (0.0) |
| Cellulitis orbital | 1 (0.0) | 1 (0.0) |
| Cephalhematoma | 1 (0.0) | 1 (0.0) |
| Chest injury | 1 (0.0) | 1 (0.0) |
| Conjunctivitis | 1 (0.0) | 1 (0.0) |
| Cow’s milk intolerance | 1 (0.0) | 1 (0.0) |
| Crying | 1 (0.0) | 1 (0.0) |
| Cyanosis | 1 (0.0) | 1 (0.0) |
| Cytomegalovirus hepatitis | 1 (0.0) | 1 (0.0) |
| Dactylitis | 0 (0.0) | 2 (0.0) |
| Diarrhea hemorrhagic | 1 (0.0) | 1 (0.0) |
| Diarrhea infectious | 2 (0.0) | 0 (0.0) |
| Drug hypersensitivity | 2 (0.0) | 0 (0.0) |
| Dyspnea | 1 (0.0) | 1 (0.0) |
| Electrical burn | 1 (0.0) | 1 (0.0) |
| Encephalitis viral | 1 (0.0) | 1 (0.0) |
| Epistaxis | 1 (0.0) | 1 (0.0) |
| Erythema multiforme | 0 (0.0) | 2 (0.0) |
| External ear cellulitis | 2 (0.0) | 0 (0.0) |
| Extrapyramidal disorder | 2 (0.0) | 0 (0.0) |
| Fracture | 1 (0.0) | 1 (0.0) |
| Gastrointestinal hemorrhage | 0 (0.0) | 2 (0.0) |
| Glomerulonephritis acute | 2 (0.0) | 0 (0.0) |
| Hematoma | 0 (0.0) | 2 (0.0) |
| Hemolysis | 1 (0.0) | 1 (0.0) |
| Hemolytic anemia | 1 (0.0) | 1 (0.0) |
| Hepatic failure | 1 (0.0) | 1 (0.0) |
| Herpangina | 2 (0.0) | 0 (0.0) |
| Hypothermia | 1 (0.0) | 1 (0.0) |
| Infantile asthma | 1 (0.0) | 1 (0.0) |
| Infected cyst | 2 (0.0) | 0 (0.0) |
| Laceration | 2 (0.0) | 0 (0.0) |
| Limb crushing injury | 0 (0.0) | 2 (0.0) |
| Lymph node abscess | 2 (0.0) | 0 (0.0) |
| Mallory-Weiss syndrome | 1 (0.0) | 1 (0.0) |
| Mastoiditis | 2 (0.0) | 0 (0.0) |
| Meningitis meningococcal | 1 (0.0) | 1 (0.0) |
| Meningitis pneumococcal | 0 (0.0) | 2 (0.0) |
| Meningitis viral | 1 (0.0) | 1 (0.0) |
| Neuroblastoma | 0 (0.0) | 2 (0.0) |
| Open fracture | 0 (0.0) | 2 (0.0) |
| Phlebitis | 1 (0.0) | 1 (0.0) |
| Pneumonia influenza | 1 (0.0) | 1 (0.0) |
| Pyloric stenosis | 1 (0.0) | 1 (0.0) |
| Rash | 2 (0.0) | 0 (0.0) |
| Respiratory syncytial virus infection | 0 (0.0) | 2 (0.0) |
| Road traffic accident | 0 (0.0) | 2 (0.0) |
| Shigella infection | 1 (0.0) | 1 (0.0) |
| Sickle cell anemia | 1 (0.0) | 1 (0.0) |
| Sickle cell anemia with crisis | 0 (0.0) | 2 (0.0) |
| Sleep apnea syndrome | 1 (0.0) | 1 (0.0) |
| Staphylococcal sepsis | 1 (0.0) | 1 (0.0) |
| Streptococcal sepsis | 2 (0.0) | 0 (0.0) |
| Syncope | 1 (0.0) | 1 (0.0) |
| Tooth abscess | 2 (0.0) | 0 (0.0) |
| Typhoid fever | 1 (0.0) | 1 (0.0) |
| Abdominal adhesions | 0 (0.0) | 1 (0.0) |
| Abdominal distension | 1 (0.0) | 0 (0.0) |
| Abdominal injury | 0 (0.0) | 1 (0.0) |
| Abdominal mass | 1 (0.0) | 0 (0.0) |
| Abdominal wall abscess | 0 (0.0) | 1 (0.0) |
| Abscess of eyelid | 1 (0.0) | 0 (0.0) |
| Acarodermatitis | 0 (0.0) | 1 (0.0) |
| Accidental overdose | 1 (0.0) | 0 (0.0) |
| Acid base balance abnormal | 1 (0.0) | 0 (0.0) |
| Acquired immunodeficiency syndrome | 1 (0.0) | 0 (0.0) |
| Acute hemorrhagic edema of infancy | 0 (0.0) | 1 (0.0) |
| Acute lung injury | 1 (0.0) | 0 (0.0) |
| Adenovirus infection | 0 (0.0) | 1 (0.0) |
| Agitation | 0 (0.0) | 1 (0.0) |
| Alcohol use | 0 (0.0) | 1 (0.0) |
| Apneic attack | 0 (0.0) | 1 (0.0) |
| Appendicitis perforated | 1 (0.0) | 0 (0.0) |
| Arthritis | 0 (0.0) | 1 (0.0) |
| Ascariasis | 0 (0.0) | 1 (0.0) |
| Aspiration | 0 (0.0) | 1 (0.0) |
| Back injury | 1 (0.0) | 0 (0.0) |
| Balanoposthitis | 1 (0.0) | 0 (0.0) |
| Binge eating | 1 (0.0) | 0 (0.0) |
| Brain injury | 1 (0.0) | 0 (0.0) |
| Brain neoplasm | 1 (0.0) | 0 (0.0) |
| Brain edema | 0 (0.0) | 1 (0.0) |
| Breast abscess | 0 (0.0) | 1 (0.0) |
| Bullous impetigo | 1 (0.0) | 0 (0.0) |
| Burns first degree | 0 (0.0) | 1 (0.0) |
| CNS ventriculitis | 1 (0.0) | 0 (0.0) |
| Capillary fragility | 1 (0.0) | 0 (0.0) |
| Cardiac failure congestive | 1 (0.0) | 0 (0.0) |
| Cat scratch disease | 0 (0.0) | 1 (0.0) |
| Cerebral palsy | 0 (0.0) | 1 (0.0) |
| Cerebrospinal fistula | 1 (0.0) | 0 (0.0) |
| Chemical injury | 1 (0.0) | 0 (0.0) |
| Cholangitis | 1 (0.0) | 0 (0.0) |
| Cholecystitis | 0 (0.0) | 1 (0.0) |
| Coagulopathy | 1 (0.0) | 0 (0.0) |
| Coarctation of the aorta | 0 (0.0) | 1 (0.0) |
| Colostomy infection | 1 (0.0) | 0 (0.0) |
| Compartment syndrome | 1 (0.0) | 0 (0.0) |
| Congenital absence of bile ducts | 1 (0.0) | 0 (0.0) |
| Congenital oral malformation | 0 (0.0) | 1 (0.0) |
| Congenital syphilis | 1 (0.0) | 0 (0.0) |
| Conjunctivitis allergic | 0 (0.0) | 1 (0.0) |
| Conjunctivitis bacterial | 0 (0.0) | 1 (0.0) |
| Conjunctivitis chlamydial | 0 (0.0) | 1 (0.0) |
| Conjunctivitis infective | 1 (0.0) | 0 (0.0) |
| Connective tissue disorder | 0 (0.0) | 1 (0.0) |
| Contusion | 1 (0.0) | 0 (0.0) |
| Cutaneous loxoscelism | 1 (0.0) | 0 (0.0) |
| Dermatitis bullous | 1 (0.0) | 0 (0.0) |
| Dermatitis contact | 1 (0.0) | 0 (0.0) |
| Dermatitis diaper | 1 (0.0) | 0 (0.0) |
| Dermatitis exfoliative | 1 (0.0) | 0 (0.0) |
| Dermatosis | 1 (0.0) | 0 (0.0) |
| Dermoid cyst | 1 (0.0) | 0 (0.0) |
| Diabetes mellitus | 1 (0.0) | 0 (0.0) |
| Diabetic ketoacidosis | 1 (0.0) | 0 (0.0) |
| Disseminated intravascular coagulation | 1 (0.0) | 0 (0.0) |
| Dysentery | 0 (0.0) | 1 (0.0) |
| Dyspepsia | 1 (0.0) | 0 (0.0) |
| Dystonia | 0 (0.0) | 1 (0.0) |
| Effusion | 1 (0.0) | 0 (0.0) |
| Encephalopathy | 0 (0.0) | 1 (0.0) |
| Enteritis infectious | 1 (0.0) | 0 (0.0) |
| Enterococcal sepsis | 1 (0.0) | 0 (0.0) |
| Erysipelas | 1 (0.0) | 0 (0.0) |
| Extradural hematoma | 0 (0.0) | 1 (0.0) |
| Extremity necrosis | 0 (0.0) | 1 (0.0) |
| Eye burns | 1 (0.0) | 0 (0.0) |
| Eye injury | 1 (0.0) | 0 (0.0) |
| Eyelid injury | 0 (0.0) | 1 (0.0) |
| Face injury | 0 (0.0) | 1 (0.0) |
| Failure to thrive | 1 (0.0) | 0 (0.0) |
| Feeding disorder neonatal | 0 (0.0) | 1 (0.0) |
| Finger amputation | 1 (0.0) | 0 (0.0) |
| Flushing | 0 (0.0) | 1 (0.0) |
| Food allergy | 0 (0.0) | 1 (0.0) |
| Fungal skin infection | 1 (0.0) | 0 (0.0) |
| Gastritis erosive | 0 (0.0) | 1 (0.0) |
| Gastrointestinal disorder | 0 (0.0) | 1 (0.0) |
| Genital lesion | 1 (0.0) | 0 (0.0) |
| Gingival bleeding | 1 (0.0) | 0 (0.0) |
| Gingival injury | 0 (0.0) | 1 (0.0) |
| Gingivitis | 0 (0.0) | 1 (0.0) |
| Grand mal convulsion | 0 (0.0) | 1 (0.0) |
| Guillain-Barre syndrome | 0 (0.0) | 1 (0.0) |
| Hematochezia | 1 (0.0) | 0 (0.0) |
| Hemorrhagic disorder | 1 (0.0) | 0 (0.0) |
| Hemorrhagic urticaria | 1 (0.0) | 0 (0.0) |
| Hantaviral infection | 1 (0.0) | 0 (0.0) |
| Heart disease congenital | 0 (0.0) | 1 (0.0) |
| Heat rash | 0 (0.0) | 1 (0.0) |
| Hepatitis acute | 0 (0.0) | 1 (0.0) |
| Hepatoblastoma | 1 (0.0) | 0 (0.0) |
| Herpes simplex | 0 (0.0) | 1 (0.0) |
| Herpes virus infection | 0 (0.0) | 1 (0.0) |
| Histiocytosis hematophagic | 0 (0.0) | 1 (0.0) |
| Hydronephrosis | 0 (0.0) | 1 (0.0) |
| Hyperadrenalism | 1 (0.0) | 0 (0.0) |
| Hypercalcemia | 1 (0.0) | 0 (0.0) |
| Hyperglycemia | 0 (0.0) | 1 (0.0) |
| Hypersplenism | 1 (0.0) | 0 (0.0) |
| Hypertension | 1 (0.0) | 0 (0.0) |
| Hypochromic anemia | 1 (0.0) | 0 (0.0) |
| Hypothyroidism | 1 (0.0) | 0 (0.0) |
| Hypoxic-ischemic encephalopathy | 1 (0.0) | 0 (0.0) |
| Immunodeficiency | 0 (0.0) | 1 (0.0) |
| Infantile colic | 0 (0.0) | 1 (0.0) |
| Infantile spasms | 0 (0.0) | 1 (0.0) |
| Infected bites | 0 (0.0) | 1 (0.0) |
| Infected fistula | 0 (0.0) | 1 (0.0) |
| Infective pulmonary exacerbation of cystic fibrosis | 1 (0.0) | 0 (0.0) |
| Injury | 0 (0.0) | 1 (0.0) |
| Intestinal hemorrhage | 1 (0.0) | 0 (0.0) |
| Intestinal perforation | 1 (0.0) | 0 (0.0) |
| Intracardiac mass | 1 (0.0) | 0 (0.0) |
| Intracranial pressure increased | 0 (0.0) | 1 (0.0) |
| Iron deficiency anemia | 1 (0.0) | 0 (0.0) |
| Klebsiella bacteremia | 1 (0.0) | 0 (0.0) |
| Klebsiella sepsis | 1 (0.0) | 0 (0.0) |
| Lactose intolerance | 0 (0.0) | 1 (0.0) |
| Laryngeal edema | 0 (0.0) | 1 (0.0) |
| Laryngomalacia | 1 (0.0) | 0 (0.0) |
| Leukaemoid reaction | 0 (0.0) | 1 (0.0) |
| Limb traumatic amputation | 0 (0.0) | 1 (0.0) |
| Lobar pneumonia | 1 (0.0) | 0 (0.0) |
| Lower limb fracture | 0 (0.0) | 1 (0.0) |
| Lung disorder | 1 (0.0) | 0 (0.0) |
| Lung infection | 1 (0.0) | 0 (0.0) |
| Malabsorption | 0 (0.0) | 1 (0.0) |
| Meningomyelocele | 1 (0.0) | 0 (0.0) |
| Metabolic acidosis | 1 (0.0) | 0 (0.0) |
| Milk allergy | 0 (0.0) | 1 (0.0) |
| Multi-organ failure | 1 (0.0) | 0 (0.0) |
| Muscular weakness | 0 (0.0) | 1 (0.0) |
| Myoclonic epilepsy | 0 (0.0) | 1 (0.0) |
| Nasal abscess | 0 (0.0) | 1 (0.0) |
| Necrotising fasciitis | 0 (0.0) | 1 (0.0) |
| Nephroblastoma | 0 (0.0) | 1 (0.0) |
| Neutropenia | 0 (0.0) | 1 (0.0) |
| Obstructive airways disorder | 0 (0.0) | 1 (0.0) |
| Oliguria | 1 (0.0) | 0 (0.0) |
| Open wound | 1 (0.0) | 0 (0.0) |
| Oral candidiasis | 1 (0.0) | 0 (0.0) |
| Osteomyelitis | 1 (0.0) | 0 (0.0) |
| Otitis externa | 1 (0.0) | 0 (0.0) |
| Pancytopenia | 1 (0.0) | 0 (0.0) |
| Paronychia | 0 (0.0) | 1 (0.0) |
| Parotitis | 1 (0.0) | 0 (0.0) |
| Periostitis | 0 (0.0) | 1 (0.0) |
| Pharyngeal abscess | 1 (0.0) | 0 (0.0) |
| Pneumocystis jiroveci infection | 1 (0.0) | 0 (0.0) |
| Pneumonia adenoviral | 0 (0.0) | 1 (0.0) |
| Pneumonia bacterial | 1 (0.0) | 0 (0.0) |
| Pneumonia pneumococcal | 0 (0.0) | 1 (0.0) |
| Porencephaly | 0 (0.0) | 1 (0.0) |
| Post-traumatic epilepsy | 1 (0.0) | 0 (0.0) |
| Postoperative fever | 0 (0.0) | 1 (0.0) |
| Prerenal failure | 1 (0.0) | 0 (0.0) |
| Presyncope | 0 (0.0) | 1 (0.0) |
| Procedural hemorrhage | 0 (0.0) | 1 (0.0) |
| Pseudomembranous colitis | 1 (0.0) | 0 (0.0) |
| Psychomotor retardation | 0 (0.0) | 1 (0.0) |
| Pulmonary edema | 0 (0.0) | 1 (0.0) |
| Pulmonary sepsis | 1 (0.0) | 0 (0.0) |
| Pyelocaliectasis | 1 (0.0) | 0 (0.0) |
| Pyomyositis | 1 (0.0) | 0 (0.0) |
| Rash scarlatiniform | 0 (0.0) | 1 (0.0) |
| Rectal hemorrhage | 0 (0.0) | 1 (0.0) |
| Renal failure acute | 0 (0.0) | 1 (0.0) |
| Respiratory disorder | 1 (0.0) | 0 (0.0) |
| Respiratory tract infection | 0 (0.0) | 1 (0.0) |
| Respiratory tract infection viral | 0 (0.0) | 1 (0.0) |
| Retinoblastoma | 1 (0.0) | 0 (0.0) |
| Retinoblastoma bilateral | 0 (0.0) | 1 (0.0) |
| Roseola | 1 (0.0) | 0 (0.0) |
| Sacroiliitis | 0 (0.0) | 1 (0.0) |
| Salmonellosis | 1 (0.0) | 0 (0.0) |
| Scarlet fever | 0 (0.0) | 1 (0.0) |
| Selective IgA immunodeficiency | 1 (0.0) | 0 (0.0) |
| Sepsis syndrome | 1 (0.0) | 0 (0.0) |
| Skin candida | 0 (0.0) | 1 (0.0) |
| Skin infection | 0 (0.0) | 1 (0.0) |
| Skin edema | 1 (0.0) | 0 (0.0) |
| Spinal muscular atrophy | 1 (0.0) | 0 (0.0) |
| Staphylococcal skin infection | 1 (0.0) | 0 (0.0) |
| Status epilepticus | 1 (0.0) | 0 (0.0) |
| Subdural hematoma | 0 (0.0) | 1 (0.0) |
| Sudden death | 0 (0.0) | 1 (0.0) |
| Sudden infant death syndrome | 0 (0.0) | 1 (0.0) |
| Systemic inflammatory response syndrome | 1 (0.0) | 0 (0.0) |
| Tendon injury | 0 (0.0) | 1 (0.0) |
| Tenosynovitis | 0 (0.0) | 1 (0.0) |
| Thalassemia sickle cell | 0 (0.0) | 1 (0.0) |
| Therapeutic hypothermia | 0 (0.0) | 1 (0.0) |
| Thrombocytopenia | 1 (0.0) | 0 (0.0) |
| Thyroglossal cyst infection | 1 (0.0) | 0 (0.0) |
| Toe amputation | 0 (0.0) | 1 (0.0) |
| Tongue injury | 1 (0.0) | 0 (0.0) |
| Tooth fracture | 1 (0.0) | 0 (0.0) |
| Torticollis | 1 (0.0) | 0 (0.0) |
| Traumatic hematoma | 1 (0.0) | 0 (0.0) |
| Trigger finger | 1 (0.0) | 0 (0.0) |
| Type 1 diabetes mellitus | 0 (0.0) | 1 (0.0) |
| Ulcerative keratitis | 1 (0.0) | 0 (0.0) |
| Urosepsis | 1 (0.0) | 0 (0.0) |
| VIIth nerve paralysis | 0 (0.0) | 1 (0.0) |
| Ventricular arrhythmia | 1 (0.0) | 0 (0.0) |
| Viral sepsis | 0 (0.0) | 1 (0.0) |
| Viral tonsillitis | 0 (0.0) | 1 (0.0) |
| Vulvovaginal injury | 1 (0.0) | 0 (0.0) |
| Vulvovaginitis | 0 (0.0) | 1 (0.0) |
| Wound dehiscence | 1 (0.0) | 0 (0.0) |

*Further safety information is provided in the clinical study register for COMPAS (<http://www.gsk-clinicalstudyregister.com/files2/771cae3d-32dd-4441-bb00-78a7812eb2df>)
