# Supplementary material for: Efficacy of Pneumococcal Nontypable Haemophilus influenzae Protein D Conjugate Vaccine (PHiD-CV) in Young Latin American Children: A Double-Blind Randomized Controlled Trial
Source: PLoS Med. 2014 Jun 3;11(6):e1001657. doi: 10.1371/journal.pmed.1001657 (PMC4043495; doi:10.1371/journal.pmed.1001657)
Supplement: Text S1 — Ethical considerations and informed consent. (DOCX) [file pmed.1001657.s012.docx]

**Text S1 Ethical considerations and informed consent**

The trial was designed in accordance with ICH Good Clinical Practice (GCP) guidelines and the 1996 Declaration of Helsinki. During the course of the study, when issues relating to compliance with GCP guidelines or the protocol were identified, they were investigated and corrective actions were implemented consistent with GCP guidelines and in order to achieve compliance with the protocol.

An Independent Data Monitoring Committee (IDMC) supervised study progress and safety of the children by reviewing serious adverse events, assessing potential treatment harm, and making recommendations to GlaxoSmithKline Vaccines regarding safety measures, study design, and reporting or analysis plans. The committee included seven external experts in infectious diseases and/or statistics from Latin America, the USA, Europe, and Africa, and met at least every 6 months, dependent on study stage.

Parents and legally authorized representatives were made aware of the study by different means, including distribution of brochures in health centers and informing pregnant women at maternity centers or parents at vaccination clinics. Recruitment, informed consent, and vaccination procedures typically occurred in participating primary healthcare centers and routine vaccination sites. All informed consent material was approved by local Institutional Review Boards (IRBs) and met all applicable regulatory requirements. The study protocol was approved by national public health authorities and ethical review committees for each study site (listed in Table S1). The trial’s purpose, procedures, and parental responsibilities were explained in detail to each parent. Written informed consent was obtained from children’s parents/guardians prior to the performance of any study-specific procedures in accordance with GCP, all applicable regulatory requirements, and the Declaration of Helsinki, except for the deviations described below. Each child was provided with a study identification card or personalized sticker for insertion in a vaccination card and a telephone number to contact study personnel.

In Colombia and Panama, issues involving the informed consent process were identified. In Colombia, written informed consent was obtained from the parents of 285 children who should not have been enrolled according to Colombian law. For 283 children, informed consent was obtained from parents who were minor (both under 18 years of age). These children received at least one study vaccine dose. Parents who had become 18 years of age during the study were given the opportunity to re-consent if they were willing to let their child continue to participate in the study. For 77 children, however, both parents were still minor. Of these, 76 were withdrawn from the study and blinding was broken. In addition, one child had been withdrawn (consent withdrawal) before unblinding could be done. Two children with parents with mental illness were also enrolled in Colombia and received at least one vaccine dose. These children were withdrawn from the study and blinding was broken. Children who were part of the control group were offered vaccination with a licensed pneumococcal conjugate vaccine. Children who were part of the PHiD-CV group completed their vaccination schedule with PHiD-CV.

In Panama, there were two separate issues involving the informed consent process. In both cases, the situations were discussed with the IDMC, which agreed with the corrective actions described below. Ethics committees and competent authorities were consulted accordingly.

1. During the early phases of study enrolment, there was a lack of clarity in Panamanian law regarding the ability of minor parents to provide informed consent to let their child participate in a clinical study. Prompted by the findings in Colombia, a local law firm and the IRB were consulted. The sponsor was advised and made all possible efforts to follow the process to obtain consent from the grandparents and to re-consent parents when they reached legal age. Therefore the data of these children were used for the interim analysis on the primary objective of this study. However, during quality check and re-monitoring activities initiated later in 2011, it was found that re-consent had, in fact, not been obtained from some minor parents when they reached the legal age and in some cases grandparents had not been asked to confirm consent. The IRB was informed and recommended additional attempts to obtain re-consent from minor parents when they reached the legal age and, in the event re-consent could not be obtained, the IRB agreed to allow use of the data. In summary, parents of 150 children re-consented, the parents of 60 children could not be re-contacted or did not agree to re-consent and, for 53 children, the parents’ age could not be confirmed. In addition, original informed consent forms signed by the parents of 31 children in Panama were lost during re-monitoring activities. In line with guidance from the European Medicines Agency, a descriptive sensitivity analysis was conducted on the primary objective in which children with informed consent issues were excluded to confirm the validity of the interim analysis. Also, children with informed consent issues were excluded from the end of study analyses. Results of the sensitivity analysis are provided in the main text.
2. In addition, it was discovered that an incorrect version of the informed consent form for the immunogenicity subset was used in Panama. Specifically, parents were asked to sign a version of the form that included all the information on the study procedures related to efficacy endpoints but inadvertently did not specify the collection of the blood samples required for the immunogenicity analyses and completion of diary cards required for the reactogenicity analyses. However, the collection of blood samples and completion of diary cards were explained verbally to parents during the informed consent process and they followed study procedures relevant for the immunogenicity subset during the study. When the error was detected, in agreement with the Independent Ethics Committee, parents were re-contacted to confirm their agreement to the use of the immunogenicity data for their child. For 262 children, parents/guardians could not be contacted to provide consent or did not agree to the use of immunogenicity data. In addition, two children were excluded because the original informed consent forms were lost during the re-monitoring activities. Therefore, 264 children were excluded from the intent-to-treat cohort for immunogenicity. Details on the number of children involved and results of the immunogenicity analyses are described in the main text, Figure S1, and Table S7.
