# Supplementary material for: Efficacy of Pneumococcal Nontypable Haemophilus influenzae Protein D Conjugate Vaccine (PHiD-CV) in Young Latin American Children: A Double-Blind Randomized Controlled Trial
Source: PLoS Med. 2014 Jun 3;11(6):e1001657. doi: 10.1371/journal.pmed.1001657 (PMC4043495; doi:10.1371/journal.pmed.1001657)
Supplement: Text S2 — Trial protocol. (PDF) [file pmed.1001657.s013.pdf]

Sponsor:
GlaxoSmithKline Biologicals
Rue de l'Institut 89
1330 Rixensart, Belgium

**Study vaccine**	GlaxoSmithKline (GSK) Biologicals' 10-valent pneumococcal-protein D-diphtheria toxoid-tetanus toxoid conjugate vaccine 10Pn-PD-DiT
**eTrack study number and abbreviated title**	109563 (10PN-PD-DIT-028)
**Date of approval**	Final Version, 08 December 2006
**Date of approval of amendment 1**	Amendment 1, 26 November 2007
**Date of approval of amendment 2**	Amendment 2, 20 March 2008
**Date of approval of amendment 3**	Amendment 3, 25 November 2008
**Date of approval of amendment 4**	Amendment 4, 11 May 2009
**Date of approval of amendment 5**	Amendment 5, 14 December 2009
**Date of approval of amendment 6**	Amendment 6, 09 September 2010
**Title**	COMPAS: a phase III study to demonstrate efficacy of GlaxoSmithKline Biologicals' 10 valent pneumococcal vaccine (GSK1024850A) against Community Acquired Pneumonia and Acute Otitis Media
**Detailed title**	Clinical Otitis Media and Pneumonia Study (COMPAS): a phase III , double-blind, randomized, controlled, multicentre study to demonstrate the efficacy of GlaxoSmithKline (GSK) Biologicals' 10-valent pneumococcal conjugate vaccine (GSK1024850A) against Community Acquired Pneumonia (CAP) and Acute Otitis Media (AOM)

Co-ordinating author Mireille Venken, Thomas Moens PhD, Scientific Writers

Contributing authors

- Juan Pablo Yarzabal MD, MSc, Javier Ruiz Guiñazú, MD, Clinical Development Managers
- Leyla Hernandez, Raquel Merino, Kaat Vandorpe, Frederic Henry, ***Mercedes Lojo-Suarez,*** Global Study Managers
- Isabelle Henckaerts, Scientist, Clinical Immunology
- ***Sonia Schoonbroodt**, **Expert Scientist, Clinical Immunology***
- Dorota Borys, MD, Director Clinical Development (Pneumococcal vaccines)
- Lode Schuerman MD, Director, Scientific Advisor (Pneumococcal vaccines)
- Patricia Lommel, Biostatistician
- William Hausdorff PhD, Director Epidemiology
- Ricardo Ruttiman MD, Clinical Director R&D and Medical Affairs Argentina and Southern Cone

(Amended 09 September2010)

GSK Biologicals' Protocol DS V 12.3

Copyright 2006 GlaxoSmithKline group of companies. This is an open-access material distributed under the terms of the Creative Commons Attribution License (available at: http://creativecommons.org/licenses/by/3.0/legalcode), which permits use, distribution, and reproduction in any medium, provided the original author and source are properly credited, and is otherwise in compliance with the license.

**eTrack study number and abbreviated title**	109563 (10PN-PD-DIT-028)
**Date of approval**	Final Version, 08 December 2006
**Date of approval of amendment 1**	Amendment 1, 26 November 2007
**Date of approval of amendment 2**	Amendment 2, 20 March 2008
**Date of approval of amendment 3**	Amendment 3, 25 November 2008
**Date of approval of amendment 4**	Amendment 4, 11 May 2009
**Date of approval of amendment 5**	Amendment 5, 14 December 2009
**Date of approval of amendment 6**	Amendment 6, 09 September2010
**Detailed Title**	Clinical Otitis Media and Pneumonia Study (**COMPAS**): a phase III , double-blind, randomized, controlled, multicentre study to demonstrate the efficacy of GlaxoSmithKline (GSK) Biologicals' 10-valent pneumococcal conjugate vaccine (GSK1024850A) against Community Acquired Pneumonia (CAP) and Acute Otitis Media (AOM)

Note to the Editor:

Please note the following minor correction (highlighted in the text) was made to the last amended version (September 9, 2010) of this protocol:

Details on *Rotarix* vaccine formulation in Table 17, page 102 and Table 16, page 239 were blinded for Intellectual Property reasons.

Investigator Agreement

**eTrack study number and abbreviated title**	109563 (10PN-PD-DIT-028)
**Detailed Title**	Clinical Otitis Media and Pneumonia Study (**COMPAS**): a phase III , double-blind, randomized, controlled, multicentre study to demonstrate the efficacy of GlaxoSmithKline (GSK) Biologicals' 10-valent pneumococcal conjugate vaccine (GSK1024850A) against Community Acquired Pneumonia (CAP) and Acute Otitis Media (AOM)

I agree:

- To assume responsibility for the proper conduct of the study at this site.
- To conduct the study in compliance with this protocol, any mutually agreed future protocol amendments, and with any other study conduct procedures provided by GlaxoSmithKline Biologicals (GSK Biologicals).
- To ensure that all persons assisting me with the study are adequately informed about the GSK Biologicals investigational product(s) and other study-related duties and functions as described in the protocol.
- Not to implement any changes to the protocol without agreement from the sponsor and prior review and written approval from the Institutional Review Board (IRB) or Independent Ethics Committee (IEC), except where necessary to eliminate an immediate hazard to the subjects, or where permitted by all applicable regulatory requirements (for example, for administrative aspects of the study).
- That I am thoroughly familiar with the appropriate use of the vaccine(s), as described in this protocol, and any other information provided by the sponsor, including, but not limited to, the following: the current Investigator's Brochure (IB) or equivalent document, IB supplement (if applicable), prescribing information (in the case of a marketed vaccine).
- That I am aware of, and will comply with, "Good Clinical Practice" (GCP) and all applicable regulatory requirements.
- That I have been informed that certain regulatory authorities require the sponsor to obtain and supply, as necessary, details about the investigator's ownership interest in the sponsor or the investigational product, and more generally about his/her financial ties with the sponsor. GSK Biologicals will use and disclose the information solely for the purpose of complying with regulatory requirements.

Hence I:

- Agree to supply GSK Biologicals with any necessary information regarding ownership interest and financial ties (including those of my spouse and dependent children).
- Agree to promptly update this information if any relevant changes occur during the course of the study and for 1 year following completion of the study.
- Agree that GSK Biologicals may disclose any information it has about such ownership interests and financial ties to regulatory authorities.
- Agree to provide GSK Biologicals with an updated Curriculum Vitae and other FDA required documents.

Synopsis

**Detailed Title**	Clinical Otitis Media and Pneumonia Study (**COMPAS**): a phase III , double-blind, randomized, controlled, multicentre study to demonstrate the efficacy of GlaxoSmithKline (GSK) Biologicals' 10-valent pneumococcal conjugate vaccine (GSK1024850A) against Community Acquired Pneumonia (CAP) and Acute Otitis Media (AOM)
**Indication/Study population**	Three dose primary vaccination of healthy infants between 6-16 weeks of age at the time of the first vaccination, followed by a booster dose at 15-18 months
**Rationale**	This study will evaluate the efficacy of GSK Biologicals' 10-valent pneumococcal PD-conjugate vaccine against CAP and AOM up to the end of the second year of life
**Objectives**	**Primary**

To demonstrate the efficacy of a 3-dose primary course followed by a booster dose in the second year of life with the 10Pn-PD-DiT vaccine against likely bacterial CAP cases (B-CAP) in the entire study cohort. Likely bacterial CAP is defined as radiologically confirmed CAP cases with either alveolar consolidation/pleural effusion on the chest X-ray (CXR), or with non-alveolar infiltrates but with CRP $\geq$ 40 mg/L.

Criteria for efficacy:

Efficacy against likely bacterial CAP will be demonstrated if the one-sided p-value calculated for the null hypothesis Ho= [B-CAP vaccine efficacy (VE)$\leq$ 0%] is lower than the alpha level defined in Section 9.3.1.1.

Secondary

- To demonstrate the efficacy of the 10Pn-PD-DiT vaccine against clinically confirmed Acute Otitis Media (C-AOM) cases in the 7,000 children enrolled in Panama.

  Criteria for efficacy:

  Efficacy against clinically confirmed AOM will be demonstrated if the one-sided p-value calculated for the null hypothesis Ho= [C-AOM (VE)$\leq$ 0%] is lower than 2.5%

- To assess the efficacy of the 10Pn-PD-DiT vaccine against CAP with alveolar consolidation or pleural

effusion on chest X-ray (C-CAP)

- ~~To assess the efficacy of the 10Pn-PD-DiT vaccine in preventing bacteriologically confirmed AOM cases (B-AOM) caused by *Haemophilus influenzae* and vaccine serotypes, cross-reactive and other *Streptococcus pneumoniae* (in the 7,000 subjects enrolled in Panama)~~
- ***To assess the efficacy of the 10Pn-PD-DiT vaccine in preventing bacteriologically confirmed AOM cases (B-AOM) caused by any bacterial pathogen (in the 7,000 subjects enrolled in Panama)***
- ***To assess the efficacy of the 10Pn-PD-DiT vaccine in preventing bacteriologically confirmed AOM cases (B-AOM) caused by vaccine serotypes, cross-reactive and other Streptococcus pneumoniae serotypes (in the 7,000 subjects enrolled in Panama)***
- ***To assess the efficacy of the 10Pn-PD-DiT vaccine in preventing bacteriologically confirmed AOM cases (B-AOM) caused by Haemophilus influenzae (in the 7,000 subjects enrolled in Panama)***
- ***To assess the efficacy of the 10Pn-PD-DiT vaccine in preventing bacteriologically confirmed AOM cases (B-AOM) caused by non-typeable Haemophilus influenzae (in the 7,000 subjects enrolled in Panama)***
- ***To assess the efficacy of the 10Pn-PD-DiT vaccine in preventing bacteriologically confirmed AOM cases (B-AOM) caused by other AOM pathogens (for example Moraxella catarrhalis, Group A streptococci and Staphylococcus aureus) (in the 7,000 subjects enrolled in Panama)***
- To document the impact of the 10Pn-PD-DiT vaccine against ~~confirmed~~ CAP cases ***with alveolar consolidation or pleural effusion on chest X-ray (C-CAP)*** with ~~respiratory tract infections~~ ***positive respiratory viral test***
- ***To document the impact of the 10Pn-PD-DiT vaccine against CAP cases with any abnormal CXR with positive respiratory viral test***
- To document the impact of the 10Pn-PD-DiT vaccine against likely bacterial CAP (B-CAP) cases with positive respiratory viral ~~diagnostic~~ test

  (Amended 09 September2010)
- To document the impact of the 10Pn-PD-DiT vaccine against suspected CAP cases

- To document the impact of the 10Pn-PD-DiT vaccine against CAP cases with any abnormal CXR
- To document the impact of the 10Pn-PD-DiT vaccine against suspected CAP cases with CRP ≥ 40 mg/L, regardless of CXR reading
- To document the impact of the 10Pn-PD-DiT vaccine against suspected CAP cases with CRP ≥ 80 mg/L, regardless of CXR reading
- To document the impact of the 10Pn-PD-DiT vaccine against suspected CAP cases with CRP ≥ 120 mg/L, regardless of CXR reading
- To document the impact of the 10Pn-PD-DiT vaccine against CAP cases with either alveolar consolidation/pleural effusion on the chest X-ray or with non-alveolar infiltrates with CRP ≥ 80 mg/L
- To document the impact of the 10Pn-PD-DiT vaccine against CAP cases with either alveolar consolidation/pleural effusion on the chest X-ray or with non-alveolar infiltrates with CRP ≥ 120 mg/L
- To document the impact of the 10Pn-PD-DiT vaccine against bacteriologically culture-confirmed IPD caused by any of the 10 pneumococcal vaccine serotypes (VT-IPD)
- To document the impact of the 10Pn-PD-DiT vaccine in preventing VT-IPD identified through positive culture or through nonculture pneumococcal diagnosis with additional nonculture VT serotyping
- To document the impact of the 10Pn-PD-DiT vaccine in preventing invasive disease caused by cross-reactive pneumococcal serotypes, other pneumococcal serotypes and *H. influenzae*
- To document the impact of the 10Pn-PD-DiT vaccine in reducing nasopharyngeal carriage of *S. pneumoniae* (vaccine serotypes and others) and *H. influenzae* (in the ‘Carriage’subset of 2,000 children in Panama)
- To document the impact of the 10Pn-PD-DiT vaccine on antibiotic prescriptions (in the ‘Carriage’ subset of 2,000 children in Panama)
- To assess the immune response to the 10Pn-PD-DiT vaccine, one month after dose 3, before, one month and approximately 8 months after the booster dose in terms of serotype specific ELISA antibody concentrations,

serotype specific opsonophagocytic activity and anti-PD antibody responses (in a subset of 500 children in Argentina and Panama, totaling 1,000)

- To assess the reactogenicity of the 10Pn-PD-DiT vaccine in terms of solicited general and local symptoms occurring within the 4-day period after each study vaccine dose (in a subset of 500 children in Argentina and Panama, totaling 1,000)
- To assess the safety of the 10Pn-PD-DiT vaccine in terms of unsolicited adverse events occurring, starting at the administration of the first vaccine dose up to visit 9 (24-27 months of age), in the 7,000 subjects enrolled in Panama
- To assess the safety of the 10Pn-PD-DiT vaccine in terms of SAEs (all children) occurring during the entire study period starting at the administration of the first vaccine dose up to study end

Study design

- Experimental design: multicentre, randomized, controlled study with two parallel groups:
  - *10Pn-PD-DiT group*: 12,000 subjects receiving GSK Biologicals' 10-valent pneumococcal conjugate vaccine
  - *Control group*: 12,000 subjects receiving a non-pneumococcal control vaccine regimen
- Treatment allocation: balanced allocation (1:1)
- Vaccination schedule: primary vaccination course of 2-4-6 months, followed by a booster dose at 15-18 months of age.
- Blinding: double-blind/observer-blind (see Section 6.6)
- In all groups DTPa-IPV/Hib (Infanrix IPV/Hib) or DTPa-HBV-IPV/Hib (Infanrix hexa) will be co-administered according to the following immunization scheme**:**

10Pn-PD-DiT group:

Vaccine	2 mo	4 mo	6 mo	15-18 mo
10Pn-PD-DiT	X	X	X	X
DTPa-HBV-IPV/Hib	X	X	X	
DTPa-IPV/Hib				X

Control group:

Vaccine	2 mo	4 mo	6 mo	15-18 mo
HBV	X	X	X	
HAV				X
DTPa-IPV/Hib	X	X	X	X

- All subjects will participate in at least 7 scheduled visits, and a study conclusion contact.
- Three subsets of subjects will be defined:
  - ‘Immuno and reacto’ subset: 500 subjects in Panama and Argentina, totaling 1,000,
  - ‘Carriage’ subset: a subset of 2,000 subjects of all subjects enrolled in Panama
  - ‘Additional immuno’ subset: a subset of approximately 3,500 subjects of all subjects (not participating in the ‘Immuno and reacto’ subset) enrolled in Panama.
- Type of study: self-contained study
- Randomisation by a central internet randomisation program (SBIR)
- Data collection: Remote Data Entry
- Duration of the study: for each subject the duration of the study will be at least 22 months
- The target recruitment time will be approximately 18 months (depending on the time needed to complete target enrolment), starting from first subject enrolled in the study.
- ~~The study will end when the number of subjects with B-CAP required for the final analysis (see Section 9.3.1.1) will be reached, but not before a minimal efficacy follow-up up to 24-27 months of age (visit 9) for each subject was performed.~~
- ***The study end will depend on the outcome of the planned B-CAP interim analysis. If the outcome on the primary endpoint is conclusive, the study end (completion of contact 10 for each subject) will be organized as soon as possible. If the outcome is not conclusive, the study end will be organized approximately between September and December 2011.***
- ~~At the end of the study, the investigator will ask the parents/guardians of each subject in Panama if they would be willing to let their child/ward participate in a~~

~~follow-up study. If the subject's parents/guardians decline to participate in the follow-up study, refusal will be documented in the subject's eCRF~~.

(Amended 09 September2010)

Number of subjects

Total target enrolment will be 24,000 healthy male and female infants in order to obtain 21,600 evaluable subjects (10,800 in both the 10Pn-PD-DiT group and the control group) for the according-to-protocol (ATP) efficacy analysis

Primary endpoint

Occurrence of likely bacterial CAP cases (B-CAP) defined as radiologically confirmed CAP cases with either alveolar consolidation/pleural effusion on the chest X-ray or with non-alveolar infiltrates but with CRP ≥ 40 mg/L.

Secondary endpoints

Efficacy:

- Occurrence of clinically confirmed AOM cases (C-AOM) (*in all subjects enrolled in Panama*)
- Occurrence of CAP cases with alveolar consolidation or pleural effusion on the Chest X-ray (C-CAP)
- ~~Occurrence of bacteriologically confirmed AOM cases (B-AOM) caused by vaccine serotypes, cross-reactive and other pneumococcal serotypes or by *H. influenzae (in all subjects enrolled in Panama)*~~
- ***Occurrence of bacteriologically confirmed AOM cases (B-AOM) caused by any bacterial pathogen (in all subjects enrolled in Panama)***
- ***Occurrence of bacteriologically confirmed AOM cases (B-AOM) caused by vaccine serotypes, cross-reactive and other pneumococcal serotypes (in all subjects enrolled in Panama)***
- ***Occurrence of bacteriologically confirmed AOM cases (B-AOM) caused by H. influenzae (in all subjects enrolled in Panama)***
- ***Occurrence of bacteriologically confirmed AOM cases (B-AOM) caused by non-typeable H. influenzae (in all subjects enrolled in Panama)***
- ***Occurrence of bacteriologically confirmed AOM cases (B-AOM) caused by other AOM pathogens (for example Moraxella catarrhalis, Group A streptococci and Staphylococcus aureus) (in all subjects enrolled in Panama)***
- Occurrence of ~~confirmed~~ CAP cases ***with alveolar consolidation or pleural effusion on the Chest X-ray (C-***

CAP) ~~associated with respiratory viral infection~~ ***with positive respiratory viral test***

- ***Occurrence of CAP cases with any abnormal CXR with positive respiratory viral test***
- Occurrence of likely bacterial CAP (B-CAP) cases with positive respiratory viral test

(Amended 09 September2010)

- Occurrence of suspected CAP cases
- Occurrence of CAP cases with any abnormal CXR
- Occurrence of suspected CAP cases with CRP ≥ 40 mg/L, regardless of CXR reading
- Occurrence of suspected CAP cases with CRP ≥ 80 mg/L, regardless of CXR reading
- Occurrence of suspected CAP cases with CRP ≥ 120 mg/L, regardless of CXR reading
- Occurrence of CAP cases with either alveolar consolidation/pleural effusion on the chest X-ray or with non-alveolar infiltrates but with CRP ≥ 80 mg/L
- Occurrence of CAP cases with either alveolar consolidation/pleural effusion on the chest X-ray or with non-alveolar infiltrates but with CRP ≥ 120 mg/L
- Occurrence of bacteriologically culture confirmed IPD cases caused by any of the 10 pneumococcal vaccine serotypes (VT-IPD)
- Occurrence of VT-IPD cases identified through positive culture or through nonculture pneumococcal diagnostic tests (for example Binax NOW or Latex agglutination) with additional nonculture VT serotyping
- Occurrence of IPD cases due to cross-reactive pneumococcal serotypes and other pneumococcal serotypes
- Occurrence of invasive disease cases due to *H. influenzae*

Carriage *(in the 'Carriage' subset, a subgroup of 2,000 subjects enrolled in Panama)*:

- Occurrence of vaccine serotypes, cross-reactive or other *S. pneumoniae* serotypes and *H. influenzae* in the nasopharynx
- Acquisition of new *S. pneumoniae* and/or *H. influenzae* strains in the nasopharynx

Antibiotic treatment *(in the 'Carriage' subset, a subgroup of 2,000 subjects enrolled in Panama)*:

- Occurrence of antibiotic prescriptions during the entire study period

Safety *(in all subjects):*

- Occurrence of serious adverse events from the administration of the first vaccine dose up to study end

Safety (*in all subjects enrolled in Panama):*

- Occurrence of any unsolicited adverse event from the administration of the first vaccine dose up to visit 9 (24-27 months of age)

Reactogenicity *(in the 'Immuno and reacto' subset of 1,000 subjects (500 subjects respectively in Argentina and Panama)):*

- Occurrence of solicited local symptoms (redness, swelling, pain) within 4 days (day 0 – day 3) after each study vaccine administration
- Occurrence of solicited general symptoms (fever, irritability/fussiness, drowsiness, loss of appetite) within 4 days (day 0 – day 3) after each study vaccine administration

Immunogencity *(in the 'Immuno and reacto' subset of 1,000 subjects (500 subjects respectively in Argentina and Panama)):*

One month after the third vaccination (at approx. 7 months of age), just before the booster dose (at 15-18 months of age), one month post-booster dose (at 16-19 months of age) with GSK Biologicals' 10-valent pneumococcal conjugate vaccine and at last scheduled visit (24-27 months of age):

- Pneumococcal antibody concentrations against serotypes 1, 4, 5, 6A, 6B, 7F, 9V, 14, 18C, 19A, 19F and 23F ≥ 0.20 µg/mL as measured by 22F-ELISA
- Antibody concentrations against pneumococcal serotypes 1, 4, 5, 6A, 6B, 7F, 9V, 14, 18C, 19A, 19F and 23F
- Opsonophagocytic activity against pneumococcal serotypes 1, 4, 5, 6A, 6B, 7F, 9V, 14, 18C, 19A, 19F and 23F
- Antibody concentrations against protein D

- Seropositivity status, defined as:
  - Pneumococcal antibody concentrations against serotypes 1, 4, 5, 6A, 6B, 7F, 9V, 14, 18C, 19A, 19F and 23F ≥ 0.05 µg/mL
  - Opsonophagocytic activity against pneumococcal serotypes 1, 4, 5, 6A, 6B, 7F, 9V, 14, 18C, 19A, 19F and 23F ≥ 8
  - Anti-PD antibody concentrations ≥ 100 EL.U/mL

TABLE OF CONTENTS

PAGE

SYNOPSIS.....5

LIST OF ABBREVIATIONS.....22

GLOSSARY OF TERMS.....26

1. INTRODUCTION.....29
   1.1. Pneumococcal diseases.....29
   1.2 Haemophilus influenzae.....31
   1.3 Pneumococcal vaccine development.....31
   1.4 Immunological correlates of protection.....33
   1.5 Rationale for the study.....34
   1.6 Likely bacterial CAP definition.....35

2. OBJECTIVES.....37
   2.1. Primary objective.....37
   2.2. Secondary objectives.....38

3. STUDY DESIGN OVERVIEW.....40
   3.1. Study design.....40
   3.2. Detailed vaccination scheme.....43

4. STUDY COHORT.....44
   4.1. Number of subjects / centres.....44
   4.2. Inclusion criteria for enrolment.....44
   4.3. Exclusion criteria for enrolment.....45
   4.4. Exclusion criteria for further study participation.....45
   4.5. Elimination criteria during the study.....47
   4.6. Warnings and precautions.....48
   4.7. Contraindications to vaccination.....50

5. CONDUCT OF STUDY.....51
   5.1. Ethics and regulatory considerations.....51
      5.1.1. Institutional Review Board/Independent Ethics Committee (IRB/IEC).....51
      5.1.2. Informed consent.....53
   5.2. General study aspects.....55
   5.3. Subject identification.....56
   5.4. Outline of study procedures.....56
      5.4.1. Scheduled visits and study procedures for all subjects.....56
      5.4.2. Scheduled visits and study procedures for ‘Immuno and reacto’ subset.....58
      5.4.3. Scheduled visits and study procedures for ‘Additional immuno’ subset.....61
      5.4.4. Scheduled visits and study procedures for ‘Carriage’ subset.....64
   5.5. Detailed description of study stages/visits.....67
      5.5.1. Scheduled visits.....67

5.5.1.1. Scheduled visits to be performed....67
5.5.2. Disease surveillance section and unscheduled visits....79
5.5.2.1. Community Acquired Pneumonia (CAP) surveillance....79
5.5.2.1.1. Definitions....79
5.5.2.1.2. Surveillance procedures....80
5.5.2.1.3. Sample handling at local laboratories....81
5.5.2.1.4. Assessment of the severity of pneumonia....82
5.5.2.1.5. Data flow....82
5.5.2.2. Acute Otitis Media (AOM) surveillance....83
5.5.2.2.1. Case definitions....83
5.5.2.2.2. Surveillance procedures....84
5.5.2.2.3. Sample handling....86
5.5.2.2.4. Assessment of severity of AOM....87
5.5.2.2.5. Data flow....88
5.5.2.3. Invasive disease surveillance....88
5.5.2.3.1. Case definitions....88
5.5.2.3.2. Surveillance procedures....89
5.5.2.3.3. Sample handling....90
5.5.2.3.4. Assessment of the severity of invasive disease caused by S. pneumoniae or H. influenzae....93
5.5.2.3.5. Data flow....94
5.5.2.4. Data collection....94
5.6. Sample handling and analysis of biological samples during -the scheduled visits....94
5.6.1. Blood samples taken from the 'Immuno and reacto' and the 'Additional immuno' subsets....94
5.6.2. Nasopharyngeal swabs taken from the 'Carriage' subset....94
5.6.3. Microbiological assessments....95
5.7. Laboratory assays....96
5.7.1. Exploratory laboratory assays....97
5.7.1.1. Avidity anti-PD ELISA....97
5.7.1.2. Protein D enzymatic inactivation assay....97
5.7.2. Immunological read-outs....98
5.7.3. Endpoints for suboptimal response....99

6. INVESTIGATIONAL PRODUCT AND ADMINISTRATION....99
6.1. Study vaccines....99
6.2. Co-administered and concomitant vaccines....99
6.3. Dosage and administration....102
6.3.1. Injection technique....102
6.3.2. GlaxoSmithKline Biologicals' 10Pn-PD-DiT conjugate, *Engerix-B* or *Havrix* vaccines....103
6.3.3. GlaxoSmithKline Biologicals' DTPa-HBV-IPV/Hib or DTPa-IPV/Hib vaccine....103
6.4. Storage....104
6.5. Treatment allocation and randomization....105
6.5.1. Randomization of supplies....105
6.5.2. Randomization of subjects....105

6.6. Method of blinding and breaking the study blind 106
6.7. Replacement of unusable vaccine doses 106
6.8. Packaging 107
6.9. Vaccine accountability 107
6.10. Concomitant medication/treatment/vaccination 107
6.11. Concomitant vaccination 108
6.11.1. GlaxoSmithKline Biologicals' Varilrix vaccine 108
6.11.1.1. Injection technique 108
6.11.1.2. Contraindications 108
6.11.2. Neisvac-C vaccine 109
6.11.2.1. Injection technique 109
6.11.2.2. Contraindications 109
6.11.3. GlaxoSmithKline Biologicals' Rotarix vaccine 109
6.11.3.1. Contraindications 109

7. ADVERSE EVENTS AND SERIOUS ADVERSE EVENTS 110
7.1. Definition of an adverse event 110
7.2. Definition of a serious adverse event 111
7.3. Documentation of cases of disease 112
7.3.1. Documentation of any meningitis and/or invasive disease 112
7.3.2. Documentation of CAP and AOM 112
7.4. Clinical laboratory parameters and other abnormal assessments qualifying as adverse events and serious adverse events 112
7.5. Time period, frequency, and method of detecting adverse events and serious adverse events 113
7.5.1. Solicited adverse events 114
7.6. Evaluating adverse events and serious adverse events 115
7.6.1. Assessment of intensity 115
7.6.2. Assessment of causality 116
7.6.3. Medically attended visits 117
7.7. Follow-up of adverse events and serious adverse events and assessment of outcome 117
7.8. Prompt reporting of serious adverse events to GSK Biologicals 119
7.8.1. Time frames for submitting serious adverse event reports to GSK Biologicals 119
7.8.2. Completion and transmission of serious adverse event reports to GSK Biologicals 119
7.9. Regulatory reporting requirements for serious adverse events 120
7.10. Post-study adverse events and serious adverse events 120
7.11. Pregnancy 121
7.12. Treatment of adverse events 121

8. SUBJECT COMPLETION AND WITHDRAWAL 121
8.1. Subject completion 121
8.2. Subject withdrawal 121
8.2.1. Subject withdrawal from the study 121
8.2.2. Subject withdrawal from investigational product 122

9. DATA EVALUATION: CRITERIA FOR EVALUATION OF OBJECTIVES 122
9.1. Primary endpoint 122
9.2. Secondary endpoints 122
9.3. Estimated sample size 125

9.3.1. Efficacy objectives 125
9.3.1.1. Primary objective (B-CAP ~~or C-AOM~~) 125
9.3.1.2. Secondary objective (clinical AOM – C-AOM) at study end 129
9.3.1.3. Secondary objective (C-CAP) at study end 129
9.3.1.4. Secondary objective (bacteriologically confirmed AOM – B-AOM) at study end 130
9.3.1.5. Secondary objective (VT-IPD) at study end 131
9.3.2. Carriage objective in a subset of subjects 131
9.3.3. Safety objective 132
9.4. Study cohorts to be evaluated 133
9.5. Derived and transformed data 134
9.6. Final analyses (at study end: see definition in Section 3.1) 135
9.6.1. Analysis of demographics/baseline characteristics 135
9.6.2. Analysis of efficacy 135
9.6.2.1. Case accountability 135
9.6.2.1.1. Rules for CAP cases 135
9.6.2.1.2. Rules for AOM cases 136
9.6.2.1.3. Rules for ID cases 136
9.6.2.2. Analysis of primary objective 137
9.6.2.2.1. According-to-protocol (ATP) analysis = primary analysis 137
9.6.2.2.2. Total vaccinated cohort analysis 137
9.6.2.3. Analysis of secondary efficacy objectives 138
9.6.3. Analysis of carriage 139
9.6.4. Analysis of antibiotic treatment 139
9.6.5. Analysis of immunogenicity 140
9.6.6. Analysis of safety 141
9.7. Planned interim analysis 142
9.7.1. Sequence of analysis. 142
9.7.2. Consideration for the interim analysis 143

10. ADMINISTRATIVE MATTERS 143

11. REFERENCES 144

LIST OF TABLES

PAGE

Table 1 Effect of specificity of CAP case definition on VE estimates ... 35

Table 2 Effect of the use of CRP measurements on VE estimates and the assessment of the overall impact of vaccination on the CAP disease burden for non-consolidated pneumonia cases ... 35

Table 3 VE and VAR estimates in the South African trial, when including CRP in the CAP case definition ... 36

Table 4 Children at High Risk of Invasive Pneumococcal Infection* ... 47

Table 5 List of study procedures applicable for all subjects ... 56

Table 6 Intervals between ‘standard’ study visits for all subjects ... 57

Table 7 List of study procedures for subjects participating in the ‘Immuno and reacto’ subset of the study ... 59

Table 8 Intervals between study visits for the ‘Immuno and reacto’ subset ... 60

Table 9 List of study procedures for subjects participating in the ‘Additional immuno’ subset of the study ... 62

Table 10 Intervals between study visits for the ‘Additional immuno’ subset ... 63

Table 11 List of study procedures for subjects participating in the ‘Carriage’ subset of the study ... 65

Table 12 Intervals between study visits for the ‘Carriage’ subset ... 66

Table 13 Age-specific vital signs and laboratory variables indicative for SIRS ... 94

Table 14 Laboratory Assays ... 96

Table 15 Immunological read-outs ... 98

Table 16 Vaccines administered in the study ... 100

Table 17 Formulation of Vaccines ... 101

Table 18 Dosage and Administration ... 102

Table 19 Solicited local adverse events ... 114

Table 20 Solicited general adverse events ... 114

Table 21 Intensity scales for solicited symptoms ... 115

Table 22 Power for B-CAP efficacy according to the number of events at interim and final analyses 126

~~Table 23~~ ~~Likely Bacterial CAP incidences~~ 128

Table 24 Total number of first episodes required to show C-AOM VE > 0% with 80% and 90% power, according to various true VE 129

Table 25 Total number of first episodes required to show C-CAP VE > 0% with 80% and 90% power, according to various true VE 130

Table 26 Confirmed CAP incidences 130

Table 27 Total number of first episodes required to show B-AOM VE > 0% with 80% and 90% power, according to various true VE 131

Table 28 Total number of first episodes required to show VT-IPD VE > 0% with 80% and 90% power, according to various true VE 131

Table 29 Power to detect group difference in carriage of *S. pneumoniae* (vaccine serotypes) and *H. influenzae* based on fixed percentage of subjects with positive cultures (%) in Control group and various true VE (N= 1000 subjects/group) 132

Table 30 Probability to detect/rule out an increase in the percentage of subjects with rare SAE 133

LIST OF FIGURES

PAGE

Figure 1 The “Likely bacterial CAP” case definition 37

Figure 2 General study design 40

Figure 3 Study design of the ‘Immuno and reacto’ subset 58

Figure 4 Study design of the ‘Additional immuno’ subset 61

Figure 5 Study design of the ’Carriage’ subset 64

Figure 6 Procedure for follow-up of AOM cases 85

Figure 7 Projection of the number of first B-CAP episodes from 2 weeks after dose 3 (Total vaccinated cohort) (10% drop-out /year) 127

~~Figure 8~~ ~~Estimated evolution of the number of B-CAP cases~~ 128

LIST OF APPENDICES

PAGE

Appendix A World Medical Association Declaration of Helsinki 150

Appendix B Administrative Matters 154

Appendix C Handling of Biological Samples Collected by the Investigator............... 160

Appendix D Shipment of Biological Samples 163

Appendix E Vaccine supplies, packaging and accountability 164

Appendix F Definitions of radiographic outcomes and guidelines for radiographic techniques 167

Appendix G Guidelines for Middle ear Fluid collection with Channel Directed Tympanocentesis (CTD) Speculum... 171

Appendix H Procedures for the detection of *Streptococcus pneumoniae* and *Haemophilus influenzae* in each country and at the Central Laboratory.. 173

Appendix I AOM severity scales used in COMPAS 178

Appendix J Mathematical details about sample size determination......................... 181

Appendix K Amendments and Administrative Changes to the Protocol 183

List of Abbreviations

**AE**	Adverse event
**AOM**	Acute Otitis Media
**ATP**	According To Protocol
**B-AOM**	Bacteriologically confirmed AOM
**B-CAP**	Likely bacterial CAP (enlarged CAP case definition)
**C-AOM**	Clinically confirmed AOM
**CAP**	Community Acquired Pneumonia
**C-CAP**	Consolidated CAP
**CI**	Confidence Interval
**CHMP**	Committee for Human Medicinal Products
**CRA**	Clinical Research Associate
**CRF/eCRF**	Case Report Form/electronic Case Report Form
**CRM_{197}**	Non-toxic cross reacting mutant of diphtheria toxin isolated from cultures of *Corynebacterium diphtheriae* strain C7 (β197)
**CRP**	C reactive protein
**CXR**	Chest X-ray
**DT**	Diphtheria toxoid (may also be referred to as Di)
**DTP**	Diphtheria-tetanus-pertussis vaccine
**DTPa**	Diphtheria-tetanus-acellular pertussis vaccine
**DTPa-HBV-IPV/Hib**	Diphtheria-tetanus-acellular pertussis-hepatitis B-inactivated polio virus vaccine to be mixed with a lyophilized *Haemophilus influenzae* type b tetanus conjugate vaccine
**DTPa-IPV/Hib**	Diphtheria-tetanus-acellular pertussis-inactivated polio virus vaccine to be mixed with a lyophilized *Haemophilus influenzae* type b tetanus conjugate vaccine

**eCRF**	Electronic case report form
**EL.U**	ELISA units
**ELISA**	Enzyme-linked immunosorbent assay
Engerix™-B	GSK Biologicals' liquid hepatitis B vaccine, referred to as *Engerix-B* throughout the text..
**EPI**	Extended Program on Immunization
**FDA**	Food and Drug Administration, United States
**FHA**	Filamentous Haemagglutinin
**GCP**	Good Clinical Practice
**GMC**	Geometric mean antibody concentration
**GMT**	Geometric mean antibody titer
**GSK**	GlaxoSmithKline
**GSM**	Global Study Manager
Havrix™	GSK Biologicals' hepatitis A vaccine, referred to as *Havrix* throughout the text.
H. influenzae	*Haemophilus influenzae*
**Hib**	*Haemophilus influenzae* type b
**HBs**	Hepatitis B surface antigen
**HBV**	Hepatitis B virus (vaccine)
**IB**	Investigator Brochure
**ICF**	Informed Consent Form
**ID**	Invasive Disease
**ID_{50}**	50 % inhibitory dose
**IDMC**	Independant Data Monitoring Committee
**IPD**	Invasive Pneumococcal Disease
**IEC**	Independent Ethics Committee

Infanrix hexa™	GSK Biologicals' diphtheria-tetanus-acellular pertussis, hepatitis B virus-inactivated poliovirus and *Haemophilus influenzae* type b vaccine (DTPa-HBV-IPV/Hib), referred to as *Infanrix hexa* throughout the text.
**IPV**	Inactivated polio virus (vaccine)
**IRB**	Institutional Review Board
**µg**	microgram
**LPP**	Local Protocol Procedures
**mL**	millilitre
**MedDRA**	Medical Dictionary for Regulatory Activities
**MIC**	Minimal Inhibitory Concentration
**MMR**	A combined vaccine against measles, mumps and rubella.
**NC-CAP**	Non-consolidated pneumonia
Neisvac®-C	A tetanus toxoid-conjugated meningococcal C (*Neisseria meningitidis* group C) vaccine (Baxter), referred to as *Neisvac-C* throughout the text.
**NTHi**	Non-typable *Haemophilus influenzae*
**PCT**	Procalcitonine
**PD**	Protein D, a surface protein of 42 kDa, highly conserved among encapsulated and non-encapsulated froms of *Haemophilus influenzae*
**Pn**	Pneumococcal
Prevenar®* or *Prevnar®	7-valent pneumococcal conjugate vaccine with a mutant of diphtheria toxoid called CRM_{197} as protein carrier. Serotypes: 4, 6B, 9V, 14, 18C, 19F and 23F (Wyeth), referred to throughout the text as *Prevnar*.
**PRN**	Pertactin
**PRP**	Polyribosyl Ribitol Phosphate
**PS**	Polysaccharide
**PT**	Pertussis Toxoid

**Rotarix™**	GSK Biologicals' oral live attenuated HRV vaccine, referred throughout the document as *Rotarix*
**SAE**	Serious Adverse Event
S. pneumoniae	*Streptococcus pneumoniae*
**S-AOM**	Clinically suspected AOM
**SOP**	Standard Operating Procedure
**STGG medium**	Skim milk-Tryptone-Glucose-Glycerine medium
**TT**	Tetanus toxoid
Varilrix™	GSK Biologicals' varicella vaccine containing a live attenuated Oka strain varicella virus, referred to as *Varilrix* throughout the text.
**VT-IPD**	Bacteriologically culture confirmed IPD caused by any of the 10 pneumococcal vaccine serotypes.
**WHO**	World Health Organization
**10Pn-PD-DiT**	Liquid 10-valent pneumococcal conjugate vaccine with protein D and diphtheria and tetanus toxoids as protein carriers.

Glossary of Terms

**Adverse event:**	Any untoward medical occurrence in a patient or clinical investigation subject, temporally associated with the use of a medicinal product, whether or not considered related to the medicinal product.<br><br>An AE can therefore be any unfavourable and unintended sign (including an abnormal laboratory finding), symptom, or disease (new or exacerbated) temporally associated with the use of a medicinal product. For marketed medicinal products, this also includes failure to produce expected benefits (i.e. lack of efficacy), abuse or misuse.
**Blinding:**	A procedure in which one or more parties to the trial are kept unaware of the treatment assignment in order to reduce the risk of biased study outcomes. In a single-blind trial, the investigator and/or his staff are aware of the treatment assignment but the subject is not. In an observer-blind study, the subject and the study personnel involved in the clinical evaluation of the subjects are blinded while other study personnel may be aware of the treatment allocation. When the investigator and sponsor staff who are involved in the treatment or clinical evaluation of the subjects and review/analysis of data are also unaware of the treatment assignments, the study is double blind. Partially-blind is to be used for study designs with different blinding levels between different groups, e.g. double-blinded consistency lots which are open with respect to the control group. The level of blinding is maintained throughout the conduct of the trial, and only when the data are cleaned to an acceptable level of quality will appropriate personnel be unblinded or when required in case of a serious adverse event.
**Co-administered vaccines**	DTPa-IPV/Hib and DTPa-HBV-IPV/Hib (*Infanrix hexa*™) will be referred to as co-administered vaccines in this study protocol
**Concomitant vaccines**	MMR, *Varilrix*™, Rotarix***™*** and *Neisvac®-C* will be referred to as concomitant vaccines in this study protocol
**Eligible:**	Qualified for enrolment into the study based upon strict adherence to inclusion/exclusion criteria.
**eTrack:**	GSK's clinical trials tracking tool

**Evaluable:**	Meeting all eligibility criteria, complying with the procedures defined in the protocol, and, therefore, included in the according-to-protocol (ATP) analysis (see Section 9 for details on criteria for evaluability).
**Global Study Manager:**	An individual assigned by and centrally located at GSK Biologicals at Rixensart who is responsible for assuring the co-ordination of the operational aspects and proper conduct of a clinical study, including compliance with International Conference on Harmonization (ICH) Harmonized Tripartite Guideline for Good Clinical Practice (GCP) and GSK policies and standard operating procedures.
**Investigational product:**	A pharmaceutical form of an active ingredient or placebo being tested or used as a reference in a clinical trial, including a product with a marketing authorization when used in a way different from the approved form, or when used for an unapproved indication, or when used to gain further information about an approved use.
**Medical Monitor:**	An individual medically qualified to assume the responsibilities of the sponsor (GSK Biologicals) especially in regards to the ethics, clinical safety of a study and the assessment of adverse events.
**Protocol amendment:**	ICH defines a protocol amendment as: “A written description of a change(s) to or formal clarification of a protocol.” GSK Biologicals further details this to include a change to an approved protocol that affects the safety of subjects, scope of the investigation, study design, or scientific integrity of the study.
**Protocol administrative change:**	A protocol administrative change addresses changes to only logistical or administrative aspects of the study. N.B. Any change that falls under the definition of a protocol amendment (e.g. a change that affects the safety of subjects, scope of the investigation, study design, or scientific integrity of the study) MUST be prepared as an amendment to the protocol.
**Randomization:**	Process of random attribution of treatment to subjects in order to reduce bias of selection
**Site Monitor:**	An individual assigned by the sponsor who is responsible for assuring proper conduct of clinical studies at one or more investigational sites.

Solicited adverse event: Adverse events (AEs) to be recorded as endpoints in the clinical study. The presence/occurrence/intensity of these events is actively solicited from the subject or an observer during a specified post-vaccination follow-up period.

Study Monitor: An individual assigned by the sponsor who is responsible for assuring proper conduct of a clinical study.

Subject: Term used throughout the protocol to denote an individual that has been contacted in order to participate or participates in the clinical study, either as a recipient of the investigational product(s) or as a control.

Subject number: A unique number identifying a subject, assigned to each subject consenting to participate in the study.

Treatment: Term used throughout the clinical study to denote a set of investigational product(s) or marketed product(s) or placebo intended to be administered to a subject, identified by a unique number, according to the study randomization or treatment allocation.

Treatment number: A unique number identifying a treatment to a subject, according to the study randomization or treatment allocation.

Unsolicited adverse event: Any adverse event (AE) reported in addition to those solicited during the clinical study. Also any “solicited” symptom with onset outside the specified period of follow-up for solicited symptoms will be reported as an unsolicited adverse event.

1. INTRODUCTION

1.1. Pneumococcal diseases

Streptococcus pneumoniae, a gram-positive diplococcus, is a normal inhabitant of the upper respiratory tract of humans and spreads from person to person in droplets of respiratory secretions. Rates of colonization depend on a number of variables including race, age, exposure to young children and population [Ghaffar et al, 1999]. The pneumococcus is a common causative agent for a wide range of clinical diseases such as bacterial meningitis, sepsis, pneumonia and otitis media.

Streptococcus pneumoniae is considered to be the predominant cause of severe pneumonia among children in developing countries and contributes to the major share of pneumonia deaths. In these countries, *S. pneumoniae* causes one to two million deaths every year among children under five years of age [Mulholland et al, 1999; UNICEF, 2006; Wardlaw et al, 2006], most of which are due to pneumonia, and a high proportion occurs in the first year of life.The contribution of pneumococci to pneumonia has been difficult to define given the problems of establishing the bacterial etiology of pneumonia [Obaro and Madhi, 2006]. However, three studies have evaluated the impact of 7- or 9-valent CRM-conjugate vaccines on WHO defined “consolidated” radiographic pneumonia (irrespective of the etiological agent) and showed a 20.5 to 37.0 percent reduction in radiologically confirmed pneumonia (9.0 percent for HIV-positive individuals) [Black et al, 2002; Cutts et al, 2005; Klugman et al, 2003; Hansen et al, 2006]. In addition; recent analysis of data from the 9-valent CRM-conjugate pneumococcal vaccine trial in South Africa [Madhi et al, 2005aa; Madhi et al, 2006] has demonstrated that the typical consolidated CAP cases only represent a relatively small proportion of the CAP disease burden that is preventable by pneumococcal vaccines (i.e. the sensitivity of the consolidated CAP case definition is not optimal). This means that the true public health impact (and cost-effectiveness) of a pneumococcal conjugate vaccine may be markedly underestimated if only alveolar consolidation is included.

S. pneumoniae is also a major cause of acute otitis media (AOM). Despite the widespread use of antibiotics, complications of pneumococcal AOM still occur, including secretory otitis media, mastoiditis and meningitis, and often need further interventional therapy [Bluestone et al, 2000]. The disease is most prevalent in early childhood, with the peak age-specific attack-rate occurring from 6 to 18 months of age. It has been reported that approximately 60% of children have had at least one episode of AOM by one year of age and that more than 20 million cases occur each year in children under two years in the US [Teele et al, 1989]. Infants with severe and recurrent otitis media with persistent middle ear infection are at risk for behavioural problems and poor development of speech, language and cognitive abilities [Klein et al, 2001].

AOM is the most frequent bacterial infection of children and the primary reason for the prescription of antibiotics by paediatricians [Cohen et al, 1996; McCaig et al, 1995]. The average physician may examine more than 14,000 ears each year [Pichichero, 2000a]. Nevertheless, the medical literature suggests that AOM is frequently overdiagnosed [Rosenfeld, 2002]. The gold standard for the diagnosis of AOM in clinical trials is

tympanocentesis for the determination of the presence of Middle Ear Fluid (MEF) with subsequent culture for identification of causative pathogens [Berman, 1995; Giebink 1991; Klein, 1994; Pichichero, 1998a; Pichichero, 1998b] In the clinical context, tympanocentesis could also be indicated in hyperallergic children, when the tympanum is highly bulging, to document bacterial etiology (by culture and antibiogram) and in antibiotic treatment failures [François, 1993].

In studies performed in Latin America and Europe [Rosenblüt et al, 2001; Arguedas et al, 2003; Arguedas et al, 2005; Hausdorff et al, 2002; Eskola et al, 2001; Prymula et al, 2006], on middle ear fluid obtained through tympanocentesis from children with AOM, the most common bacterial pathogen causing AOM was *S. pneumoniae* (33-62%). Between 3 and 49 month of age the most common *pneumococci* serotypes isolated from middle ear fluid (MEF) were: 19F (26.1%), 6B (14.5%), 9V (8.7%), 16F (8.7%), 14 (5.8%), 23F (5.8%), 3 (5.8%) and 6A (5.8%) [Arguedas et al, 2005]. Serotype distribution was similar in children younger than 24 months of age or 24 month of age or older.-

S. pneumoniae has also been identified as a major cause of invasive disease.The highest incidence of invasive pneumococcal disease (IPD) is found in children under two years of age and in elderly adults [Butler et al, 1996]. The annual incidence of IPD in industrialized countries has been reported to be as high as 160/100 000 in children under two years of age [Hausdorff et al, 2000]. Since the introduction of widespread routine immunization with Prevnar in the US (June 2000), the estimated incidence in the US of invasive pneumococcal infections among children aged < 12 months and 12-23 months has fallen with incidences for 2002 of 38.5/100 000 and 31.5/100 000 population, respectively [Centers for Disease Control and Prevention, 2002]. In Western Europe, reported rates of IPD are much lower than the IPD rates in the US prior to licensure of Prevnar, due to different blood-culture rates and practices compared to those in the US, leading to an under-reporting of milder cases of pneumococcal bacteremia [Hausdorff et al, 2001]. In Latin America however, high incidences of invasive disease were observed in children under 23 months of age. In a three-year study spanning 2000-2003, population-based surveillance of invasive disease of children 2-23 months old in Argentina revealed an incidence of 207 cases/100,000 children-year [Tregnaghi et al, 2006]. Slightly less than half was due to hospitalized cases, with the rest seen as outpatients. In the same study, rates of community-acquired pneumonia (all etiologies) with dense consolidation seen upon X-ray were approximately 10-fold higher, at 2422 cases/10^5 children/year. Only a small fraction of cases were definitively identified as pneumococcal by blood and pleural fluid culture, which yielded an incidence of 95 cases/10^5 children/year. In Chile, a surveillance study of invasive disease caused by *S. pneumoniae* in hospitalized children was performed in 2 administrative regions of Santiago from 1994 to 1996 [Levine et al, 1998]. Annual incidence of invasive disease in children less than 2 years was 78.3/100,000 children-year (60.2/100,000 in infants age 0-11 months and 18.1/100,000 in toddlers age 12-23 months.

The prevalence of multiple antibiotic resistant pneumococci has increased dramatically over the past decade [Appelbaum et al, 1996; Whitney et al, 2000; McCormick et al, 2003] and this has prompted further efforts to develop vaccination for the prevention of pneumococcal diseases in infants. Vaccination could also be an effective way to decrease

the carriage and spread of antibiotic-resistant pneumococci, especially in crowded settings such as day-care centres [Dagan et al, 2001].

1.2 *Haemophilus influenzae*

Haemophilus influenzae is a small gram-negative cocco-bacillus exclusively pathogenic for humans, and the infection it causes range from asymptomatic colonization of the upper respiratory tract to serious invasive diseases such as meningitis (Plotkin & Mortimer – Second Edition). Although *H. influenzae* type B disease is now virtually eliminated in areas with high rates of Hib immunization, infections caused by non-typable *H. influenzae* remain a significant cause of respiratory tract infections.

H. influenzae together with *S. pneumoniae* have been identified as the major pathogens involved otitis media. Studies performed in Latin America and Europe [Rosenblüt et al, 2001; Arguedas et al, 2003; Arguedas et al, 2005; Hausdorff et al, 2002; Eskola et al, 2001; Prymula et al, 2006] to document the etiology of AOM in children based on MEF culture obtained through tympanocentesis, showed that *H. influenzae* was the second most common pathogen after *S. pneumoniae* isolated in 20-34% of the MEF samples.

Haemophilus influenzae plays also an important role in the colonization of the nasopharynx. In countries worldwide, between 30 and 50% of children around 2 years of age are colonized with this pathogen [García-Rodríguez et al, 2002]. The reported rates of bacterial acquisition and carriage vary extensively between different studies and geographical areas. These differences have been related to genetic background variables and socio-economic conditions including housing, access to health care, poor hygiene, family size, overcrowded living conditions, day-care contact, number of siblings, etc.

1.3 Pneumococcal vaccine development

The polysaccharide capsule is the most important virulence factor of *S. pneumoniae* and contributes to the progression of the disease by virtue of its antiphagocytic properties [Mitchell et al, 1997]. Ninety serologically distinct serotypes of *S. pneumoniae* have been described, varying in the structure of their polysaccharide capsule [Henrichsen et al, 1995]. Pneumococcal vaccines based on immunization with capsular polysaccharide have been licensed since 1977. The licensed 23-valent unconjugated plain polysaccharide vaccines are designed to provide coverage of approximately 90% of the most frequently reported isolates in the US and most other countries [Martindale, 1999]. However, a satisfactory immune response is not obtained in children under two years of age, and their use in this age group is therefore not recommended.

Furthermore, the protection induced with polysaccharide vaccines is short-lived, i.e. no immunological memory is formed because of the T-cell independent nature of polysaccharide antigens [Dintzis, 1992]. Polysaccharide antigens can however be made to induce a T-cell-response by their covalent coupling with proteins. *S. pneumoniae* polysaccharide conjugate vaccines are currently licensed or under development with the aim of producing T-cell dependent antigens which yield boostable antibody levels after repeated injection, promote antibody class switching and affinity maturation, provide protection to children under 2 years of age and induce immunological memory. Various

carrier proteins have been used in *S. pneumoniae* conjugate vaccines that underwent clinical testing: diphtheria and tetanus toxoid, CRM_{197} (a non-toxic cross-reacting mutant diphtheria toxin molecule), and lipo-polysaccharide depleted group B meningococcal outer membrane protein complex [Klein, 1999].

To date, one 7-valent pneumococcal conjugate vaccine has been licensed for use in several parts of the world, under the trade name of Prevnar. This 7-valent pneumococcal conjugate vaccine comprises capsular polysaccharides from pneumococcal serotypes 4, 6B, 9V, 14, 18C, 19F and 23F, each conjugated to CRM_{197}. Efficacy was 97.4 % (95 % confidence interval: 82.7–99.9 %) in preventing vaccine-serotype IPD [Black et al, 2000], 21 % against X-ray confirmed pneumonia [Black et al, 2002] and 57 % against vaccine-serotype pneumococcal AOM [Eskola et al, 2001]. Since June 2000, the 7-valent pneumococcal conjugate vaccine has been recommended in the US for routine immunization for all children under two years of age and for children aged 24-59 months who are at high risk for pneumococcal infections [Centers for Disease Control and Prevention, 2004]. In Europe, Prevenar has been recommended for universal mass vaccination in several countries including Germany, Spain, Belgium, France and UK.

Following licensure of Wyeth's 7-valent pneumococcal conjugate vaccine (Prevnar) in the USA and in Europe, regulatory approval of new pneumococcal conjugate vaccines for the invasive disease indication will be based on immunological criteria, in comparison to Prevnar. In June 2003, a WHO meeting reached a consensus towards a threshold of response that could be used as a basis for licensure: the percentage of subjects with a pneumococcal antibody concentration above 0.35 µg/mL using an ELISA without pre-adsorption with 22F polysaccharide, or equivalent depending on the ELISA technique used (e.g. 0.20 µg/mL using GSK Biologicals' 22F ELISA) [Henckaerts et al, 2006; WHO, 2005]. Although the use of a single threshold for all serotypes is expected to be acceptable for the demonstration of non-inferiority to Prevnar, a consensus, based on currently available efficacy data [Klugman et al, 2003; O'Brien et al, 2003], has not been reached on a correlate of protection for IPD in children.

GSK Biologicals produced an 11-valent pneumococcal conjugate vaccine that uses protein D (PD) as carrier (11Pn-PD vaccine) and contains four additional serotypes to those in Prevnar (serotypes 1, 3, 5 and 7F). Protein D is a 42 kD cell-surface lipoprotein which is highly conserved among capsulated and unencapsulated strains of *H. influenzae* and has been shown to enhance clearance of *H. influenzae* in the chinchilla AOM model [Bakaletz et al, 1999]. The 11Pn-PD vaccine was tested in a large AOM efficacy trial (Undeca-Pn-010, POET) conducted in the Czech and Slovak Republics [Prymula et al, 2006]. Results from the POET trial demonstrated a statistically significant and clinically relevant protective effect of vaccination on the overall AOM disease burden (33.6%), as well as on the number of AOM episodes caused by both vaccine *S. pneumoniae* serotypes (57.6%) or *H. influenzae* (35.6%). These results therefore confirmed the potential of the PD carrier protein to provide additional protection against *H. influenzae*.The lack of protective efficacy against AOM caused by serotype 3, together with other data indicating an impaired serotype 3 antibody response to the 11Pn-PD booster dose in the second year of life, led the company to decide to remove serotype 3 from the final vaccine formulation to give the 10-valent pneumococcal conjugate vaccine 10Pn-PD-DiT.

Furthermore, in order to optimise the immune response, GSK Biologicals developed other candidate vaccine formulations in which each pneumococcal polysaccharide was separately conjugated to either protein D (PD), diphtheria toxoid (DT) or tetanus toxoid (TT). A series of different 11–valent vaccine formulations was tested in Phase II feasibility trials in order to evaluate the use of DT or TT as protein carriers for some of the serotypes and the impact of different dosages of polysaccharide. Results from these studies have led to the selection of a final 10-valent formulation (10Pn-PD-DiT) for final development and licensure.

Please refer to the current Investigator Brochure for a review of the pre-clinical and clinical studies.

1.4 Immunological correlates of protection

At present, widely accepted immunological correlates of protection exist for certain antigens only and consist of defined humoral antibody responses above which there is a high likelihood of protection in the absence of any host factors that might increase susceptibility to the infectious agent [CHMP, 2005].

In POET, GSK Biologicals' 11-valent pneumococcal PD-conjugate vaccine was demonstrated to provide 35.6% [3.8-57.0] protection against AOM episodes caused by Non-typeable Haemophilus influenzae [Prymula et al, 2006]. In addition, a statistically significant reduction of 42.6% [1.3-66.6] was observed in nasopharyngeal carriage of H. influenzae. These findings are of clinical significance in view of the importance of this organism as a major cause of AOM and lower respiratory tract infections, and given the predominant role taken by NTHi as cause of recurrent or refractory AOM in the US since introduction of Prevenar.

Based on these observations and following the CHMP recommendations [CHMP, 2005],an effort should be made to describe the correlation between the immune response against Protein D and the protective efficacy against H. influenzae. In POET, a post-primary vaccination blood sample was taken from all 4,968 subject enrolled in the study, for the purpose of exploring such a correlation. Apart from the protocol defined randomly selected immunogenicity subset of approximately 300 subjects, serum samples were not tested, but stored for further evaluation in the context of the assessment of the correlate of protection. After the POET study end and database unblinding, blood samples from the approximately 100 subjects that developed an AOM episode caused by NTHi, were further evaluated to characterize the immune response against protein D. No correlate could however so far be identified that would predict protection against NTHi AOM espisodes or H. influenzae carriage based on evaluation of anti-PD immune responses following primary vaccination only.

Ideally, confirmation of an immunological correlate for protection (at least in the short-term) should be based on exploration of immune responses in at least a subset of vaccinees during clinical studies of protective efficacy. Evaluation and characterization of the responses induced by the PD carrier protein before and after booster vaccination are considered necessary to further attempt fully understanding the relationship with the protection provided against NTHi. Therefore, the study design foresees a subset of

subjects in which pre- and post-booster vaccination blood samples will be drawn and stored for further exploration after study end of the correlation between protection against AOM episodes caused by (Non-typeable) Haemophilus influenzae and results of the measurement and characterization of responses induced by the PD carrier protein.

1.5 Rationale for the study

Pneumococcal vaccination may be one of the most important new developments in immunization in the coming decade, with the potential to prevent substantial morbidity and mortality due to lower respiratory tract infections in large parts of the world. Respiratory tract infections are however of complex nature, and although a wide variety of infectious agents are known to be able to cause pneumonia, including *Streptococcus pneumoniae* and *Haemophilus influenzae*, the bacterial identification of the causal agent of pneumonia is notoriously difficult. It has therefore been difficult in the past to generate clear evidence of the public health impact of vaccines on pneumonia disease burden in children, resulting in substantial delays in introducing Hib conjugate vaccines in many parts of the world.

One way to document the amount of disease preventable by vaccination is through a randomized controled clinical trial ("a vaccine probe study") [Mulholland, 2004]. It has been argued that the existence of an effective vaccine precludes the conduct of a controlled trial unless the trial is a comparison with the licensed vaccine. In the case of pneumococcal vaccination the issue is unclear. First because, even if a pneumococcal conjugate vaccine is licensed, it is not widely available to those populations where the disease burden is high, but secondly because different vaccines may be more or less effective under different conditions. Therefore comparative data to a licensed vaccine, for which the pneumonia impact in the involved countries has not yet clearly been documented, might not provide sufficiently robust data to guide decision makers. In the case of GSK's pneumococcal conjugate vaccine, the use of the PD carrier protein potentially increases the possible impact on lower respiratory tract infections, since in addition to reducing disease caused by pneumococci, also *Haemophilus influenzae* disease might be impacted. This makes it even more important that GSK's 10Pn-PD-DiT vaccine is evaluated in those populations where the public health impact of the vaccine is potentially big, in order to generate data that will allow governments and policy makers to decide for vaccine introduction.

In addition to pneumonia, the pneumococcus causes a variety of other conditions including sometimes lifethreatening invasive pneumococcal diseases such as meningitis and severe bacteraemia, or usually less serious but extremely frequent acute otitis media. For these reasons, COMPAS is designed to demonstrate the efficacy of GSK Biological’s 10Pn-PD-DiT pneumococcal conjugate vaccine against “likely bacterial” CAP (B-CAP) in Argentina, Colombia and Panama or against clinically confirmed AOM cases (C-AOM) in subjects enrolled in Panama. In addition, impact on CAP with alveolar consolidation or pleural effusion on chest x-ray (C-CAP), bacteriologically confirmed AOM and ID caused by S. pneumoniae and H. influenzae will be evaluated. Finally the correlation will be explored between protection against AOM caused by (Non-typeable) Haemophilus influenzae and the measurement and characterization of responses induced by the PD carrier protein.

1.6 Likely bacterial CAP definition

Results published by Shabir Madhi [Madhi et al, 2005b] illustrate clearly that the more specific the case definition used, the higher the vaccine efficacy estimate, but because sensitivity of the case definition decreases at the same time, the overall impact on the disease burden expressed as Vaccine Attributable Reduction (VAR) decreases (Table 1).

Table 1 Effect of specificity of CAP case definition on VE estimates

	n vaccine	n control	Efficacy (%) (95% CI)	VAR*
Clinical LRTI	1033	1106	7 (-1 to 14)	172
Clinical severe pneumonia (WHO def)	591	662	11 (1 to 20)	164
Clinical pneumonia (WHO def)	566	681	17 (7 to 26)	267
Pneumonia with alveolar consolidation	169	212	20 (3 to 35)	100
Vaccine serotype bacteraemic pneumonia	2	6	67 (-65 to 93)	9

*VAR= Vaccine Attributable Reduction (in cases/100,000)
Madhi, 2005

Alternative case definitions have been analysed to identify suitable criteria that would increase the sensitivity of the pneumonia case definition, without reducing its specificity.

C-reactive protein (CRP) is an acute phase protein that is almost undetectable in serum from normal subjects, but that can be found in high concentrations in case of inflammatory processes (such as bacterial infections or rheumatoid arthritis), malignancies and tissue necrosis such as myocardial infarction. CRP increases very rapidly, sometimes even before the sedimentation rate is increased, with also a rapid return to normal levels.

The results shown in Table 2 involve CRP in the pneumonia case definition, and are based on a post-hoc analysis performed on a subset of the data from the South African 9-valent CRM efficacy trial (subjects enrolled during the specific time window in the trial and for which CRP measurement was performed) [Madhi et al, 2006]. The following table shows results for HIV negative suspected CAP cases for which the CXR did not reveal alveolar consolidation infiltrates.

Table 2 Effect of the use of CRP measurements on VE estimates and the assessment of the overall impact of vaccination on the CAP disease burden for non-consolidated pneumonia cases

	n vaccine	n control	Efficacy (%) (95% CI)	VAR*
Pneumonia without alveolar consolidation				
Clinical LRTI	570	579	1.6 (-10.3 to 12.2)	48
Clinical severe pneumonia (WHO def)	316	351	10.0 (-4.6 to 22.6)	188
CRP ≥ 40 mg/L	85	124	31.5 (9.8 to 48.0)	209
CRP ≥ 80 mg/L	35	59	40.7 (10.0 to 60.9)	129
CRP ≥ 120 mg/L	19	32	40.6 (-4.7 to 66.3)	70

*VAR= Vaccine Attributable Reduction (in cases/100,000)
Madhi, 2006

From these analyses, the CRP criterion (≥40 mg/L) appears to be the most promising in terms of providing a high sensitivity (high VAR and therefore increased power to demonstrate efficacy in a vaccine study) and maintaining a high specificity (high vaccine efficacy (VE)). It would also reduce the chance of underestimating the burden of pneumococcal pneumonia that is preventable with a pneumococcal conjugate vaccine [Madhi, 2007]. Therefore, an endpoint called "likely bacterial CAP" (defined as radiologically confirmed CAP cases with either alveolar consolidation/pleural effusion or with non-alveolar infiltrates but with CRP ≥ 40mg/L), was chosen to measure the efficacy of the 10Pn-PD-DiT vaccine in this study.

The extension of the alveolar consolidated CAP case definition by the addition of X-ray confirmed CAP cases without alveolar consolidation but with increased CRP was evaluated by Madhi [2007]. Results confirm that the "likely bacterial CAP" endpoint allows better description of the overall reduction of lower respiratory tract infections following pneumococcal vaccination, while maintaining specificity and therefore vaccine efficacy (Table 3).

Table 3 VE and VAR estimates in the South African trial, when including CRP in the CAP case definition

	VE	(95% CI)	VAR1	(95% CI)
Clinical LRTI with abnormal Chest X-ray	9	(-7; 22)	150	(-117; 367)
Severe LRTI2 with abnormal Chest X-ray	9	(-11; 25)	90	(-110; 250)
CXR-AC	15	(-6; 32)	134	(54; 286)
Any abnormal Chest X-ray with CRP ≥40 mg/L	22	(7; 35)	350	(111; 557)
Any abnormal Chest X-ray with CRP ≥120 mg/L	19	(1; 34)	204	(11; 365)

[1] VAR: Vaccine Attributable Reduction expressed as cases prevented per 100,000 children in the study
[2] Severe LRTI according to WHO definition
Madhi, 2007

Procalcitonin (PCT, at a low threshold (≥ 0.25 ng/mL) has shown to be useful in directing the use of antibiotics in adults with pneumonia [Christ-Crain et al, 2004], and in a recent meta-analysis it showed to offer some advantages over CRP for discriminating bacterial from non-bacterial infections [Simon et al, 2004]. Recent observations [Madhi et al, 2005a] demonstrated that many children with viral associated pneumonia have a bacterial superinfection; however high levels of CRP and PCT may be associated with bacterial co-infection. Therefore, although not taken into account for the Likely bacterial CAP definition, serum from each suspected CAP case will be analysed to measure procalcitonin levels.

Figure 1 The "Likely bacterial CAP" case definition

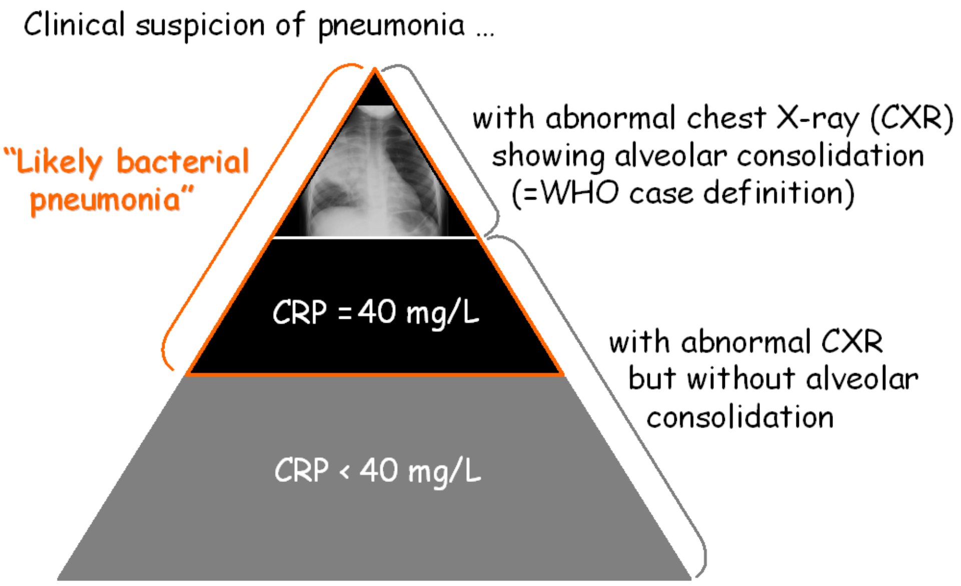

2. OBJECTIVES

2.1. Primary objective

To demonstrate the efficacy of a 3-dose primary course followed by a booster dose in the second year of life with the 10Pn-PD-DiT vaccine against likely bacterial CAP cases (B-CAP) in the entire study cohort. Likely bacterial CAP is defined as radiologically confirmed CAP cases with either alveolar consolidation/pleural effusion on the chest X-ray (CXR), or with non-alveolar infiltrates but with CRP ≥ 40 mg/L.

Criteria for efficacy:

Efficacy against likely bacterial CAP will be demonstrated if the one-sided p-value calculated for the null hypothesis Ho= [B-CAP vaccine efficacy (VE)≤ 0%] is lower than the alpha level defined in Section 9.3.1.1.

Refer to Section 9.1 for definition of the primary endpoint.

2.2. Secondary objectives

- To demonstrate the efficacy of the 10Pn-PD-DiT vaccine against clinically confirmed Acute Otitis Media (C-AOM) cases in the 7,000 children enrolled in Panama.

  Criteria for efficacy:

  Efficacy against clinically confirmed AOM will be demonstrated if the one-sided p-value calculated for the null hypothesis Ho= [C-AOM (VE)$\leq$ 0%] is lower 2.5%.

- To assess the efficacy of the 10Pn-PD-DiT vaccine against CAP with alveolar consolidation or pleural effusion on chest X-ray (C-CAP)
- ~~To assess the efficacy of the 10Pn-PD-DiT vaccine in preventing bacteriologically confirmed AOM cases (B-AOM) caused by *Haemophilus influenzae* and vaccine serotypes, cross-reactive and other *Streptococcus pneumoniae* (in the 7,000 subjects enrolled in Panama)~~
- ***To assess the efficacy of the 10Pn-PD-DiT vaccine in preventing bacteriologically confirmed AOM cases (B-AOM) caused by any bacterial pathogen (in the 7,000 subjects enrolled in Panama)***
- ***To assess the efficacy of the 10Pn-PD-DiT vaccine in preventing bacteriologically confirmed AOM cases (B-AOM) caused by vaccine serotypes, cross-reactive and other Streptococcus pneumoniae serotypes (in the 7,000 subjects enrolled in Panama)***
- ***To assess the efficacy of the 10Pn-PD-DiT vaccine in preventing bacteriologically confirmed AOM cases (B-AOM) caused by Haemophilus influenzae (in the 7,000 subjects enrolled in Panama)***
- ***To assess the efficacy of the 10Pn-PD-DiT vaccine in preventing bacteriologically confirmed AOM cases (B-AOM) caused by non-typeable Haemophilus influenzae (in the 7,000 subjects enrolled in Panama)***
- ***To assess the efficacy of the 10Pn-PD-DiT vaccine in preventing bacteriologically confirmed AOM cases (B-AOM) caused by other AOM pathogens (for example Moraxella catarrhalis, Group A streptococci and Staphylococcus aureus) (in the 7,000 subjects enrolled in Panama)***
- To document the impact of the 10Pn-PD-DiT vaccine against ~~confirmed~~ CAP cases ***with alveolar consolidation or pleural effusion on chest X-ray (C-CAP)*** with ~~respiratory tract infections~~ ***positive respiratory viral test***
- ***To document the impact of the 10Pn-PD-DiT vaccine against CAP cases with any abnormal CXR with positive respiratory viral test***
- To document the impact of the 10Pn-PD-DiT vaccine against likely bacterial CAP (B-CAP) cases with positive respiratory viral ~~diagnostic~~ test

  (Amended 09 September2010)

- To document the impact of the 10Pn-PD-DiT vaccine against confirmed CAP cases with respiratory tract infections
- To document the impact of the 10Pn-PD-DiT vaccine against suspected CAP cases
- To document the impact of the 10Pn-PD-DiT vaccine against CAP cases with any abnormal CXR
- To document the impact of the 10Pn-PD-DiT vaccine against suspected CAP cases with CRP ≥ 40 mg/L, regardless of CXR reading
- To document the impact of the 10Pn-PD-DiT vaccine against suspected CAP cases with CRP ≥ 80 mg/L, regardless of CXR reading
- To document the impact of the 10Pn-PD-DiT vaccine against suspected CAP cases with CRP ≥ 120 mg/L, regardless of CXR reading
- To document the impact of the 10Pn-PD-DiT vaccine against CAP cases with either alveolar consolidation/pleural effusion on the chest X-ray or with non-alveolar infiltrates with CRP ≥ 80 mg/L
- To document the impact of the 10Pn-PD-DiT vaccine against CAP cases with either alveolar consolidation/pleural effusion on the chest X-ray or with non-alveolar infiltrates with CRP ≥ 120 mg/L
- To document the impact of the 10Pn-PD-DiT vaccine on likely bacterial CAP (B-CAP) with positive respiratory viral diagnostic test
- To document the impact of the 10Pn-PD-DiT vaccine against bacteriologically culture-confirmed IPD caused by any of the 10 pneumococcal vaccine serotypes (VT-IPD)
- To document the impact of the 10Pn-PD-DiT vaccine in preventing VT-IPD identified through positive culture or through nonculture pneumococcal diagnosis with additional nonculture VT serotyping
- To document the impact of the 10Pn-PD-DiT vaccine in preventing invasive disease caused by cross-reactive pneumococcal serotypes, other pneumococcal serotypes and *H. influenzae*
- To document the impact of the 10Pn-PD-DiT vaccine in reducing nasopharyngeal carriage of *S. pneumoniae* (vaccine serotypes and others) and *H. influenzae* (in the ‘Carriage’subset of 2,000 children in Panama)
- To document the impact of the 10Pn-PD-DiT vaccine on antibiotic prescriptions (in the ‘Carriage’ subset of 2,000 children in Panama)
- To assess the immune response to the 10Pn-PD-DiT vaccine, one month after dose 3, before, one month and approximately 8 months after the booster dose in terms of serotype specific ELISA antibody concentrations, serotype specific opsonophagocytic activity and anti-PD antibody responses (in a subset of 500 children in Argentina and Panama, totaling 1,000)

- To assess the reactogenicity of the 10Pn-PD-DiT vaccine in terms of solicited general and local symptoms occurring within the 4-day period after each study vaccine dose (in a subset of 500 children in Argentina and Panama, totaling 1,000)
- To assess the safety of the 10Pn-PD-DiT vaccine in terms of unsolicited adverse events occurring, starting at the administration of the first vaccine dose up to visit 9 (24-27 months of age), in the 7,000 subjects enrolled in Panama
- To assess the safety of the 10Pn-PD-DiT vaccine in terms of SAEs (all children) occurring during the entire study period starting at the administration of the first vaccine dose up to study end

Refer to Section 9.2 for definitions of secondary endpoints.

3. STUDY DESIGN OVERVIEW

3.1. Study design

Figure 2 General study design

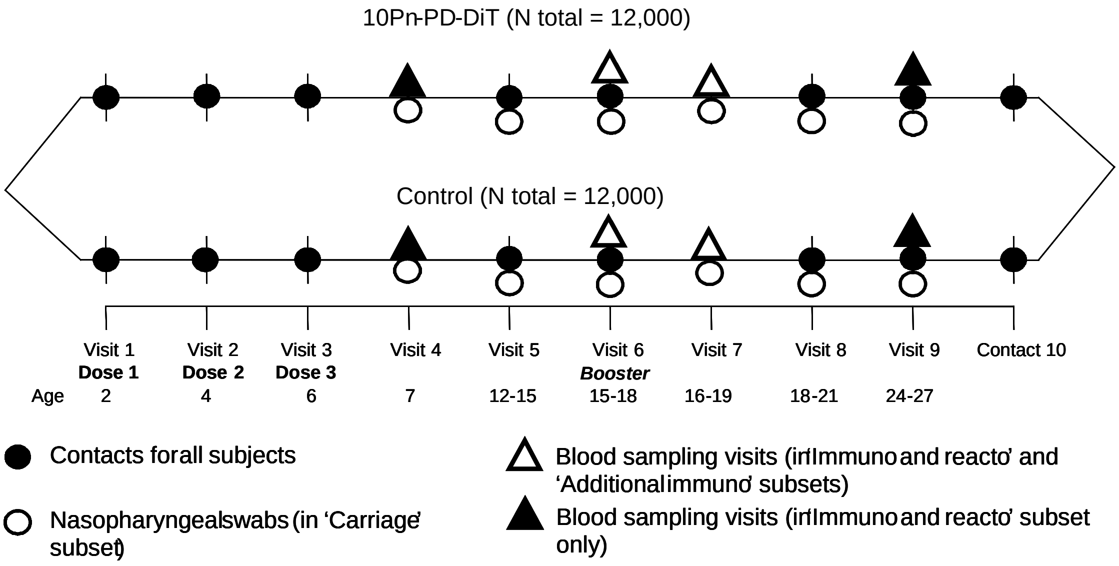

Note: although age windows for different visits sometimes overlap, study procedures for different visits can not be grouped at one visit, and intervals between visits as defined in Section 5.4 must be respected.

- Experimental design: multicenter, randomized, controlled trial with two parallel groups:
  - 10Pn-PD-DiT group: 12,000 subjects receiving GSK Biologicals' 10-valent pneumococcal conjugate vaccine
  - Control group: 12,000 subjects receiving a non-pneumococcal control vaccine regimen (detailed vaccination scheme see Section 3.2)
- The trial will be conducted in Argentina, Colombia and Panama.

- Blinding: double blind/observer-blind (see Section 6.6).
- Treatment allocation: balanced allocation (1:1)
- Three-dose primary vaccination course starting at 6 to 16 weeks of age, with allowable intervals between two consecutive primary doses ranging from 49 to 83 days. Booster dose will be administered at 15-18 months of age.
- DTPa-IPV/Hib (*Infanrix* IPV/Hib) or DTPa-HBV-IPV/Hib (*Infanrix hexa*) vaccine will be co-administered to all subjects.
- A MMR vaccine will be administered as a concomitant vaccine to all subjects in the three countries at Visit 5 (12-15 months of age), as per local recommendations and country availability.
- HAV vaccine: All subjects will receive 2 doses of HAV vaccine, the first dose at 12 months of age, the second dose at 18-21 months of age. The last HAV dose, given at 18-21 months of age, should be given at least 28 days after the study vaccine booster dose, because the control group also receives a HAV dose at 15-18 months of age as part of the blinded study vaccines. These vaccine administrations cover the local hepatitis A vaccine recommendations (one dose of Hep A vaccine by the age of 12 months in Argentina and two doses of hepatitis A vaccine at 12 and 18 months in Colombia and Panama).
- In Argentina, licensed Neisvac-C vaccine against *Neisseria meningitidis* group C will be offered to all subjects (also subjects in the 10Pn-PD-DiT vaccine group) at 12 months of age in order to provide a benefit to the control group.
- In Colombia and Panama, licensed Varicella vaccine will be offered to all subjects (also subjects in the 10Pn-PD-DiT vaccine group) at 12 months of age in order to provide a benefit to the control group.
- In Colombia, 2 doses of licensed Rotarix vaccine will be offered to all subjects (also subjects in the 10Pn-PD-DiT vaccine group) within the first six months of life.
- Type of study: self-contained study
- Randomisation via a central internet randomisation program (SBIR)
- Data collection: Remote Data Entry (RDE)
- Duration of the study: for each subject the duration of the study will be at least 22 months.
- Target recruitment time will be approximately 18 months (depending on the time needed to complete target enrolment), starting from first subject enrolled.
- All subjects will contribute to the efficacy evaluation against CAP and ID as well as to the overall safety (SAEs) assessment. Only subjects from Panama will contribute to the efficacy evaluation against AOM.
- Scheduled vaccination visits will take place approximately at 2, 4, 6 and 15-18 months of age. In addition, two visits for HAV vaccination according to EPI at 12-15 and 18-21 months of age respectively and a last visit at 24-27 months of age will-be

scheduled-in all subjects. A study conclusion contact will be scheduled for all subjects at study end.

- Risk factors for pneumococcal and/or H.influenzae infections (agammaglobulinemia, HIV infection, sickle cell disease, nephrotic syndrome, chronic renal failure, organ transplantation, diabetes mellitus, congestive heart failure and CSF leaks), breastfeeding history and composition of the household will be checked at Visits 1, 5 and 9.
- Unscheduled visits will take place for all subjects each time an episode of CAP, AOM or ID occurs.
- A nested study population of approximately 1,000 subjects (500 in respectively Argentina and Panama) ('Immuno and reacto' subset) will contribute to the assessment of solicited symptoms and the assessment of vaccine immunogenicity. This nested population will consist of subjects attending predetermined health care facilities, and additional scheduled visits will be organised at 7 and 16-19 months of age to accommodate with the additional immunogenicity assessments. Blood samples will be taken one month post primary schedule (7 months of age), just before booster (15-18 months of age), one month after booster (16-19 months of age) and at the last scheduled visit (24-27 months of age).
- Unsolicited adverse events will only be followed in all subjects enrolled in Panama.
- Acute otitis media cases will be followed in the complete study population of 7,000 subjects enrolled in Panama. Additional unscheduled visits will take place when AOM episodes occur in this population, and samples of MEF will be obtained for bacteriological confirmation of the AOM episode, upon signature of a specific informed consent form.
- For determination of nasopharyngeal carriage, a subset of 2,000 children (which is a subgroup of all subjects enrolled in Panama) ('Carriage' subset) will have nasopharyngeal swab samples taken at 6 time points during the study period: at 7 months of age, at 12-15 months of age, at the moment of the booster injection (15-18 months of age), approximately one month and 3 months after the booster dose, and when the subject will reach 24-27 months of age.
- 'Additional immuno' subset: a subset of approximately 3,500 children (not participating in the 'Immuno and reacto' subset) in Panama will have two blood samples taken: just before the booster injection (Visit 6 at 15-18 months of age) and one month post booster (Visit 7 at 16-19 months of age), to be stored and used after study end for exploring the correlation of protein D related responses with occurrence of AOM caused by non-typable Haemophilus influenzae (NTHi)**.**
- The study is designed in order to reach the ~~628~~ 535 B-CAP cases ~~and the 628 C-AOM cases~~ required for the interim analysis (see Section 9.3.1.1) approximately 24 months after study start. The evaluation of ~~these~~ ***this*** primary endpoint~~s~~ can however be delayed in case accumulation of cases is slower than expected. ~~If accumulation of cases is faster, the analysis of one or both of the primary endpoints can be done earlier, but a minimum study duration of 18 months after study start will be respected in order to allow a clinically meaningful duration of follow-up in a sufficient number of subjects. Efficacy follow-up will continue for all subjects until study end.~~

- ~~The study will end when the number of subjects with B-CAP required for the final analysis (see Section 9.3.1.1) will be reached, but not before a minimal efficacy follow-up up to 24-27 months of age (visit 9) for each subject was performed.~~
- ***The study end will depend on the outcome of the planned B-CAP interim analysis. If the outcome on the primary endpoint is conclusive, the study end (completion of contact 10 for each subject) will be organized as soon as possible. If the outcome is not conclusive, the study end will be organized approximately between September and December 2011.***
- ~~At the end of the study, the investigator will ask the parents/guardians of each subject in Panama if they would be willing to let their child/ward participate in a follow-up study. If the subject's parents/guardians decline to participate in the follow-up study, refusal will be documented in the subject's eCRF.~~
- For individual subjects, study completion will be documented by a study conclusion contact.

  (Amended 09 September2010)

3.2. Detailed vaccination scheme

Group	Vaccine	2 mo	4 mo	6 mo	15-18 mo
10Pn-PD-DiT	10Pn-PD-DiT	X	X	X	X
	Infanrix hexa	X	X	X	
	DTPa-IPV/Hib				X
Control	HBV	X	X	X	
	HAV				X
	DTPa-IPV/Hib	X	X	X	X

In addition to these blinded study vaccines, the following vaccines will be administered or are recommended in Agentina, Colombia and Panama:

Hepatitis B vaccination at birth is recommended in the three countries

MMR vaccine is recommended in the three countries at 12 months of age. According to these recommendations MMR vaccine provided by the local Expanded Program on Immunization (EPI) will be administered to all subjects at 12 months of age.

To comply with local recommendations for hepatitis A vaccination (one dose of HAV vaccine by the age of 12 months in Argentina and two doses of HAV vaccine at 12 and 18 months of age in Colombia and Panama), all subjects will receive 2 doses of HAV vaccine, the first dose at 12 months of age, and the second dose at 18-21 months of age. The last HAV dose should be given at least 28 days after the study vaccine booster dose, because the control group also receives a HAV dose at 15-18 months of age as part of the blinded study vaccines.

In Argentina, licensed Neisvac-C vaccine against Neisseria meningitidis group C will be offered to all subjects (also subjects in the 10Pn-PD-DiT vaccine group) at 12 months of age in order to provide a benefit to the control group.

In Colombia and Panama, licensed Varicella vaccine will be offered to all subjects (also subjects in the 10Pn-PD-DiT vaccine group) at 12 months of age in order to provide a benefit to the control group.

In Colombia, 2 doses of licensed Rotarix vaccine will be offered to all subjects (also subjects in the 10Pn-PD-DiT vaccine group) within the first six months of life.

4. STUDY COHORT

4.1. Number of subjects / centres

For all study centers together, the target enrolment will be 24,000 healthy male and female infants between 6 and 16 weeks of age (between 42 and 118 days) to be enrolled over a 12 month period in order to obtain 21,600 evaluable subjects (10,800 in both the 10Pn-PD-DiT group and the control group) for the ATP efficacy analysis. If enrolment would be delayed, the sample size could be adapted in order to maintain the estimated timing of the interim analysis 24 months after study start. The sample size might need to be adapted according to the review of the global incidence of B-CAP and C-AOM cases during the study. The IDMC will be involved in the review of the enrolment and case incidences in order to recommend measures to be taken by GSK (see Section 5.2 on IDMC). See Section 9.3 for a detailed description of the criteria used in the estimation of sample size.

4.2. Inclusion criteria for enrolment

All subjects must satisfy the following criteria at study entry:

- A male or female between, and including, 6 and 16 weeks of age (between 42 and 118 days) at the time of the first vaccination. Pre-term born infants (born after a gestation period of less than 37 weeks) can be included in the study starting from 8 weeks of chronological age at the time of first vaccination and up to 16 weeks of chronological age (between 56 and 118 days).
- In each site, subjects should be living in the area covered by the surveillance system for CAP, ID and AOM (see country specific LPPs).
- Written informed consent obtained from the parent or guardian of the subject.
- Free of any known or suspected health problems (as established by medical history and clinical examination before entering into the study), that would contraindicate the initiation of routine immunizations outside a clinical trial context.

- Subjects for whom the investigator believes that their parents/guardians can and will comply with the requirements of the protocol (e.g., completion of the diary cards, return for follow-up visits).

4.3. Exclusion criteria for enrolment

The following criteria should be checked at the time of study entry. If any apply at the time of study entry, the subject must not be included in the study:

- Use of any investigational or non-registered drug or planned use during the study period.
- Previous and planned concomitant vaccinations:
- Use or planned use of any investigational or non-registered vaccine other than the study vaccine(s).
- Previous vaccination against diphtheria, tetanus, pertussis, *Haemophilus influenzae* type b, hepatitis A and/or *S. pneumoniae*. Locally recommended EPI vaccines to be given at birth (such as BCG, Hepatitis B, or OPV) are allowed, but should be administered at least one month before the first dose of the study vaccine.Other locally recommended vaccines (such as influenza or rotavirus vaccines, recommended either through the EPI program or through national immunization campaigns) are always allowed, even if concomitantly administered with the study vaccines, but should be documented in the eCRF.
- Previous or planned vaccination with a registered pneumococcal vaccine such as Prevnar is not allowed. If Prevnar immunization needs to be initiated, due to the presence of a high risk disease for pneumococcal infections for which the Prevnar vaccine is made locally available, the subject can not be enrolled in the study and should be referred to the specific Prevnar immunization program.
- History of allergic disease or reactions likely to be exacerbated by any component of the vaccines.
- History of any neurologic disorders or seizures.
- Acute disease at the time of enrolment (Acute disease is defined as the presence of a moderate or severe illness with or without fever. All vaccines can be administered to persons with a minor illness such as diarrhoea, mild upper respiratory infection with or without temperature increase, i.e oral/axillary/tympanic temperature <37.5°C / rectal temperature <38.0°C). Presence of acute disease and/or temperature greater than the above-mentioned cut-offs warrants delay of enrolment until the illness has improved.
- For Colombia: infants with low birth weight (<2.500g).

4.4. Exclusion criteria for further study participation

The following criteria should be checked for all subjects at each scheduled and unscheduled visit subsequent to the first visit.

- High risk disease for pneumococcal infections is defined by the guidelines of the American Academy of Paediatrics [American Academy of Pediatrics, 2000] and also the Argentinean Paediatric Society [Normas Nacionales de Vacunacion, 2008] (see Table 4). Diagnosis of high risk disease for pneumococcal infections will be evaluated according to medical judgement (as established by medical history and clinical examination) and confirmed when needed by the appropriate test(s). In case of diagnosis of a high risk condition for pneumococcal infection requiring pneumococcal conjugate vaccination in a subject enrolled in the study, the subject will be unblinded in order to guide appropriate individual management of the case:
  - All subjects will continue study vaccination and follow-up according to the procedures planned in the protocol. The date of unblinding for these subjects will be encoded in the e CRF and efficacy follow-up data collected after this date will no longer contribute to the primary efficacy analysis.
  - Subjects randomized to the 10Pn-PD-DiT vaccine group will not receive additional vaccines, since they already received pneumococcal conjugate vaccination in addition to the DTPa-HBV-IPV/Hib study vaccine.
  - Subjects randomized to the control group will need to receive a licensed pneumococcal conjugate vaccine according to the age-appropriate immunization schedule, in addition to the DTPa-IPV/Hib, HBV and HAV study vaccines.
  - If a licensed Pneumococcal conjugate vaccine is made available through a targeted national immunization program for these high risk groups, the investigator will make sure that the subjects belonging to these high risk groups will receive that vaccine. If no locally licensed Pneumococcal conjugate vaccine is available, it will be provided to the investigator by GSK Biologicals.
  - Administration of the licensed pneumococcal conjugate vaccine will be documented in the eCRF.
- For each case, where by mistake, vaccines with antigens common to the antigens contained in the study or co-administered vaccines are administered outside of the context of the study, the investigator will need to evaluate whether the subject can still continue participation in the study.

Table 4 Children at High Risk of Invasive Pneumococcal Infection*

High risk	
1	Sickle Cell Disease (SCD), congenital or acquired asplenia, or splenic dysfunction
2	Infection with HIV
Presumed high risk (attack rate not calculated)	
1	Congenital immune deficiency: some B- (humoral) or T-lymphocyte deficiencies, complement deficiencies (particularly C1, C2, C3, and C4 deficiencies), or phagocytic disorders (excluding chronic granulomatous disease).
2	Chronic cardiac disease (particularly cyanotic congenital heart disease and cardiac failure)
3	Chronic pulmonary disease (including cystic fibrosis, rheumatic pneumonia, tuberculosis, idiopatic intersticial difuse fibrosis of the lung, pulmonary aspergilosis and asthma treated with high-dose oral corticosteroid therapy defined as more than 14 days of prednisone or equivalent, ≥0.5 mg/kg/day. Inhaled and topical steroids are allowed)
4	Cerebrospinal fluid leaks
5	Chemotherapy
6	Chronic renal insufficiency, including nephrotic syndrome
7	Chronic hepatopathy
8	Diseases associated with immunosuppressive therapy or radiation therapy (including malignant neoplasms, leukemias, lymphomas, and Hodgkin’s disease) and solid organ transplantation**
9	Diabetes mellitus
10	Multiple myeloma
11	Preterm infants (born after gestation period ≤ 32 weeks) and/or a birth weight ≤ 1.500g (for Argentina only)

* Adapted from [American Academy of Pediatrics, 2000] and [Normas Nacionales de Vacunacion, 2008]

4.5. Elimination criteria during the study

The following criteria should be checked for all subjects at each scheduled and unscheduled visit subsequent to the first visit. If any of the following elimination criteria become applicable during the study, withdrawal of the subject from the study will not necessarily be required, but the subject may no longer be considered eligible for the according-to-protocol (ATP) immunogenicity and efficacy analyses. See Section 9.4 for definition of study cohorts to be evaluated.

Elimination from ATP analyses for immunogenicity and efficacy:

- Chronic administration (defined as more than 14 days) of immunosuppressants or other immune-modifying drugs during the study period. For corticosteroids, this will mean prednisone, or equivalent, ≥ 0.5 mg/kg/day. Inhaled and topical steroids are allowed.
- Major congenital defects or serious chronic illness.
- Any confirmed or suspected immunosuppressive or immunodeficient condition based on medical history and physical examination (no laboratory testing required).
- Administration of immunoglobulins and/or any blood derived products during the study period.

In addition for each scheduled visit:

Elimination from safety and immunogenicity ATP analyses:

- Use of any investigational or non-registered product (drug or vaccine) other than the study vaccine(s) during the entire study period.

4.6. Warnings and precautions

The vaccines used in this study could possibly induce similar side effects to those commonly observed after the administration of licensed vaccines such as:

- Local reactions at injection site: pain, redness and swelling.
- General reactions: irritability, crying, loss of appetite, vomiting, diarrhoea, fever, drowsiness and restlessness.

These local and general reactions rarely last longer than 24 hours.

Pneumococcal vaccine

The 10-valent pneumococcal conjugate candidate vaccine has been administered safely in more then 3,500 infants. In addition, related 11-valent pneumococcal conjugate vaccine formulations have been administered to >8,000 subjects for primary immunization and to >4,600 subjects as booster dose and proved to be safe. These vaccines only contain pneumococcal capsular polysaccharide and single bacterial carrier proteins and will therefore not cause pneumococcal or *Haemophilus influenzae* diseases.

DTPa-HBV-IPV/Hib (Infanrix hexa) or DTPa-IPV/Hib (Infanrix IPV/Hib)

Infanrix hexa and *Infanrix* IPV/Hib has been safely administered to a large number of children and will not cause diphtheria, tetanus, pertussis (whooping cough), hepatitis B, polio or *Haemophilus influenzae* type b disease.

The child/ward may feel pain or discomfort at the injection site and you may see redness or swelling at the injection site. However, these effects usually clear up within a few days. Other side effects which can occur are: diarrhoea, loss of appetite, vomiting, fever (more than 38°C), sleepiness, irritability, abnormal crying, restlessness, nervousness, seizures or fits, upper respiratory tract infection, bronchitis, skin rash, red skin rash, hives, injection site reactions including hard lump at the site of injection and fatigue.

The following adverse events constitute precautions specific to DTPa vaccine administration. If any of these adverse experiences occurs, the subject may be withdrawn at the discretion of the investigator.

- Fever of ≥ 40.5°C (rectal temperature) or ≥ 40.0°C (oral, axillary or tympanic temperature) within 48 hours of vaccination.
- Collapse or shock-like state (hypotonic-hyporesponsive episode) within 48 hours of vaccination.

- Persistent, inconsolable crying occurring within 48 hours of vaccination and lasting ≥ 3 hours.
- Seizures with or without fever occurring within 3 days of vaccination.

Additional side effects that have been reported very rarely (less than 1 per 10,000 doses of vaccine) in the days after vaccination with *Infanrix hexa* include: collapse or periods of unconsciousness or lack of awareness, swollen glands in the neck, armpit or groin, bleeding or bruising more easily than normal and diffuse swelling of the entire injected limb.

As observed with other DTPa-containing vaccines, cases of large swelling reactions of the injected limb, involving an increase of circumference, and sometimes involving the entire injected limb, have been very rarely reported following the booster dose administered in the second year of life. These reactions in general began within 48 hours of vaccination and have spontaneously resolved over an average of 4 days without sequelae. As in other pertussis containing vaccines, extremely rare cases of collapse or shock-like state (hypotonic-hyporesponsiveness episode) and convulsions within 2 to 3 days of vaccination have been reported. All the subjects recovered without sequelae.

Vaccine against hepatitis A (Havrix) and against hepatitis B (Engerix B)

Past clinical studies have shown that *Havrix* and *Engerix B* can be given concomitantly with *Infanrix hexa*, *Infanrix* IPV/Hib and pneumococcal conjugate vaccines. The studies demonstrated that the immune responses and the safety profiles of the administered vaccines were unaffected.

Varicella vaccine (Varilrix)

As for other varicella vaccines, cases of varicella disease have been shown to occur in persons who have previously received *Varilrix*. These breakthrough cases are usually mild, with a fewer number of lesions and less fever and cough with respect to cases in unvaccinated individuals.

Transmission of the Oka vaccine virus has been shown to occur at a very low rate in seronegative contacts of vaccinees. However, transmission has not been confirmed to occur in the absence of vaccine-associated cutaneous lesions in the vaccinee.

Varilrix should not be administered intradermally. Varilrix must under no circumstances be administered intravenously.

Men C vaccine (NeisVac-C)

Vaccination with *NeisVac-C* will not cause meningococcal group C disease.

From the side effects reported in the patient information leaflet for *NeisVac-C* the following were not listed above: relapse of certain kidney disorders, purple spots or blotches under the skin, nausea, muscle pain and reduced muscle tone, abnormal or reduced sensation.

Human rotavirus vaccine (Rotarix)

Rotarix is a vaccine that is administered orally and should under no circumstances be be injected. The administration of Rotarix should be postponed in subjects suffering from diarrhoea or vomiting.

From the side effects commonly reported after administration of Rotarix the following were not listed above: flatulence, abdominal pain, regurgitation of food and fatigue. Uncommonly constipation was reported. Side effects rarely reported, for Rotarix include: upper respiratory tract infection, hoarseness, rhinorrhoea, dermatitis, rash, muscle cramp and gastroenteritis.

Contacts of recent Rotarix vaccinees should be advised to observe personal hygiene (e.g. wash their hands after changing child's nappies). In a large safety trial in which 63,225 subjects were vaccinated with either Rotarix or with placebo, there was no evidence of an increased risk of intussusception (part of the gut pulls inward like a telescope) in the Rotarix group as compared with the placebo group.

4.7. Contraindications to vaccination

The following adverse events (AEs) constitute absolute contraindications to further administration of study or co-administered vaccines. If any of these adverse events occur during the study, the subject must not receive additional doses of vaccine but may continue other study procedures at the discretion of the investigator (see Section 8). The subject must be followed until resolution of the event, as with any AE (see Section 7.7):

- Anaphylactic reaction following previous administration of vaccine(s).
- Hypersensitivity to any component of the study or co-administered vaccines (10Pn-PD-DiT, DTPa-based combination vaccines, *Havrix* and *Engerix-B*) or signs of hypersensivity following previous immunizations

The following AEs constitute contraindications to administration of study vaccine at that point in time; if any one of these AEs occurs at the time scheduled for vaccination, the subject may be vaccinated at a later date, within the time window specified in the protocol (see Section 5.4), or withdrawn at the discretion of the investigator (see Section 8). The subject must be followed until resolution of the event, as with any AE (see Section 7.7).

- Acute disease at the time of vaccination. (Acute disease is defined as the presence of a moderate or severe illness with or without fever. All vaccines can be administered to persons with a minor illness such as diarrhea, mild upper respiratory infection with or without low-grade fever, i.e. Oral/Axillary/Tympanic temperature <37.5°C / Rectal temperature <38.0°C). In case of acute disease, the study visit should be postponed until the illness has improved.
- Febrile illness defined as oral, axillary or tympanic temperature ≥ 37.5°C, rectal temperature ≥ 38.0°C. A temperature greater than or equal to these cut-offs warrants deferral of the vaccination pending recovery of the subject.

DTPa-HBV-IPV/Hib (*Infanrix hexa*) or DTPa-IPV/Hib (*Infanrix* IPV/Hib)

The following adverse events constitute absolute contraindications to administration of DTPa-HBV-IPV/Hib or DTPa-IPV/Hib; if any of these adverse events occur during the study, the investigator must decide which vaccine to give to the subject for these antigens:

Absolute contraindications:

- These vaccines are contra-indicated if the infant has experienced an encephalopathy, defined as an acute, severe central nervous system disorder occurring within 7 days following vaccination and generally consisting of major alterations in consciousness, unresponsiveness, generalized or focal seizures that persist more than a few hours, with failure to recover within 24 hours.

N.B. Contraindication to the administration of the DTPa-HBV-IPV/Hib or the DTPa-IPV/Hib vaccines does not constitute contraindication to the administration of the pneumococcal vaccine. Children who will not receive one of these vaccines may still receive the study pneumococcal vaccine: the decision is left to the discretion of the investigator.

5. CONDUCT OF STUDY

5.1. Ethics and regulatory considerations

The study will be conducted according to Good Clinical Practice (GCP), the 1996 Declaration of Helsinki (see Appendix A), and local rules and regulations of the country.

Submission of the protocol and any protocol amendments to regulatory agencies will occur in accordance with local regulatory requirements. For some countries, submission to the local regulatory authority may not be required. When submission to the local regulatory authority is required, the timing of the submission relative to IEC/IRB submission or approval and whether or not the authority will provide their approval of or favourable opinion on the protocol or amendment before it can be implemented will depend on local regulatory requirements.

5.1.1. Institutional Review Board/Independent Ethics Committee (IRB/IEC)

The IRB/IEC must be constituted according to the local laws/customs of each participating country. The ICH Harmonized Tripartite Guideline for Good Clinical Practice recommends that the IRB/IEC should include:

a. At least five members.

b. At least one member whose primary area of interest is in a non-scientific area.

c. At least one member who is independent of the institution/ study site.

Only those IRB/IEC members who are independent of the investigator and the sponsor of the study should vote/ provide opinion on a study-related matter.

A list of IRB/IEC members with their professions and qualifications should be obtained by the investigator.

This protocol and any other documents that the IRB/IEC may need to fulfil its responsibilities, including subject recruitment procedures and information about payments and compensation available to subjects, will be submitted to the IRB/IEC by the investigator. Written and dated unconditional approval/favourable opinion from the IRB/IEC of the protocol and amendment (if any and applicable), written informed consent form, consent form updates (if any), subject recruitment procedure(s) (e.g. advertisements), and any other written information to be provided to subjects must be in the possession of the investigator and GSK before commencement of the study. This approval/favourable opinion must refer to the study by study title and number with exact protocol version and date, and should identify the documents reviewed and state the date of review. Relevant GSK Biologicals' data will be supplied by the investigator to the hospital/ university/ independent IRB/IEC for review and approval of the protocol. Verification of the unconditional approval/favourable opinion of the IRB/IEC will be transmitted by the investigator to GSK Biologicals' Study Monitor prior to shipment of vaccine supplies.

No deviations from, or changes to, the protocol should be initiated without prior written sponsor and IRB/IEC approval/ favourable opinion of an appropriate amendment, except when necessary to eliminate immediate hazards to the subjects or where permitted by all applicable regulatory requirements or when the change(s) involves only logistical or administrative aspects of the study (e.g., change of monitor[s], telephone number[s].) Administrative changes and amendments not submitted for approval are submitted to the IRB/IEC for information only. However, written verification that such documents were submitted should be obtained. Approvals/ verifications must be transmitted in writing GSK Biologicals' Study Monitor by the investigator.

The IRB/IEC must be informed by the investigator of:

- all subsequent protocol amendments, informed consent changes or revisions of other documents originally submitted for review,
- serious and/or unexpected adverse events occurring during the study, where required,
- all subsequent protocol administrative changes (for information, except for US studies),
- new information that may affect adversely the safety of the subjects or the conduct of the study,
- an annual update and/or request for re-approval, where required,
- when the study has been completed, where required.

If a trial is prematurely terminated or suspended for reasons including, but not limited to, safety or ethical issues or severe non-compliance, the sponsor will promptly inform the

regulatory authorities of the termination or suspension and the reason(s) for the termination or suspension. If required by applicable regulations, the investigator must inform the IEC/IRB promptly and provide the reason for the suspension or termination (see Appendix B for further details).

5.1.2. Informed consent

In obtaining and documenting informed consent, the investigator should comply with the applicable regulatory requirement(s), and should adhere to GCP and to the ethical principles that have their origin in the 1996 version of the Declaration of Helsinki. Prior to the beginning of the trial, the investigator should have the IRB/IEC's written approval/favourable opinion of the written informed consent form and any other written information to be provided to the subjects' parents/guardians.

Freely given informed consent should be obtained from every subjects' parents/guardians prior to clinical trial participation.

Information should be given in both oral and written form whenever possible and as deemed appropriate by the IRB/IEC.

An investigator or designee will describe the protocol to potential subjects' parents/guardians face to face. The Informed Consent Form may be read to the subjects' parents/guardians, but, in any event, the investigator or designate shall give the subjects' parents/guardians ample opportunity to inquire about details of the study and ask any questions before dating and signing the Informed Consent Form.

While informed consent information can be presented to groups at an initial information session, each subject's parents/guardians must be given the opportunity to individually pose questions to the investigator or designate prior to the subject's parents/ guardians dating and signing the Informed Consent Form.

The Informed Consent Form must be in a language fully comprehensible to the prospective subjects' parents/guardians. Informed consent shall be documented by the use of a written consent form approved by the IRB/IEC and signed and dated by the parents/guardians and by the person who conducted the informed consent discussion. The signature confirms the consent is based on information that has been understood. Specific attention should be given to:

- Illiterate individuals: all illiterate individuals will have the study, and the Informed Consent Form explained to them point by point by the interviewer in the presence of at least one impartial witness following local regulations. The witness will personally sign and date the consent form. Oral witnessed consent will replace written consent only in countries where the local custom is contrary or if the parents'/guardians' incapacity precludes this and provided that the local legal obligations are fulfilled.
- Minors (< 18 years of age): local law should be applied.

Each subject's signed informed consent form must be kept on file by the investigator for possible inspection by Regulatory Authorities and/or GSK Biologicals' professional and

Regulatory Compliance persons. The parents/guardians should receive a copy of the signed and dated written informed consent form and any other written information provided to the subjects' parents/guardians, and should receive copies of any signed and dated consent form updates. Any amendments to the written information will be provided to subjects' parents/guardians.

Both the informed consent discussion and the written informed consent form and any other written information to be provided to the subjects' parents/guardians should include explanations of the following:

a. That the trial involves research.

b. The purpose of the trial.

c. The trial treatment(s) and the probability for random assignment to each treatment.

d. The trial procedures to be followed, including all invasive procedures, and all diagnostic procedures that will be undertaken in the case the study subject would develop CAP, Invasive Disease and/or AOM. Specific approval to perform tympanocentesis (separate section in the AOM ICF) will be solicited by the ENT specialist to the subjct's parents/guardians prior to the tympanocentesis procedure in case of AOM occurring in a subject participating in the AOM subset in Panama.

e. The subject's parents'/guardians' responsibilities.

f. Those aspects of the trial that are experimental.

g. The reasonably foreseeable risks or inconveniences to the subjects and, when applicable, to an embryo, fetus or nursing infant.

h. The reasonable expected benefits. When there is no intended clinical benefit to subjects, and/or subjects' parents/guardians should be made aware of this.

i. The alternative procedure(s) or course(s) of treatment/ methods of prevention that may be available to subjects, and their important potential benefits and risks.

j. The compensation and/or treatment available to subjects in the event of trial-related injury.

k. The anticipated prorated payment, if any, to subjects' parents/guardians for participating in the trial.

l. The anticipated expenses, if any, to parents/guardians for participating in the trial.

m. That the subjects' participation in the trial is voluntary and subjects' parents/guardians may refuse to participate or withdraw from the trial, at any time, without penalty or loss of benefits to which subjects are otherwise entitled.

n. That the monitor(s), the auditor(s), the IRB/IEC, and the regulatory authority(ies) will be granted direct access to the subject's original medical records for verification of clinical trial procedures and/or data, without violating the confidentiality of subjects, to the extent permitted by the applicable laws and regulations and that, by signing a written informed consent, the subject's parents/guardians is authorizing such access.

o. That records identifying subjects will be kept confidential and, to the extent permitted by the applicable laws and/or regulations, will not be made publicly available. If the results of the trial are published, subjects' identity will remain confidential.

p. That the subjects' parents/guardians will be informed in a timely manner if information becomes available that may be relevant to the subjects' parents/guardians willingness for continued participation in the trial.

q. The person(s) to contact for further information regarding the trial and the rights of trial subjects, and who to contact in the event of trial-related injury.

r. The foreseeable circumstances and/or reasons under which a subject's participation in the trial may be terminated.

s. The expected duration of a subject's participation in the trial.

t. The approximate number of subjects involved in the trial.

GSK Biologicals will prepare a model Informed Consent Form which will embody all the elements described above. While it is strongly recommended that this model document be followed as closely as possible, the informed consent requirements given in this document are not intended to pre-empt any local regulations which require additional information to be disclosed for informed consent to be legally effective. Clinical judgement, local regulations and requirements should guide the final structure and content of the document.

The investigator has the final responsibility for the final presentation of Informed Consent Form, respecting the mandatory requirements of local regulations. The consent form generated by the investigator with the assistance of the sponsor's representative, must be approved (along with the protocol, and any other necessary documentation) by the IRB/IEC and be acceptable to GSK Biologicals.

5.2. General study aspects

An Independent Data Monitoring Committee (IDMC) will be organised for this study to protect the ethical and safety interests of the subjects recruited, while securing as far as possible the scientific validity of the data. The IDMC will review primary and secondary endpoint cases, to identify potential treatment harm and all causes of mortality/morbidity and to identify potential treatment benefit.

Responsibilities of the IDMC include the following:

– Review of data collection methods, safety/efficacy monitoring procedures and making recommendations for additions or adjustments, as applicable. However, visits to the investigational sites should be avoided once the study has started in order to avoid unblinding or any other potential bias.

– Recommendations for maintaining, or breaking the blind where necessary, in the course of reviewing the results. Specification of the procedure for unblinding when needed, e.g.complete unblinding or designated A and B unblinding.

– Recommendations for stopping rules for efficacy/safety when appropriate.

5.3. Subject identification

Subject numbers will be assigned sequentially to subjects consenting to participate in the study, according to the range of subject numbers allocated to each study centre.

5.4. Outline of study procedures

During the study, there will be scheduled (see Table 5 and Section 5.5.1) and unscheduled visits (see Section 5.5.2). Unscheduled visits will take place on an as needed basis, and will be reported in the specific sections of the eCRF of each study subject.

5.4.1. Scheduled visits and study procedures for all subjects

All study subjects, participating in the study, will have at least seven scheduled visits and a final study conclusion contact (see Table 5).

Table 5 List of study procedures applicable for all subjects

Study visit<br><br>Age of subject<br><br>Study timing	Visit 1<br><br>6-16 weeks<br>Month 0	Visit 2<br><br>± 4 months<br>Month 2	Visit 3<br><br>± 6 months<br>Month 4	Visit 4[1]<br><br>± 7 months<br>Month 5	Visit 5<br><br>12-15 months<br>Month 10-13	Visit 6<br><br>15-18 months<br>Month 13-16	Visit 7[1]<br><br>16-19 months<br>Month 14-17	Visit 8<br><br>18-21 months<br>Month 16-19	Visit 9<br><br>24-27 months<br>Month 22-25	Contact 10<br><br><br>Study end
Informed consent	●									
Check inclusion criteria	●									
Check exclusion criteria	●									
Demography, including gestational age	●									
Medical history	●									
Pre-vaccination history	●									
Check elimination criteria		●	●		●	●		●	●	
Check exclusion criteria for further study participation		O	O		O	O		O	O	
Check risk factors	●				●				●	
Check warnings, precautions and contraindications	O	O	O		O	O		O		
Physical examination	●	●	●		●	●		●	●	
Weight/height recording	●	●	●			●			●	
Pre-vaccination body temperature	●	●	●			●				
Randomization	●									
Vaccination (study and co-administered vaccines)	●	●	●			●				
Vaccination (concomitant vaccines)[2]					●			●		

Study visit<br><br>Age of subject<br><br>Study timing	VISIT 1<br><br>6-16 weeks<br>Month 0	VISIT 2<br><br>± 4 months<br>Month 2	VISIT 3<br><br>± 6 months<br>Month 4	VISIT 4[1]<br><br>± 7 months<br>Month 5	VISIT 5<br><br>12-15 months<br>Month 10-13	VISIT 6<br><br>15-18 months<br>Month 13-16	VISIT 7[1]<br><br>16-19 months<br>Month 14-17	VISIT 8<br><br>18-21 months<br>Month 16-19	VISIT 9<br><br>24-27 months<br>Month 22-25	CONTACT 10<br><br><br>Study end
Record any concomitant vaccination	●	●	●		●	●		●	●	
Reporting of unsolicited Adverse Events[3]		●	●		●	●		●	●	
Reporting of convulsions[4]								●		
Reporting of Serious Adverse Events		●	●		●	●		●	●	●
Study Conclusion										●

Note:
● is used to indicate a study procedure that requires documentation in the individual CRF (eCRF).
O is used to indicate a study procedure that does not require documentation in the individual eCRF.
[1]Study activities during grey-coloured visits are only applicable for subjects in the 'Immuno and reacto' and/or the 'Carriage' subsets (see Table 6 and Table 8, respectively)
[2]Concomitant vaccines: in addition to the blinded study vaccines, subjects will be vaccinated with *(1)* MMR at the age of 12-15 months (according to local EPI), and *(2)* with HAV vaccine at the age of 12-15 months (visit 5) and at 18-21 months of age (since subjects in the control group receive a dose of HAV vaccine at visit 6, the last HAV dose should be administered at least 28 days after the stuy vaccine booster dose) (study benefit= vaccination against HAV at 18-21 months of age in Argentina and at 12-15 and 18-21 months of age in Panama and Colombia). *(3)* In Argentina all subjects will be vaccinated at 12-15 months of age against *Neisseria meningitidis* group C and in Colombia and Panama all subjects will be vaccinated against varicella at 12-15 months of age (study benefit= vaccination against Neisseria in Argentina and against varicella in Colombia and Panama). (4) In Colombia all subjects will be vaccinated against HRV at visit 2 and visit 3 (study benefit= vaccination against HRV).
[3]Only applicable for subjects enrolled in Panama
[4]Applicable for subjects enrolled in Argentina, Colombia and Panama

It is the investigator's responsibility to ensure that the intervals between visits/contacts are strictly followed. These intervals determine each subject's evaluability in the according to protocol analyses (see Sections 9 and 9.4 for details of criteria for evaluability and cohorts to be analyzed).

Table 6 Intervals between 'standard' study visits for all subjects

Interval	Size of interval
Visit 1→Visit 2	49-83 days
Visit 2→Visit 3	49-83 days
Visit 5	12-15 months of age
Visit 5→Visit 6	≥ 28 days
Visit 6	15-18 months of age
Visit 6→Visit 8	≥ 28 days
Visit 8	18-21 months of age
Visit 9	24-27 months of age

Some children will be allocated to one of three subsets (see Table 7, Table 9 and Table 11). The subsets will differ by the number and nature of the study assessments made during the course of the study as described below.

5.4.2. Scheduled visits and study procedures for 'Immuno and reacto' subset

Subjects enrolled in the **'Immuno and reacto' subset** will have the same seven scheduled visits and final study conclusion contact as all subjects plus two additional scheduled visits (see Figure 3 and Table 7). A group of 1,000 subjects (500 respectively in Argentina and Panama) will be part of this subset. A first blood sample will be obtained at visit 4, one month after vaccination 3, while the second and third blood sample will be taken respectively just before and one month after the administration of the booster vaccination (at visits 6 and 7). A fourth blood sample for evaluation of antibody persistence will be taken at 24-27 months of age (last scheduled visit). The subjects from the 'Immuno and reacto' subset will also have to record on diary cards daily solicited symptoms from day 0 to 3 after each of the four study vaccine doses. The subjects belonging to this subset will be subjects enrolled at specific sites (predetermined health care facilities) in each country. These sites will be selected before starting the clinical trial.

Figure 3 Study design of the 'Immuno and reacto' subset

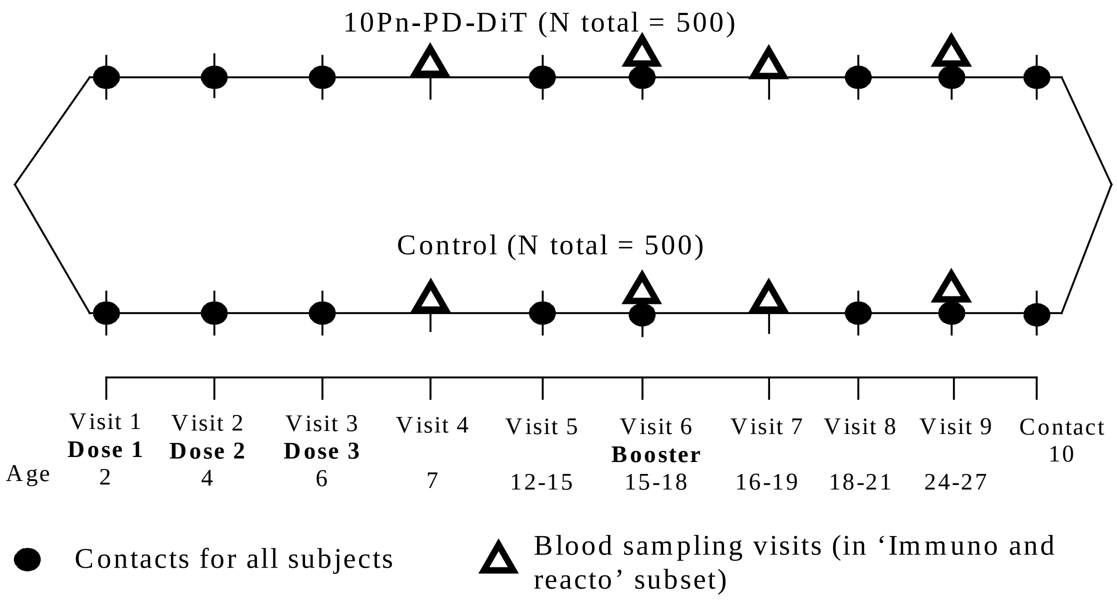

Note: although age windows for different visits sometimes overlap, study procedures for different visits can not be grouped at one visit, and intervals between visits as defined in Table 8 must be respected.

Table 7 List of study procedures for subjects participating in the 'Immuno and reacto' subset of the study

Study visit<br>Age of subject<br>Study timing<br>Blood sampling timepoints	Visit 1<br>6-16 weeks<br>Month 0	Visit 2<br>± 4 months<br>Month 2	Visit 3<br>± 6 months<br>Month 4	Visit 4<br>± 7 months<br>Month 5<br>Post III (M5)	Visit 5<br>12-15 months<br>Month 10-13	Visit 6<br>15-18 months<br>Month 13-16<br>Post III (M13)	Visit 7<br>16-19 months<br>Month 14-17<br>Post IV (M14)	Visit 8<br>18-21 months<br>Month 16-19	Visit 9<br>24-27 months<br>Month 22-25<br>Post IV (M22)	Contact 10<br>Study end
Informed consent	●									
Check inclusion criteria	●									
Check exclusion criteria	●									
Demography, including gestational age	●									
Medical history	●									
Pre-vaccination history	●									
Check elimination criteria		●	●	●	●	●	●	●	●	
Check exclusion criteria for further study participation		O	O	O	O	O	O	O	O	
Check risk factors	●				●				●	
Check warnings, precautions and contraindications	O	O	O		O	O		O		
Physical examination	●	●	●	●	●	●	●	●	●	
Weight/height recording	●	●	●			●			●	
Pre-vaccination body temperature	●	●	●			●				
Randomization	●									
Blood sampling: for antibody determination (3.5 ml)				●		●	●		●	
Vaccination (study and co-administered vaccines)	●	●	●			●				
Vaccination (concomitant vaccines)[1]					●			●		
Return of diary cards		O	O	O			O			
Diary card transcription		●	●	●			●			
Daily post-vaccination recording of solicited symptoms (Days 0-3) by subjects' parents/guardians	●	●	●			●				

Study visit<br>Age of subject<br>Study timing<br>Blood sampling timepoints	VISIT 1<br>6-16 weeks<br>Month 0	VISIT 2<br>± 4 months<br>Month 2	VISIT 3<br>± 6 months<br>Month 4	VISIT 4<br>± 7 months<br>Month 5<br>Post III (M5)	VISIT 5<br>12-15 months<br>Month 10-13	VISIT 6<br>15-18 months<br>Month 13-16<br>Post III (M13)	VISIT 7<br>16-19 months<br>Month 14-17<br>Post IV (M14)	VISIT 8<br>18-21 months<br>Month 16-19	VISIT 9<br>24-27 months<br>Month 22-25<br>Post IV (M22)	CONTACT 10<br>Study end
Record any concomitant vaccination	●	●	●	●	●	●	●	●	●	
Reporting of unsolicited Adverse Events[2]		●	●	●	●	●	●	●	●	
Reporting of convulsions								●		
Reporting of Serious Adverse Events		●	●	●	●	●	●	●	●	●
Study Conclusion										●

Note:
● is used to indicate a study procedure that requires documentation in the individual CRF (eCRF).
O is used to indicate a study procedure that does not require documentation in the individual eCRF.
[1]Concomitant vaccines: in addition to the blinded study vaccines, subjects will be vaccinated with (1) MMR at the age of 12-15 months (according to local EPI), and (2) with HAV vaccine at the age of 12-15 months (visit 5) and at 18-21 months of age (since subjects in the control group receive a dose of HAV vaccine at visit 6, the last HAV dose should be administered at least 28 days after the stuy vaccine booster dose) (study benefit= vaccination against HAV at 18-21 months of age in Argentina and at 12-15 and 18-21 months of age in Panama). (3) In Argentina all subjects will be vaccinated at 12-15 months of age against Neisseria meningitidis group C and in Panama all subjects will be vaccinated against varicella at 12-15 months of age (study benefit= vaccination against Neisseria in Argentina and against varicella in Panama).
[2]Only applicable for subjects enrolled in Panama

It is the investigator's responsibility to ensure that the intervals between visits/contacts are strictly followed. These intervals determine each subject's evaluability in the according to protocol analyses (see Sections 9 and 9.4 for details of criteria for evaluability and cohorts to be analyzed).

Table 8 Intervals between study visits for the 'Immuno and reacto' subset

Interval	Size of interval
Visit 1→Visit 2	49-83 days
Visit 2→Visit 3	49-83 days
Visit 3→Visit 4	28-42 days
Visit 5	12-15 months of age
Visit 5→Visit 6	≥ 28 days
Visit 6	15-18 months of age
Visit 6→Visit 7	28-42 days
Visit 7	16-19 months of age
Visit 7→Visit 8	≥ 28 days
Visit 8	18-21 months of age
Visit 9	24-27 months of age

5.4.3. Scheduled visits and study procedures for ‘Additional immuno’ subset

Subjects enrolled in the ‘Additional immuno’ subset will have the same seven scheduled visits and final study conclusion contact as all subjects plus one additional scheduled visit (see Figure 4 and Table 9). A group of 3,500 subjects in Panama will be part of this subset. A first blood sample will be obtained at Visit 6, just before the booster injection, while the second blood sample will be taken one month after the administration of the booster vaccination (at visit 7)**. (Amended 09 September2010)**

Figure 4 Study design of the ‘Additional immuno’ subset

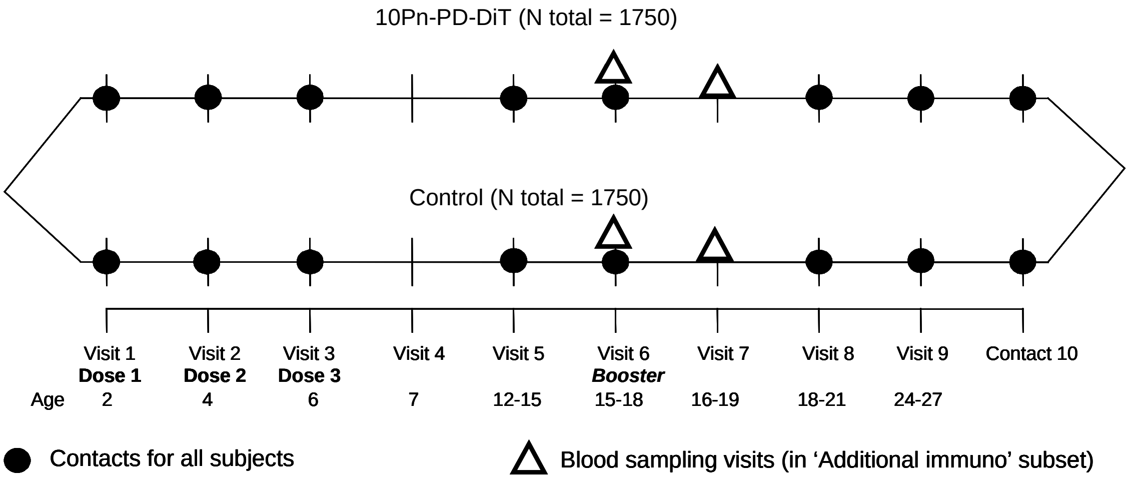

Note: although age windows for different visits sometimes overlap, study procedures for different visits can not be grouped at one visit, and intervals between visits as defined in Table 10 must be respected.

Table 9 List of study procedures for subjects participating in the 'Additional immuno' subset of the study

Study visit<br>Age of subject<br>Study timing<br>Blood sampling timepoints	VISIT 1<br>6-16 weeks<br>Month 0	VISIT 2<br>± 4 months<br>Month 2	VISIT 3<br>± 6 months<br>Month 4	VISIT 4<br>± 7 months<br>Month 5<br>Post III (M5)	VISIT 5<br>12-15 months<br>Month 10-13	VISIT 6<br>15-18 months<br>Month 13-16<br>Post III (M13)	VISIT 7<br>16-19 months<br>Month 14-17<br>Post IV (M14)	VISIT 8<br>18-21 months<br>Month 16-19	VISIT 9<br>24-27 months<br>Month 22-25<br>Post IV (M22)	CONTACT 10<br>Study end
Informed consent	●									
Check inclusion criteria	●									
Check exclusion criteria	●									
Demography, including gestational age	●									
Medical history	●									
Pre-vaccination history	●									
Check elimination criteria		●	●		●	●	●	●	●	
Check exclusion criteria for further study participation		O	O		O	O	O	O	O	
Check risk factors	●				●				●	
Check warnings, precautions and contraindications	O	O	O		O	O		O		
Physical examination	●	●	●		●	●	●	●	●	
Weight/height recording	●	●	●			●			●	
Pre-vaccination body temperature	●	●	●			●				
Randomization	●									
Blood sampling: for antibody determination (3.5 ml)						●	●			
Vaccination (study and co-administered vaccines)	●	●	●			●				
Vaccination (concomitant vaccines)[1]					●			●		

Study visit<br><br>Age of subject<br><br>Study timing<br><br>Blood sampling timepoints	VISIT 1<br><br>6-16 weeks<br>Month 0	VISIT 2<br><br>± 4 months<br>Month 2	VISIT 3<br><br>± 6 months<br>Month 4	VISIT 4<br><br>± 7 months<br>Month 5<br>Post III (M5)	VISIT 5<br><br>12-15 months<br>Month 10-13	VISIT 6<br><br>15-18 months<br>Month 13-16<br>Post III (M13)	VISIT 7<br><br>16-19 months<br>Month 14-17<br>Post IV (M14)	VISIT 8<br><br>18-21 months<br>Month 16-19	VISIT 9<br><br>24-27 months<br>Month 22-25<br>Post IV (M22)	CONTACT 10<br><br>Study end
Record any concomitant vaccination	●	●	●		●	●	●	●	●	
Reporting of unsolicited Adverse Events		●	●		●	●	●	●	●	
Reporting of convulsions[2]								●		
Reporting of Serious Adverse Events		●	●		●	●	●	●	●	●
Study Conclusion										●

Note:
● is used to indicate a study procedure that requires documentation in the individual CRF (eCRF).
O is used to indicate a study procedure that does not require documentation in the individual eCRF.
[1]Concomitant vaccines: in addition to the blinded study vaccines, subjects will be vaccinated with (1) MMR at the age of 12-15 months (according to local EPI, and (2) with HAV vaccine at the age of 12-15 months (visit 5) and at 18-21 months of age (since subjects in the control group receive a dose of HAV vaccine at visit 6, the last HAV dose should be administered at least 28 days after the stuy vaccine booster dose) (study benefit= vaccination against HAV at 12-15 and 18-21 months of age in Panama). (3) In Panama all subjects will be vaccinated against varicella at 12-15 months of age (study benefit= vaccination against varicella in Panama).

It is the investigator’s responsibility to ensure that the intervals between visits/contacts are strictly followed. These intervals determine each subject’s evaluability in the according to protocol analyses (see Sections 9 and 9.4 for details of criteria for evaluability and cohorts to be analyzed).

Table 10 Intervals between study visits for the ‘Additional immuno’ subset

Interval	Size of interval
Visit 1→Visit 2	49-83 days
Visit 2→Visit 3	49-83 days
Visit 3→Visit 4	28-42 days
Visit 5	12-15 months of age
Visit 5→Visit 6	≥ 28 days
Visit 6	15-18 months of age
Visit 6→Visit 7	28-42 days
Visit 7	16-19 months of age
Visit 7→Visit 8	≥ 28 days
Visit 8	18-21 months of age
Visit 9	24-27 months of age

5.4.4. Scheduled visits and study procedures for 'Carriage' subset

Subjects enrolled in the **'Carriage' subset** will have the same seven scheduled visits and final study conclusion contact as all subjects plus two additional scheduled visits (see Figure 5 and Table 11). These subjects (2,000 children in Panama) will have six nasopharyngeal swabs collected between the end of the primary vaccination series and the end of the study period. The subjects belonging to this subset will be subjects enrolled at selected sites.

Figure 5 Study design of the 'Carriage' subset

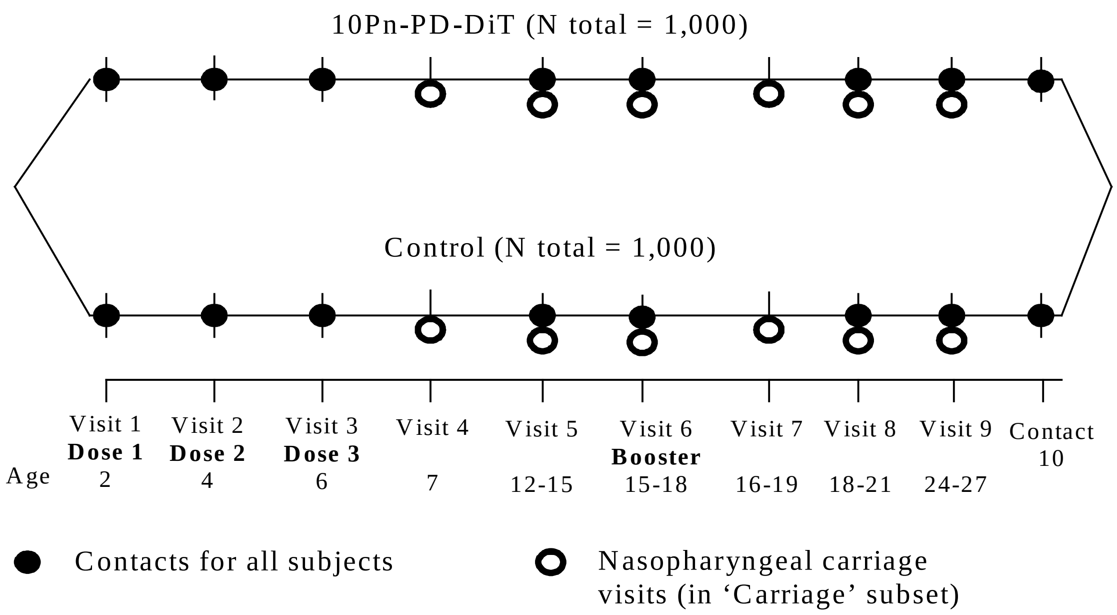

Note: although age windows for different visits sometimes overlap, study procedures for different visits can not be grouped at one visit, and intervals between visits as defined in Table 12 must be respected.

Table 11 List of study procedures for subjects participating in the 'Carriage' subset of the study

Study visit<br>Age of subject<br>Study timing<br>Swab sampling timepoint	VISIT 1<br>6-16 weeks<br>Month 0	VISIT 2<br>± 4 months<br>Month 2	VISIT 3<br>± 6 months<br>Month 4	VISIT 4<br>± 7 months<br>Month 5<br>Post III (M5)	VISIT 5<br>12-15 months<br>Month 10-13<br>Post III (M10)	VISIT 6<br>15-18 months<br>Month 13-16<br>Post III (M13)	VISIT 7<br>16-19 months<br>Month 14-17<br>Post IV (M14)	VISIT 8<br>18-21 months<br>Month 16-19<br>Post IV (M16)	VISIT 9<br>24-27 months<br>Month 22-25<br>Post IV (M22)	CONTACT 10<br>Study end
Informed consent	●									
Check inclusion criteria	●									
Check exclusion criteria	●									
Demography, including gestational age	●									
Medical history	●									
Pre-vaccination history	●									
Check elimination criteria		●	●	●	●	●	●	●	●	
Check exclusion criteria for further study participation		O	O	O	O	O	O	O	O	
Check risk factors	●				●				●	
Check warnings, precautions and contraindications	O	O	O		O	O		O		
Physical examination	●	●	●	●	●	●	●	●	●	
Weight/height recording	●	●	●			●			●	
Pre-vaccination body temperature	●	●	●			●				
Randomization	●									
Nasopharyngeal swab samples				●	●	●	●	●	●	
Vaccination (study and co-administered vaccines)	●	●	●			●				
Vaccination (concomitant vaccines)[1]					●			●		
Record any concomitant vaccination	●	●	●	●	●	●	●	●	●	

Study visit<br>Age of subject<br>Study timing<br>Swab sampling timepoint	VISIT 1<br>6-16 weeks<br>Month 0	VISIT 2<br>± 4 months<br>Month 2	VISIT 3<br>± 6 months<br>Month 4	VISIT 4<br>± 7 months<br>Month 5<br>Post III (M5)	VISIT 5<br>12-15 months<br>Month 10-13<br>Post III (M10)	VISIT 6<br>15-18 months<br>Month 13-16<br>Post III (M13)	VISIT 7<br>16-19 months<br>Month 14-17<br>Post IV (M14)	VISIT 8<br>18-21 months<br>Month 16-19<br>Post IV (M16)	VISIT 9<br>24-27 months<br>Month 22-25<br>Post IV (M22)	CONTACT 10<br><br>Study end
Record any antibiotic treatment (diary card)		●	●	●	●	●	●	●	●	
Reporting of unsolicited Adverse Events		●	●	●	●	●	●	●	●	
Reporting of convulsions								●		
Reporting of Serious Adverse Events		●	●	●	●	●	●	●	●	●
Study Conclusion										●

Note:
● is used to indicate a study procedure that requires documentation in the individual CRF (eCRF).
O is used to indicate a study procedure that does not require documentation in the individual eCRF.
[1]Concomitant vaccines: in addition to the blinded study vaccines, subjects will be vaccinated with (1) MMR at the age of 12-15 months (according to local EPI), and (2) with HAV vaccine at the age of 12-15 months (visit 5) and at 18-21 months of age (since subjects in the control group receive a dose of HAV vaccine at visit 6, the last HAV dose should be administered at least 28 days after the stuy vaccine booster dose) (study benefit= vaccination against HAV at 12-15 and 18-21 months of age in Panama). (3) In Panama all subjects will be vaccinated against varicella at 12-15 months of age (study benefit= vaccination against varicella in Panama).

It is the investigator's responsibility to ensure that the intervals between visits/contacts are strictly followed. These intervals determine each subject's evaluability in the according to protocol analyses (see Sections 9 and 9.4 for details of criteria for evaluability and cohorts to be analyzed).

Table 12 Intervals between study visits for the 'Carriage' subset

Interval	Size of interval
Visit 1→Visit 2	49-83 days
Visit 2→Visit 3	49-83 days
Visit 3→Visit 4	28-42 days
Visit 5	12-15 months of age
Visit 5→Visit 6	≥ 28 days
Visit 6	15-18 months of age
Visit 6→Visit 7	28-42 days
Visit 7	16-19 months of age
Visit 7→Visit 8	≥ 28 days
Visit 8	18-21 months of age
Visit 9	24-27 months of age

5.5. Detailed description of study stages/visits

5.5.1. Scheduled visits

The number of visits and the procedures performed at the different visits will vary according to the subset assignment.

Vaccination will be performed by a person other than the one who performs/records reactogenicity/efficacy assessments (observer-blind) in order to ensure double-blind conditions (see Section 6.6).

5.5.1.1. Scheduled visits to be performed

Visit 1: At 6 – 16 weeks of age (between 42 and 118 days); Study Month 0

Study procedures for ALL SUBJECTS

- Written Informed Consent
- Check inclusion/exclusion criteria
- Assessment of demographic data, including gestational age
- Assessment of medical history
- Assessment of vaccination history (including BCG, HBV and OPV at birth)
- Check warnings, precautions and contraindications to vaccination
- Physical examination
- Recording of weight and height
- Pre-vaccination assessment of body temperature
- Check risk factors
- Randomization and attribution of treatment number
- Vaccination: intramuscular administration of one dose of each of the study and co-administered vaccines according to the guidelines set out in Section 6.3.

  The vaccinees will be observed closely for at least 30 minutes with the appropriate medical treatment readily available in case of a rare anaphylactic reaction following the administration of any study vaccine.
- Record any concomitant vaccination (see Section 6.10)
- The subject's parents/guardians will be instructed to contact the investigator immediately should their child manifest any signs or symptoms they perceive as serious.

Additional study procedures for SUBJECTS IN PANAMA

- A diary card will be distributed to the parents/guardians. They will be instructed to record any adverse event that may have occurred from the day of visit until the next visit in the appropriate sections of the diary card.

  The completed diary card will be given to the investigator at Visit 2.

Additional study procedures for 'IMMUNO AND REACTO' SUBSET

- A diary card will be distributed to the parents/guardians. They will be instructed to record any solicited adverse event that may have occurred on Days 0-3 post-vaccination in the appropriate sections of the diary card.

  The completed diary card will be given to the investigator at Visit 2.

Additional study procedures for 'CARRIAGE' SUBSET

- A diary card will be distributed to the parents/guardians. They will be instructed to record any antibiotic medication taken between the day of visit and the next visit in the appropriate sections of the diary card.

  The completed diary card will be given to the investigator at Visit 2.

**Visit 2: at ±4 months of age; Study Month 2**

Study procedure for ALL SUBJECTS

- Check elimination criteria
- Check exclusion criteria for further study participation
- Check warnings, precautions and contraindications to vaccination
- Physical examination
- Recording of weight and height
- Pre-vaccination assessment of body temperature
- Vaccination: intramuscular administration of one dose of each of the study and co-administered vaccines according to the guidelines set out in Section 6.3.

  The vaccinees will be observed closely for at least 30 minutes with the appropriate medical treatment readily available in case of a rare anaphylactic reaction following the administration of any study vaccine.
- Record any concomitant vaccination (see Section 6.10)
- Record any serious adverse event that may have occurred since previous visit
- The subject's parents/guardians will be instructed to contact the investigator immediately should their child manifest any signs or symptoms they perceive as serious.

Additional study procedures for SUBJECTS IN PANAMA

- Return, verification and transcription of diary card with reporting of any adverse event since last visit.
- A diary card will be distributed to the parents/guardians. They will be instructed to record any adverse event that may have occurred from the day of visit until the next visit in the appropriate section of the diary card.

  The completed diary card will be given to the investigator at Visit 3.

Additional study procedures for ‘IMMUNO AND REACTO’ SUBSET ONLY

- Return, verification and transcription of diary card with reporting of solicited adverse events that occurred on Days 0-3 after the previous study vaccine dose.
- A new diary card will be distributed to the parents/guardians. They will be instructed to record any solicited adverse event that may have occurred on Days 0-3 post-vaccination in the appropriate sections of the diary card.

  The completed diary card will be given to the investigator at Visit 3.

Additional study procedures for ‘CARRIAGE’ SUBSET

- Return, verification and transcription of diary card with reporting of antibiotic medication since last visit.
- A diary card will be distributed to the parents/guardians. They will be instructed to record any antibiotic medication taken between the day of visit and the next visit in the appropriate sections of the diary card.

  The completed diary card will be given to the investigator at Visit 3.

**Visit 3: at ±6 months of age; Study Month 4**

Study procedures for ALL SUBJECTS

- Check elimination criteria
- Check exclusion criteria for further study participation
- Check warnings, precautions and contraindications to vaccination
- Physical examination
- Recording of weight and height
- Pre-vaccination assessment of body temperature
- Vaccination: intramuscular administration of one dose of each of the study and co-administered vaccines according to the guidelines set out in Section 6.3.

  The vaccinees will be observed closely for at least 30 minutes with the appropriate medical treatment readily available in case of a rare anaphylactic reaction following the administration of any study vaccine.
- Record any concomitant vaccination (see Section 6.10)
- Record any serious adverse event that may have occurred since previous visit

- The subject's parents/guardians will be instructed to contact the investigator immediately should their child manifest any signs or symptoms they perceive as serious.

Additional study procedures for SUBJECTS IN PANAMA

- Return, verification and transcription of diary card with reporting of any adverse event since last visit
- A diary card will be distributed to the parents/guardians. They will be instructed to record any adverse event that may have occurred from the day of visit until the next visit in the appropriate section of the diary card.

  The completed diary card will be given to the investigator at Visit 4 if the subject is also part of the 'Immuno and reacto' or 'Carriage' subsets in Panama and at Visit 5 otherwise.

Additional study procedures for 'IMMUNO AND REACTO' SUBSET

- Return, verification and transcription of diary card with reporting of solicited adverse events that occurred on Days 0-3 after the previous study vaccine dose
- A new diary card will be distributed to the parents/guardians. They will be instructed to record any solicited adverse event that may have occurred on Days 0-3 post-vaccination in the appropriate sections of the diary card.

  The completed diary card will be given to the investigator at Visit 4.

Additional study procedures for 'CARRIAGE' SUBSET

- Return, verification and transcription of diary card with reporting of antibiotic medication since last visit
- A diary card will be distributed to the parents/guardians. They will be instructed to record any antibiotic medication taken between the day of visit and the next visit in the appropriate sections of the diary card.

  The completed diary card will be given to the investigator at Visit 4.

Visit 4: at 7 months of age; Study Month 5
'IMMUNO AND REACTO' or 'CARRIAGE' SUBSETS ONLY

Study procedures for 'IMMUNO AND REACTO' SUBSET

- Check elimination criteria
- Check exclusion criteria for further study participation
- Physical examination
- Collection of a blood sample for serology: at least 3.5 ml of whole blood. The sample will be centrifuged and the serum collected (see Section 5.6). The serum will be stored at -20°c before transfer to GSK Biologicals for the laboratory assays (see Section 5.7).

  When materials are provided by GSK Biologicals, it is **MANDATORY** that all clinical samples (including serum samples) will be collected and stored using exclusively those materials in the appropriate manner. The use of other materials could result in

the exclusion of the subject from the ATP analysis (See Section 9.4 for definition of study cohorts to be evaluated). The investigator must ensure that his/her personnel and the laboratory(ies) under his/her supervision comply with this requirement. However, when GSK Biologicals does not provide material for collecting and storing clinical samples, then appropriate materials from the investigator's site are to be used. Refer to Appendix C and Appendix D.

- Record any concomitant vaccination (see Section 6.10)
- Record any serious adverse event that may have occurred since previous visit
- The subject's parents/guardians will be instructed to contact the investigator immediately should their child manifest any signs or symptoms they perceive as serious.
- Return, verification and transcription of diary card with reporting of solicited adverse events that occurred on Days 0-3 after the previous study vaccine dose

Study procedures for 'CARRIAGE' SUBSET

- Check elimination criteria
- Check exclusion criteria for further study participation
- Physical examination
- Return, verification and transcription of diary card with reporting of antibiotic medication since last visit
- A diary card will be distributed to the parents/guardians. They will be instructed to record any antibiotic medication taken between the day of visit and the next visit in the appropriate sections of the diary card.

The completed diary card will be given to the investigator at Visit 5.

- Collect a nasopharyngeal swab as described in Section 5.6.2.
- Record any concomitant vaccination (see Section 6.10)
- Record any serious adverse event that may have occurred since previous visit
- The subject's parents/guardians will be instructed to contact the investigator immediately should their child manifest any signs or symptoms they perceive as serious.

Visit 5: at 12-15 months of age; Study Month 10-13

Study procedures for ALL SUBJECTS

- Check elimination criteria
- Check exclusion criteria for further study participation
- Check warnings, precautions and contraindications to vaccination
- Physical examination
- Check risk factors
- Concomitant vaccination: intramuscular administration of one dose of non-study HAV vaccine (*Havrix*), and intramuscular (*Neisvac-C*) or subcutaneous (*Varilrix* and MMR) administration of one dose of concomitant vaccines as set out in Section 6.11.

  The vaccinees will be observed closely for at least 30 minutes with the appropriate medical treatment readily available in case of a rare anaphylactic reaction following vaccination.
- Record any concomitant vaccination (see Section 6.10)
- Record any serious adverse event that may have occurred since previous visit
- The subject's parents/guardians will be instructed to contact the investigator immediately should their child manifest any signs or symptoms they perceive as serious.

Additional study procedures for SUBJECTS IN PANAMA

- Return, verification and transcription of diary card with reporting of any adverse event since last visit
- A diary card will be distributed to the parents/guardians. They will be instructed to record any adverse event that may have occurred from the day of visit until the next visit in the appropriate section of the diary card.

  The completed diary card will be given to the investigator at Visit 6.

Additional study procedures for 'CARRIAGE' SUBSET

- Return, verification and transcription of diary card with reporting of antibiotic medication since last visit
- A diary card will be distributed to the parents/guardians. They will be instructed to record any antibiotic medication taken between the day of visit and the next visit in the appropriate sections of the diary card.

  The completed diary card will be given to the investigator at Visit 6.
- Collect a nasopharyngeal swab as described in Section 5.6.2.

Visit 6: at 15-18 months of age; Study Month 13-16

Study procedures for ALL SUBJECTS

- Check elimination criteria
- Check exclusion criteria for further study participation
- Check warnings, precautions and contraindications to vaccination
- Physical examination
- Recording of weight and weight
- Pre-vaccination assessment of body temperature
- Vaccination: intramuscular administration of one dose of each of the study and co-administered vaccines according to the guidelines set out in Section 6.3.

  The vaccinees will be observed closely for at least 30 minutes with the appropriate medical treatment readily available in case of a rare anaphylactic reaction following the administration of any study vaccine.
- Record any concomitant vaccination (see Section 6.10)
- Record any serious adverse event that may have occurred since previous visit
- The subject's parents/guardians will be instructed to contact the investigator immediately should their child manifest any signs or symptoms they perceive as serious.

Additional study procedures for SUBJECTS IN PANAMA

- Return, verification and transcription of diary card with reporting of any adverse event since last visit
- A diary card will be distributed to the parents/guardians. They will be instructed to record any adverse event that may have occurred from the day of visit until the next visit in the appropriate section of the diary card.

  The completed diary card will be given to the investigator at Visit 7 if the subject is also part of the 'Immuno and reacto'**,** 'Carriage' and 'Additional immuno' subsets in Panama and at Visit 8 otherwise.

Additional study procedures for 'IMMUNO AND REACTO' SUBSET

- Collection of a blood sample for serology: at least 3.5 ml of whole blood. The sample will be centrifuged and the serum collected (see Section 5.6). The serum will be stored at -20°c before transfer to GSK Biologicals for the laboratory assays (see Section 5.7).

  When materials are provided by GSK Biologicals, it is mandatory that all clinical samples (including serum samples) will be collected and stored using exclusively those materials in the appropriate manner. The use of other materials could result in the exclusion of the subject from the ATP analysis (See Section 9.4 for definition of study cohorts to be evaluated). The investigator must ensure that his/her personnel

and the laboratory(ies) under his/her supervision comply with this requirement. However, when GSK Biologicals does not provide material for collecting and storing clinical samples, then appropriate materials from the investigator's site are to be used. Refer to Appendix C and Appendix D.

- A new diary card will be distributed to the parents/guardians. They will be instructed to record any solicited adverse event that may have occurred on Days 0-3 post-vaccination in the appropriate sections of the diary card:

  The completed diary card will be given to the investigator at Visit 7.

N.B. If parents/guardian observe any large injection site reactions (defined as swelling with a diameter > 50 mm; noticeable diffuse swelling or noticeable increase of limb circumference), they will be asked to contact study personnel and to bring the child as soon as possible to the investigator's office for evaluation (see Section 7.5.1).

**Additional study procedures for SUBJECTS PART OF THE 'ADDITIONAL IMMUNO' SUBSET IN PANAMA**

- Collection of a blood sample for serology: at least 3.5 ml of whole blood. The sample will be centrifuged and the serum collected (see Section 5.6). The serum will be stored at -20°c before transfer to GSK Biologicals for the laboratory assays (see Section 5.7).

  When materials are provided by GSK Biologicals, it is **MANDATORY** that all clinical samples (including serum samples) will be collected and stored using exclusively those materials in the appropriate manner. The use of other materials could result in the exclusion of the subject from the ATP analysis (See Section 9.4 for definition of study cohorts to be evaluated). The investigator must ensure that his/her personnel and the laboratory(ies) under his/her supervision comply with this requirement. However, when GSK Biologicals does not provide material for collecting and storing clinical samples, then appropriate materials from the investigator's site are to be used. Refer to Appendix C and Appendix D**.**

Additional study procedures for 'CARRIAGE' SUBSET

- Return, verification and transcription of diary card with reporting of antibiotic medication since last visit

- A diary card will be distributed to the parents/guardians. They will be instructed to record any antibiotic medication taken between the day of visit and the next visit in the appropriate sections of the diary card.

  The completed diary card will be given to the investigator at Visit 7.

- Collect a nasopharyngeal swab as described in Section 5.6.2.

Visit 7: at 16-19 months of age; Study Month 14-17
'IMMUNO AND REACTO', 'ADDITIONAL IMMUNO' and **'**CARRIAGE' SUBSETS ONLY

Study procedures for 'IMMUNO AND REACTO' SUBSET

- Check elimination criteria
- Check exclusion criteria for further study participation
- Physical examination
- Collection of a blood sample for serology: at least 3.5 ml of whole blood. The sample will be centrifuged and the serum collected (see Section 5.6). The serum will be stored at -20°c before transfer to GSK Biologicals for the laboratory assays (see Section 5.7).

  When materials are provided by GSK Biologicals, it is **MANDATORY** that all clinical samples (including serum samples) will be collected and stored using exclusively those materials in the appropriate manner. The use of other materials could result in the exclusion of the subject from the ATP analysis (See Section 9.4 for definition of study cohorts to be evaluated). The investigator must ensure that his/her personnel and the laboratory(ies) under his/her supervision comply with this requirement. However, when GSK Biologicals does not provide material for collecting and storing clinical samples, then appropriate materials from the investigator's site are to be used. Refer to Appendix C and Appendix D.
- Record any concomitant vaccination (see Section 6.10)
- Record any serious adverse event that may have occurred since previous visit
- The subject's parents/guardians will be instructed to contact the investigator immediately should their child manifest any signs or symptoms they perceive as serious.
- Return, verification and transcription of diary card with reporting of solicited adverse events that occurred on Days 0-3 after the previous study vaccine dose

Study procedures for 'ADDITIONAL IMMUNO' SUBSET

- Check elimination criteria
- Check exclusion criteria for further study participation
- Physical examination
- Collection of a blood sample for serology: at least 3.5 ml of whole blood. The sample will be centrifuged and the serum collected (see Section 5.6). The serum will be stored at -20°c before transfer to GSK Biologicals for the laboratory assays (see Section 5.7).

  When materials are provided by GSK Biologicals, it is **MANDATORY** that all clinical samples (including serum samples) will be collected and stored using exclusively those materials in the appropriate manner. The use of other materials could result in the exclusion of the subject from the ATP analysis (See Section 9.4 for definition of

study cohorts to be evaluated). The investigator must ensure that his/her personnel and the laboratory(ies) under his/her supervision comply with this requirement. However, when GSK Biologicals does not provide material for collecting and storing clinical samples, then appropriate materials from the investigator's site are to be used. Refer to Appendix C and Appendix D.

- Record any concomitant vaccination (see Section 6.10)
- Record any serious adverse event that may have occurred since previous visit.
- The subject's parents/guardians will be instructed to contact the investigator immediately should their child manifest any signs or symptoms they perceive as serious.

Study procedures for 'CARRIAGE' SUBSET

- Check elimination criteria
- Check exclusion criteria for further study participation
- Physical examination
- Return, verification and transcription of diary card with reporting of antibiotic medication since last visit
- A diary card will be distributed to the parents/guardians. They will be instructed to record any antibiotic medication taken between the day of visit and the next visit in the appropriate sections of the diary card.

  The completed diary card will be given to the investigator at Visit 8.
- Record any concomitant vaccination (see Section 6.10)
- Record any serious adverse event that may have occurred since previous visit
- The subject's parents/guardians will be instructed to contact the investigator immediately should their child manifest any signs or symptoms they perceive as serious.
- Collect a nasopharyngeal swab as described in Section 5.6.2.

Additional study procedures for SUBJECTS PART OF THE 'IMMUNO AND REACTO', 'ADDITIONAL IMMUNO' AND 'CARRIAGE' SUBSETS IN PANAMA

- Return, verification and transcription of diary card with reporting of any adverse event since last visit.
- A diary card will be distributed to the parents/guardians. They will be instructed to record any adverse event that may have occurred from the day of visit until the next visit in the appropriate section of the diary card.

  The completed diary card will be given to the investigator at Visit 8.

Visit 8: at 18-21 months of age; Study Month 16-19

Study procedures for ALL SUBJECTS

- Check elimination criteria
- Check exclusion criteria for further study participation
- Check warning, precautions and contraindications to vaccination
- Physical examination
- Record any concomitant vaccination (see Section 6.10)
- Record any serious adverse event that may have occurred since previous visit
- Reporting of convulsions
- The subject's parents/guardians will be instructed to contact the investigator immediately should their child manifest any signs or symptoms they perceive as serious.
- Concomitant vaccination: intramuscular administration of one dose of non-study HAV vaccine (Havrix), as set out in Section 6.3.2.

  The vaccinees will be observed closely for at least 30 minutes with the appropriate medical treatment readily available in case of a rare anaphylactic reaction following vaccination.

Additional study procedures for SUBJECTS IN PANAMA

- Return, verification and transcription of diary card with reporting of any adverse event since last visit.
- A diary card will be distributed to the parents/guardians. They will be instructed to record any adverse event that may have occurred from the day of visit until the next visit in the appropriate section of the diary card.

  The completed diary card will be given to the investigator at Visit 9.

Additional study procedures for 'CARRIAGE' SUBSET

- Return, verification and transcription of diary card with reporting of antibiotic medication since last visit
- A diary card will be distributed to the parents/guardians. They will be instructed to record any antibiotic medication taken between the day of visit and the next visit in the appropriate sections of the diary card.

  The completed diary card will be given to the investigator at Visit 9.
- Collect a nasopharyngeal swab as described in Section 5.6.2.

Visit 9: at 24-27 months of age; Study Month 22-25

Study procedures for ALL SUBJECTS

- Check elimination criteria
- Check exclusion criteria for further study participation
- Check risk factors
- Physical examination
- Recording of weight and height
- Record any concomitant vaccination (see Section 6.10)
- Record any serious adverse event that may have occurred since previous visit
- The subject's parents/guardians will be instructed to contact the investigator immediately should their child manifest any signs or symptoms they perceive as serious.

Additional study procedures for SUBJECTS IN PANAMA

- Return, verification and transcription of diary card with reporting of any adverse event since last visit

Additional study procedures for the 'IMMUNO AND REACTO' SUBSET

- Collection of a blood sample for serology: at least 3.5 ml of whole blood. The sample will be centrifuged and the serum collected (see Section 5.6). The serum will be stored at -20°c before transfer to GSK Biologicals for the laboratory assays (see Section 5.7).

  When materials are provided by GSK Biologicals, it is **MANDATORY** that all clinical samples (including serum samples) will be collected and stored using exclusively those materials in the appropriate manner. The use of other materials could result in the exclusion of the subject from the ATP analysis (See Section 9.4 for definition of study cohorts to be evaluated). The investigator must ensure that his/her personnel and the laboratory(ies) under his/her supervision comply with this requirement. However, when GSK Biologicals does not provide material for collecting and storing clinical samples, then appropriate materials from the investigator's site are to be used. Refer to Appendix C and Appendix D.

Additional study procedures for 'CARRIAGE' SUBSET

- Return, verification and transcription of diary card with reporting of antibiotic medication since last visit
- Collect a nasopharyngeal swab as described in Section 5.6.2.

Contact 10: at the time of study end

Study procedures for ALL SUBJECTS

- Record any serious adverse event that may have occurred since previous visit
- Enter study completion record for the study

5.5.2. Disease surveillance section and unscheduled visits

5.5.2.1. Community Acquired Pneumonia (CAP) surveillance

5.5.2.1.1. Definitions

The clinical suspicion of pneumonia is left to the judgment of the treating physician.

A case of suspected CAP involves either any subject who is referred to have a CXR performed as part of the clinical assessment of a febrile syndrome or an acute respiratory infection (ARI), or a hospitalized child who has a CXR performed within 2 days prior to, or within the first 3 days after hospital admission, as part of the clinical assessment of a febrile syndrome or an ARI.

Interpretation of the CXRs will be done by an independent panel of readers (see Section 5.5.2.1.2 for procedure and qualifications of images) based on the digitalized images and the concepts and definitions mentioned hereunder.

Chest X-ray (CXR) with **consolidation** is defined as a CXR with a dense, often homogeneous, confluent alveolar infiltrate that may encompass an entire lobe or segment, or a fluffy, mass-like, cloud-like density that erases heart and diaphragm borders (silhouette sign) and that often contains air bronchograms.

Pleural effusion is defined as a fluid collecting in the pleural space around the lung, seen radiologically as a dense rim (the same density as the chest-wall muscles) interposed between the lung and the ribs.

Confirmed CAP is defined as a suspected CAP case whose CXR reveals the presence of abnormal pulmonary infiltrates as per the judgement of the independent Panel of readers. These abnormal pulmonary infiltrates can be either with or without alveolar consolidation/pleural effusion.

- **CAP with alveolar consolidation or pleural effusion (C-CAP)** is defined as a subject with confirmed CAP with alveolar consolidation or pleural effusion on CXR.
- **Non-consolidated pneumonia (NC-CAP)** is defined as a subject with confirmed CAP without alveolar consolidation (no alveolar infiltrates on CXR) or pleural effusion.
- **Likely bacterial CAP case (B-CAP) (primary study endpoint)** is defined as a radiologically confirmed CAP case with either alveolar consolidation/pleural effusion on the chest X-ray, or Non-consolidated pneumonia, but with CRP $\geq$ 40 mg/L.
- **No pneumonia**: this diagnosis will be made if the CXR performed for a suspected CAP case reveals no abnormal pulmonary infiltrates.
- **Bacteraemic CAP**: any confirmed CAP case with a positive *S. pneumoniae* or *H. influenzae* blood culture. These cases will be considered for the efficacy

analyses of CAP endpoints. Bacteraemic CAP cases will also be counted as a case of invasive disease.

5.5.2.1.2. Surveillance procedures

The clinical suspicion of pneumonia is left to the judgment of the treating physician. According to the WHO case definition, pneumonia should typically be suspected if the following symptoms are present [WHO, 1991; WHO, 1994]:

For children under 2 months of age

- Fast breathing defined as a respiratory rate of 60 per minute or above confirmed by a second reading after a period of at least 10 minutes
- Marked chest indrawing
- Cyanosis
- Non-specific signs including:
  - the infant stops feeding well
  - the infant is abnormally sleepy or difficult to wake
  - fever or hypothermia (body temperature <35.5°C)
  - convulsions

For children aged 2 months to 4 years of age

- Fast breathing with cough or difficult breathing without wheezing. Fast breathing is defined as:
  - a respiratory rate of 50 per minute or above in children aged 2-11 months
  - a respiratory rate of 40 per minute or above in children aged 1-4 years
  - Respiratory rates are to be recorded when the child is calm and not crying.
- Chest indrawing excluding intercostals or supraclavicular retraction with cough or difficult breathing without wheezing
- Cyanosis
- Not able to drink

For the purpose of the study, suspected pneumonia cases (see definition in Section 5.5.2.1.1) will be captured by qualified study personnel either at the physician's office, the emergency room or the X-ray department. Clinical management of suspected CAP cases will be done according to the local routine practices, including prescription of local laboratory tests and chest X-ray (CXR). Wheezing diseases (such as bronchiolitis or hyperreactive bronchial syndromes) should not follow the study procedures, unless a suspicion of pneumonia is diagnosed, based on the suspected CAP case definition (refer to Section 5.5.2.1.1). Reading of the CXR for clinical diagnostic and treatment purposes will be done according to the routine practice by the local radiologist, physician and/or paediatrician. The reporting of the event, either as an unsolicited AE or as a SAE, whatever applicable (depending on the clinical signs and symptoms and the subset in which the subject is enrolled) will be done according to the treating physician's

judgement and will not be interfered by the study specific procedures for confirmation of the pneumonia diagnosis as described below.

For guidelines for radiology, refer to Appendix F.

Digital CXR images from a suspected CAP cases in study participants will be sent to the Central Reading Panel for further classification (refer to X Ray Workbook). The reading of the image will be classified as (1) consolidated CAP, (2) non-consolidated CAP, (3) no pneumonia or (4) uninterpretable CXR. For more details about the definitions of radiographic outcomes, please refer to Appendix F. All suspected CAP cases occurring in subjects participating in the study will be reported in the eCRF, regardless of the confirmation of the diagnosis by the local radiologist, the treating physician/pediatrician **or the Central X-ray Reading Panel of the study**.

For all CAP cases, the following investigations will be performed:

- It is recommended to perform blood culture for all CAP cases with consolidation according to the judgement of the treating physician, and according to the guidelines provided in the invasive disease surveillance section (see Section 5.5.2.3.3).
- A serum sample for CRP and procalcitonin (PCT) determination will be collected from all suspected CAP cases at the time or at the latest within a maximum of 12 hours after ***a*** chest X-ray was taken or within 12 hours after hospitalization and preferably before the first administration of any antibiotic. Samples will be analyzed later at the Central Lab to measure CRP and PCT levels.
- In addition, whenever possible and within 5 days after initiation of symptoms, efforts will be made to collect a nasopharyngeal aspirate sample in any suspected CAP case. Samples will be shipped to the Central Lab to analyse the presence of specific respiratory viruses: adenovirus, RSV, Flu A & B, and para-influenza 1, 2 and 3.
- The WBC count and formula and the CRP value of the local lab analysis will be entered in the CAP section of the eCRF if available.

Please refer to country-specific LPPs for more details.

5.5.2.1.3. Sample handling at local laboratories

Serum samples

Blood sample will be obtained from all suspected CAP cases. The serum sample will be split into two aliquots: one aliquot for local CRP determination (according to local practices) and a second aliquot to be kept at –20°C or at -70°C until shipment to the Central Lab for quantitative CRP and PCT determination.

Nasopharyngeal aspirate

Whenever possible and within 5 days after initiation of symptoms, nasopharyngeal aspirate samples will be obtained from all suspected CAP cases.

Ideally, a nasopharyngeal aspirate samples should be stored at -70°C as soon as possible after collection because a loss of infectivity occurs over time, resulting in the diminished likeliehood of a positive result. However, if immediate delivery to the laboratory is not

possible, specimens will be stored in a refrigerator (2-8°C) or placed on wet ice or a cold pack for a maximum of 6 hours. Loss of viability is slower at refrigerating temperatures than at ambient temperatures; samples containing labile viruses (e.g. respiratory syncytial virus (RSV) at low titers are those most likely to show loss of infectivity with delayed transport.

The sample will be split into two aliquots and stored at -70°C: one to be prepared for further shipment to the Central Lab, and a second one for future vaccine related testing (refer to Investigator Manual Part I - Biochemistry and Virology for further details on sample collection, handling and storage). Freezing at higher temperatures and freeze-thaw cycles must be avoided [Hoberman et al, 1997; Isenberg, 1998].

5.5.2.1.4. Assessment of the severity of pneumonia

The medical records of each suspected CAP case will be inspected by study personnel to identify the following clinical parameters. The occurrence of any of these parameters during the course of the CAP episode will be reported in the eCRF.

- Tachypnea: defined as a respiratory rate, in absence of crying or fever, of:
  - >60 bpm (0-2 months of age)
  - >50 bpm (2-11 months of age)
  - >40 bpm (1–4 years)
- Chest indrawing (lower chest wall drawse when patient breaths)
- Presence of cough
- Oxygen need / support
- Cyanosis (peripherical or central)
- Grunting
- Auscultation findings (crepitations, absence of pulmonary sounds, wheezing)
- Indication of inpatient treatment (Hospitalization need)
- Inability to take food or drinks
- Lethargy
- Irritability

5.5.2.1.5. Data flow

Digital CXR images of all suspected CAP cases will be sent to the Central Reading Panel. Results of the interpretations by the Central Reading Panel will be transferred directly to GSK, and will not be sent back to the study investigators, since only the local reading of the CXR according to local routine practices will guide diagnostic and therapeutic decisions (Refer to COMPAS X Ray Workbook)**.**

5.5.2.2. Acute Otitis Media (AOM) surveillance

5.5.2.2.1. Case definitions

A case of **clinically suspected AOM (S-AOM)** is defined as a child who meets the following AOM criteria, as per the judgment of a primary care or emergency room physician:

- At least one of the following criteria:
  - either the visual appearance of the tympanic membrane (i.e. redness, bulging, loss of light reflex),
  - or the presence of middle ear effusion (as demonstrated by simple or pneumatic otoscopy or by microscopy),

AND

- At least two of the following clinical symptoms, which should have started within the last 5 days:
  - ear pain
  - ear discharge
  - hearing loss
  - fever (rectal temperature ≥ 38°C, or temperature ≥ 37.5°C for any other route)
  - lethargy
  - irritability
  - anorexia
  - vomiting
  - diarrhea

A case of **clinically confirmed AOM (C-AOM)** is defined as a child who meets the above mentioned AOM case definition, as per the judgment of an ENT specialist.

A case of **bacteriologically confirmed AOM (B-AOM)** is defined as a child with C-AOM, whose MEF grew a bacterial pathogen classically recognized as a causative agent of AOM (e.g. *Streptococcus pneumoniae*, *H. influenzae*, *M. catarrhalis*, Group A streptococci, *S. aureus* etc).

Chronic suppurative otitis media is defined by a continuous discharge, over at least 6 weeks, from the middle ear through a perforation of the tympanic membrane.

Recurrent AOM is defined as at least 3 AOM episodes confirmed by an ENT specialist within 6 months or 4 episodes within one year, regardless of the etiology.

5.5.2.2.2. Surveillance procedures

At study entry parents/guardians of subjects enrolled in Panama will be advised to consult the study physician not only if their child is sick or has ear pain, but also in case of spontaneous perforation of the tympanic membrane and in all cases of spontaneous ear discharge. If the study physician suspects an episode of AOM, according to the above-mentioned case definition, he will then refer the child to an ENT specialist who participates in the trial as investigator. In case of suspicion of AOM, study paediatricians and physicians on duty in emergency departments will be asked not to initiate antibiotic treatment before the referral to the ENT specialist.

For surveillance, the following steps will be followed (see also Figure 6):

1. Sick children will be evaluated by the study physician in one of the predetermined centers.
2. If clinical suspicion of AOM is established, relevant clinical data will be collected and the patient will be referred to the Ear, Nose and Throat (ENT) study specialist.
3. If the AOM case is not confirmed by the ENT specialist, patient will be treated accordingly and relevant clinical data will be registered into the CRF. No other study procedures are foreseen.
4. If the specialist clinically confirms the presence of AOM, relevant clinical data will be registered in the CRF. The ENT specialist will assess the severity of the case (refer to Section 5.5.2.2.4) and, based on the results, he/she will decide the following steps. If tympanocentesis will be performed, the ENT specialist will explain the technique, benefits and potential risks in depth. Subsequently, he/she will invite the subject’s parent/guardian to sign the specific ICF authorizing the performance of tympanocentesis.
5. Tympanocentesis will be performed using the Channel Directed Tympanocentesis speculum (See Appendix G for details of the procedure).
6. Middle ear Fluid (MEF) sample obtained through the tympanocentesis will be inoculated in transport medium and sent within 12 hours to a local laboratory for bacteriological testing (culture, isolation and antibiotic sensitivity for therapeutic guidance according to local practice).
7. Treatment will be indicated to the patient, following local standard guidelines.

An AOM episode will be considered to be bacteriologically confirmed if any of the following bacterial pathogens is identified in the MEF sample: *S. pneumoniae, H. influenzae, M. catarrhalis* and *S. pyogenes*. If *Streptococcus pneumoniae* or *H. influenzae* is isolated at local laboratory, additional bacteriological testing (serotyping and antiobiotic resistance) will be performed at the Central Laboratory. Results from the Central Laboratory will not be sent back to the investigator. Details of the lab procedures performed at the Central lab can be found in Appendix H and in the Lab Workbook.

Figure 6 Procedure for follow-up of AOM cases

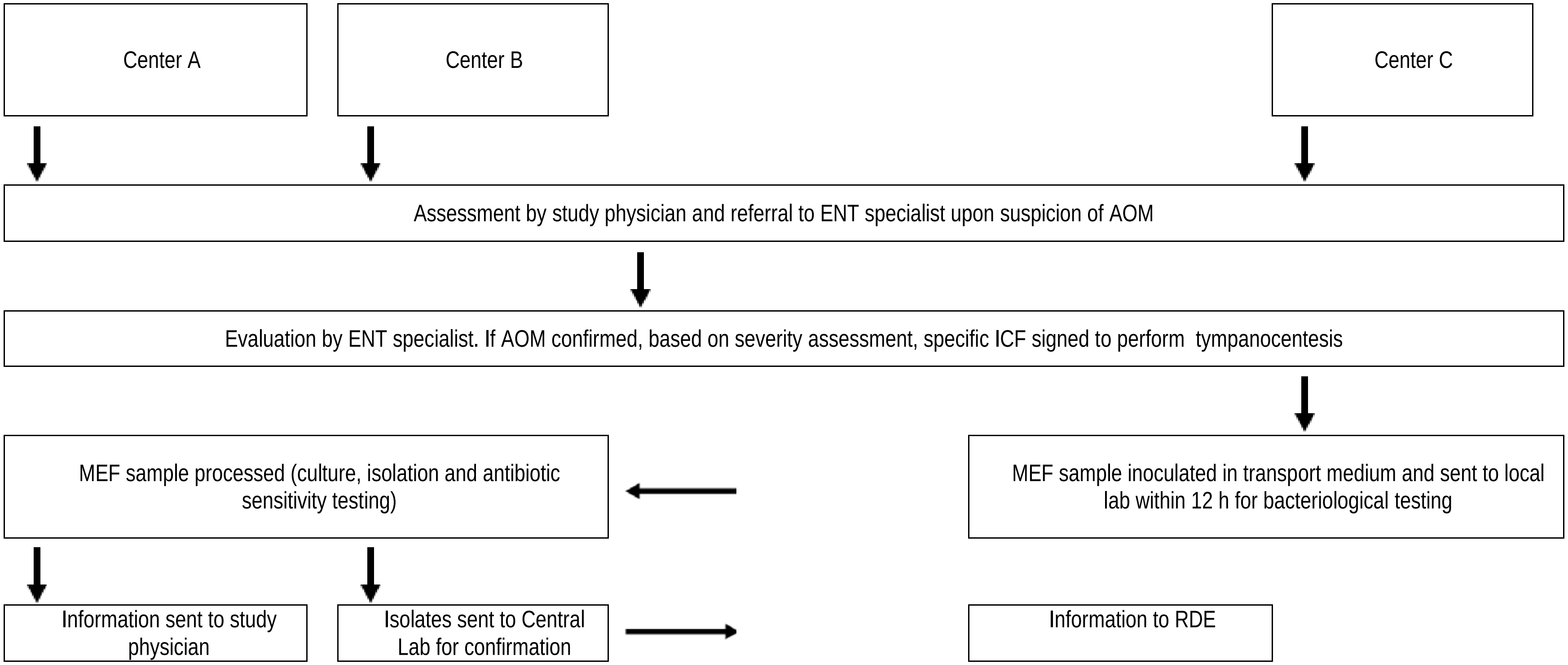

Each unscheduled visit during which acute otitis media is diagnosed will be recorded in the electronic CRF. See Section 5.5.2.2.1 for case definition.

Please refer to country-specific LPPs for more details.

5.5.2.2.3. Sample handling

Middle ear fluid will be collected by tympanocentesis (when tympanic membrane is intact), provided parents/guardians agreed with the performance of tympanocentesis via a specific informed consent form. Tympanocentesis can be easily and safely performed using a Channel Directed Tympanocentesis (CTD) speculum (see Appendix G). In case of spontaneous drainage, MEF will be collected by aspiration using the same CDT device and technique described above**.**

All efforts should be made to optimize diagnosis conditions (i. e. child calm and still, external ear canal thoroughly cleaned, good visibility of the eardrum) and thereby maximise the chances of collecting a sample of MEF.

In case of an intact eardrum, the method is briefly outlined below.

- The use of systemic analgesics before tympanocentesis is left to the discretion of the physician. Because of their anti-microbial activity, local anaesthetics will not be used.
- The use of mask and gloves is highly recommended to avoid contamination of the sample.
- Tympanocentesis is performed under otoscopy through the reusable CTD® Aspirator, attached to the CTD® Speculum.
- The external ear canal must be carefully cleaned with a curet and sterilized with an alcohol-soaked sterile cotton swab.
- After compressing the aspirator bulb, position the speculum in the ear of the subject.
- Determine the point of intended impact in the interior half of the tympanic membrane.
- Perforate membrane by extending the needle.
- Middle ear fluid is drawn gently by releasing pressure on the bulb.

In case of spontaneous/accidental rupture of the tympanic membrane, MEF should be collected within a maximum of 48 hours by aspiration in the ear canal using the CTD® Aspirator, attached to the CTD® Speculum. The ear canal should be thoroughly cleaned before the procedure to avoid masking of NTHi by local flora.

Disposables are provided by GlaxoSmithKline Biologicals.

To obtain best results in the isolation of *S. pneumoniae* and *H. influenzae*, all samples will be inoculated into transport medium (Amies agar gel without charcoal) and sent within 12 hours to the local lab for bacteriological testing [Van Horn et al, 1998].

Identification of colonies of gram-positive cocci (such as *S. pneumoniae*) and gram-negative bacilli (such as *H. influenzae*), as well as antibiotic sensitivity testing will be done at local laboratory [Manual of Clinical Microbiology, 1995]. In those laboratories where additional automated Vytek system is available, differentiation can be made through this system. All this information will be sent to the treating study physician for therapeutic guidance according to local practice.

At the local lab, the most representative colonies will be stored in a cryo-conservation medium (refer to COMPAS Lab Workbook) and kept at -70°C.

Isolates will be kept at these conditions until having confirmation of the date of the shipment to the Central Lab and will be subcultured in blood and chocolate agar 3-5 days prior to the shipment. The most representative bacterial colonies will be selected and transferred to Amies-charcoal transport medium and immediately shipped at room temperature to the Central Lab (refer to Investigator Laboratory Manual - Part II for further details).

5.5.2.2.4. Assessment of severity of AOM

At the primary or study physician's level, the case will be assessed based on the definition of clinically suspected AOM (see Section 5.5.2.2.1). Additionally, severity of each suspected AOM case will be determined according to the following clinical parameters (refer also to Appendix I), and data will be recorded in the specific pages of the eCRF.

- Temperature
- Irritability
- Ear ache
- Tugging
- Feeding
- Sleeping

Based on otoscopy and clinical data previously taken by the primary or study physician, the ENT specialist will determine whether the AOM case is clinically confirmed or not. The following parameters will be reported in the eCRF.

- Side of the affected ear
- Presence of redness of tympanic membrane
- Presence of bulging of tympanic membrane
- Presence of spontaneous perforation
- OS 8 Scoring (Refer to Appendix I)
- Macroscopic aspects of MEF (serous, bloody, purulent, other)
- Additional case information per side (report starting date if spontaneous drainage occurred)

5.5.2.2.5. Data flow

Results of the identification of the MEF isolates with information on antibiotic sensitivity, as performed at the local labs, will be transfered to the local physician. Results of the identification of the MEF isolates will also be transferred to GSK, as well as serotyping and antibiotic sensitivity testing performed at the Central Laboratory. In order to preserve the blinding of the study, the information from the Central Laboratory will ony be made available to the study investigators after the end of the study

5.5.2.3. Invasive disease surveillance

5.5.2.3.1. Case definitions

For study purposes, invasive disease is defined as any disease where *S. pneumoniae* or *H. influenzae* are identified in normally sterile body fluids. The invasive disease will be considered:

- as confirmed when *S. pneumoniae* or *H. influenzae* are isolated from a normally sterile body fluid such as blood, cerebrospinal fluid (CSF), pleural effusion, synovial fluid, peritoneal fluid or aspired abscess.
- as probable when no *S. pneumoniae* or *H. influenzae* are isolated from the above mentioned fluids, but when detection of antigens of *S. pneumoniae* and/or *H. influenzae* is positive when using tests such as Latex agglutination or immunochromatographic Binax™ NOW test (only when meningitis is suspected).

The following clinical syndromes of focal invasive disease will be recorded in the eCRF:

- Meningitis case definitions:

  Purulent meningitis:

  – CSF with >10 polymorphonuclears/mm3 or

  – CSF with >100 leukocytes/mm3 or

  – CSF with 10-99 leukocytes/mm3, and either glucose <30 mg/dl or protein > 100 mg/dl

  Do not use protein and glucose from bloody CSF specimens to meet the probable bacterial meningitis case definition if the ratio of red blood cells to white blood cells > 500:1

  Confirmed meningitis due to *S. pneumoniae* or *H. influenzae*:

  – presence of S. pneumoniae or H. influenzae in CSF culture or

  – purulent meningitis with blood culture positive for S. pneumoniae or H. influenzae

  Probable bacterial meningitis due to *S. pneumoniae* or *H. influenzae*:

  – Purulent meningitis with negative result of CSF and blood culture but with antigen testing (Latex or Binax) positive for S. pneumoniae or H. influenzae in CSF.

– Meningitis with blood culture positive for S. pneumoniae or H. influenzae, with negative result of CSFculture but antigen testing (Latex or Binax) positive for S. pneumoniae or H. influenzae in CSF**.**

- Bacteraemic pneumonia: pneumonia confirmed by radiological evidence of any lung infiltrate and the presence of S. pneumoniae or H. influenzae identified by the blood culture.
- Empyema: isolation of S. pneumoniae or H. influenzae from pleural fluid obtained by thoracentesis, or pleural fluid with polymorphonuclear cells and isolation of S. pneumoniae or H. influenzae from blood culture.
- Peritonitis: isolation of S. pneumoniae or H. influenzae from peritoneal fluid obtained by laparocentesis, paracentesis or laparotomy.
- Osteomyelitis: isolation of S. pneumoniae or H. influenzae from bone aspirate or clinical and imagenology compatible with osteomyelitis and isolation of S. pneumoniae or H. influenzae from blood culture.
- Soft tissue infection: positive blood culture for S. pneumoniae or H. influenzae and clinical signs of cellulitis or abscesses or isolation of S. pneumoniae or H. influenzae from abscess aspirated material.
- Septic arthritis: clinical-radiological presentation compatible with acute arthritis and positive blood culture for S. pneumoniae or H. influenzae or isolation of S. pneumoniae or H. influenzae from material obtained by arthocentesis or arthrotomy.
- Pericarditis: isolation of S. pneumoniae or H. influenzae from fluid obtained by pericardiocentesis or pericardiotomy or presence of pleural friction rubs, muted heart sounds or alterations indicative of a pericardial effusion in thoracic radiology, echocardiogram or electrocardiogram and isolation of S. pneumoniae or H. influenzae from blood culture.

Invasive disease without focal infection such as **bacteremia** (positive blood culture for *S. pneumoniae* or *H. influenzae* indicating viable bacteria in blood without an obvious focus of infection) will also be reported. In addition, generalized infections will be reported as:

- Sepsis: defined as the presence of a Systemic Inflammatory Response Syndrome (SIRS) as a result of suspected or proven infection [for definition see Goldstein et al, 2005].
- Severe sepsis: Sepsis plus one of the following: cardiovascular organ dysfunction OR acute respiratory distress syndrome OR two or more other organ dysfunctions.
- Septic shock: Sepsis and cardiovascular organ dysfunction.

5.5.2.3.2. Surveillance procedures

Identification of cases of invasive disease will be based on clinical suspicion followed by a positive culture. The clinical suspicion of invasive disease cases is left to the judgment of the treating physician. Blood culture is recommended in all cases of febrile illness (axillary fever ≥39.0°C or rectal temperature ≥39.5°C) without overt focus of infection or with suspicion of meningitis, pneumonia, empyema, septic arthritis, peritonitis,

osteomyelitis, pericarditis or bacterial infection of soft tissue. In addition, a blood culture should be seriously considered in children with a recent (within 24 hours) history of febrile illness (axillary fever ≥39.0°C or rectal temperature ≥39.5°C) especially if antipyretic treatment could have interfered with the temperature measurement at the time of physical examination of the child. In those countries where dengue is endemic, such as Panama, it is recommended to perform dengue testing, according to local practices.

Cases of invasive disease occurring in study participants will in addition be captured by study personnel in each site through review of the records of the microbiology laboratories to identify study subjects in which *S. pneumoniae* or *H. influenzae* was isolated from blood cultures or other normally sterile body fluids and/or CSF in which testing for *S. pneumoniae* or *H. influenzae* antigens (Binax or Latex) was positive.

Cases of invasive disease occurring in study participants will be further documented in the invasive disease section of the eCRF. For each case, antibiotics taken within 3 days prior to Normally Sterile Body Fluid (NSBF) collection will be recorded. If the subject is hospitalized or if the adverse event is considered to be serious (refer to Section 7.2 for the definition of serious adverse events (SAEs)), the event should also be notified to GSK according to the SAE reporting procedures defined in Section 7.6. Any case of meningitis, regardless of the etiology, should be reported in the SAE Report Form as a serious adverse event and in the meningitis report form and should be recorded in a specific eCRF page. In case of a confirmed meningitis, the corresponding pages in the Invasive Disease section, including the Lumbar Puncture section, should be filled in. In case of a probable meningitis, only the Lumbar Puncture section should be filled in.

The practical organization of the surveillance for invasive diseases caused by *S. pneumonia* or *H. influenzae* in the study subjects will be described in country specific LPPs.

5.5.2.3.3. Sample handling

Blood culture:

Blood sampling for hemoculture should be collected as soon as possible after a spike of fever and before the start of antimicrobial therapy where possible.

If blood samples for other tests are to be taken at the same venepuncture, the blood culture bottles should be inoculated first to avoid contamination. It is preferable to take samples for blood culture separately.

Inspect the blood culture bottles for damage or defect before use. Ensure that the blood culture bottles were stored according to the manufacturers specifications and that the expiry date is not exceeded.

To avoid contamination of the blood culture, it is required to disinfect adequately the site of venipuncture. Cleanse with water and soap if necessary and disinfect with 70% isopropyl or ethyl alcohol. Hereafter, treat the area with iodine-based solution and allow to dry completely.

Disinfect the septum of the blood culture bottle with ethanol, methanol or isopropyl alcohol and allow to dry (the use of iodine-based disinfectants is NOT recommended for some commercial systems as this is said to affect the integrity of the butyl rubber septum).

Withdraw blood from a peripheral vein. Samples should not be taken through an intravenous catheter or other access device unless no other access is available.

After taking a blood sample, blood culture will be performed, using a licensed fully automated continuously monitoring blood culture system. Blood culturing should be started as soon as possible after sampling. In the meanwhile, blood should not be stored in a refrigerator but at room temperature with airconditioning (15-25°C).

Use a single paediatric bottle appropriate for small volumes of blood. The recommended ideal volume for this purpose is 3 mL [Isenberg, 1998]. Note that different manufacturers market different formats of bottles. Do not exceed the manufacturer's recommended maximum volume for each bottle (maximum 4 mL when using BactAlert system; for the BacTec system the bottle has to be filled at least up to the indicated level with a maximum of 4 mL).

Once the incubator detects a positive sample, process the sample immediately. Positive automated cultures (or other types of cultures) will be sub-cultured by plating onto chocolate and blood agar plates and incubated aerobically with 5-10% CO_2 at 35°C for 48 hours. In those laboratories where additional automated system is available, (Vytek), differentiation can be made through this system. If the sample became positive during the night and there is no trained person available, wait until the next shift to process the sample. If the blood culture bottle has been incubated for more than 10 hours after it has been detected to be positive, a dramatically low viable cells count has been described for pneumococci [Casetta et al, 1996]. Therefore remaining sample of each positive blood culture bottle should be divided into 2 cryovials (1.5 mL each) and kept at -70°C for possible further analysis (see COMPAS Lab workbook).

Bacterial isolates will be kept in duplicate at a country level in cryo-conservation medium (refer to COMPAS Lab Workbook) at -70°C. Once having the confirmation of the date of the shipment to the Central Lab, isolates will be subcultered in blood and chocolate agar media and prepared for transportation in Amies Charcoal medium at room temperature (see Investigator Laboratory Manual - Part II). The Central Lab will confirm viability of the strains (log sheet) to the local lab at a country level (refer to COMPAS Lab Workbook). In addition, local requirements for national surveillance programs will be fulfilled. Back-up samples will be destroyed unless local requirements for relevant national surveillance programs define differently.

Other body fluids

Normally sterile body fluids such as cerebrospinal fluid, pleural effusion, synovial fluid, peritoneal fluid or pericardial fluid will be examined by direct smears and cultured in parallel on appropriate chocolate and blood agar plates. It is also recommended that these fluids are inoculated in special broth for automated culture as is used for blood culture. Positive automated cultures (or other types of cultures) will be sub-cultured by plating

onto chocolate and blood agar plates and incubating aerobically with 5-10% CO_2 at 35°C for 48 hours. In those laboratories where additional automated system is available, (Vytek), differentiation can be made through this system. Bacterial isolates will be kept in duplicate in the same way as for blood sample isolates (see previous paragraph).

If possible, a remaining sample of the original body fluids should be kept and stored at -70°C until collection by GSK or a GSK-designated laboratory, even in case the bacterial subculturing resulted negative (see COMPAS Lab Workbook).

In case of a suspicion of meningitis with collection of CSF, in addition to the local lab procedures and the bacteriological culture as described above, performance of Binax Now test will be mandatory. For this purpose GSK will ensure the availability of this test at each local lab and data will be collected in the eCRF.

The Binax NOW® *Streptococcus pneumoniae* Test is a rapid immunochromatographic (ICT) in vitro assay for the detection of *Streptococcus pneumoniae* (*S. pneumoniae*) C-polysaccharide and will be used in this study only on cerebral spinal fluid (CSF) of patients with suspected meningitis. It is intended, in conjunction with culture and other methods, to aid in the diagnosis of pneumococcal meningitis.

Collect CSF according to standard procedures and store at room temperature (15-25°C) for up to 24 hours before testing. Alternatively, properly collected CSF may be refrigerated (2-8°C) or frozen (-20°C) for up to 1 week before testing.

Test Limitations: A negative Binax NOW® test does not exclude infection with *S. pneumoniae*. Therefore, the results of this test as well as culture results, serology or other antigen detection methods should be used in conjunction with clinical findings to make an accurate diagnosis. In addition, the Binax NOW® *Streptococcus pneumoniae* Test has not been evaluated on patients taking antibiotics for more than 24 hours or on patients who have recently completed an antibiotic regimen. The effects of over-the-counter drugs have not been determined on persons with pneumococcal meningitis.

Other laboratory procedures

Isolation and testing

At a local level, in the participating countries, the following laboratory procedures will be performed. Identification of colonies of gram-positive cocci as *S. pneumoniae* will be based on inhibition by optochin and bile solubility [Manual of Clinical Microbiology, 1995]. Identification of colonies of *H. influenzae* will be done by incubation on chocolate agar plates by Factors X and V growth requirements and biochemical tests [Austrian, 1976]. Positive blood cultures must be sampled within 10 hours after being detected as positive.

Local storage of isolates and transportation to Central Laboratory

All *S. pneumoniae* and *H. influenzae* strains isolated in the context of the current study in all participating countries will be kept in cryo-conservation medium (refer to COMPAS Lab Workbook) at -70°C until preparation for transport. For this purpose, strains will be

subcultured on chocolate and blood agar media, the most representative colonies will be selected, transferred to Amies charcoal transport medium and immediately sent at room temperature to the Central Laboratory where the strains will be serotyped according to established procedures [Austrian, 1976].

Serotyping at the Central Lab

For capsular typing of recovered pneumococci, the quellung reaction will be used according to established procedures [Austrian, 1976]. The absence of slide agglutination in the presence of a to f antisera (*Haemophilus influenzae* Agglutinating Sera MUREX ZM 20-25) will be used to identify non-encapsulated (non-typeable) *H. influenzae* strains.

For more details, please refer to Appendix H and the Lab Workbook.

5.5.2.3.4. *Assessment of the severity of invasive disease caused by S. pneumoniae or H. influenzae*

The severity of each case of invasive disease will be reported using the standard sepsis terminology in pediatrics as agreed at the international pediatric consensus conference and published by Goldstein [Goldstein et al, 2005]. The results of the assessment, to be reported in the eCRF, will be expressed in terms of:

1. Absence of systemic inflammatory response syndrome (SIRS)
2. Presence of systemic inflammatory response syndrome (SIRS)

The consensus definition for SIRS includes the presence of at least two of the following four criteria, one of which must be abnormal temperature or leukocyte count [Goldstein et al, 2005]:

1. Core temperature of >38.5°C or <36°C measured by rectal, bladder, oral, or central catheter probe.
2. Tachycardia (see Table 13) in the absence of external stimulus, chronic drugs, or painful stimuli; or otherwise unexplained persistent evaluation over a time period of > 30 minutes. Bradycardia (see Table 13), in the absence of external vagal stimulus, β -blocker drugs, or congenital heart disease; or otherwise unexplained persistent depression over a time period of > 30 minutes.
3. Tachypnea (see Table 13) or mechanical ventilation for an acute process not related to underlying neuromuscular disease or the receipt of general anesthesia.
4. Leukocyte count elevated or depressed for age (not secondary to chemotherapy-induced leucopenia) or >10% immature neutrophils (see Table 13).

Age-specific vital signs and laboratory variables indicative for SIRS are presented in Table 13. Lower values for heart rate and leukocyte count correspond to the 5th percentile and upper values for heart rate, respiration rate, or leukocyte count correspond to the 95th percentile.

Table 13 Age-specific vital signs and laboratory variables indicative for SIRS

Age group	Heart rate, (beats/minute)		Respiratory rate, (breaths/min)	Leukocyte count ($10^3/mm^3$)
	Tachycardia	Bradycardia	Tachypnea	
1 month to 12 months (infant)	>180	<90	>34	>17.5 or <5
>12 months – 60 months	>140	Not applicable	>22	>15.5 or <6

5.5.2.3.5. Data flow

Investigators will receive relevant microbiological information from the local lab according to local routine practices. Results of the identification, serotyping and antibiotic sensitivity testing of the blood culture and other isolates at the Central Lab will be transferred directly to the database of GSK. In order to preserve the blinding of the study, this information will only be made available to the study investigators after the end of the study.

5.5.2.4. Data collection

Remote Data Entry (RDE) will be used as the method for data collection, which means that subject information will be entered into a computer at the investigational site.

5.6. Sample handling and analysis of biological samples during - the scheduled visits

5.6.1. Blood samples taken from the ‘Immuno and reacto’ and the ‘Additional immuno’ subsets

A total of 3.5 ml of blood will be taken per subject at each of the blood sampling visits (in subjects of the ‘Immuno and Reacto’ or ‘Additional immuno’ subsets) for further immunological analysis. After blood centrifugation and serum separation, samples will be stored at -20°C until collection by the sponsor. The aliquots of serum (approximately 2.0 mL) will be sent to GSK Biologicals. See Section 5.7 for the tests to be performed.

5.6.2. Nasopharyngeal swabs taken from the ‘Carriage’ subset

Nasopharyngeal swabs will be collected by study personnel, using the technique outlined hereafter. A paediatric rayon-tipped swab with a flexible aluminium shaft will be used (provided by GlaxoSmithKline Biologicals). The patient’s head should be tipped slightly backward; the individual taking the specimen should place his or her hand behind the child’s neck or on his or her forehead. The swab should be passed directly backwards

without tipping the swab up or down. In a sitting child, the nasal passage runs parallel to the floor not parallel to the bridge of the nose. Force should not be used while inserting the swab through the nares and passing to the nasopharynx. Rotating the swab should help the swab move since the swab should travel smoothly with minimal resistance. If resistance is encountered, the swab should be removed and the other nostril tried. Once in place, the swab must be rotated 180° and left it in place for 5 seconds to saturate the tip before removing it slowly. Often, failure to obtain a good specimen results from the failure to pass the swab into the nasopharynx. To ensure proper collection, the swab should be passed a distance that is almost the distance between the nostril and the lobe of the ear [O'Brien et al, 2003].

GlaxoSmithKline Biologicals will provide disposables.

After taking the swabs, all samples should be transferred to medium and sent to the local bacteriology laboratory. Refer to Investigator Lab manual Part II for details**.** There, samples will be plated and incubated on blood and chocolate agar. If *S. pneumoniae* or *H. influenzae* colonies are identified, these colonies will be inoculated into a cryo-conservation medium (refer to COMPAS Lab Workbook) and kept at -70°C. Hereafter, at least 3-5 days before the date of shipment to the Central Laboratory, the bacterial samples kept in a cryo-conservation medium (refer to COMPAS Lab Workbook) will be subcultured and incubated again on chocolate and blood agar. The most representative colonies will be selected and inoculated into Amies-charcoal transport medium and sent to Central Laboratory for confirmation.

5.6.3. Microbiological assessments

For each subject, evidence and identification of Haemophilus influenzae, Streptococcus pneumoniae and other bacterial pathogens (for example Moraxella catarrhalis, Group A streptococci and Staphylococcus aureus) in nasopharyngeal specimens will be investigated using bacteriological culture and other bacteriological procedures as described in the COMPAS Lab Workbook at the local bacteriological laboratory.

Identified S. pneumoniae and H. influenzae will undergo further testing for identification of serotypes at the Central Laboratory, GSK Biologicals laboratory or other laboratory designated by GSK Biologicals.

H. influenzae isolates will undergo further subtyping for discrimination of Haemophilus haemolyticus species using new molecular biology tools at GSK Biologicals' laboratory or a validated laboratory designated by GSK Biologicals using standardized and qualified/validated procedures.

(Amended 09 September2010)

5.7. Laboratory assays

As serum represents approximately 50% of whole blood, the approximate blood volumes drawn at Visit 4, Visit 6, Visit 7 and at Visit 9 ('Immuno and Reacto' subset) will be 3.5 mL respectively in order to obtain 2 ml of serum.

After blood centrifugation and serum separation, samples will be stored at -20°C until collection by the sponsor. The aliquots of serum (approximately 2.0 mL) will be sent to GSK Biologicals for the tests described in the following paragraphs.

- Pneumococcal serotype specific total IgG antibodies (antibodies to 1, 4, 5, 6B, 7F, 9V, 14, 18C, 19F and 23F) will each be measured by 22F-inhibition ELISA [Concepcion et al, 2001; Henckaerts et al, 2006]. The antibody concentration will be determined by logistic log comparison of the ELISA curves with a standard reference serum 89-SF available from the US Food and Drug Administration (FDA) for which concentration of IgG and IgM to the 10 serotypes are known in µg/mL [Quataert et al, 1995]. The cut-off of the assay is 0.05 µg/mL.
- *S. pneumoniae* opsonophagocytic activity will be measured by a killing-assay using a HL 60 cell line [Romero-Steiner et al, 1997; Henckaerts et al, 2007]. The results will be presented as the dilution of serum (opsonic titre) able to sustain 50 % killing of live pneumococci under the assay conditions. The cut-off of the assay is an opsonic dilution of 8.
- The antibody concentrations (ELISA) and opsonophagocytic activity (OPA) to the pneumococcal cross-reactive serotypes 6A and 19A (cut-off 0.05 µg/mL for ELISA and cut-off 8 for OPA) will also be determined.
- Anti-PD antibodies will be determined using an ELISA assay developed by GSK Biologicals. Concentration of specific PD antibodies will be determined, using a standard reference serum. The cut-off of the assay is 100 EL.U/mL.

Table 14 Laboratory Assays

Antigen	Assay method	Test Kit/ Manufacturer	Assay unit	Assay cut-off	Laboratory[1]
10 pneumococcal vaccine serotypes (1, 4, 5, 6B, 7F, 9V, 14, 18C, 19F and 23F) and 2 pneumococcal cross-reactive serotypes 6A and 19A	ELISA	in-house	µg/mL	0.05	GSK
10 pneumococcal vaccine serotypes (1, 4, 5, 6B, 7F, 9V, 14, 18C, 19F and 23F) and 2 pneumococcal cross-reactive serotypes 6A and 19A	Opsonophagocytic assay	in-house	dilution for 50% killing	8	GSK
protein D	ELISA	in-house	EL.U/mL	100	GSK

[1]Or designated validated laboratory

The GSK Biologicals' laboratory at Rixensart has established Quality Control Procedures and an established Quality System supported by procedures. The activities of the clinical

laboratories are audited regularly for quality assessment by an internal (sponsor-dependent) but laboratory-independent Quality Department.

5.7.1. Exploratory laboratory assays

The measure of anti-protein D (PD) antibodies by the anti-PD IgG ELISA has been shown inappropriate to discriminate healthy individuals to those who have developed AOM disease against NTHi. Different test developments have been initiated to develop a clinically pertinent assay; the most promising strategies currently followed to correlate serological response to protein D with protection are outlined below. It is worth noting that the list is not exhaustive and that other tests aiming to detect anti-PD protective antibodies might be developed. Provided technical qualification, these tests will be evaluated for their ability to differentiate protected from unprotected individuals.

5.7.1.1. Avidity anti-PD ELISA

Avidity of antibodies for their target antigen is recognized as a possible measure of their protective functionality. This functional test is based on the dissociation of low affinity antibody-antigen complexes in presence of a chaotropic agent such as sodium thiocyanate. The results are expressed as an avidity index (AI) which is the percentage of anti-PD antibodies remaining bound to PD after treatment with sodium thiocyanate.

5.7.1.2. Protein D enzymatic inactivation assay

In an infant rat infection model, it has been observed that wild-type NTHi strains induce ciliary lost in epithelial cells; this effect is not observed when a strain deleted for the gene encoding PD is used. The protein D is an enzyme with a glycerophosphoryl diester phosphodiesterase (GLPQ) activity producing choline from glycerophosphorylcholine. Choline is known to interact with PAF receptor (Platelet Activating Factor) and may trigger ciliary effacement through protein G transduction pathway. These observations indicate that the GLPQ enzymatic activity might be critical for bacterial colonization. Hence, antibodies with a specific GLPQ inhibitory effect could be a good indicator of protective immunity. To detect such antibodies, a coupled enzymatic-optical test to assess the GLPQ activity of PD and its possible inhibition by human immune sera assay has been developed.

Briefly Glycerol-3-phosphate (G3P) produced from L-a-Glycerophosphorylcholine by the GLPQ activity is converted into dihydroxyacetone phosphate by a Glycerol-3-phosphate dehydrogenase (GPDH) added to the system. GPDH activity needs NAD+ as co-factor which is reduced into NADH during the enzymatic reaction. The rate of NADH production is followed by measuring the increase in absorbance at 340 nm. Presence of inhibitory antibodies in a given serum is thus assessed by a low level of NADH production. The test has been adapted to a microtiter plate format and the results are expressed as a percentage of inhibition**.**

5.7.2. Immunological read-outs

Table 15 Immunological read-outs

Number of subjects	Group	Blood sampling timepoint			Antigen[2]
		Timing	Months of age	Visit no.	
1,000 subjects in 'Immuno and Reacto' subset	All	Post III (M 5)<br>Post III (M13)<br>Post IV (M14)<br>Post IV (M22)	7<br>15-18<br>16-19<br>24-27	4<br>6<br>7<br>9	-Pneumococcal serotypes 1, 4, 5, 6B, 7F, 9V, 14, 18C, 19F, and 23F, and pneumococcal cross-reactive serotypes 6A and 19A (ELISA)<br>-Pneumococcal serotypes 1, 4, 5, 6B, 7F, 9V, 14, 18C, 19F, and 23F, and pneumococcal cross-reactive serotypes 6A and 19A (OPA)<br>-Protein D

It should be noted that for the biological tests (pneumococcal opsonophagocytic activity [OPA]) sterile aliquots are prepared before initiating ELISA testing.

In case of insufficient blood sample volume to perform all assays for all antibodies as planned, the samples will be aliquoted for biological tests and analyzed according to the following priority ranking:

5. Pneumococcal antibodies against the 10 pneumococal vaccine serotypes and the pneumococcal cross-reactive serotypes 6A and 19A (ELISA)
6. Opsonophagocytic activity against the 10 pneumococcal vaccine serotypes and the pneumococcal cross-reactive serotypes 6A and 19A (OPA)
7. Antibodies against Protein D

Samples will not be labelled with information that directly identifies the subjects but will be coded with the identification number of the subject.

Any additional serology on antigens contained in the study vaccines may be performed if deemed necessary by GSK Biologicals, if any findings in the present study or in other studies necessitate further investigation of the immunogenicity of the study vaccine. In this case, the ranking above may also be changed.

Collected samples may be used for purposes related to the quality assurance of the laboratory tests described in this protocol. This may include the management of the quality of the current tests, the maintenance or improvement of these current tests, the development of new test methods for the markers described in this protocol, as well as making sure that new tests are comparable to previous methods and work reliable.

It may be that any findings in the present or in other studies necessitate further investigation by GSK Biologicals into the efficacy or immunogenicity of the study vaccine and its constituents under study or further research in the diseases under study. Under these circumstances, additional testing on the samples may be performed by GSK Biologicals outside the scope of this protocol.

A GSK Biologicals Research & Development Position Paper is available which describes the rationale for and some examples of what these further investigations might include.

Any sample testing will be done in line with the consent of the individual subject.

Any human pharmacogenetic testing is not planned and would require require additional separate consent from the individual subjects and the ethics committee approval after the appropriate protocol amendment would be approved. Any anti-HIV testing will also require specific consent and ethics committee approval.

Refer also to protocol Appendix B, where it is noted that the Investigator cannot perform any other biological assays except those described in the protocol or its amendment(s).

Collected samples will be stored for up to 15 years (counting from when the last subject performed the last study visit), unless local rules, regulations or guidelines require different timeframes or different procedures, which will then be in line with the subject consent. These extra requirements need to be communicated formally to and discussed and agreed with GSK Biologicals.

5.7.3. Endpoints for suboptimal response

At the time that this protocol was prepared, a pneumococcal correlate of protection had not been clearly established. The endpoints for suboptimal responses and the need for vaccinating children with licensed pneumococcal conjugate vaccines will be re-evaluated in the light of scientific knowledge at study completion.

6. INVESTIGATIONAL PRODUCT AND ADMINISTRATION

6.1. Study vaccines

The candidate 10Pn-PD-DiT vaccine has been developed and manufactured by GSK Biologicals.

The Quality Control Standards and Requirements for the candidate vaccine are described in separate release protocols and the required approvals have been obtained.

The GSK Biologicals' *Engerix-B*, given at 2, 4 and 6 months of age, and *Havrix*, administered at 15-18 months of age, serve as control vaccines, and comply with the specifications given in the manufacturer's Summary of Product Characteristics.

6.2. Co-administered and concomitant vaccines

The GSK Biologicals' DTPa-IPV/Hib, *Infanrix hexa*, Rotarix and *Varilrix* vaccines comply and *Neisvac-C* is assumed to comply with the specifications given in the manufacturer's Summary of Product Characteristics. See Table 16 for details on the study, co-administered and concomitant vaccines.

Table 16 Vaccines administered in the study

Vaccine	Group	Age of administration (months)	Type of product	Blinding
Study vaccines				
10Pn-PD-DiT	10Pn-PD-DiT	2, 4, 6 and 15-18	Randomized (Investigational product)	Blinded
Engerix-B	Control	2, 4 and 6	Randomized (no commercial lot)	Blinded
Havrix	Control	15-18	Randomized (no commercial lot)	Blinded
Co-administered vaccines				
Infanrix hexa	10Pn-PD-DiT	2, 4 and 6	Randomized (no commercial lot)	Blinded
DTPa-IPV/Hib	10Pn-PD-DiT<br>Control	15-18<br>2, 4, 6 and 15-18	Randomized (no commercial lot)	Blinded
Concomitant vaccines*				
Havrix	All subjects	12 and 18-21	Commercial lot	Unblinded
Neisvac-C	All subjects in Argentina	12	Commercial lot	Unblinded
Varicella	All subjects in Panama	12	Commercial lot	Unblinded
Rotarix	All subjects in Colombia	2 doses within the first 6 months of life	Commercial lot	Unblinded

*These vaccines are benefits from the study

Other locally recommended vaccines (such as influenza or rotavirus vaccines, recommended either through the EPI program or through national immunization campaigns) are always allowed, even if concomitantly administered with the study vaccines, but should be documented in the eCRF.

Table 17 Formulation of Vaccines

Vaccine	Formulation (per dose)	Presentation	Vol. (mL)
GSK Biologicals' 10-valent Pn-PD-DIT vaccine	Protein D carrier: 1 μg of each PS for serotypes 1, 5, 6B, 7F, 9V, 14 and 23F and 3μg for serotype 4 conjugated to PD<br>Tetanus toxoid carrier with AH spacer: 3 μg of capsular PS of serotypes 18C conjugated to TT_{AH}<br>Diphtheria toxoid: 3 μg of capsular PS of serotype 19F conjugated to DT<br>Protein carrier content: ~12 μg PD, ~ 4.5 μg DT, ~ 7 μg TT<br>0.5 mg aluminium (Al^{3+}) as aluminium phosphate adjuvant	Whitish liquid in vial or prefilled syringe	0.5
GSK Biologicals' HAV vaccine (*Havrix*)	HAV (strain HM 175) 720 EL.U<br>Aluminium as salt: 0.25 mg	Whitish liquid in vial or prefilled syringe	0.5
GSK Biologicals' HBV vaccine (*Engerix-B*)	10μg of HBsAg, adsorbed on aluminium oxide; 0.25 mg Al^{3+}	Whitish liquid in prefilled syringe	0.5
GSK Biologicals' DTPa-HBV-IPV/Hib vaccine (*Infanrix hexa*)	Diphtheria toxoid ≥ 30IU (25Lf);<br>Tetanus toxoid ≥ 40IU (10Lf);<br>Pertussis antigens: PT 25 μg,<br>FHA 25 μg, PRN 8 μg;<br>Hepatitis B surface antigen (HBsAg): 10 μg;<br>Poliovirus type 1: 40 D antigen units, poliovirus type 2: 8 D antigen units, poliovirus type 3: 32 D antigen units;<br>PRP 10 μg conjugated to 20-40 μg tetanus toxoid<br>Phenoxyethanol ≤ 2.5 mg<br>Aluminium as salts 0.82 mg	DTPa-HBV-IPV component as a whitish liquid in vial or pre-filled syringes<br>+<br>Hib component as a white freeze-dried pellet in a monodose vial. to be reconstituted with DTPa-HBV-IPV vaccine	0.5
GSK Biologicals' DTPa-IPV/Hib vaccine	Diphtheria toxoid ≥ 30IU (25Lf);<br>Tetanus toxoid ≥ 40IU (10Lf);<br>Pertussis antigens: PT 25 μg,<br>FHA 25 μg, PRN 8 μg;<br>Poliovirus type 1: 40 D antigen units, poliovirus type 2: 8 D antigen units, poliovirus type 3: 32 D antigen units;<br>2-phenoxyethanol 2.5 mg<br>PRP 10 μg conjugated to 20-40 μg tetanus toxoid<br>Aluminium as salts 0.5 mg	DTPa-IPV component as a whitish liquid in vial or pre-filled syringes<br>+<br>Hib component as a white freeze-dried pellet in a monodose vial. to be reconstituted with DTPa-IPV vaccine	0.5
MenC vaccine (*Neisvac-C*)	*Neisseria meningitidis* group C (strain C11) polysaccharide (de-O-acetylated) conjugated to 10-20 μg tetanus toxoid adsorbed on 0.5 mg Al^{3+} (aluminium hydroxide)	A semi-opaque white to off-white suspension in vial or prefilled syringe	0.5
GSK Biologicals' varicella (*Varilrix*) vaccine	Live attenuated Oka strain varicella virus (≥ $10^{3.3}$pfu/dose)	Slightly pinkish freeze-dried pellets in vial + pre-filled syringe of diluent	0.5

Vaccine	Formulation (per dose)	Presentation	Vol. (mL)
GSK Biologicals' live oral attenuated HRV (*Rotarix*) vaccine	*Details on Rotarix vaccine formulation were blinded for Intellectual Property (IP) reasons.*	Lyophilized vaccine in monodose glass vial. Diluent *(composition blinded for IP reasons)* supplied separately.<br><br>Liquid buffer in pre-filled syringe	NA<br><br>1.3

Note: MMR vaccine will be administered according to local EPI program.

Refer to Appendix E for details of vaccine supplies.

6.3. Dosage and administration

Table 18 Dosage and Administration

Visit	Vaccination	Dose	Vaccine[1]	Route[2]	Site[3]	Side[4]
1-2-3	10Pn-PD-DiT or *Engerix-B*	1-2-3	Candidate 10Pn-PD-DiT vaccine or Control *Engerix-B* vaccine	IM	T	R
6	10Pn-PD-DiT or *Havrix*	4	Candidate 10Pn-PD-DiT vaccine or Control *Havrix* vaccine	IM	D or T	R
1-2-3	*Infanrix hexa* or DTPa-IPV/Hib	1-2-3	Co-administered vaccine	IM	T	L
6	DTPa-IPV/Hib	4	Co-administered vaccine	IM	D or T	L

[1]Vaccine / Control / Co-administered
[2]Intramuscular (IM)
[3]Thigh (T); Deltoid region of upper arm (D)
[4]Left (L)/ Right (R)

Refer to Section 3.2 for details on the immunization scheme

The vaccinees will be observed closely for at least 30 minutes following the administration of vaccines, with appropriate medical treatment readily available in case of a rare anaphylactic reaction.

6.3.1. Injection technique

In the first year of life, intramuscular injections are administered into the anterolateral region of the thigh (see details below). In the second year of life, IM injections can also be performed in the deltoid muscle. **The buttock should not be used for administration of vaccines** because of the potential risk of injury to the sciatic nerve and the risk of decreased immunogenicity because of inadvertent subcutaneous injection or injection into deep fat tissue.

For all intramuscular injections, the needle should be long enough to reach the muscle mass and prevent vaccine from seeping into subcutaneous tissue, but not so long as to involve underling nerves and blood vessels or bone. Vaccinators should be familiar with

the anatomy of the area into which they are injecting vaccine. Although it is recommended to follow the guidelines provided below, an individual decision on needle size and site of injection must be made for each person on the basis of age, and the size of the muscle.

Injections in the thigh

In the majority of infants (< 12 months of age), a 25 mm (1 inch), 22-25 gauge needle is sufficient to penetrate muscle in the infant's thigh. The following injection technique is recommended [Bergeson et al, 1982]:

The needle should be inserted in the upper lateral quadrant of the thigh, directed inferiorly at an angle of 45 degrees with the long axis of the leg, and posteriorly at a 45-degree angle to the tabletop, with the subject supine. During the injection, the tissues of the injection site are compressed with the free hand, increasing the penetrable muscle mass and stabilizing the extremity.

If injections into the anterolateral thigh are performed in children aged ≥ 12 months of age, the needle should be 25 mm (1 inch).

Injections in the deltoid

In children aged ≥ 12 months, injection in the deltoid can be performed if the muscle size is adequate, using a 25 mm (1 inch), 22-25 gauge needle.

Intramuscular injections should be administered with caution to subjects with thrombocytopenia or a bleeding disorder since bleeding may occur following an intramuscular administration to these subjects. Firm pressure should be applied to the injection site (without rubbing) for at least two minutes.

For all types of vaccine injection, the vaccinees will be observed closely for at least 30 minutes following the administration of vaccines, with appropriate medical treatment readily available in case of a rare anaphylactic reaction.

6.3.2. GlaxoSmithKline Biologicals' 10Pn-PD-DiT conjugate, *Engerix-B* or *Havrix* vaccines

The prefilled syringes containing the liquid pneumococcal conjugate or control vaccines must be shaken before use to obtain a homogeneous whitish suspension. One dose of 0.5 mL of the vaccines will be injected **intramuscularly** into the **right anterolateral thigh or right deltoid for the booster dose** (see Section 6.3.1). Vaccines should not be administered in the buttock or intradermally since this may result in a lower immune response.

6.3.3. GlaxoSmithKline Biologicals' DTPa-HBV-IPV/Hib or DTPa-IPV/Hib vaccine

To prepare GSK Biologicals' co-administered DTPa-HBV-IPV/Hib vaccine (*Infanrix hexa*) or DTPa-IPV/Hib vaccine for administration, the syringe containing the liquid

DTPa-HBV-IPV or DTPa-IPV suspension should be well shaken in order to obtain a homogeneous turbid white suspension.

The DTPa-HBV-IPV or DTPa-IPVsuspension and the Hib powder should be inspected visually for any foreign particulate matter and/or abnormal physical appearance. In the event of either being observed, discard the vaccine.

The vaccine is reconstituted by adding the contents of the syringe to the vial containing the Hib powder. It is good clinical practice to only inject a vaccine when it has reached room temperature. In addition, a vial at room temperature ensures sufficient elasticity of the rubber closure to minimise any coring of rubber particles. To achieve this, the vial should be kept at room temperature (25 ± 3°C) for at least 5 minutes before entering the syringe's needle and reconstituting the vaccine. Mix thoroughly until the Hib powder is completely dissolved. Aspirate the reconstituted vaccine back into the syringe.

The reconstituted vaccine presents as a slightly more cloudy suspension than the liquid component alone. This is normal and does not impair the performance of the vaccine. In the event of other variation being observed, the vaccine should be discarded.

One dose (0.5 mL) of the mixed DTPa-HBV-IPV/Hib or DTPa-IPV/Hib vaccine should be administered by deep **intramuscular** injection into the **left anterolateral thigh or left deltoid for the booster dose** (see Section 6.3.1).

The mixed vaccine should be used immediately after reconstitution. To administer the vaccine, a new needle should be used.

6.4. Storage

All investigational products to be administered to subjects must be stored in a safe and locked place with no access by unauthorized personnel.

Vaccines will be stored at the defined temperature range (i.e. +2 to +8°C).

The storage temperature of vaccines will be monitored daily while using validated temperature monitoring devices and the temperature measurements will be recorded during working days, preferably at the same time of the day (e.g. at the beginning of the day). Freezing indication will be continuously controlled by an appropriate device placed close to the vaccines.

Any temperature deviation, i.e. temperature outside the defined range (i.e. +2 to +8°C), must be reported within 24 hours to the sponsor (i.e. Study Monitor/ GSK Local Contact/ GSK Biologicals).

Following exposure to a temperature deviation, vaccines will not be used until written approval is given by the sponsor.

Storage conditions for transport of vaccines from country medical department or dispatch centre to study sites or between sites are described in Appendix E and according to the local SOP about management of clinical trial supplies.

6.5. Treatment allocation and randomization

The target enrolment for this multicountry and multicenter study will be 24,000 eligible subjects (12,000 in both the 10Pn-PD-DiT group and the control group) in order to achieve 21,600 evaluable subjects (10,800 in both 10Pn-PD-DiT and the control group) in the ATP cohort for efficacy.

6.5.1. Randomization of supplies

A randomization list will be generated at GSK Biologicals, Rixensart, using a standard SAS® (Statistical Analysis System) program and will be used to number the vaccines (see Section 3.2). A randomization blocking scheme (1:1 ratio) will be used to ensure that balance between treatments is maintained: a treatment number will identify uniquely the vaccine doses to be administered to the same subject.

To allow GSK Biologicals to take advantage of greater rates of recruitment than anticipated at individual centres in this study, and to thus reduce the overall study recruitment period, an over-randomization of supplies will be prepared.

The vaccine doses will be distributed to each study centre, respecting the randomization block size.

6.5.2. Randomization of subjects

The treatment allocation at the investigator site will be performed using a central randomization system on Internet (SBIR). The randomization algorithm will use a minimization procedure accounting for centre.

The person in charge of the vaccination or other study personnel will access the randomization system on Internet. Upon providing a subject number for the subject, the randomization system will use the minimization algorithm to determine the treatment number to be used for the subject.

The actual treatment number used for first vaccination of the subject must be recorded by the investigator in the eCRF (Rando/Treatment Allocation Section).

6.6. Method of blinding and breaking the study blind

This study will be conducted in a double-blind/observer-blind fashion. Although the 10Pn-PD-DiT, the HBV and the HAV vaccines are whitish liquid in prefilled syringes, *Havrix* and *Engerix-B* have a slightly different visual appearance than 10Pn-PD-DiT. Therefore, in this trial, an observer-blind procedure will be followed to ensure the double-blinding of the study: the person responsible for the preparation and administration of the vaccine will not be involved in the efficacy or safety/reactogenicity evaluation of the vaccine.

In order to keep the blinding, any analysis conducted before all study data are available and cleaned will be performed by an independent statistician (see section 9.7). In addition ~~The serological and efficacy~~ data, which might lead to the unblinding of the treatment groups, will not be available during the course of the trial to any investigator or any person involved in the clinical conduct of the study (including data cleaning). **(Amended 09 September2010)**

The investigator, or person designated by the investigator, should contact GSK Biologicals' Central Safety physician directly or via the local safety contact (see below and Study Contact for Emergency Code Break in Sponsor Information page) to discuss the need for emergency unblinding. No set of individual codes (code break envelopes) will be held at the local GSK Biologicals Safety Office or GSK Biologicals' Central Safety Office. The GSK Biologicals' Central Safety Office will be allowed to access the individual randomization code through SBIR. The code will be broken by the Central Safety physician (see below and Study Contact for Emergency Code Break in Sponsor Information page) only in the case of medical events that the investigator/physician in charge of the subject feels cannot be treated without knowing the identity of the study vaccine(s).

**GSK Biologicals Central Safety Physician<br>(Study Contact for Emergency Code Break)**
Phones for 7/7 day availability
Tel: ~~+32 10 85 48 43~~ ***+32 10 85 64 00***<br>~~Fax: +32 2 656 51 16 or +32 2 656 80 09~~<br>~~Mobile phones for 7/7 day availability:~~<br>~~+32 472 906 600~~<br>Back-up ~~mobile~~ phone contact:<br>~~+32 474 53 48 68~~ ***+32 10 85 6401***

(Amended 09 September2010)

6.7. Replacement of unusable vaccine doses

Additional vaccine doses will be provided to replace those that are unusable (see Appendix E for details of supplies).

In addition to the vaccine doses provided for the planned number of subjects (including over-randomization when applicable), an appropriate amount of numbered additional

doses will be supplied. In case a vaccine dose is broken or unusable, the investigator should replace it with a numbered replacement vaccine dose. Although the sponsor need not be notified immediately in these cases (except in the case of cold-chain failure), documentation of the use of the replacement vaccine must be recorded by the investigator on the vaccine administration page of the eCRF and on the vaccine accountability form.

The investigator should use the central randomization system (SBIR) to obtain the replacement vial/syringe number. The system will ensure, that the replacement vial/syringe is of the same formulation as the randomized vaccine.

6.8. Packaging

See Appendix E.

6.9. Vaccine accountability

See Appendix E.

6.10. Concomitant medication/treatment/vaccination

Study procedures for ALL SUBJECTS

Any recommended vaccine and any vaccine not foreseen in the study protocol administered since birth and during the entire study period is to be recorded with trade name (if the trade name is not available, the generic name is allowed), route of administration and date(s) of administration. Refer to Sections 4.3, 5.4 and 6.11.

Any systemic antibiotic treatment (oral or parenteral) administered at ANY time during the period starting with administration of the first dose of study vaccine and ending at visit 9 (24-27 months of age) for treatment of a CAP, AOM or ID episode are to be recorded with generic name of the medication (trade names are allowed for combination drugs, i.e. multi-component drugs), medical indication, total daily dose, route of administration, start and end dates of treatment. Topical antibiotic treatments such as eyedrops do not require recording.

Concomitant medication administered for the treatment of an SAE, at any time, must be recorded on the SAE Report Form with generic name of the medication (trade names are allowed for combination drugs only), medication indication (including which SAE), total daily dose, route of administration, start and end dates of treatment. Refer to Section 7.2 for definition of SAE.

Study procedures for ‘CARRIAGE’ SUBSET ONLY

At each study visit, the investigator should question the subject's parents/guardian about any systemic antibiotic treatment (oral or parenteral) administered at ANY time during the period starting with administration of the first dose of study vaccine and ending at visit 9 (24-27 months of age). This information should be recorded with generic name of the medication (trade names are allowed for combination drugs, i.e. multi-component

drugs), medical indication, total daily dose, route of administration, start and end dates of treatment. Topical antibiotic treatments such as eyedrops do not require recording.

6.11. Concomitant vaccination

6.11.1. GlaxoSmithKline Biologicals' Varilrix vaccine

The diluent and the reconstituted Varilrix vaccine should be inspected visually for any foreign particulate matter and/or variation of physical aspect prior to administration. In the event of either being observed, discard the diluent or the reconstituted vaccine.

Varilrix must be reconstituted by adding the contents of the supplied container of diluent to the vial containing the pellet. After the addition of the diluent to the pellet, the mixture should be well shaken until the pellet is completely dissolved in the diluent.

One dose (0.5 mL) of the varicella vaccine should be administered by subcutaneous administration. The upper arm (deltoid region) is the preferred site of injection.

The mixed vaccine should be used immediately after reconstitution.

6.11.1.1. Injection technique

Subcutaneous injections

These injections should be given in the upper arm by inserting the needle in a pinched-up fold of skin and subcutaneous tissue. A 23 or 25-gauge needle, 5/8-3/4 inch long is recommended. The needle should be changed between withdrawing the vaccine into the syringe and injecting it into the child.

The vaccinees will be observed closely for at least 30 minutes following the administration of vaccines, with appropriate medical treatment readily available in case of a rare anaphylactic reaction.

6.11.1.2. Contraindications

Varilrix is contra-indicated in subjects with a total lymphocyte count less than 1200 per mm^3 or presenting other evidence of lack of cellular immune competence.

Varilrix is contra-indicated in subjects with known systemic hypersensitivity to neomycin, but a history of contact dermatitis to neomycin is not a contra-indication.

6.11.2. Neisvac-C vaccine

6.11.2.1. Injection technique

Neisvac-C vaccine should be injected intramuscularly.

6.11.2.2. Contraindications

This vaccine is contra-indicated in subjects with hypersensitivity to any component of the vaccine, including tetanus toxoid.

As with any vaccine, administration of Neisvac-C should be postponed for subjects suffering from an acute severe febrile illness.

6.11.3. GlaxoSmithKline Biologicals' Rotarix vaccine

To prepare the oral live attenuated HRV (*Rotarix*) vaccine for administration, the entire content of the prefilled syringe (diluent) should be injected into the vial of the lyophilized product. The vial should be shaken well to resuspend the vaccine. The entire volume of the resuspended product should be withdrawn into the same syringe and the resuspended product should then be administered promptly as a single ORAL dose. Rotarix should under no circumstances be injected.

Should the subject regurgitate or vomit after the vaccine administration, a new vaccine dose may be administered at that visit.

6.11.3.1. Contraindications

This vaccine is contra-indicated in subjects with hypersensitivity to any component of the vaccine:

- Known hypersensitivity after previous administration of the HRV vaccine or to any component of the vaccine.
- History of chronic gastrointestinal disease including any uncorrected congenital malformation of the gastrointestinal tract.
- Previous history of intussusception (IS).

Administration of *Rotarix* should be postponed for subjects suffering from an acute severe febrile illness or from a gastroenteritis (GE) within 7 days preceding the study vaccine administration (i.e. diarrhoea with or without vomiting).

7. ADVERSE EVENTS AND SERIOUS ADVERSE EVENTS

The investigator is responsible for the detection and documentation of events meeting the criteria and definition of an adverse event (AE) for subjects enrolled in Panama or serious adverse event (SAE) for all subjects. During the study, the investigator or site staff will be responsible for detecting AEs and SAEs, as detailed in this section of the protocol.

Each subject's parents/guardians will be instructed to contact the investigator immediately should the subject manifest any signs or symptoms they perceive as serious.

7.1. Definition of an adverse event

An AE is any untoward medical occurrence in a clinical investigation subject, temporally associated with the use of a medicinal product, whether or not considered related to the medicinal product.

An AE can therefore be any unfavourable and unintended sign (including an abnormal laboratory finding), symptom, or disease (new or exacerbated) temporally associated with the use of a medicinal product. For marketed medicinal products, this also includes failure to produce expected benefits (i.e. lack of efficacy), abuse or misuse.

Examples of an AE include:

- Exacerbation of a chronic or intermittent pre-existing condition including either an increase in frequency and/or intensity of the condition.
- New conditions detected or diagnosed after investigational product administration even though it may have been present prior to the start of the study.
- Signs, symptoms, or the clinical sequelae of a suspected interaction.
- Signs, symptoms, or the clinical sequelae of a suspected overdose of either investigational product or a concurrent medication (overdose per se should not be reported as an AE/SAE).
- Signs, symptoms temporally associated with vaccine administration.

Examples of an AE DO NOT include:

- Medical or surgical procedure (e.g., endoscopy, appendectomy); the condition that leads to the procedure is an AE.
- Situations where an untoward medical occurrence did not occur (social and/or convenience admission to a hospital).
- Anticipated day-to-day fluctuations of pre-existing disease(s) or condition(s) present or detected at the start of the study that do not worsen.
- The disease/disorder being studied, or expected progression, signs, or symptoms of the disease/disorder being studied, unless more severe than expected for the subject's condition.

AEs may include pre- or post-treatment events that occur as a result of protocol-mandated procedures (i.e. invasive procedures, modification of subject's previous therapeutic regimen).

N.B. AEs to be recorded as endpoints (solicited events in subjects included in the 'Immuno and reacto' subset) are described in Section 7.5.1. All other AEs in subjects enrolled in Panama will be recorded as UNSOLICITED AEs.

Example of events to be recorded in the medical history section of the eCRF:

- Pre-existing conditions or signs and/or symptoms present in a subject prior to the start of the study (i.e. prior to the first study vaccination).

7.2. Definition of a serious adverse event

A serious adverse event (SAE) is any untoward medical occurrence that:

a. results in death,

b. is life-threatening,

NOTE: The term 'life-threatening' in the definition of 'serious' refers to an event in which the subject was at risk of death at the time of the event. It does not refer to an event, which hypothetically might have caused death, if it were more severe.

c. requires hospitalization or prolongation of existing hospitalization,

NOTE: In general, hospitalization signifies that the subject has been detained (usually involving at least an overnight stay) at the hospital or emergency ward for observation and/or treatment that would not have been appropriate in the physician's office or out-patient setting. Complications that occur during hospitalization are AEs. If a complication prolongs hospitalization or fulfils any other serious criteria, the event is serious. When in doubt as to whether "hospitalization" occurred or was necessary, the AE should be considered serious.

Hospitalization for elective treatment of a pre-existing condition that did not worsen from baseline is not considered an AE.

d. results in disability/incapacity, or

NOTE: The term disability means a substantial disruption of a person's ability to conduct normal life functions. This definition is not intended to include experiences of relatively minor medical significance such as uncomplicated headache, nausea, vomiting, diarrhoea, influenza, and accidental trauma (e.g. sprained ankle) which may interfere or prevent everyday life functions but do not constitute a substantial disruption.

e. is a congenital anomaly/birth defect in the offspring of a study subject.

f. Medical or scientific judgement should be exercised in deciding whether reporting is appropriate in other situations, such as important medical events that may not be immediately life-threatening or result in death or hospitalization but may jeopardize the subject or may require medical or surgical intervention to prevent one of the

other outcomes listed in the above definition. These should also be considered serious. Examples of such events are invasive or malignant cancers, intensive treatment in an emergency room or at home for allergic bronchospasm, blood dyscrasias or convulsions that do not result in hospitalization.

7.3. Documentation of cases of disease

7.3.1. Documentation of any meningitis and/or invasive disease

The investigator will make sure that all relevant and supportive information (clinical, biological etc…) of any subject participating in this study with (indications of) bacteraemia, septicemia, meningitis, meningism or any other symptom indicating possible pneumococcal or *Haemophilus influenzae* disease is made available to fully document the case. The investigator will immediately contact the local monitor and discuss further diagnostic possibilities in order to properly document the case.

Any case of meningitis**,** regardless of the etiology, should be reported in the SAE Report Form as a serious adverse event and in the meningitis report form, and should be recorded in a specific eCRF page. In case of a confirmed meningitis, the corresponding pages in the Invasive Disease section, including the Lumbar Puncture section, should be filled in. In case of a probable meningitis, only the Lumbar Puncture section should be filled in.

7.3.2. Documentation of CAP and AOM

The investigator will make sure that all relevant and supportive information (clinical, biological etc…) of any subject participating in this study with CAP or AOM is made available to fully document the case.

7.4. Clinical laboratory parameters and other abnormal assessments qualifying as adverse events and serious adverse events

In all subjects, abnormal laboratory findings (e.g., clinical chemistry, haematology, urinalysis) or other abnormal assessments (e.g., ECG, X-rays, vital signs etc) that are detected during the study or are present at baseline and significantly worsen following the start of the study and that are judged by the investigator to be clinically significant, will be recorded as SAEs if they meet the definition of an SAE, provided in Section 7.2. If these abnormal findings are not considered to be an SAE, they will be reported as AEs if they meet the definition of an AE as provided in Section 7.1, and if the subject is enrolled in Panama.

The investigator will exercise his or her medical and scientific judgement in deciding whether an abnormal laboratory finding or other abnormal assessment is clinically significant.

7.5. Time period, frequency, and method of detecting adverse events and serious adverse events

The investigator will inquire about the occurrence of AEs/SAEs at every visit/contact during the study.

Study procedures for SUBJECTS IN PANAMA

All AEs occurring from the administration of the first vaccine dose up to visit 9 (24-27 months of age) must be recorded on the Adverse Event form in the subject's eCRF, irrespective of intensity or whether or not they are considered vaccination-related. All AEs either observed by the investigator or one of his clinical collaborators or reported by the subject’s parent/guardian spontaneously or in response to a direct question will be evaluated by the investigator. AEs not previously documented in the study will be recorded in the Adverse Event form within the subject's eCRF. The nature of each event, date and time (where appropriate) of onset, outcome, intensity and relationship to vaccination should be established. Refer to Section 7.7.

As a consistent method of soliciting AEs, the subject’s parent/guardian should be asked a non-leading question such as:

"Has your child acted differently or felt different in any way since receiving the vaccine or since the last visit?"

AEs already documented in the eCRF, i.e. at a previous assessment, and designated as “not recovered/not resolved” or “recovering/resolving” should be reviewed at subsequent visits, as necessary. If these have resolved, the documentation in the eCRF should be completed.

Study procedures for ALL SUBJECTS

For all subjects, even if not part of the subjects enrolled in Panama, AEs have to be documented in case***:*** 1) this AE is a reason for withdrawal of the subject from the study or from study vaccination, or 2) the AE refers to a convulsion episode occurring within 30 days after booster vaccination (Visit 6), and to be reported at Visit 8. Any case of convulsions occurring within 30 days after vaccine administration at Visit 6 should be recorded in a specific eCRF page. Otherwise, safety assessment in subjects who are not part of the ‘Immuno and reacto’ subset is limited to the SAE reporting.

The standard time period for collecting and recording SAEs will begin at the first receipt of study vaccine/ control/ co-administered vaccine and will continue during the entire study period (until 24 months of age). See Section 7.8 for instructions for reporting and recording SAEs. In addition, in order to fulfil international reporting obligations, SAEs that are related to study participation (e.g. protocol-mandated procedures, invasive tests, a change from existing therapy) will be collected and recorded from the time the subject consents to participate in the study until she/he is discharged**.**

When a reportable AE/SAE occurs, it is the responsibility of the investigator to review all documentation (e.g., hospital progress notes, laboratory, and diagnostics reports) relative to the event. The investigator will then record all relevant information regarding an AE/SAE on the eCRF or SAE Report Form as applicable. It is not acceptable for the investigator to send photocopies of the subject's medical records to GSK Biologicals in lieu of the appropriate completed AE/SAE pages. However, there may be instances when copies of medical records for certain cases are requested by GSK Biologicals. In this instance, all direct subject identifiers (e.g. name, address, initials etc) will be blinded on the copies of the medical records prior to submission to GSK Biologicals.

The investigator will attempt to establish a diagnosis of the event based on signs, symptoms, and/or other clinical information. In such cases, the diagnosis should be documented as the AE/SAE and not the individual signs/symptoms.

7.5.1. Solicited adverse events

Study procedures for 'IMMUNO AND REACTO' SUBSET ONLY

Solicited local (injection site) AEs

Table 19 Solicited local adverse events

Pain at injection site
Redness at injection site
Swelling at injection site

N.B. If parents/guardians observe any large injection site reactions (defined as swelling with a diameter > 50 mm; noticeable diffuse swelling or noticeable increase of limb circumference) after booster study vaccine dose given at 15-18 months of age, they will be asked to contact study personnel and to bring the child as soon as possible to the investigator's office for evaluation. The investigator will record detailed information, describing the adverse event on a specific "Large Injection Site reactions report".

Solicited general AEs

Table 20 Solicited general adverse events

Drowsiness
Fever
Irritability/ Fussiness
Loss of appetite

N.B. Temperature will be recorded in the evening. Should additional temperature measurements be performed at other times of day, the highest temperature will be recorded.

Intensity of solicited AEs will be assessed as follows:

Table 21 Intensity scales for solicited symptoms

Adverse Event	Intensity grade	Parameter
Pain at injection site	0	Absent
	1	Minor reaction to touch
	2	Cries/protests on touch
	3	Cries when limb is moved/spontaneously painful
Redness at injection site		Record greatest surface diameter in mm
Swelling at injection site		Record greatest surface diameter in mm
Fever*		Record temperature in °C
Irritability/Fussiness	0	Behaviour as usual
	1	Crying more than usual/ no effect on normal activity
	2	Crying more than usual/ interferes with normal activity
	3	Crying that cannot be comforted/ prevents normal activity
Drowsiness	0	Behaviour as usual
	1	Drowsiness easily tolerated
	2	Drowsiness that interferes with normal activity
	3	Drowsiness that prevents normal activity
Loss of appetite	0	Appetite as usual
	1	Eating less than usual/ no effect on normal activity
	2	Eating less than usual/ interferes with normal activity
	3	Not eating at all

*Fever is defined as: rectal temperature ≥38.0°C/ axillary/oral/ tympanic temperature ≥37.5°C.

The maximum intensity of local injection site redness/swelling will be scored at GSK Biologicals as follows:

0 : Absent
1 : >0 to ≤ 20 mm
2 : >20 to ≤ 30 mm
3 : > 30 mm

Grade 3 fever will be defined as rectal temperature > 40.0°C

7.6. Evaluating adverse events and serious adverse events

7.6.1. Assessment of intensity

The investigator will make an assessment of the maximum intensity that occurred over the duration of the event for all reportable unsolicited symptoms, including SAEs reported during the study. The assessment will be based on the investigator’s clinical judgement. The intensity of each AE and SAE recorded in the eCRF or SAE Report Form, as applicable, should be assigned to one of the following categories:

1 (mild)	=	An AE which is easily tolerated by the subject, causing minimal discomfort and not interfering with everyday activities.
2 (moderate)	=	An AE which is sufficiently discomforting to interfere with normal everyday activities.
3 (severe)	=	An AE which prevents normal, everyday activities. (In a young child, such an AE would, for example, prevent attendance at school/ kindergarten/ a day-care centre and would cause the parents/ guardians to seek medical advice.)

An AE that is assessed as Grade 3 (severe) should not be confused with a SAE. Grade 3 is a category utilized for rating the intensity of an event; and both AEs and SAEs can be assessed as Grade 3. An event is defined as 'serious' when it meets the definition as described in Section 7.2.

7.6.2. Assessment of causality

The investigator is obliged to assess the relationship between the administration of investigational product and the occurrence of each reportable AE/SAE. The investigator will use clinical judgement to determine the relationship. Alternative causes, such as natural history of the underlying diseases, concomitant therapy, other risk factors and the temporal relationship of the event to the administration of the investigational product will be considered and investigated. The investigator will also consult the Investigator Brochure and/or Product Information, for marketed products, in the determination of his/her assessment.

There may be situations when a SAE has occurred and the investigator has minimal information to include in the initial report to GSK Biologicals. However, it is very important that the investigator always makes an assessment of causality for every event prior to transmission of the SAE Report Form to GSK Biologicals. The investigator may change his/her opinion of causality in light of follow-up information, amending the SAE Report Form accordingly. The causality assessment is one of the criteria used when determining regulatory reporting requirements.

In case of concomitant administration of multiple vaccines, it may not be possible to determine the causal relationship of general AEs to the individual vaccines administered. The investigator should, therefore, assess whether the AE could be causally related to vaccination rather than to the individual vaccines.

All solicited local (injection site) reactions will be considered causally related to vaccination. Causality of all other AEs should be assessed by the investigator using the following question:

Is there a reasonable possibility that the AE may have been caused by the investigational product?

NO	:	The AE is not causally related to administration of the study vaccine(s). There are other, more likely causes and administration of the study vaccine(s) is not suspected to have contributed to the AE.
YES	:	There is a reasonable possibility that the vaccine(s) contributed to the AE.

Non-serious and serious AEs will be evaluated as two distinct events. If an event meets the criteria to be determined "serious" (see Section 7.2 for definition of serious adverse event), it will be examined by the investigator to the extent to be able to determine ALL contributing factors applicable to each serious adverse event.

Other possible contributors include:

- Medical history
- Other medication
- Protocol required procedure
- Other procedure not required by the protocol
- Lack of efficacy of the vaccine(s), if applicable
- Erroneous administration
- Other cause (specify).

7.6.3. Medically attended visits

For each solicited and unsolicited symptom the subject experiences and that is to be reported according to the subset the subject belongs to, the subject's parents/guardians will be asked if they received medical attention defined as hospitalization, an emergency room visit or a visit to or from medical personnel (medical doctor) for any reason and this information will be recorded in the eCRF.

7.7. Follow-up of adverse events and serious adverse events and assessment of outcome

After the initial AE/SAE report, the investigator is required to proactively follow each subject and provide further information to GSK Biologicals on the subject's condition.

All AEs and SAEs documented at a previous visit/contact and designated as not recovered/not resolved or recovering/resolving will be reviewed at subsequent visits/contacts.

Investigators will follow-up subjects:

- with SAEs or subjects withdrawn from the study as a result of an AE, until the event has resolved, subsided, stabilized, disappeared, the event is otherwise explained, or the subject is lost to follow-up;
- or, in the case of other non-serious AEs occurring in subjects enrolled in Panama, until they complete the study or they are lost to follow-up.

Clinically significant laboratory abnormalities occurring in subjects enrolled in Panama will be followed up until they have returned to normal, or a satisfactory explanation has been provided. Additional information (including but not limited to laboratory results) relative to the subsequent course of such an abnormality must be made available to the Study Monitor.

GSK Biologicals may request that the investigator perform or arrange for the conduct of supplemental measurements and/or evaluations to elucidate as fully as possible the nature and/or causality of the AE or SAE. The investigator is obliged to assist. If a subject dies during participation in the study or during a recognized follow-up period, GSK Biologicals will be provided with a copy of any available post-mortem findings, including histopathology.

New or updated information will be recorded on the originally completed SAE Report Form, with all changes signed and dated by the investigator. The updated SAE report form should be resent to GSK Biologicals within 24 hours of receipt of the follow-up information as outlined in Section 7.8.

Outcome of any non-serious AE occurring in subjects enrolled in Panama or any SAE occurring in the entire study cohort and reported during the entire study will be assessed as:

- Recovered/resolved
- Not recovered/not resolved
- Recovering/resolving
- Recovered with sequelae/resolved with sequelae
- Fatal (SAEs only).

7.8. Prompt reporting of serious adverse events to GSK Biologicals

7.8.1. Time frames for submitting serious adverse event reports to GSK Biologicals

SAEs will be reported promptly to GSK once the investigator determines that the event meets the protocol definition of an SAE. The investigator or designate will fax the SAE reports to GSK Biologicals' Study Contact for Serious Adverse Event Reporting **WITHIN 24 HOURS OF HIS/HER BECOMING AWARE OF THESE EVENTS**. Additional or follow-up information relating to the initial SAE report is also to be reported to the GSK Biologicals' Study Contact for Serious Adverse Event Reporting within 24 hours of receipt of such information.

7.8.2. Completion and transmission of serious adverse event reports to GSK Biologicals

Once an investigator becomes aware that a SAE has occurred in a study subject, she/he will report the information to GSK within 24 hours as outlined in Section 7.8.1. The SAE Report Form will always be completed as thoroughly as possible with all available details of the event, signed by the investigator (or designee), and forwarded to GSK within the designated time frames. If the investigator does not have all information regarding an SAE, he/she will not wait to receive additional information before notifying GSK of the event and completing the form. The form will be updated when additional information is received and forwarded to GSK **WITHIN 24 HOURS** as outlined in Section 7.8.1.

The investigator will always provide an assessment of causality at the time of the initial report as described in Section 7.6.2.

Facsimile (Fax) transmission of the SAE Report Form is the preferred method to transmit this information to the Study Contact for Reporting SAEs. In rare circumstances and in the absence of facsimile equipment, notification by telephone is acceptable, with a copy of the SAE Report Form sent by overnight mail. Initial notification via the telephone does not replace the need for the investigator to complete and sign the SAE Report Form within 24 hours as outlined in Section 7.8.1.

In the event of a death determined by the investigator to be related to vaccination, sending of the fax must be accompanied by telephone call to the Study Contact for Reporting SAEs.

Study Contact for Reporting SAEs
**Please see Sponsor Information Sheet for contact details**
**Back-up Study Contact for Reporting SAEs**
**GSK Biologicals Regional Clinical Safety Physician**<br>Tel: +55-21-21 41 60 77<br>Fax: +55-21-21 41 62 32<br>Mobile phone for 7/7 day availability: +55-21-93 66 02 74<br>**24/24 hour and 7/7 day availability**

7.9. Regulatory reporting requirements for serious adverse events

The investigator will promptly report all SAEs to GSK in accordance with the procedures detailed in Section 7.8. GSK Biologicals has a legal responsibility to promptly notify, as appropriate, both the local regulatory authority and other regulatory agencies about the safety of a product under clinical investigation. Prompt notification of SAEs by the investigator to the Study Contact for Reporting SAEs is essential so that legal obligations and ethical responsibilities towards the safety of other subjects are met.

The investigator, or responsible person according to local requirements, will comply with the applicable local regulatory requirements related to the reporting of SAEs to the IRB/IEC and, if required, to the applicable government authority.

Investigator safety reports are prepared according to the current GSK policy and are forwarded to investigators as necessary. An investigator safety report is prepared for a SAE(s) that is both attributable to investigational product and unexpected. The purpose of the report is to fulfil specific regulatory and Good Clinical Practice (GCP) requirements, regarding the product under investigation.

An investigator who receives an investigator safety report describing a SAE(s) or other specific safety information (e.g., summary or listing of SAEs) from GSK Biologicals will file it with the Investigator Brochure or other appropriate study documentation and will notify the IRB or IEC, if appropriate according to local requirements.

7.10. Post-study adverse events and serious adverse events

A post-study AE/SAE is defined as any event that occurs outside of the AE/SAE detection period defined in Section 7.5. Investigators are not obligated to actively seek AEs or SAEs in former study participants.

However, if the investigator learns of any SAE, including a death, at any time after a subject has been discharged from the study, and he/she considers the event reasonably related to the investigational product, the investigator will promptly notify the Study Contact for Reporting SAEs.

7.11. Pregnancy

Not applicable.

7.12. Treatment of adverse events

Treatment of any adverse event is at the sole discretion of the investigator and according to local medical practice. Specific medication will be made available by the Sponsor to comply with existing local programs for treatment of the most common adverse events related to vaccination.

8. SUBJECT COMPLETION AND WITHDRAWAL

8.1. Subject completion

A subject who participated to the study conclusion (Contact 10) foreseen in the protocol is considered to have completed the study.

8.2. Subject withdrawal

Subjects who are withdrawn because of SAEs/AEs must be clearly distinguished from subjects who are withdrawn for other reasons. In case of withdrawal due to an AE, the AE has to be documented in the appropriate section of the eCRF, even if the subject is not included in the subset enrolled in Panama. Investigators will follow subjects who are withdrawn as result of a SAE/AE until resolution of the event (see Section 7.7).

Withdrawals will not be replaced.

8.2.1. Subject withdrawal from the study

From an analysis perspective, a ‘withdrawal’ from the study is any subject who did not participate in the study conclusion (Contact 10).

A subject qualifies as a ‘withdrawal’ from the study when no study procedure has occurred, no follow-up has been performed and no further information has been collected for this subject from the date of withdrawal/last contact.

Investigators will make an attempt to contact those subjects who do not return for scheduled visits or follow-up.

Information relative to the withdrawal will be documented on the Study Conclusion page of the eCRF. The investigator will document whether the decision to withdraw from the study was made by the subject’s parent or guardian or the investigator and which of the following possible reasons (applicable for all subjects) was responsible for withdrawal:

- serious adverse event

- non-serious adverse event
- protocol violation (specify)
- consent withdrawal, not due to an adverse event
- moved from the study area
- lost to follow-up
- other (specify).

8.2.2. Subject withdrawal from investigational product

A ‘withdrawal’ from the investigational product is any subject who does not receive the complete study immunization course, i.e. when no further planned study vaccine dose is administered from the date of withdrawal. A subject withdrawn from the investigational product may not necessarily be withdrawn from the study as further study procedures or follow-up may be performed (efficacy, safety or immunogenicity) if planned in the study protocol.

Information relative to premature discontinuation of the investigational product will be documented on the Vaccine Administration page of the eCRF. The investigator will document whether the decision to discontinue further vaccination / treatment was made by the subject’s parent or guardian or the investigator and which of the following possible reasons was responsible for withdrawal:

- serious adverse event,
- non-serious adverse event,
- other (specify).

9. DATA EVALUATION: CRITERIA FOR EVALUATION OF OBJECTIVES

9.1. Primary endpoint

Occurrence of likely bacterial CAP cases (B-CAP) defined as radiologically confirmed CAP cases with either alveolar consolidation/pleural effusion on the chest X-ray or with non-alveolar infiltrates but with CRP $\geq$ 40 mg/L.

9.2. Secondary endpoints

Efficacy:

- Occurrence of clinically confirmed AOM cases (C-AOM) (*in all subjects enrolled in Panama*)
- Occurrence of CAP cases with alveolar consolidation or pleural effusion on the Chest X-ray (C-CAP)

- ~~Occurrence of bacteriologically confirmed AOM cases (B-AOM) caused by vaccine serotypes, cross-reactive and other pneumococcal serotypes or by *H. influenzae (in all subjects enrolled in Panama)*~~
- ***Occurrence of bacteriologically confirmed AOM cases (B-AOM) caused by any bacterial pathogen (in all subjects enrolled in Panama)***
- ***Occurrence of bacteriologically confirmed AOM cases (B-AOM) caused by vaccine serotypes, cross-reactive and other pneumococcal serotypes (in all subjects enrolled in Panama)***
- ***Occurrence of bacteriologically confirmed AOM cases (B-AOM) caused by H. influenzae (in all subjects enrolled in Panama)***
- ***Occurrence of bacteriologically confirmed AOM cases (B-AOM) caused by non-typeable H. influenzae (in all subjects enrolled in Panama)***
- ***Occurrence of bacteriologically confirmed AOM cases (B-AOM) caused by other AOM pathogens (for example Moraxella catarrhalis, Group A streptococci and Staphylococcus aureus) (in all subjects enrolled in Panama)***
- Occurrence of ~~confirmed~~ CAP cases ***with alveolar consolidation or pleural effusion on the Chest X-ray (C-CAP)*** ~~associated with respiratory viral infection~~ ***with positive respiratory viral test***
- ***Occurrence of CAP cases with any abnormal CXR with positive respiratory viral test***
- ***Occurrence of likely bacterial CAP (B-CAP) cases with positive respiratory viral test***

  (Amended 09 September2010)

- Occurrence of suspected CAP cases
- Occurrence of CAP cases with any abnormal CXR
- Occurrence of suspected CAP cases with CRP ≥ 40 mg/L, regardless of CXR reading
- Occurrence of suspected CAP cases with CRP ≥ 80 mg/L, regardless of CXR reading
- Occurrence of suspected CAP cases with CRP ≥ 120 mg/L, regardless of CXR reading
- Occurrence of CAP cases with either alveolar consolidation/pleural effusion on the chest X-ray or with non-alveolar infiltrates but with CRP ≥ 80 mg/L
- Occurrence of CAP cases with either alveolar consolidation/pleural effusion on the chest X-ray or with non-alveolar infiltrates but with CRP ≥ 120 mg/L
- Occurrence of bacteriologically culture confirmed IPD cases caused by any of the 10 pneumococcal vaccine serotypes (VT-IPD)

- Occurrence of VT-IPD cases identified through positive culture or through nonculture pneumococcal diagnostic tests (for example Binax NOW or Latex agglutination) with additional nonculture VT serotyping
- Occurrence of IPD cases due to cross-reactive pneumococcal serotypes and other pneumococcal serotypes
- Occurrence of invasive disease cases due to *H. influenzae*

Carriage *(in the 'Carriage' subset, a subgroup of 2,000 subjects enrolled in Panama)*:

- Occurrence of vaccine serotypes, cross-reactive or other *S. pneumoniae* serotypes and *H. influenzae* in the nasopharynx
- Acquisition of new *S. pneumoniae* and/or *H. influenzae* strains in the nasopharynx

Antibiotic treatment *(in the 'Carriage' subset, a subgroup of 2,000 subjects enrolled in Panama)*:

- Occurrence of antibiotic prescriptions during the entire study period

Safety *(in all subjects):*

- Occurrence of serious adverse events from the administration of the first vaccine dose up to study end

Safety *(in all subjects enrolled in Panama):*

- Occurrence of any unsolicited adverse event from the administration of the first vaccine dose up to visit 9 (24-27 months of age)

Reactogenicity *(in the 'Immuno and reacto' subset of 1,000 subjects (500* subjects respectively in Argentina and Panama))*:*

- Occurrence of solicited local symptoms (redness, swelling, pain) within 4 days (day 0 – day 3) after each study vaccine administration
- Occurrence of solicited general symptoms (fever, irritability/fussiness, drowsiness, loss of appetite) within 4 days (day 0 – day 3) after each study vaccine administration

Immunogencity *(in the 'Immuno and reacto' subset of 1,000 subjects (500* subjects respectively in Argentina and Panama))*:*

One month after the third vaccination (at approx. 7 months of age), just before the booster dose (at 15-18 months of age), one month post-booster dose (at 16-19 months of age) with GSK Biologicals' 10-valent pneumococcal conjugate vaccine and at last scheduled visit (24-27 months of age):

- Pneumococcal antibody concentrations against serotypes 1, 4, 5, 6A, 6B, 7F, 9V, 14, 18C, 19A, 19F and 23F ≥ 0.20 µg/mL as measured by 22F-ELISA
- Antibody concentrations against pneumococcal serotypes 1, 4, 5, 6A, 6B, 7F, 9V, 14, 18C, 19A, 19F and 23F

- Opsonophagocytic activity against pneumococcal serotypes 1, 4, 5, 6A, 6B, 7F, 9V, 14, 18C, 19A, 19F and 23F
- Antibody concentrations against protein D
- Seropositivity status, defined as:
  - Pneumococcal antibody concentrations against serotypes 1, 4, 5, 6A, 6B, 7F, 9V, 14, 18C, 19A, 19F and 23F ≥ 0.05 µg/mL
  - Opsonophagocytic activity against pneumococcal serotypes 1, 4, 5, 6A, 6B, 7F, 9V, 14, 18C, 19A, 19F and 23F ≥ 8
  - Anti-PD antibody concentrations ≥ 100 EL.U/mL

9.3. Estimated sample size

9.3.1. Efficacy objectives

9.3.1.1. Primary objective (B-CAP ~~or C-AOM~~)

The primary objective is to demonstrate that the vaccine efficacy (VE), defined as 1-Hazard ratio (HR), induced by the 10Pn-PD-DiT vaccine (three doses in subjects ≤ 18 months of age or four doses in subjects > 18 months of age) in preventing the first episode of likely bacterial CAP (B-CAP) is greater than 0%, as compared to the control group.

COMPAS study design would allow early efficacy conclusion if true vaccine efficacy would be in line with our current estimation (25%) while maintaining the possibility to conclude later if the VE would be slightly lower than anticipated (for example, 20%). This leads to the introduction of a planned interim analysis when we will reach a required number of B-CAP cases.

In order to keep the overall one-sided significance level for the primary endpoint at 2.5%, the following alpha adjustment based on the Pocock approach will be applied to take into account this interim analysis:

When the number of first B-CAP from 14 days post dose 3 reported in the ATP cohort reaches 535, the ~~The~~ group receiving the 10Pn-PD-DiT vaccine will be compared to the Control group using a one-sided test at 1.75% nominal alpha level ~~(at interim analysis)~~ ***i.e.*** ~~and at 1.19% nominal alpha level (at final analysis). The success will be established~~ ***the interim analysis will be considered conclusive*** if the one-sided p-value calculated for the null hypothesis H0=[B-CAP VE ≤ 0%] is lower than 1.75% ~~(at interim analysis) and lower than 1.19% (at final analysis) (EAST Survival Module: One sided test with Early rejection of H0 only)~~.

If the interim analysis is not conclusive the study will continue and study end will be organized approximately between September and December 2011. The final analysis will then take place and the group receiving the 10Pn-PD-DiT vaccine will be compared to the Control group using a one-sided test and an adjusted nominal alpha

level. The nominal alpha level will be adjusted according to the number of B-CAP at interim analysis and at the final analysis to ensure the total type I error is maintained to 2.5%.

Table 22 provides the power for B-CAP efficacy at the interim analysis and at the final analysis according to various VE assumption and first B-CAP cases at final analysis. If the true VE is 25%, the study has 87.6% power to be conclusive at the interim analysis. If the interim is not conclusive, Figure 8 provides projection on the number of evaluable B-CAP expected at study end (=end 2011) and shows that one can consider that the total number of evaluable B-CAP cases at the final analysis will be between 600 and 700.

Therefore if the true VE is 20% and the interim analysis is not conclusive despite a 66% power at the interim there is at least 9.7% chance to conclude at the final analysis with a global power of at least 75.7%

Table 22 Power for B-CAP efficacy according to the number of events at interim and final analyses

Number of events at final analysis	*1-sided alpha at final analysis**	*True VE*	*Power at Interim*	*Power at final***	*Global Power*
600	*2.024016%*	*20.0%*<br>*22.5%*<br>*25.0%*	*66.0%*<br>*78.1%*<br>*87.6%*	*9.7%*<br>*7.9%*<br>*5.4%*	*75.7%*<br>*86.0%*<br>*93.0%*
625	*1.894783%*	*20.0%*<br>*22.5%*<br>*25.0%*	*66.0%*<br>*78.1%*<br>*87.6%*	*11.6%*<br>*9.4%*<br>*6.4%*	*77.6%*<br>*87.5%*<br>*91.0%*
650	*1.790330%*	*20.0%*<br>*22.5%*<br>*25.0%*	*66.0%*<br>*78.1%*<br>*87.5%*	*11.6%*<br>*9.5%*<br>*6.6%*	*77.6%*<br>*87.6%*<br>*94.1%*
675	*1.704119%*	*20.0%*<br>*22.5%*<br>*25.0%*	*66.0%*<br>*78.1%*<br>*87.6%*	*12.2%*<br>*10.7%*<br>*7.3%*	*79.2%*<br>*88.8%*<br>*94.9%*
700	*1.631312%*	*20.0%*<br>*22.5%*<br>*25.0%*	*66.0%*<br>*78.1%*<br>*87.6%*	*14.7%*<br>*11.8%*<br>*8.0%*	*80.7%*<br>*89.9%*<br>*95.5%*

** nominal alpha at final analysis is adjusted to reach 2.5% global alpha considering an interim analysis with 535 events and an interim nominal 1-sided alpha of 1.75% (EAST, 1-sided test for a binomial distribution, see Appendix J on how testing VE can be expressed as testing one proportion)*
*** probability to succeed at the final analysis and to fail at the interim analysis*

Figure 7 Projection of the number of first B-CAP episodes from 2 weeks after dose 3 (Total vaccinated cohort) (10% drop-out /year)

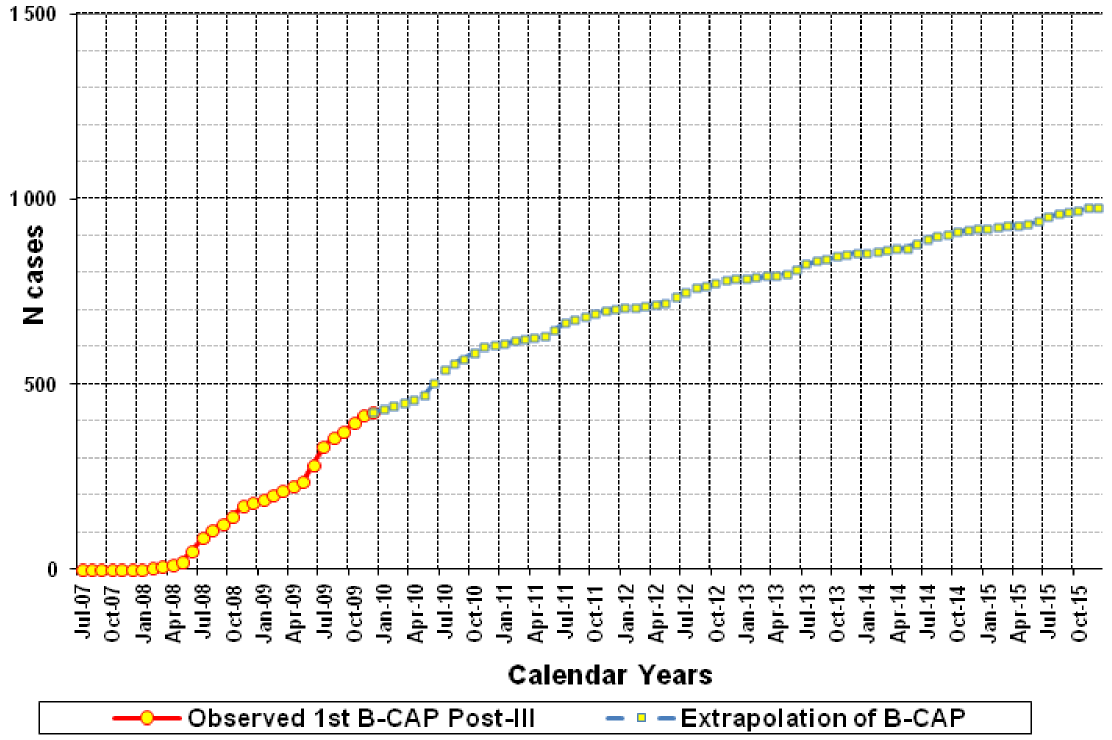

Observed 1st B-CAP Post-dose III = number of observed 1st B-CAP cases up to December 2009. Extrapolation of B-CAP = extrapolation beyond December 2009 assuming no limit in study duration but taking into account an expected drop of 50% in incidence of B-CAP (compared to the observed incidence in the second year of life) when children reach 2 years of age.

~~Considering a 1:1 randomization ratio, a true VE against B-CAP of 20% a total number of **1045 first B-CAP episodes** (= subjects with evaluable likely bacterial CAP) in the study will be needed to demonstrate VE > 0% with 90% power at final analysis (PASS 2005 - one-sided test for one proportion, nominal type I error of 1.19%, see details in Appendix J).~~

~~The interim analysis will be done when we will reach **535 first B-CAP episodes, with a minimum study duration of 18 months after study start (see Section 9.7).**~~

~~Considering the nominal 1-sided alpha level of 1.75% to be used at the interim analysis, the power to demonstrate VE > 0% at the time of the interim analysis will be 66% based on a true VE of 20%, 87% based on a true VE of 25% and 97% if the true VE is 30% (PASS 2005 - one-sided test for one proportion, nominal type I error of 1.75%, see details in Appendix J).~~

~~To be considered as evaluable for primary analysis, an episode must appear in the ATP cohort for efficacy, more than two weeks after the administration of the third dose of study vaccine, and must fulfil the criteria defined in Section 4.5~~

Based on the incidence rates as shown in Table 23, an enrolment period of 12 months and 10% withdrawal in the ATP cohort for efficacy, a total number of **24,000 subjects** (**12,000 subjects per group**) will need to be enrolled in order to reach the initial planned number of first B-CAP episodes of at least 1200 approximately 36 months after study start (study end).

Table 23 Likely Bacterial CAP incidences

Endpoint	1-5 months of age	6-11 months of age	12-23 months of age	24-35 months of age
B-CAP* (/100,000 child months)	345.35	329.40	245.40	179.56

*Note that we make the hypothesis that 15% of cases ocurs in excluded high risk groups (percentage included in the referenced incidence rates)
Source: Santiago (2004-2005, INE)

Figure 8 shows the estimated evolution over time of the number of B-CAP cases occurring during the entire study period.

Figure 8 Estimated evolution of the number of B-CAP cases

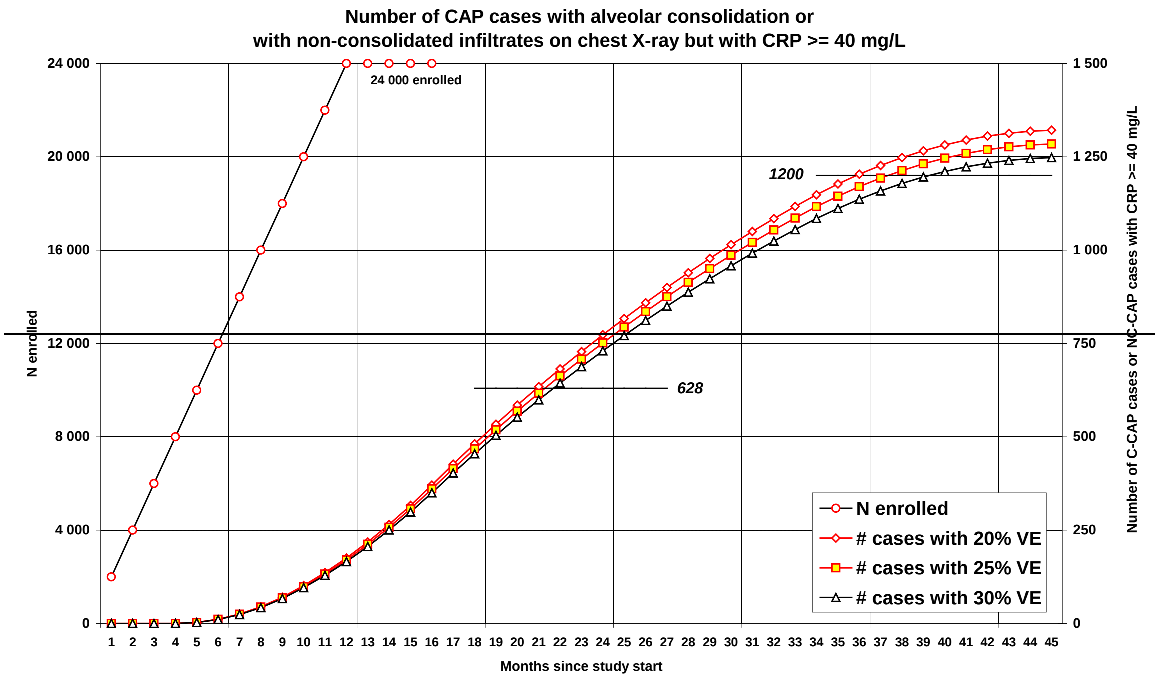

Based on a 1:1 randomization ratio, 12 months enrollment, 24,000 enrolled subjects and 10% withdrawal, an estimated true VE against B-CAP of 20%, LL of 95%CI > 0%, power = 90%, ATP efficacy follow-up starting 2 weeks after the third vaccine dose, baseline incidence estimates according to the average of the incidence rates of Santiago de Chile (Jan-2004 to Dec-2005, INE) (see Table 23)

(Amended 09 September2010)

9.3.1.2. Secondary objective (clinical AOM – C-AOM) at study end

The first secondary objective is to demonstrate that the VE induced by the 10Pn-PD-DiT vaccine (three doses in subjects ≤ 18 months of age or four doses in subjects > 18 months of age) in preventing the first episode of clinically confirmed AOM cases (C-AOM) is greater than 0%, as compared to the control group.

Considering a 1:1 randomization ratio and various true C-AOM VE, Table 24 shows the total number of first evaluable C-AOM episodes needed to demonstrate C-AOM VE > 0% with 80 or 90% power (one-sided test, nominal type I error of 2.5%).

Table 24 Total number of first episodes required to show C-AOM VE > 0% with 80% and 90% power, according to various true VE

	Required first episodes of C-AOM *	
True VE	80% power	90% power
10%	2,891	3,845
15%	1,226	1,637
20%	662	880
25%	405	535
30%	270	350
35%	184	244
40%	134	178
45%	99	132
50%	77	99
55%	61	77

*PASS 2005 - one-sided test for one proportion, nominal type I error of 2.5%.

The group receiving the 10Pn-PD-DiT vaccine will be compared to the Control group using a one-sided test at 2.5% nominal alpha level at final analysis. The success will be established if the primary objective has been reached and if the one-sided p-value calculated for the null hypothesis H0=[C-AOM VE ≤ 0%] is lower than 2.5% (at final analysis) (EAST Survival Module: One sided tests with Early rejection of H0 only).

9.3.1.3. Secondary objective (C-CAP) at study end

The efficacy of the 10Pn-PD-DiT vaccine in preventing confirmed CAP cases (C-CAP) will also be evaluated as secondary endpoint.

Considering a 1:1 randomization ratio and various true C-CAP VE, Table 25 shows the total number of first evaluable C-CAP episodes needed to demonstrate C-CAP VE > 0% with 80 or 90% power (one-sided test, nominal type I error of 2.5%).

Table 25 Total number of first episodes required to show C-CAP VE > 0% with 80% and 90% power, according to various true VE

True VE	Required first episodes of C-CAP*		Number of C-CAP 36 months after study start
	80% power	90% power	
20%	662	880	356
25%	405	535	347
30%	270	350	337
35%	184	244	327
40%	134	178	317
45%	99	132	307
50%	77	99	297

*PASS 2005 - one-sided test for one proportion, nominal type I error of 2.5%

Based on the incidence rates as shown in Table 26, an enrolment period of 12 months, 10% withdrawal in the ATP cohort for efficacy and a total number of 24,000 enrolled subjects, Table 25 shows the number of C-CAP that will be reached 36 months after study start based on various true VE. Table 25 thus shows that based on the previous hypotheses, we would have 80% power to show C-CAP VE > 0% if the true VE would be 30% and 90% power if the true VE would be 35%.

Table 26 Confirmed CAP incidences

Endpoint	1-5 months of age	6-11 months of age	12-23 months of age	24-35 months of age
C-CAP* (/100,000 child months)	110.45	84.96	71.97	64.53

*Note that we make the hypothesis that 15% of cases occurs in excluded high risk groups (percentage included in the referenced incidence rates)
Source: Santiago (2004-2005, INE)

9.3.1.4. Secondary objective (bacteriologically confirmed AOM – B-AOM) at study end

The efficacy of the 10Pn-PD-DiT vaccine in preventing bacteriologically confirmed AOM cases (B-AOM) caused by vaccine serotypes, cross-reactive and other pneumococcal serotypes or by *H. influenzae* will be evaluated as secondary endpoints.

Considering a 1:1 randomization ratio and various true B-AOM VE, Table 27 shows the total number of first evaluable B-AOM episodes needed to demonstrate B-AOM VE > 0% with 80 or 90% power (one-sided test, nominal type I error of 2.5%).

Table 27 Total number of first episodes required to show B-AOM VE > 0% with 80% and 90% power, according to various true VE

True VE	Required first episodes of B-AOM*	
	80% power	90% power
10%	2,891	3,845
15%	1,226	1,637
20%	662	880
25%	405	535
30%	270	350
35%	184	244
40%	134	178
45%	99	132
50%	77	99
55%	61	77

*PASS 2005 - one-sided test for one proportion, nominal type I error of 2.5%

9.3.1.5. Secondary objective (VT-IPD) at study end

Impact on bacteriologically culture confirmed IPD cases caused by any of the 10 pneumococcal vaccine serotypes (VT-IPD) will also be documented. As sufficient VT-IPD cases to reach statistical significant result can't be guaranteed, VT-IPD endpoint will only be descriptive.

Considering a 1:1 randomization ratio and various true VT-IPD VE, Table 28 shows the total number of first evaluable VT-IPD episodes needed to demonstrate VT-IPD VE > 0% with 80 or 90% power (one-sided test, nominal type I error of 2.5%).

Table 28 Total number of first episodes required to show VT-IPD VE > 0% with 80% and 90% power, according to various true VE

True VE	Required first episodes of VT-IPD*	
	80% power	90% power
60%	47	61
65%	37	47
70%	30	37
75%	23	30
80%	17	23
85%	15	17
90%	12	15
95%	9	9

*PASS 2005 - one-sided test for one proportion, nominal type I error of 2.5%

9.3.2. Carriage objective in a subset of subjects

The efficacy of the 10Pn-PD-DiT vaccine in reducing nasopharyngeal carriage of *S. pneumoniae* (vaccine serotypes) and *H. influenzae* will be evaluated as secondary objective in a subset of 2000 subjects in Panama (1000 subjects per group).

Considering a 1:1 randomization ratio and various true VE, Table 29 shows the power to detect group difference (i.e. LL of 95% CI for VE > 0%) based on fixed percentage of

subjects with positive cultures (based on the number of subjects with collected cultures) in the control group at 18 months of age (one-sided test, nominal type I error of 2.5%).

Table 29 Power to detect group difference in carriage of *S. pneumoniae* (vaccine serotypes) and *H. influenzae* based on fixed percentage of subjects with positive cultures (%) in Control group and various true VE (N= 1000 subjects/group)

Carriage endpoint	% in Control group *	VE	% in 10Pn-PD-DiT group	Power **
VT S.pneumoniae	17.8%	25%	13.4%	76.7%
		30%	12.5%	90.6%
		35%	11.6%	97.3%
		40%	10.7%	99.5%
		45%	9.8%	99.9%
H. influenzae	28.7%	25%	21.5%	95.6%
		30%	20.1%	99.4%
		35%	18.7%	99.9%
		40%	17.2%	100%
		45%	15.8%	100%

* References: "Nasopharyngeal Carriage of Streptococcus pneumoniae in a Cohort of Healthy, Chilean New Born Infants Followed from Age 0 to 24 months" - Lagos R. / Nasopharyngeal Colonisation with *S. pneumoniae* and/or *H. influenzae*, in a cohort of Chilean infants
** PASS 2005 - Power using 2 independent proportions, 1:1 randomization ratio, one sided test to detect group difference, alpha=2.5%

9.3.3. Safety objective

The safety profile of the 10Pn-PD-DiT vaccine when co-administered with DTPa-based combination vaccines with respect to the occurrence of serious adverse events (SAE) collected for all planned 24,000 enrolled subjects (12,000 per group) throughout the entire study period will be evaluated as secondary objective.

The analysis will estimate and compare the percentage of subjects with any SAEs, overall and according to MedDRA primary terms between 10Pn-PD-DiT and Control groups, using 95% CI for group difference and one-sided nominal p-value for the null hypothesis of no group difference. Potential safety signals will be based on nominal p-value below 2.5%.

Practically, based on standardized asymptotic p-value, a statistically significant difference will be observed when 4 subjects in one group develop a specific SAE compared to 0 subjects in the other group (or 7 compared to 1), whatever the number of subjects (greater than 50) enrolled in each group in a 1:1 ratio.

Considering the multiplicity of comparisons and the exploratory nature of the evaluation, the risk of false safety signals is much higher than 2.5%. Therefore, any signal will be further examined for clinical plausibility and relevance.

Table 30 shows that a doubling in rare SAE following 10Pn-PD-DiT+DTPa-based combination vaccines will be detected with more than 80% power for any event with an incidence of at least 0.2% in the Control group.

Table 30 Probability to detect/rule out an increase in the percentage of subjects with rare SAE

Incidence of rare SAE in Control group	Probability that lower limit of the 95% CI for the absolute difference in incidence is below Δ considering that 10Pn-PD-DiT group has same incidence as Control group (Pass 2005 - one-sided equivalence test on difference in proportions, alpha=2.5%)				Incidence in 10Pn-PD-DiT group that will lead to a statistically significant increase (one sided p-value < 2.5%, 80% power, PASS 2005 - 2 independent proportions-arcsine and continuity correction)
	Δ=0.1%	Δ=0.2%	Δ=0.5%	Δ= 1%	
0.10%	62%	99%	>99%	>99%	0.26%
0.20%	39%	91%	>99%	>99%	0.40%
0.50%	19%	58%	>99%	>99%	0.80%
1.0%	12%	34%	97%	>99%	1.40%

* The expected rate of rare serious adverse event reported throughout the entire study period is around 0.1-0.2% (reference 347414/010 (Undeca-Pn-010) – POET study).

9.4. Study cohorts to be evaluated

Total Vaccinated cohort

The total vaccinated cohort will include all vaccinated subjects. Thus, the total vaccinated cohort for analysis of safety will include all subjects with at least one vaccine administration documented and the total vaccinated cohort for analysis of efficacy/immunogenicity will include vaccinated subjects for whom data concerning efficacy/immunogenicity endpoint measures are available. The total vaccinated cohort analysis will be performed per treatment actually administered.

According-To-Protocol (ATP) cohort for analysis of efficacy

The ATP cohort for analysis of efficacy will include all evaluable subjects (i.e. those meeting all eligibility criteria, complying with the procedures and intervals defined in the protocol, with no elimination criteria during the study) for whom data concerning efficacy endpoint measures are available. This will include subjects having received at least three primary doses of study vaccine, with available contact beyond day 14 post study vaccine dose 3.

The intervals between study visits that will be considered for inclusion in the ATP cohort for analysis of efficacy will be 35 to 97 days between vaccination visits 1 and 2 and visits 2 and 3, although the investigator is requested to respect as much as possible the intervals specified in Table 6 (Section 5.4.1).

According-To-Protocol (ATP) cohort for analysis of safety

The ATP cohort for analysis of safety will include all subjects:

– who have received at least one dose of study vaccine/control according to their random assignment,

– for whom administration route of study vaccine /control is known,

– who have not received a vaccine not specified or forbidden in the protocol,

– for whom the randomization code has not been broken

According To Protocol (ATP) cohort for analysis of immunogenicity

The ATP cohort for analysis of immunogenicity will include all evaluable subjects (i.e. those meeting all eligibility criteria, complying with the procedures and intervals defined in the protocol, with no elimination criteria during the study) for whom data concerning immunogenicity endpoint measures are available. This will include subjects for whom assay results are available for antibodies against at least one study vaccine antigen component after vaccination.

The intervals between study visits that will be considered for inclusion in the ATP cohort for analysis of immunogenicity will be 35 to 97 days between vaccination visits 1 and 2 and between vaccination visits 2 and 3 and will be 28 to 48 days between third vaccination at visit 3 and blood sampling at visit 4 and between booster dose at visit 6 and post-booster blood sampling at visit 7, although the investigator is requested to respect as much as possible the intervals specified in Table 8 (Section 5.4.2).

9.5. Derived and transformed data

- The cut-off values for the assays used for evaluation of the immune responses are defined by the laboratory before the analysis and are described in Section 5.7.
- A seronegative subject is a subject whose concentration/titer is below the cut-off value.
- A seropositive subject is a subject whose concentration/titer is greater than or equal to the cut-off value.
- The Geometric Mean Concentrations/Titers (GMCs/GMTs) calculations are performed by taking the anti-log of the mean of the log concentration/titer transformations. Antibody concentrations/titers below the cut-off of the assay will be given an arbitrary value of half the cut-off for the purpose of GMC/GMT calculation.
- Handling of missing data:
  - For a given subject and a given efficacy/immunogenicity measurement, missing or non-evaluable measurements will not be replaced. Therefore, an analysis will exclude subjects with missing or non-evaluable measurements.
  - For the analysis of solicited symptoms, missing or non-evaluable measurements will not be replaced. Therefore the analysis of the solicited symptoms based on

the Total Vaccinated cohort will include only subjects/doses with documented safety data (i.e. symptom screen/sheet completed).

– For the analysis of unsolicited adverse events/serious adverse event/concomitant medication, all vaccinated subjects will be considered and subjects who did not report an event will be considered as subjects without an event.

9.6. Final analyses (at study end: see definition in Section 3.1)

In order to keep the blinding, any analysis of immunogenicity or carriage before the final analysis will be performed by an independent statistician.

For sequence of analysis refer to section 9.7.1.

(Amended 09 September2010)

9.6.1. Analysis of demographics/baseline characteristics

For each group, demographic characteristics (age, gestational age, gender, race and risk factors for pneumococcal infections) of each study cohort will be tabulated; the mean age at each vaccine dose (plus range and standard deviation) will be calculated and the distribution of subjects enrolled among the study countries/centres will be tabulated.

9.6.2. Analysis of efficacy

9.6.2.1. Case accountability

Preliminary remark:the counting rules specified in this section do not modify the reporting procedures for CAP, AOM and ID cases. These counting rules will be applied at the time of the statistical analysis of the study results. Individual episodes should still be reported by the investigator even if according to the counting rule it appears that the case might not be considered to be a new episode.

9.6.2.1.1. Rules for CAP cases

All suspected CAP cases occurring during the entire study must be reported as described in section 5.5.2.1.2.

For CAP, the start date of a clinical episode corresponds to the date when symptoms first were observed. In case this date is not known or defined, the date when the CXR was taken will be considered to be the start date. From an analysis point of view, a new CAP episode will be considered to have started if 30 days or more have elapsed since the start date of the previous CAP episode. If less than 30 days have elapsed since the start date of the previous CAP episode, the new CXR will be considered related to the same CAP episode. If for the same CAP episode, the outcome of consecutive CXR readings evolve over time, the most serious diagnosis will be taken into consideration.

9.6.2.1.2. Rules for AOM cases

- All clinical episodes of OM will be reported, including new episodes in a same subject.
- In children with ventilating tubes, tube-related otorrhea is considered as an episode of OM.
- The start date of an episode is the date of the visit during which the diagnosis is confirmed by the ENT specialist.
- If an AOM visit occurs within 30 days after the start date of a previous AOM episode, it will be considered as a part of the same clinical episode but could eventually, according to the bacterial culture results, be considered as part of the same or as a new bacterial episode:
  - if the latter visit is associated with the same bacterial pathogen and serotype as the one isolated during the previous AOM visit or if no bacterial pathogen could be isolated, the visit will be considered as part of the same bacterial episode
  - if a different bacterial pathogen or a different serotype is isolated during the latter visit than the one isolated during the previous AOM visit, the visit will be considered as the start of a new bacterial episode
- From an analysis point of view, if an AOM visit occurs at least 30 days after the date of confirmation by the ENT specialist of a previous AOM episode, it will be considered as a new episode.
- If a mixed flora is identified in the MEF of a child presenting with AOM (e.g., Streptococcus pneumoniae and NTHi, Streptococcus pneumoniae and Moraxella catarrhalis, or two different pneumococcal serotypes) it will be considered as a single clinical episode, but as separate bacterial episodes (one per isolated bacteria).
- Bilateral AOM with two different bacterial pathogens or serotypes is considered as one clinical episode and one bacterial episode, but as 2 episodes when looking to each bacterial pathogen.

9.6.2.1.3. Rules for ID cases

From an analysis point of view, if the same pneumococcal or H. influenzae serotype is isolated with an interval of less than 30 days, both isolates will be considered related to the same invasive disease episode. If the pneumococcus or H. influenzae isolate does not share the same serotype, or if 30 days or more have elapsed since previous isolation, it will be considered as a new invasive disease episode.

9.6.2.2. Analysis of primary objective

9.6.2.2.1. According-to-protocol (ATP) analysis = primary analysis

For the ATP cohort, the first analysis of efficacy will be based on the occurrence of the primary endpoint (i.e., first episodes of likely bacterial CAP) anytime from two weeks after the administration of the third dose of the study vaccine.

The VE as compared to the control group will be evaluated using a Cox regression model on the time starting from two weeks after the administration of the third dose of the study vaccine up to the first B-CAP episode. Censoring will occur at the time of the last scheduled and unscheduled contact. For subjects who have more than 18 months of age without having received the booster dose, an episode of B-CAP will be censored at 18 months of age.

VE= (1 – hazard ratio) x 100

All subjects from the ATP cohort will contribute to the comparison.

The Cox model, including the treatment group as the only regressor, will be used to derive 95% CI for the VE.

In order to check the statistical significance, one-sided p-values for the Wald-Test obtained from the Cox proportional hazard model will be calculated. ***Nominal type I error for the interim analysis will be 1.75%.*** Nominal type I error for the final analysis will be computed according to the final number of first episodes to maintain the 2.5% type I error. ~~Assuming this is still 1.19%, the success of the primary objective will be established if the one-sided p-value calculated for the null hypothesis H0=[B-CAP VE ≤ 0%] is lower than 1.19%.~~ **(Amended 09 September2010)**

Note that cumulative hazard curves will be used to display the distribution of time to B-CAP episode, for each group.

9.6.2.2.2. Total vaccinated cohort analysis

For the total cohort, the analysis of efficacy will be based on the occurrence of the primary endpoint (i.e., first episodes of B-CAP) anytime from the time of first vaccination. Subjects without booster dose will not be censored (i.e. all episodes will be included in the analysis).

VE and 95% CI will be computed the same way as for the primary analysis on ATP cohort.

9.6.2.3. Analysis of secondary efficacy objectives

The number of episodes (first and new episodes as defined in Section 5.5.2), follow-up days, and associated rate will be presented by group, for each endpoint.

Based on the first episode of each endpoint, a 95% CI will also be constructed around the VE, as for the primary endpoint.

In order to check the statistical significance of the first secondary objective (C-AOM) one-sided p-value for the Wald-Tests obtained from the Cox proportional hazard model will be calculated. The success of the first secondary objective will be established if the primary objective has been reached and if the one-sided p-value calculated for the null hypothesis H0=[C-AOM VE ≤ 0%] will be lower than 2.5%.

From an exploratory point of view, in order to check the statistical significance of the next secondary objectives (B-AOM, C-CAP and VT-IPD), one-sided p-value for the Wald-Tests obtained from the Cox proportional hazard model will be calculated. ***Next secondary objectives will be reached*** ~~The success of a secondary objective will be established~~ if the primary and first secondary objectives ~~has~~ ***have*** been reached and if the one-sided p-value calculated for the null hypothesis H0=[VE ≤ 0%] will be lower than 2.5%. **(Amended 09 September2010)**

Finally, for AOM and CAP endpoints, VE and its 95% CI for the time to occurrence of any episodes (first and new episodes) of a specific event during the considered efficacy follow-up will also be estimated using the Andersen & Gill model (generalization of the Cox proportional hazard model) taking into account all episodes.

Change in VE over time will be investigated using Spline-smoothed estimates of hazard ratio and 95% confidence bands.

The vaccine-attributable reduction (VAR) per 1000 child-years, defined as the incidence in the control group minus the incidence in the 10Pn group will also be computed with its 95% CI. **(Amended 09 September2010)**

In addition, descriptive analysis to evaluate whether vaccine efficacy is influenced by the severity of the disease could be done to complement the primary endpoint results.

9.6.3. Analysis of carriage

‘CARRIAGE’ SUBSET ONLY

As planned on a second subset of enrolled subjects, according to availability of final and complete laboratory results, carriage analysis as described in this section could be done in a stepwise approach before the availability of final data related to other endpoints (efficacy, safety, etc.). In order to keep the blinding, any analysis of carriage before final analysis will be performed by an independent statistician. **(Amended 09 September2010)**

Positive cultures of *S. pneumoniae* serotypes and *H. influenzae* identified in the nasopharynx will be analysed for the total vaccinated cohort, from a descriptive point of view:

- Percentage of subjects with positive nasopharyngeal sample will be calculated per group, at each swab time-point.
- Vaccine efficacy estimated as [(1-Relative Risk)*100] with 95% CI will also be calculated for carriage of *S. pneumoniae* and *H. influenzae.*
- Frequency of acquisition of new bacteria /serotypes in the nasopharynx will be evaluated per group, at each swab time-point.

9.6.4. Analysis of antibiotic treatment

‘CARRIAGE’ SUBSET ONLY

Analysis will be descriptive:

-Percentage of subjects receiving at least one antibiotic treatment during the study period starting from visit 1 up to visit 9 will be calculated per group, with 95% CI. Difference between groups (10Pn-PD-DiT group minus control group) of this percentage will also be calculated with 95% CI.

-Incidence of antibiotic treatment per person years of follow-up will also be computed with 95% CI.

9.6.5. Analysis of immunogenicity

'IMMUNO AND REACTO' SUBSET ONLY

As planned on a subset of first enrolled subjects, according to availability of final and complete laboratory results, immunogenicity analysis as described in this section could be done in a stepwise approach before the availability of data related to other endpoints (efficacy, safety etc.). In order to keep the blinding ~~regarding efficacy endpoints~~, any analysis of immunogenicity before final analysis will be performed by an independent statistician.

The primary analysis will be based on the ATP cohort for analysis of immunogenicity. If the percent of enrolled subjects excluded from this ATP cohort is more than 5%, a second analysis based on the Total Vaccinated cohort will be performed to complement the ATP analysis.

The analysis will be descriptive, across and per country.

Where appropriate, for each group, at each time-point that a blood-sample result is available:

- GMCs/GMTs with 95% CIs will be tabulated for each serotype/antigen.
- Seropositivity rates with exact 95% CIs will be calculated for each appropriate serotype/antigen.
- The distribution of antibody concentrations/titers for each serotype/antigen will be displayed using tables and/or reverse cumulative distribution curves.
- For each pneumococcal serotype, at each time-point that a blood-sample result is available, geometric mean of ratios of Opsonophagocytic titres/ELISA concentrations will also be tabulated with 95% CIs.

9.6.6. Analysis of safety

ALL SUBJECTS

Serious adverse events and withdrawal due to serious adverse event(s)/adverse event(s) will be described in detail.

95%CI for group difference (10Pn-PD-DiT group minus Control group) in the percentage of subjects with specific SAEs, overall and according to MedDRA primary terms and one-sided nominal p-value for the null hypothesis of no group difference will be computed.

'IMMUNO AND REACTO' SUBSET ONLY FOR SOLICITED AEs AND SUBJECTS FROM PANAMA ONLY FOR UNSOLICITED AEs

Solicited adverse events will only be analysed in the defined 'Immuno and reacto' subset and unsolicited AEs will be analysed in subjects enrolled in Panama. The analysis will be descriptive, overall and per country.

The primary analysis will be based on the total vaccinated cohort. If the percentage of enrolled subjects excluded from the ATP cohort for analysis of safety is more than 5%, a second analysis based on this ATP cohort will be performed to complement the total analysis.

The percentage of subjects with at least one local adverse event (solicited and unsolicited), with at least one general adverse event (solicited and unsolicited) and with any adverse event during the 31-day (day 0 – day 30) follow-up period will be tabulated with exact 95% CI after each vaccine dose and overall primary vaccination course. The percentage of doses followed by at least one local adverse event (solicited and unsolicited), by at least one general adverse event (solicited and unsolicited) and by any adverse event will be tabulated, over the full primary vaccination course, with exact 95% CI. The same calculations will be performed for symptoms rated as grade 3.

The percentage of subjects reporting each individual solicited local and general adverse event during the 4-day (day 0-day 3) solicited follow-up period will be tabulated after each vaccine dose and overall primary vaccination course, with exact 95% CI. The percentage of doses followed by each individual solicited local and general adverse event will be tabulated, over the full primary vaccination course, with exact 95% CI.

Occurrence of fever and related fever will be reported per 0.5°C cumulative increments. For all other solicited symptoms, the same tabulation will be added for grade 3 adverse events and for adverse events with relationship to vaccination. In addition, for redness and swelling, grade 2 or 3 adverse events will be tabulated.

Large swelling reactions following booster dose will also be described.

The proportion of subjects/doses with at least one report of unsolicited adverse event classified by the Medical Dictionary for Regulatory Activities (MedDRA) and reported up to 30 days after primary vaccinations or booster dose will be tabulated with exact 95%

CI. The same tabulation will be performed for grade 3 unsolicited adverse events and for unsolicited adverse events with a relationship to vaccination. In addition, the proportion of subjects/doses with at least one report of unsolicited adverse events from visit 1 up to visit 9 (24-27 months of age) will be tabulated.

The proportion of AEs resulting in a medically attended visit will also be tabulated.

95% CI for group difference (10Pn-PD-DiT group minus control group) in the percentage of subjects with unsolicited AEs, from visit 1 up to visit 9, overall and according to MedDRA primary terms and one-sided nominal p-value for the null hypothesis of no group difference will be computed.

9.7. Planned interim analysis

9.7.1. Sequence of analysis.

B-CAP interim analysis will be conducted as described in section 9.7.2

If this interim analysis is conclusive (=primary objective is met):

- ***Additional analyses will be performed by the Independent Statistician before study end in a stepwise approach according to availability of required data:***
  - ***ATP vaccine efficacy analysis on defined secondary endpoints related to CAP.***
  - ***Partial analysis of demography characteristics (age, gender and race).***
  - ***Final analysis of immunogenicity as described in section 9.6.5.***
  - ***Final analysis of carriage as described in section 9.6.3.***
- ***Remaining analyses will be prepared by GSK at study end on cleaned and final data, including:***
  - ***Complementary analysis of CAP including the additional CAP reported from the interim analysis to study end.***
  - ***Final analysis of AOM and ID.***
  - ***Final analysis of safety.***
  - ***Final analysis of antibiotic treatment.***

If this interim analysis is NOT conclusive:

- ***The study end for a subject will be organized approximately between September and December 2011. All the analyses described in section 9.6 will be generated on cleaned and final data.***

9.7.2. Consideration for the interim analysis

An interim efficacy analysis will be performed to evaluate the efficacy to prevent the first episode of B-CAP (primary endpoint). The interim analysis will be used to determine whether positive VE can be detected earlier than anticipated based on a higher VE (interim analysis for early final conclusion).

The interim analysis will be performed when we will reach at least **535 first B-CAP episodes** and will be performed **for subjects in the ATP cohort.** In addition, a minimum study duration of 18 months after study start will be respected in order to allow a clinically meaningful duration of follow-up in a sufficient number of subjects

The interim analysis to evaluate the efficacy to prevent the first episode of B-CAP will be analyzed according to the methodology described in Section 9.6.2.2. The interim analysis will be performed by a statistician independent of the project and GSK Biologicals (i.e. from a contract research organization). Project staff within GSK Biologicals and investigator will remain blinded to the randomization codes until final analysis.

In order to check the statistical significance at the time of this interim analysis, one-sided p-values for the Wald-Tests obtained from the Cox proportional hazard model will be calculated. The success of the primary objective will be established if the one-sided nominal p-value calculated for the null hypothesis H0=[B-CAP VE ≤ 0%] is lower than 1.75%.

The results of the interim analysis will be sent to the IDMC. The IDMC will review the results and will communicate to GSK if the primary endpoint has been met. Upon request by GSK, this conclusive result (nominal p-value < 1.75%) will be shared by IDMC. In case the primary endpoint has been met, GSK might request to analyse secondary endpoints related to CAP ***and other endpoints as described in the section 9.2***. This analysis will also be performed by a statistician independent of the project and GSK Biologicals. The results will be sent to the IDMC and communicated to GSK.

Regardless of the results of the interim analysis, the study will continue up to study end. Results at the interim analysis will not be changed based on subsequent analyses. The analyses at study end will be descriptive and will complement the first analysis by providing more accurate VE.

10. ADMINISTRATIVE MATTERS

To comply with Good Clinical Practice important administrative obligations relating to investigator responsibilities, monitoring, archiving data, audits, confidentiality and publications must be fulfilled. See Appendix B for details.

11. REFERENCES

American Academy of Pediatrics, Committee on Infectious Diseases. Policy statement: recommendations for the prevention of pneumococcal infections, including the use of pneumococcal conjugate vaccine (Prevnar), pneumococcal polysaccharide vaccine, and antibiotic prophylaxis.Pediatrics 2000; 106 (2); 362-6.

Appelbaum PC, Gladkova C, Hryniewicz W et al. Carriage of antibiotic-resistant Streptococcus pneumoniae by children in Eastern and Central Europe - A multicenter study with use of standardized methods. Clin Infect Dis 1996;23:712-717.

Arguedas A, Dagan R, Soley C et al. Microbiology of otitis media in Costa Rican children, 1999 through 2001. Ped Infect Dis J 2003; 22:1063-8.

Arguedas A, Dagan R, Guevara S et al. Middle ear fluid *Streptococcus pneumoniae* serotype distribution in Costa Rican children with otitis media. Ped Infect Dis J 2005; 24: 631-634.

Austrian R. The quellung reaction, a neglected microbiologic technique. Mt Sinai J Med 1976; 43:1053-1059.

Bakaletz LO, Kenedy B-J, Novotny LA et al. Protection against development of otitis media induced by non-typeable *Haemophilus influenzae* by both active and passive immunization in a chinchilla model of virus-bacterium superinfection. Infect Immun 1999; 67: 2746-62.

Bergeson PS, Singer SA, Kaplan AR. Intramuscular injections in children. Pediatrics 1982; 70: 944-948.

Berman S. Otitis media in children. N Engl J Med 1995; 332: 1560-5.

Black S, Shinefield H, Fireman N et al. Efficacy, safety and immunogenicity of heptavalent pneumococcal conjugate vaccine in children. Pediatric Infect Dis J 2000;19:187-195.

Black B, Shinefield H, Ling S et al. Effectiveness of heptavalent pneumococcal conjugate vaccine in children younger than five years of age for prevention of pneumonia. Pdiatric Inf Dis J 2002; 21: 810-815

Bluestone C: Clinical course, complications and sequelae of acute otitis media. Pediatr Infect Dis J 2000; 19: S37-46

Butler JC, Hofmann J, Cetron MS et al. The continued emergence of drug-resistant *Streptococcus pneumoniae* in the United States: an update from the Centers for Disease Control and Prevention’s Sentinel Surveillance System. J Infect Dis 1996;271:1831-1835.

Casetta A, Derouin V, Boussougant Y. Absence of spontaneous autolysis of Streptococcus pneumoniae in aerobic fan culture bottles in a commercial blood culture system. Eur J Clin Microbiol Infect Dis 1996;15: 616-617.

Centers for Disease Control and Prevention. 2002. Active Bacterial Core Surveillance Report, Emerging Infections Program Network, *Streptococcus pneumoniae*, 2002. Available via the Internet: http://www.cdc.gov/ncidod/dbmd/abcs/survreports/spneu02.pdf (accessed January 25, 2004).

Centers for Disease Control and Prevention. Recommended Childhood and Adolescent Immunization Schedule—United States, 2004. MMWR 2004;53:Q1–4.

Cohen R, Levy C, Boucherat M et al. Enquête épidémiologique sur l'AOM en pratique de ville. Médecine et Enfance 1996: 27-31.

Concepcion N, Frasch C. Pneumococcal type 22F polysaccharide absorption improves the specificity of a pneumococcal-polysaccharide enzyme-linked immunosorbent assay. Clin Diag Lab Immun. 2001; 8: 266-272.

Cutts FT, Zaman SMA, Enwere G et al. Efficacy of nine-valent pneumococcal conjugate vaccine against pneumonia and invasive pneumococcal disease in The Gambia: randomized, double-blind, placebo-controlled trial. Lancet 2005 365: 1139-1146.

Dagan R et al. Conjugate vaccines: potential impact on antibiotic use? Int J Clin Practice 2001;Supplement 118:27-28.

Dintzis RZ. Rational design of conjugate vaccines. Pediatric Research 1992;32:376-385.

Eskola J, Kilpi T, Palmu A, et al. Efficacy of a pneumococcal conjugate vaccine against acute otitis media. NEJM 2001; 344/6:403–409.

François M. Efficacité et tolerance d'une application locale de phenazone et de chlorhydrate de lidocaine (Ootipax) dans les otites congestives du nourisson et de l'enfant. Ann Pediatr 1993: 40: 481-4.

Friedman NR, McCormick DP, Pittman C, Chonmaitree T et al. Development of a practical tool for assessing the severity of Acute Otitis Media. Pediatr Infect Dis J 2006; 25(2):101-107.

García-Rodríguez JA and Fresnadillo Martínez MJ. Dynamics of nasopharyngeal colonization by potential respiratory pathogens. J of Antimicrobial Chemotherapy 2002; 50, Suppl S2, 59-73.

Ghaffar F, Friedland I, McCracken G. Dynamics of nasopharyngeal colonization by *Streptococcus pneumoniae*. Pediatric Infect Dis J 1999;18/7:638-646.

Giebink GS, Canafax DM, Kempthorne J. Antimicrobial treatment of acute otitis media J Pediatr 1991; 119: 495-500.

Goldstein G, Giroir B, Randolph A et al. International pediatric sepsis consensus conference: definitions for sepsis and organ dysfunction in pediatrics. Pediatr Crit Care Med vol 6; pp. 2-8, 2005

Hansen J, Black S, Shinefield H et al. Effectiveness of heptavalent pneumococcal conjugate vaccine in children younger than 5 years of age for prevention of pneumonia. Updated analysis using World Health Organization standardized interpretation of chest radiographs. Ped Infect Dis J 2006; 25 (9): 779-781.

Hausdorff WP, Bryant J, Paradiso PR and Siber GR. Which pneumococcal serogroups cause the most invasive disease: implications for conjugate vaccine formulation and use, part I. Clin Infect Dis 2000;30:100-21.

Hausdorff W, Siber G, Paradiso P. Geographical differences in invasive pneumococcal disease rates and serotype frequency in young children. Lancet 2001;357:950-952

Hausdorff W, Yothers G, Dagan R et al. Multinational study of pneumococcal serotypes causing acute otitis media in children. Ped Infect Dis J 2002; 21: 1008-16.

Henckaerts I, Goldblatt D, Ashton L, Poolman J. Critical differences between pneumococcal polysaccharide-enzyme-linked immunosorbent assays with and without 22F inhibition at low antibody concentrations in pediatric sera. Clin and Vacc Immunol 2006: 356-60.

Henckaerts I, Durant N, De Grave D et al. Validation of a routine opsonophagocytosis assay to predict invasive pneumococcal disease efficacy of conjugate vaccine in children. Vaccine 2007; 25(13): 2518-2527

Henrichsen H. Six newly recognized types of *Streptococcus pneumonia*e. J Clin Microbiol 1995;33:2759-2762.

Hoberman A, Paradise JL, Wald ER. Tympanocentesis technique revisited. 1997. Pediatr Infect Dis J. 16: S25-S26.

Isenberg. Essential procedures for clinical microbiology. American Society of Microbiology (ASM), Washington DC, 1998, pp. 477.

Klein JO. Otitis media. Clin Infect Dis 1994; 19: 823-33.

Klein D. Pneumococcal disease and the role of conjugate vaccines. Microbial Drug Resistance 1999;4/2:147-157.

Klein JO. The burden of otitis media. Vaccine 2001;19:S2-S8.

Klugman KP, Madhi SA, Huebner RE, et al. A Trial of a 9-Valent Pneumococcal Conjugate Vaccine in Children with and Those without HIV Infection. N Engl J Med 2003; 349: 1341-1348.

Levine M, Lagos R, Levine O et al. Epidemiology of invasive pneumococcal infections in infants and young children in Metropolitan Santiago, Chile, a newly industrialized country. Pediatr Infect Dis J 1998; 17: 287-93.

Madhi SA, Heera JR, Kuwanda L, Klugman KP. Use of procalcitonin and C-reactive protein to evaluate vaccine efficacy against pneumonia. PLoS Medicine 2005a 2 (e38): 147-151.

Madhi S, Kuwanda L, Cutland C, Klugman K. The impact of a 9-valent pneumococcal conjugate vaccine on the public health burden of pneumonia in HIV-infected and -uninfected children. Clin Infect Dis 2005b; 40(10): 1511-18.

Madhi SA, Kohler M, Kuwanda L, Cutland C, Klugman K. Usefulness of C-reactive protein to define pneumococcal conjugate vaccine efficacy in the prevention of pneumonia. Ped Infect Dis J 2006; 25: 30-36.

Madhi S.A. World Health Organisation definition of “radiologically-confirmed pneumonia” may underestimate the true public health value of conjugate pneumonia vaccines. Vaccine 2007: 25(13): 2413-2419.

Manual of Clinical Microbiology. Sixth edition, American Society of Microbiology, Murray PR et al (Washington DC). 1995.

Martindale: The Complete Drug Reference 1999;1527.

McCaig LF, Hughes JM. Trends in antimicrobial drug prescribing among office-based physicians in the United States. JAMA 1995: 273(3): 214-9.

McCormick AW, Whitney CG, Farley MM et al. Geographic diversity and temporal trends of antimicrobial resistance in *Streptococcus pneumoniae* in the United States. Nature Medicine 2003; 9: 424-430.

Mitchell TJ, Alexander JE, Morgan PJ and Andrew PW. Molecular analysis of virulence factors of *Streptococcus pneumonia*e. J. Applied Microbiology Symposium Supplement 1997; 83:625-715.

Mulholland K. Strategies for the control of pneumococcal diseases. Vaccine 1999;17:S79-S84.

Mulholland EK. Use of vaccine trials to estimate burden of disease. J Health Popul Nutr 2004;22(3): 257-267.

Ministerio de Salud de la Nación del Argentina. Normas Nacionales de Vacunacion, Edición 2008.

Obaro S and Madhi S. Bacterial pneumonia vaccines and childhood pneumonia: are we winning, refining or redefining? Lancet Infect Dis 2006; 6: 150-61.

O'Brien KL, Moulton LH, Reid R, et al. Efficacy and safety of seven-valent conjugate pneumococcal vaccine in American Indian children: group randomised trial. Lancet 2003; 362: 355-361.

O'Brien K, Nohynek H, the WHO Pneumococcal Vaccine Trials Carriage Working Group. Report from a WHO Working Group: standard method for detecting upper respiratory carriage of *Streptococcus pneumoniae*. Pediatr Infect Dis J 2003; 22(2):133-140.

Pichichero ME. Changing the treating paradigm ofor acute otitis media in children. JAMA 1998a; 279: 1748-50.

Pichichero ME. Changing the treating paradigm for acute otitis media in childrn. JAMA 1998b; 280: 1904.

Pichichero ME. Acute Otitis Media: Part I. Improving diagnostic accuracy. American Health Physician 2000a; 61(7): 2051-56.

Prymula R, Peeters P, Chrobok V, et al. Pneumococcal capsular polysaccharides conjugated to protein D provide protectin against otitis media caused by both *Streptococcus pneumoniae* and non-typable *Haemophilus influenzae*. Lancet 2006; 367: 740-48.

Quataert SA, Kirch CS, Quackenbush Wiedl LJ, Phipps DC, Strohmeyer, Cimino CO, et al. Assignment of weight-based antibody units to a human antipneumococcal standard reference serum, lot 89-S. Clinical and Diagnostic Laboratory Immunology 1995; 2: 590-597.

Romero-Steiner S, et al. Standardization of an opsonophagocytic assay for the measurement of functional antibody activity against *Streptococcus pneumoniae* using differentiated HL-60 cells. *Clin Diagn Lab Immunol* 1997;4(4): 415-422.

Rosenblüt A, Santolaya ME, González P, Corbalán V, Avendaño LF, Martínez MA, Hormazabal JC. Bacterial and viral etiology of acute otitis media. Ped Infect Dis J 2001, 20(5): 501-507.

Rosenfeld RM. Diagnostic certainty for acute otitis media. Int J Pediatr Otorhinolaryngol 2002; 64: 89-95.

Teele DW, Klein JO, Rosner B. Epidemiology of otitis media during the first seven years of life in children in greater Boston : a prospective, cohort study. J Infect Dis1989;160:83-94.

Tregnaghi M, Ceballos A, Ruttimann R, Ussher J, Tregnaghi P, Peeters P, Hausdorff WP. Active epidemiologic surveillance of pneumonia and invasive pneumococcal disease in ambulatory and hospitalized infants in Cordoba, Argentina. Pediatr Infect Dis J 2006; 25(4):370-372

UNICEF. Pneumonia: The forgotten killer of children. The United Nations Children's Fund (UNICEF)/World Health Organization (WHO), 2006.

Van Horn K, Tóth C, Wegienek J. Viability of microorganisms in four swab systems. ASM 98th General Meeting. 1998: C436.

Vidar. Vidar Twain User's Guide 5.2. 2005. 63 p. Available via the Internet: www.filmdigitizer.com

Wardlaw T, Salama P, White Johansson E et al. Pneumonia: the leading killer of children. Lancet 2006; 368: 1048-50.

Whitney CG, Farley MM, Hadler J et al. Increasing prevalence of multidrug-resistant *Streptococcus pneumoniae* in the United States. NEJM 2000; 343: 1917-1924.

WHO. Recommendations for the production and control of pneumococcal conjugate vaccines (Annex 2). WHO Technical Report Series, No 927, 2005.

WHO. Standardization of interpretation of chest radiographs for the diagnosis of pneumonia in children. World Health Organization, Geneva, 2001.

World Health Organization (WHO). Technical bases for the WHO recommendations on the management of pneumonia in children at first-level health facilities. World Health Organization, Geneva; 1991.

World Health Organization (WHO). Acute respiratory infections in children: Case management in small hospitals in developing countries. A manual for Doctors and other Senior Health Workers. World Health Organization, Geneva; 1994.

Wold Health Organization (WHO). Manual of diagnostic imaging, radiographic techniques and projections. World Health Organization, Geneva, 2003.

Christ-Crain M, Jaccard-Stolz D et al. Effect of procalcitonin-guided treatment on antibiotic use and outcome in lower respiratory tract infections: Cluster-randomized, single-blinded intervention trial. Lancet 2004, 363: 600-607.

Simon L, Gauvin F, Amre DK et al. Serum procalcitonin and C-reactive protein levels as markers of bacterial infection: A systematic review and meta-analysis. Clinical Infect Dis 2004, 39: 206-217.

CHMP. Note for guidance on clinical evaluation of new vaccines. European Medicine Evaluation Agency, May 2005. 23 p.

Appendix A World Medical Association Declaration of Helsinki

Recommendations guiding physicians
in biomedical research involving human subjects

Adopted by the 18th World Medical Assembly
Helsinki, Finland, June 1964

and amended by the
29th World Medical Assembly
Tokyo, Japan, October 1975
35th World Medical Assembly
Venice, Italy, October 1983
41st World Medical Assembly
Hong Kong, September 1989
and the
48th General Assembly
Somerset West, Republic of South Africa, October 1996

INTRODUCTION

It is the mission of the physician to safeguard the health of the people. His or her knowledge and conscience are dedicated to the fulfilment of this mission.

The Declaration of Geneva of the World Medical Association binds the physician with the words, "The health of my patient will be my first consideration," and the International Code of Medical Ethics declares that, "A physician shall act only in the patient's interest when providing medical care which might have the effect of weakening the physical and mental condition of the patient."

The purpose of biomedical research involving human subjects must be to improve diagnostic, therapeutic and prophylactic procedures and the understanding of the etiology and pathogenesis of disease.

In current medical practice most diagnostic, therapeutic or prophylactic procedures involve hazards. This applies especially to biomedical research.

Medical progress is based on research which ultimately must rest in part on experimentation involving human subjects.

In the field of biomedical research a fundamental distinction must be recognized between medical research in which the aim is essentially diagnostic or therapeutic for a patient, and medical research, the essential object of which is purely scientific and without implying direct diagnostic or therapeutic value to the person subjected to the research.

Special caution must be exercised in the conduct of research which may affect the environment, and the welfare of animals used for research must be respected.

Because it is essential that the results of laboratory experiments be applied to human beings to further scientific knowledge and to help suffering humanity, the World Medical Association has prepared the following recommendations as a guide to every physician in biomedical research involving human subjects. They should be kept under review in the future. It must be stressed that the standards as drafted are only a guide to physicians all over the world. Physicians are not relieved from criminal, civil and ethical responsibilities under the laws of their own countries.

I. BASIC PRINCIPLES

1. Biomedical research involving human subjects must conform to generally accepted scientific principles and should be based on adequately performed laboratory and animal experimentation and on a thorough knowledge of the scientific literature.
2. The design and performance of each experimental procedure involving human subjects should be clearly formulated in an experimental protocol which should be transmitted for consideration, comment and guidance to a specially appointed committee independent of the investigator and the sponsor provided that this independent committee is in conformity with the laws and regulations of the country in which the research experiment is performed.
3. Biomedical research involving human subjects should be conducted only by scientifically qualified persons and under the supervision of a clinically competent medical person. The responsibility for the human subject must always rest with a medically qualified person and never rest on the subject of research, even though the subject has given his or her consent.
4. Biomedical research involving human subjects cannot legitimately be carried out unless the importance of the objective is in proportion to the inherent risk to the subject.
5. Every biomedical research project involving human subjects should be preceded by careful assessment of predictable risks in comparison with foreseeable benefits to the subject or to others. Concern for the interests of the subject must always prevail over the interests of science and society.
6. The right of the research subject to safeguard his or her integrity must always be respected. Every precaution should be taken to respect the privacy of the subject and to minimize the impact of the study on the subject's physical and mental integrity and on the personality of the subject.
7. Physicians should abstain from engaging in research projects involving human subjects unless they are satisfied that the hazards involved are believed to be predictable. Physicians should cease any investigation if the hazards are found to outweigh the potential benefits.
8. In publication of the results of his or her research, the physician is obliged to preserve the accuracy of the results. Reports of experimentation not in accordance with the principles laid down in this Declaration should not be accepted for publication.
9. In any research on human beings, each potential subject must be adequately informed of the aims, methods, anticipated benefits and potential hazards of the

study and the discomfort it may entail. He or she should be informed that he or she is at liberty to abstain from participation in the study and that he or she is free to withdraw his or her consent to participation at any time. The physician should then obtain the subject's freely-given informed consent, preferably in writing.

10. When obtaining informed consent for the research project the physician should be particularly cautious if the subject is in a dependent relationship to him or her or may consent under duress. In that case the informed consent should be obtained by a physician who is not engaged in the investigation and who is completely independent of this official relationship.
11. In case of legal incompetence, informed consent should be obtained from the legal guardian in accordance with national legislation. Where physical or mental incapacity makes it impossible to obtain informed consent, or when the subject is a minor, permission from the responsible relative replaces that of the subject in accordance with national legislation. Whenever the minor child is in fact able to give a consent, the minor's consent must be obtained in addition to the consent of the minor's legal guardian.
12. The research protocol should always contain a statement of the ethical considerations involved and should indicate that the principles enunciated in the present Declaration are complied with.

II. MEDICAL RESEARCH COMBINED WITH PROFESSIONAL CARE (Clinical research)

1. In the treatment of the sick person, the physician must be free to use a new diagnostic and therapeutic measure, if in his or her judgement it offers hope of saving life, re-establishing health or alleviating suffering.
2. The potential benefits, hazards and discomfort of a new method should be weighed against the advantages of the best current diagnostic and therapeutic methods.
3. In any medical study, every patient - including those of a control group, if any - should be assured of the best proven diagnostic and therapeutic method. This does not exclude the use of inert placebo in studies where no proven diagnostic or therapeutic method exists.
4. The refusal of the patient to participate in a study must never interfere with the physician–patient relationship.
5. If the physician considers it essential not to obtain informed consent, the specific reasons for this proposal should be stated in the experimental protocol for transmission to the independent committee (I, 2).
6. The Physician can combine medical research with professional care, the objective being the acquisition of new medical knowledge, only to the extent that medical research is justified by its potential diagnostic or therapeutic value for the patient.

III. NON-THERAPEUTIC BIOMEDICAL RESEARCH INVOLVING HUMAN SUBJECTS (Non-clinical biomedical research)

1. In the purely scientific application of medical research carried out on a human being, it is the duty of the physician to remain the protector of the life and health of that person on whom biomedical research is being carried out.
2. The subjects should be volunteers - either healthy persons or patients for whom the experimental design is not related to the patient's illness.
3. The investigator or the investigating team should discontinue the research if in his/her or their judgement it may, if continued, be harmful to the individual.

In research on man, the interest of science and society should never take precedence over considerations related to the well being of the subject.

Appendix B Administrative Matters

I. Responsibilities of the Investigator

- To ensure that he/she has sufficient time to conduct and complete the study and has adequate staff and appropriate facilities and equipment which are available for the duration of the study and to ensure that other studies do not divert essential subjects or facilities away from the study at hand.
- To submit an up-to-date curriculum vitae or Investigator Biography and other credentials (e.g., medical license number in the United States) to GSK and—where required—to relevant authorities. It is recommended that this documentation indicates any previous clinical research experience and history of training in GCP.
- To acquire the reference ranges for laboratory tests performed locally and, if required by local regulations, obtain the laboratory's current certification or Quality Assurance procedure manual.
- To ensure that no clinical samples (including serum samples) are retained on site or elsewhere without the approval of GSK Biologicals and the express written informed consent of the subject and/or the subject's legally authorized representative.
- To perform no other biological assays on the clinical samples site except those described in the protocol or its amendment(s).
- To prepare and maintain adequate subject source data or raw data designed to record observations, and other data pertinent to the study.
- To conduct the study in compliance with the protocol any amendment and "Good Clinical Practice" (GCP) and all applicable regulatory requirements.
- To co-operate with a representative of GSK Biologicals in the monitoring process of the study and in resolution of queries about the data.
- To permit drug regulatory agencies and GSK audits.

II. Protocol Amendments and Administrative changes

- No changes to the study protocol will be allowed unless discussed in detail with the GSK Biologicals' Clinical Development Manager/Medical Monitor and filed as an amendment/administrative change to this protocol.
- Any amendment/administrative change to the protocol will be adhered to by the participating centre(s) and will apply to all subjects. Written IRB/IEC approval of protocol amendments is required prior to implementation, except where permitted by all applicable regulatory requirements; administrative changes and amendments not submitted for approval are submitted to IRBs/IECs for information only.
- Any amendment/administrative change to the protocol will be adhered to by the participating centre(s) and will apply to all subjects. Written IRB/IEC approval of protocol amendments/administrative changes is required prior to implementation, except where permitted by all applicable regulatory requirements; administrative changes and amendments not submitted for approval are submitted to IRBs/IECs for information only.

- Submission of protocol amendments to regulatory agencies will occur in accordance with local regulatory requirements. For some countries, submission to the local regulatory authority may not be required. When submission to the local regulatory authority is required, the timing of the submission relative to IEC/IRB submission or approval and whether or not the authority will provide their approval of or favourable opinion on the amendment before it can be implemented will depend on local regulatory requirements.

III. Sponsor's Termination of Study

GSK Biologicals reserves the right to temporarily suspend or prematurely discontinue this study either at a single site or at all sites at any time for reasons including, but not limited to, safety or ethical issues or severe non-compliance. Reasons for suspension or early termination will be documented in the study file at GSK Biologicals.

If GSK Biologicals determines that suspension or early termination is needed, GSK Biologicals will discuss this with the Investigator (including the reasons for taking such action). When feasible, GSK Biologicals will provide advance notification to the investigator of the impending action prior to it taking effect.

GSK Biologicals will promptly inform, via written communication, all investigators and/or institutions conducting the study, if the study is suspended or terminated for safety reasons, and will also inform the regulatory authorities of the suspension or termination of the study and the reason(s) for the action. If required by applicable regulations, the investigator must inform the IEC/IRB promptly and provide the reason for the suspension or termination.

If the study is prematurely discontinued, all study data must be returned to GSK. In addition, arrangements will be made for all unused investigational product(s) in accordance with the applicable GSK procedures for the study. Financial compensation to investigators and/or institutions will be in accordance with the agreement established between the investigator and/or institutions and GSK.

IV. Remote Data Entry Instructions

Remote Data Entry (RDE) will be used as the method for data collection, which means that subject information will be entered into a computer at the investigational site, preferably within 5 working days of becoming available. The site will be capable of modifying the data to assure accuracy with source documentation. All new/updated information will be reviewed and verified by a GSK Biologicals' representative. This information will finally be stored in a central database maintained by GSK Biologicals. At the conclusion of the study, GSK Biologicals will archive the study data in accordance with internal procedures. In addition, the investigator will be provided with a CD-ROM of the final version of the data generated at the investigational site.

Specific instructions for use of RDE will be included in the training material provided to the investigational site.

V. Monitoring by GSK Biologicals

To ensure compliance with the protocol, monitoring visits by a professional representative of the sponsor will be scheduled to take place early in the study, during the study at appropriate intervals and after the last subject has completed the study. It is anticipated that monitoring visits will occur at a frequency defined and communicated to the investigator before study start.

These visits are for the purpose of confirming that GSK Biologicals' sponsored studies are being conducted in accordance with the ethical principles that have their origins in the Declaration of Helsinki and that are consistent with Good Clinical practice (GCP) and the applicable regulatory requirement(s) (verifying continuing compliance with the protocol, amendment(s), reviewing the investigational product accountability records, verifying that the site staff and facilities continue to be adequate to conduct the study. Direct access to all study-related site and source data/ documents is mandatory for the purpose of monitoring review. The monitor will perform a eCRF review and a Source Document verification (verifying RDE entries by comparing them with the source data/documents that will be made available by the investigator for this purpose: any data item for which the eCRF will serve as the source must be identified, agreed and documented. Data to be recorded directly into the RDE screens will be specified in writing preferably in the source documentation agreement form that is contained in both the monitor's and investigator's study file. For RDE, the monitor will mark completed and approved screens at each visit. The investigator must ensure provision of reasonable time, space and adequate qualified personnel for monitoring visits. Source data verification (SDV) must be conducted using a GSK standard SDV sampling strategy (as defined at the study start in the study specific monitoring guidelines) in which monitors will perform partial SDV for all subjects and full SDV for selected subjects.

VI. Archiving of Data

Following closure of the study, the investigator must maintain all site study records in a safe and secure location. The records must be maintained to allow easy and timely retrieval, when needed (e.g., audit or inspection), and, whenever feasible, to allow any subsequent review of data in conjunction with assessment of the facility, supporting systems, and staff. Where permitted by applicable laws/regulations or institutional policy, some or all of these records can be maintained in a validated format other than hard copy (e.g., microfiche, scanned, electronic for studies with an eCRF, for example); however, caution needs to be exercised before such action is taken. The investigator must assure that all reproductions are legible and are a true and accurate copy of the original, and meet accessibility and retrieval standards, including re-generating a hard copy, if required. Furthermore, the investigator must ensure there is an acceptable back-up of these reproductions and that an acceptable quality control process exists for making these reproductions.

GSK will inform the investigator/ institution of the time period for retaining these records to comply with all applicable regulatory requirements. However, the investigator/ institution should seek the written approval of the sponsor before proceeding with the disposal of these records. The minimum retention time will meet the strictest standard

applicable to that site for the study, as dictated by ICH GCP E6 Section 4.9, any institutional requirements or applicable laws or regulations, or GSK standards/procedures; otherwise, the minimum retention period will default to 15 years.

The investigator/ institution must notify GSK of any changes in the archival arrangements, including, but not limited to, the following: archival at an off-site facility, transfer of ownership of the records in the event the investigator leaves the site.

VII. Audits

For the purpose of compliance with Good Clinical Practice and Regulatory Agency Guidelines it may be necessary for GSK or a Drug Regulatory Agency to conduct a site audit. This may occur at any time from start to after conclusion of the study.

When an investigator signs the protocol, he agrees to permit drug regulatory agencies and GSK audits, providing direct access to source data/ documents. Furthermore, if an investigator refuses an inspection, his data will not be accepted in support of a New Drug Registration and/or Application, Biologics Licensing Application.

Having the highest quality data and studies are essential aspects of vaccine development. GSK has a Regulatory Compliance staff who audit investigational sites. Regulatory Compliance assesses the quality of data with regard to accuracy, adequacy and consistency. In addition, Regulatory Compliance assures that GSK Biologicals' sponsored studies are in accordance with GCP and that relevant regulations/guidelines are being followed.

To accomplish these functions, Regulatory Compliance selects investigational sites to audit. These audits usually take 1 to 2 days. GSK's audits entail review of source documents supporting the adequacy and accuracy of CRFs, review of documentation required to be maintained, and checks on vaccine accountability. GSK's audit therefore helps prepare an investigator for a possible regulatory agency inspection as well as assuring GSK Biologicals of the validity of the database across investigational sites.

The Inspector will be especially interested in the following items:

- Log of visits from the sponsor's representatives
- Study personnel
- Study file
- Safety reporting
- IRB/IEC and regulatory authority approvals
- Facilities
- monitoring
- Vaccine accountability
- Approved study protocol and amendments and investigator brochure

- Informed consent of the subjects (written consent [or witnessed oral if applicable])
- Medical records and other source documents supportive of eCRF data
- Reports to the IRB/IEC and the sponsor
- Record retention.
- GSK Biologicals will gladly help investigators prepare for an inspection.

VIII. Ownership, Confidentiality and Publication

Ownership:

All information provided by GSK and all data and information generated by the site as part of the study (other than a subject's medical records) are the sole property of GSK.

All rights, title, and interests in any inventions, know-how or other intellectual or industrial property rights which are conceived or reduced to practice by site staff during the course of or as a result of the study are the sole property of GSK, and are hereby assigned to GSK.

If a written contract for the conduct of the study which includes ownership provisions inconsistent with this statement is executed between GSK and the study site, that contract's ownership provisions shall apply rather than this statement.

Confidentiality:

Documented evidence that a potential investigator is aware and agrees to the confidential nature of the information related to the study must be obtained by means of a confidentiality agreement.

All information provided by GSK and all data and information generated by the site as part of the study (other than a subject's medical records) will be kept confidential by the investigator and other site staff. This information and data will not be used by the investigator or other site personnel for any purpose other than conducting the study. These restrictions do not apply to: (1) information which becomes publicly available through no fault of the investigator or site staff; (2) information which it is necessary to disclose in confidence to an IEC or IRB solely for the evaluation of the study; (3) information which it is necessary to disclose in order to provide appropriate medical care to a study subject; or (4) study results which may be published as described in the next paragraph. If a written contract for the conduct of the study which includes confidentiality provisions inconsistent with this statement is executed, that contract's confidentiality provisions shall apply rather than this statement.

Publication:

For multicentre studies, the first publication or disclosure of study results shall be a complete, joint multicentre publication or disclosure coordinated by GSK. Thereafter, any secondary publications will reference the original publication(s).

Prior to submitting for publication, presentation, use for instructional purposes, or otherwise disclosing the study results generated by the site (collectively, a “Publication”), the investigator shall provide GSK with a copy of the proposed Publication and allow GSK a period to review the proposed Publication (at least 21 (twenty-one) days [or at least 15 (fifteen) working days for abstracts/posters/presentations]. Proposed Publications shall not include either GSK confidential information other than the study results or personal data on any subject, such as name or initials.

At GSK’s request, the submission or other disclosure of a proposed Publication will be delayed a sufficient time to allow GSK to seek patent or similar protection of any inventions, know-how or other intellectual or industrial property rights disclosed in the proposed Publication.

If a written contract for the conduct of the study, which includes publication provisions inconsistent with this statement is executed, that contract’s publication provisions shall apply rather than this statement.

Appendix C Handling of Biological Samples Collected by the Investigator

Instructions for Handling of Serum Samples

When materials are provided by GSK Biologicals, it is MANDATORY that all clinical samples (including serum samples) will be collected and stored using exclusively those materials in the appropriate manner. The use of other materials could result in the exclusion of the subject from the ATP analysis. The investigator must ensure that his/her personnel and the laboratory(ies) under his/her supervision comply with this requirement. However, when GSK Biologicals does not provide material for collecting and storing clinical samples, then appropriate materials from the investigator's site are to be used.

1. Collection

The whole blood (by capillary or venous route) should be collected observing appropriate aseptic conditions. It is recommended that Vacutainer® tubes WITH integrated serum separator (e.g. Becton-Dickinson Vacutainer® SST or Corvac® Sherwood Medical) be used to minimize the risk of haemolysis and to avoid blood cell contamination of the serum when transferring to standard serum tubes.

2. Serum separation

These guidelines aim to ensure high quality serum by minimizing the risk of haemolysis, blood cell contamination of the serum or serum adverse cell toxicity at testing.

- For separation of serum using Vacutainer® tubes, the instructions provided by the manufacturer should be followed. Often the manufacturer's instruction states that the relative centrifugal acceleration known also as "G" must be "between 1000 and 1300 G" with tubes spinning for ten minutes. Error in calculation of centrifuge speed can occur when laboratory personnel confuse "G" acceleration with "RPM" (revolutions per minute). The speed of centrifugation must be calculated using the "G" rate provided in the manufacturer's instructions and the radius of the centrifuge head. After measuring the radius of the centrifuge machine, a speed/acceleration nomograph must be employed to determine the centrifuge speed in "RPM".
- Following separation, the serum should be aseptically transferred to the appropriate standard tubes using a sterile disposable pipette. The serum should be transferred as gently as possible to avoid blood cell contamination.
- The tube should not be overfilled (max. 3/4 of the total volume) to allow room for expansion upon freezing.
- The tube should be identified by the appropriate label provided by GSK Biologicals (see point 3).

3. Labelling

- The standard labels provided by GSK Biologicals should be used to label each serum sample.
- If necessary, any hand-written additions to the labels should be made using indelible ink.
- The label should be attached to the tube as follows (see diagram):
  - first attach the paper part of the label to the tube
  - then wrap the label around the tube so that the transparent, plastic part of the label overlaps with the label text and bar code and shields them.

This will ensure optimal label attachment.

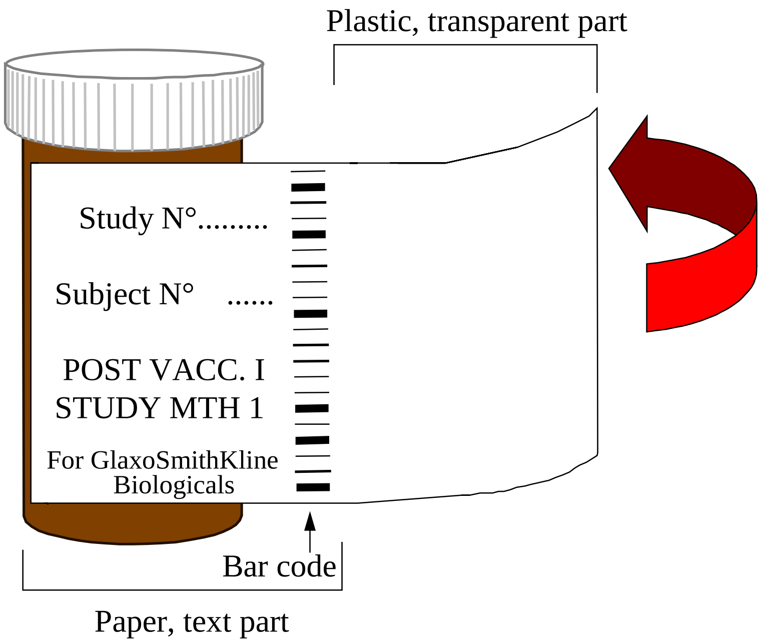

- Labels should not be attached to caps.

4. Sorting and storage

- Tubes should be placed in the GSK Biologicals' cardboard boxes in numerical order from left to right, starting from the lower left hand corner, beginning with the pre-vaccination samples series, then with the post-vaccination sample series.
- The tubes of serum should be stored in a vertical position at approximately -20°C (alternatively at approximately -70°/80°C is also acceptable) until shipment to GSK Biologicals. The storage temperature should be checked regularly and documented. Wherever possible, a backup facility for storage of serum samples should be available.
- A standard Biological Specimen Listing Form, specifying the samples being shipped for individual subjects at each timepoint, should be prepared for each shipment. A

copy of this list should be retained at the study site, while the original should be sealed in a plastic envelope and shipped with the serum samples.

- Once shipment details are known, a standard Specimen Transfer Form must be completed and faxed to GSK Biologicals to the number provided below. A copy of the Specimen Transfer Form must be in the parcel.[1]

GLAXOSMITHKLINE BIOLOGICALS

Attention Biospecimen Reception
Clinical Immunology
R & D Department/Building 44
Rue de l'Institut, 89
B-1330 Rixensart – Belgium
Telephone: +32-2-656 8949 or +32-2-656 6130
or +32-2-656 8549 or +32-2-656 6108
Fax: +32-2-656 6052
E-mail: rix.ugbiospecimen-reception@gskbio.com

[1] The Biological Specimen Listing Form and the Specimen Transfer Form are standard documents used in GSK Biologicals' clinical trials. These documents are provided by GSK Biologicals' Clinical Trials' monitor at study initiation.

Appendix D Shipment of Biological Samples

Instructions for Shipment of Serum Samples

Serum samples should be sent to GSK Biologicals at regular intervals. The frequency of shipment of samples should be decided upon by the Site Monitor, Central Study Coordinator and the investigator prior to the study start.

Serum samples should always be sent by air, preferably on a Monday, Tuesday or Wednesday, unless otherwise requested by the sponsor.

Serum samples must be placed with dry ice (maximum -20°C) in a container complying with International Air Transport Association (IATA) requirements. The completed standard Biological Specimen Listing form should always accompany the shipment.

The container must be clearly identified with the labels provided by GSK Biologicals specifying the shipment address and the storage temperature (-20°C).

The airway bill should contain the instruction for storage of samples at maximum -20°C.

A "proforma" invoice, stating a value for customs purposes only, should be prepared and attached to the container. This document should contain the instruction for storage of samples at maximum -20°C.

Details of the shipment, including:

* number of samples
* airway bill
* flight number
* flight departure and arrival times

should be sent by fax or by e-mail, two days before shipment, to:

GLAXOSMITHKLINE BIOLOGICALS
Attention Biospecimen Reception
Clinical Immunology
R & D Department/Building 44
Rue de l'Institut, 89
B-1330 Rixensart – Belgium
Telephone: +32-2-656 8949 or +32-2-656 6130
or +32-2-656 8549 or +32-2-656 6108
Fax: +32-2-656 6052
E-mail: rix.ugbiospecimen-reception@gskbio.com

Appendix E Vaccine supplies, packaging and accountability

1. Vaccine and/or other supplies

GSK Biologicals will supply the following study and co-administered vaccines, sufficient number of doses to administer to all subjects as described in the present protocol.

- 10Pn-PD-DiT vaccine in prefilled syringes
- *Engerix-B* vaccine in prefilled syringes
- *Havrix* (pediatric) vaccine in prefilled syringes
- *Infanrix hexa* (DTPa-HBV-IPV/Hib): prefilled syringe (DTPa-HBV-IPV component) + monodose vial (Hib component)
- DTPa-IPV/Hib: prefilled syringe (DTPa-IPV component) + monodose vial (Hib component)

In Argentina**,** Colombia and Panama, *Havrix* will be used for vaccination of subjects against hepatitis A at the age of 12-15 months (visit 5) and at 18-21 months of age.

In Argentina, all subjects will receive *Neisvac-C* at the age of 12 months.

All subjects in all participating countries will receive at 12 months of age the MMR vaccine from the EPI program.

In Colombia and Panama, *Varilrix* will be administered to all subjects at 12 months of age, supplied in monodose vials and container of diluent.

In Colombia, 2 doses of licensed Rotarix vaccine will be offered to all subjects within the first six months of life, supplied in monodose vials and prefilled syringes**.**

An appropriate amount of the study vaccines will be supplied for replacement in case of breakage, bad storage conditions or any other reason that would make the vaccine unusable (i.e. given by mistake to another subject). The investigator should use the central randomization system to obtain a replacement vial for any study/control or co-administered vaccine. The system will ensure that the replacement vial is the same formulation as the randomized vaccine.

An appropriate amount of vaccines will be supplied for ‘over-randomization’.

Labels for sample identification:

The investigator will receive labels from GSK Biologicals to identify samples taken from each subject from the ‘Immuno and reacto’ subset at each timepoint . Each label will contain the following information: study number, identification number for the subject (e.g. **subject number**), sampling timepoint (e.g., post vacc 3), timing (e.g., study Month 5). In addition GSK Biologicals will provide labels to identify samples taken from each subject in the ‘Additional Immuno’ subset.

Other supplies provided by GSK Biologicals:

In addition to the vaccines, the study documentation and the sample labels, the investigator will receive supplies to conduct the study such as:

- tubes with screw caps for serum samples,
- racks and cardboard boxes for the tubes of serum.

The investigator or study staff must sign a statement that he/she has received the clinical supplies for the study.

It is NOT permitted to use any of the supplies provided by GSK Biologicals for purposes other than those specified in the protocol.

2. Vaccine packaging

The vaccines will be packed in labelled boxes. Each treatment will consist of one box containing the study vaccine/control and the co-administered vaccine. To differentiate between the vaccines, a specifically coloured label will be used on the syringes of the study vaccine/control. The box label will contain, as a minimum, the following information: study number, treatment number, lot number, instructions for vaccine administration and any other local relevant regulatory requirements.

3. Vaccine shipment from GSK Biologicals Rixensart to local country medical department, dispatching centre or investigational site from local country medical department to investigational site

Upon reception of the shipment, its content, quality and maintenance of the cold-chain must be checked.

The supplies receipt documents should then be returned to:

Attention of Clinical Trial Supplies Unit
Clinical Operations Logistics
GSK Biologicals Rixensart
Fax : +32 (0)2 656 75 17
E-mail: rix.ugCTSU@gskbio.com.

In case of any temperature deviation, the official written approval for the use of vaccine must be obtained from GSK Biologicals.

4. Vaccine accountability

All monodose vials/pre-filled syringes must be accounted for on the form provided or any other form authorized by GSK.

At all times the figures on supplied, used and remaining vaccine doses should match. At the end of the study, it must be possible to reconcile delivery records with those of used and unused stocks. An explanation must be given of any discrepancies.

After approval from GSK Biologicals and in accordance with GSK SOP WWD-1100, used and unused vaccine vials/syringes should be destroyed at the study site using locally

approved biosafety procedures and documentation unless otherwise described in the protocol. If no adequate biosafety procedures are available at the study site, the used and unused vaccine vials/syringes are to be returned to an appropriate GSK Biologicals site for destruction, also in accordance with current GSK SOP WWD-1100.

5. Transfers of clinical vaccines or products from country medical department or dispatch centre to study sites or between sites

Storage temperatures must be maintained during transport and deviations must be reported to GSK for guidance. All transfers of clinical vaccines or products must be documented using the Clinical Supply Transfer Form or other local document.

All packaging and shipment procedures for transfer of clinical vaccines or products must follow procedures approved by the sponsor.

Clinical vaccines or products should always be sent by contract courier designated by the sponsor, unless otherwise requested by the sponsor.

Appendix F Definitions of radiographic outcomes and guidelines for radiographic techniques

A) Definitions of radiographic outcomes (WHO, 2001)

The reading of the image will be classified as:

- Consolidated CAP: a dense opacity that may be a fluffy consolidation of a portion of whole or of a lobe or of the entire lung, often containing air bronchograms and sometimes associated with pleural effusion. Pleural effusion refers to the presence of fluid in the pleural space between the lung and the chest wall. In most cases this will be seen at the costo-phrenic angle or as a layer of fluid adjacent to the lateral chest wall. This does not include liquid seen in the horizontal or oblique fissures. Pleural effusion is considered as primary endpoint if it is in the lateral pleural space (and not just in the minor or oblique fissure) and is spatially associated with a pulmonary parenchymal infiltrate (including other infilatrate), OR if the effusion obliterates enough of the hemithorax to obscure an opacity.
- Non-consolidated CAP: linear and patchy densities (interstitial infiltrate) in a lacy pattern involving both lungs, featuring peribronchal thickening and multiple areas of atelectasis. Lung inflation is normal to increased. It also includes minor patchy infiltrates that are not of suffiecient magnitude to constitute primary end-point consolidation, and small areas of atelectasis which in children can be difficult to distinguish from consolidation.
- No pneumonia: absence of consolidation, pleural effusion and/or abnormal infiltrates on the digital CXR image**.**
- Uninterpretable: an image is classified as “uninterpretable” if the features of the image are not interpretable in terms of presence or absence of “primary end-point” without additional images. No further reading should be made for other infiltrates on such images.

B) Guidelines for radiographic techniques (WHO, 2003).

Performance of chest X-ray

Chest AP Erect Basic

Infants and small children weighing up to 15 kg, hanging by the upper arms

- Cassette speed: cassette with screen film combination, nominal speed 200 in the cassette holder
- Cassette size: 24 x 30 cm (10 x 12 inches).
- Use Right or Left mark

Exposure volumes	KV	mAs range
Child	90	1.25-2.50

Technique

1. Put the cassette in the cassette holder. Collimate to the format.
2. Position the patient. The child is held hanging by the upper arms (If possible, its feet can be supported by a stool or the floor or by another person holding the thighs) with its back resting against the front of the cassette holder.
3. THE PERSON HOLDI NG THE CHILD, preferably one of the parents, MUST WEAR A LEAD APRON and whenever possible LEAD GLOVES.
4. Center between the nipples. COLLIMATE FURTHER, if possible.
5. Expose when the infant is not moving, preferably in INSPIRATION.

Chest AP Supine

Infant weighing up to 10 kg

- Cassette speed: cassette with screen film combination, nominal speed 200 on the cassette holder
- Cassette size: 18 x 24 cm (8 x 10 inches).
- Use Right or Left marker

Exposure volumes	KV	mAs range
Child	70	1.6-3.2

Technique

1. Center the cassette on top of the cassett holder. Collimate to the format.
2. Lie the infant on its back on the cassette. THE INFANTS’ HEAD AND LEGS MUST BE SUPPORTED. THOSE SUPPORTING, preferably the infants’ parents, MUST WEAR LEAD APRONS, and, whenever possible, LEAD GLOVES.
3. Center between the nipples. COLLIMATE FURTHER, if possible.
4. Expose when the infant is not moving, preferably in INSPIRATION.

Comments: Use a protective lead strip over the infant’s pelvic area.

Quality X-ray parameters

- ID on the right side
- Position: clavicles and ribs symmetric on each side of the spine
- Boundaries: Rib cage and costophrenics angles
- Inspiration: dome of the diaphragm is below the anterior tip of the 6th rib
- Movement: heart, diaphragm, central vessels and ribs sharply defined, without blurring
- Exposure: vascular shadows can be seen in lung periphery, thoracic vertebrae and large lower lobe vessels visible through cardiac silhouette

- Contrast: background outside patient's silhouette is black. Bones and airway easily distinguished from soft tissue.

Film speed and cassettes

The speed of the screen-film combination should be written on the cassettes. There are two kind of systems:

- Blue system (calcium-tungstate and rapid yttrium-tantalate screens), with a relative speed of 50 and a resolution of 8 lp/mm
- Green system (gadolinium-oxysulfide) the actual speed varies with the kV value: low speed at low kV values, nominal speed at 70-75 kV and little higher speed at higher kV values.

The Green system is the recommended for the CXR in children since it can adapt to the high voltage and high speed cassettes, which limits the problems due to movements of the children.

A common error seen on the field is that less powerful cassettes are available (50-60 kV) and adjustment of the exposure time is not considered. Thus, a bad quality CXR is obtained.

Film processing

- Automatic processing: if used, it is important to follow the instructions that come with the machine. It should be noted that even a small automatic film processor requires a separate, totally reliable electric supply for 3-4 kW, access to plenty clean water and well train personnel for daily use and maintenance.
- Manual film processing: can be used but requires more precision and attention from the darkroom technician, specifically if the room temperature is high and the ventilation is poor.

Digital X-ray quality parameters

It is recommended to follow the guidelines listed in the manual 'Standardization of interpretation of chest radiographs for the diagnosis of pneumonia in children' (WHO Pneumonia Vaccine Trial Investigators' Group).

The objectives are:

- To establish standard definitions for the interpretation of chest radiographs in children with suspected pneumonia for use as an epidemiological tool.
- To establish a mechanism for ensuring the quality of radiographic images stored in digital format.

Optimal interpretation of a chest radiograph will depend on the quality of the image and the methods used to interpret the image. Since interpretation will mainly be performed on digitized images, the quality of the image being interpreted will depend on the quality of the original image (the radiograph) as well as the quality of the digitization process. In

addition, the monitor and the setting of the monitor used to view the digitized images are crucial for optimal interpretation.

Quality of the original radiograph

A readable X-ray is crucial for the diagnosis of radiological pneumonia. It is recommended that radiologists at the study sites closely interact with radiographers and technicians, stressing the importance of film quality as well as adequate radiation protection measures. This is to ensure that the proportion of uninterpretable and suboptimal films are kept to a minimum, and that radiation exposure for patients as well as staff members and/or other people is kept as low as possible, and according to national and international laws and regulations. When the initial radiograph is unsatisfactory for the purposes of treating the patient, the treating physician may authorize a repeat radiograph.

A few suggested guidelines for checking film quality are as follows:

1. Exposure: ability to discern the bones, soft tissue and lungs as different densities?
2. Development: is there complete blackening of the film outside the body on the edge of the film (where the X-rays have passed through air) and maintaining whiteness in the very dense areas such as the lower thoracic spine behind the heart?
3. Positioning: are the medial ends of the clavicles approximately equidistant from the midline?

Digitization of radiographic images

Purpose-built X-ray scanning machines – the CCD film digitizes provide results that are equivalent to laser scanners but are less expensive. Most of the current study sites are proposing to use CCD scanners. This method is recommended by the World Health Organization Pneumonia Vaccine Trial Investigators' Group in their manual 'Standardization of interpretation of chest radiographs for the diagnosis of pneumonia in children' (2001, WHO/V&B/01.35).

GSK will provide Vidar scanners to harmonise the digitization of radiographic images. Additional information on the Vidar scanner is available on the Internet at: http://www.filmdigitiser.com.

Appendix G Guidelines for Middle ear Fluid collection with Channel Directed Tympanocentesis (CTD) Speculum

All efforts should be made to optimize procedure (i. e. child calm and still, external ear canal thoroughly cleaned, good visibility of the eardrum) and thereby maximise the chances of collecting a sample of MEF.

The tympanocentesis procedure is briefly outlined below.

The use of systemic analgesics before tympanocentesis is left to the discretion of the physician. Because of their anti-microbial activity, local anaesthetics will not be used.

1. After administering the analgesic of choice, attach the reusable CDT® Aspirator to the CDT® Speculum and affix the speculum to the otoscope.

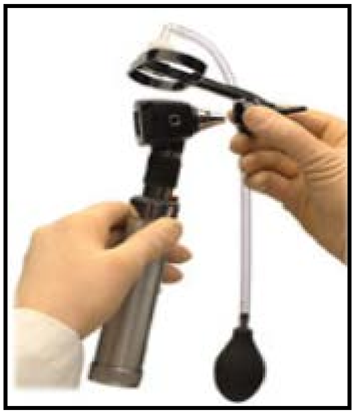

2. Compress the aspirator bulb and then position the speculum in the patient's ear. Visually acquire the point of intended impact in the inferior half of the tympanic membrane. Extend the needle to perforate the membrane, release pressure on the bulb to aspirate fluid, and then release pressure on the needle actuator to retract the needle.

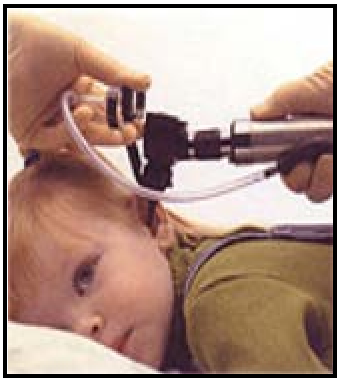

3. Withdraw the CDT® Speculum, expel the aspirated fluid to the transport medium and send to the local lab within 12 hours for bacteriological testing (culture, isolation and antibiotic sensitivity) for therapeutic guidance according to local practice.

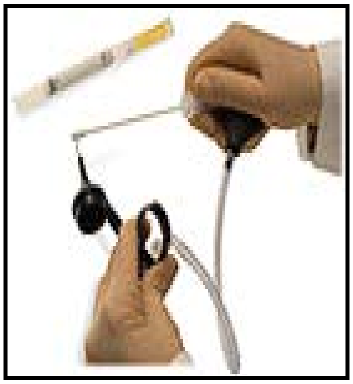

4. Incubate at 37°C.
5. For further sample handling, see Section 5.5.2.2.3.

If paracentesis is inadvertently performed, MEF should be collected by syringe aspiration in the ear canal. The ear canal should be thoroughly cleaned before the procedure to avoid masking of NTHi by local flora.

Similarly, spontaneous drainage should be collected for culture, using syringe aspiration or swabs as appropriate.

Appendix H Procedures for the detection of *Streptococcus pneumoniae* and *Haemophilus influenzae* in each country and at the Central Laboratory

For a more detailed description of the laboratory processes, please refer to the specific laboratory procedures.

1. At a country level

At the country level, the automated BactAlert or BacTec system will be used to detect presence of bacteria in blood samples, based on CO_2 production. Positive samples will be stained, examined microscopically and plated maximum within 12 hours. If gram-positive diplococci or gram-negative coccus bacilli or gram-negative diplococci are observed on the smear, they will be subcultured on a blood agar or a chocolate agar plate. Identification of eventual *S. pneumoniae* present will be done by performing the optochin and bile test.

2. Pneumococci and *H. influenzae* strains transport to the Central Laboratory Eurofins Medinet Inc..

All the strains identified as *S. pneumoniae* and *H. influenzae* must be sent to the Central Laboratory Eurofins Medinet Inc. in USA. For this purpose use the supplied transport media.

3. Eurofins Medinet Inc. Central Laboratory: laboratory activities.

Target pathogens will be sent to:

Eurofins Medinet, Inc.
13665 Dulles Technology Drive, #100R
Herndon, VA 20171
Tel: +1 703.480.2500
Fax: +1 703.480.2655
www.eurofinsmedinet.com

<u>Streptococcus pneumoniae</u>

1. *S. pneumoniae* confirmation

a. Hemolytic pattern on blood agar (alpha-hemolytic)

b. Optochin test: This test will differentiate *S. pneumoniae* from other alpha haemolytic streptococci. This test is used to determine the effect of optochin (ethylhydrocupreine hydrochloride) on a microorganism. Optochin lyses S. pneumoniae (positive test) but alpha streptococci are resistant (negative test). Pneumococci may, therefore, be differentiated from other alpha hemolytic streptococci by the formation of a zone of inhibition around a Taxo P disk (disks impregnated with approximately 5.0 ug of hydrocupreine hydrochloride [optochin]). The disk is placed on a blood agar plate heavily inoculated with a pure culture of S. pneumoniae, and optochin susceptibility is assessed after incubation overnight.

c. Bile solubility test: The bile solubility test differentiates S. pneumoniae (bile soluble) from other alpha-haemolytic streptococci (bile insoluble). This test determines the ability of an organism to be lysed in the presence of bile salts (sodium deoxycholate). Bile salts decrease surface tension at the medium-membrane interface and also cause derangement of the cell membrane. However, S. pneumoniae produces an intracellular autolytic enzyme causing a rapid autolysis of the micro-organism when growing on artificial media. The role of bile salts is to accelerate this natural autolytic process by combining with the pneumococcal cell and activating its autolysin. This test will be performed by spot testing the colony with sodium-deoxycholate and assessing its solubility (lysis)**..**

2. Susceptibility testing through the broth micro dilution test

The Minimal Inhibitory Concentration (MIC) is a quantitative measurement of in vitro activity of an antimicrobial agent against a given bacterial isolate. The standard test is performed by exposing the organism to doubling dilutions of antibiotic. The MIC is the lowest antibiotic concentration that inhibits completely the growth of the organism as determined with the naked eye.

Streptococcus pneumoniae							CMP6ADAI				
Levoflox 0.06	Levoflox 0.12	Levoflox 0.25	Levoflox 0.5	Levoflox 1	Levoflox 2	Levoflox 4	Levoflox 8	Cipro 1	Cipro 2	Cipro 4	Penicillin 0.03
Penicillin 0.06	Penicillin 0.12	Penicillin 0.25	Penicillin 0.5	Penicillin 1	Penicillin 2	Penicillin 4	Penicillin 8	Amox/clav 0.015/0.08	Amox/clav 0.03/0.015	Amox/clav 0.06/0.03	Amox/clav 0.12/0.06
Amox/clav 0.25/0.12	Amox/clav 0.5/0.25	Amox/clav 1/0.5	Amox/clav 2/1	Amox/clav 4/2	Ceftriax 0.015	Ceftriax 0.03	Ceftriax 0.06	Ceftriax 0.12	Ceftriax 0.25	Ceftriax 0.5	Ceftriax 1
Ceftriax 2	Ceftriax 4	Cefurox ax 0.12	Cefurox ax 0.25	Cefurox ax 0.5	Cefurox ax 1	Cefurox ax 2	Cefurox ax 4	Clarithrom 0.015	Clarithrom 0.03	Clarithrom 0.06	Clarithrom 0.12
Clarithrom 0.25	Clarithrom 0.5	Clarithrom 1	Azithrom 0.015	Azithrom 0.03	Azithrom 0.06	Azithrom 0.12	Azithrom 0.25	Azithrom 0.5	Azithrom 1	Azithrom 2	Azithrom 4
Erythrom 0.015	Erythrom 0.03	Erythrom 0.06	Erythrom 0.12	Erythrom 0.25	Erythrom 0.5	Erythrom 1	Erythrom 2	Erythrom 4	Clindamy 0.015	Clindamy 0.03	Clindamy 0.06
Clindamy 0.12	Clindamy 0.25	Clindamy 0.5	Clindamy 1	Vanco 0.06	Vanco 0.12	Vanco 0.25	Vanco 0.5	Vanco 1	SXT 0.06/1.2	SXT 0.12/2.4	SXT 0.25/4.75
SXT 0.5/9.5	SXT 1/19	SXT 2/38	SXT 4/76	Tetracyc 0.06	Tetracyc 0.12	Tetracyc 0.25	Tetracyc 0.5	Tetracyc 1	Tetracyc 2	Tetracyc 4	Pos Control

Antimicrobial Agent	MIC Range	# of wells
Levofloxacin	0.06-8	8
Ciprofloxacin	1-4`	3
Penicillin	0.03-8	9
Amoxicillin/clavulanate	0.015/0.008-4/2`	9
Ceftriaxone (non-men)	0.015-4	9
Cefuroxime axetil	0.12-4	6
Clarithromycin	0.015-1	7

Antimicrobial Agent	MIC Range	# of wells
Azithromycin	0.015-4	9
Erythromycin	0.015-4	9
Clindamycin	0.015-1	7
Vancomycin	0.06-1	5
Trimeth/Sulfa (SXT)	0.06/1.2-4/76`	7
Tetracycline	0.06-4	7
Pos Control		1

3. *S. pneumoniae* capsular serotyping.

S. pneumoniae capsular serotyping will be subcontracted to University Medical Center Amsterdam, National Pneumococcal Reference**.** *S. pneumoniae* capsular serotyping is performed according to the Danish Nomenclature System and it is done by Neufeld-Quellung reaction, using 12 pooled antisera and selected factor antisera (Statens Serum Institut of Denmark).The type or group is established from the reaction pattern. This system allows the identification of the most common 21 serotypes or serogroups worldwide.

4. *S. pneumoniae* strains storage

Each strain of *S. pneumoniae* completely processed as described in this protocol will be stored by freezing at –70ºC (Brucella broth with 20% glycerol) at the Central Laboratory Eurofins Medinet Inc.

<u>Haemophilus influenzae</u>

1. *H. influenzae* confirmation

a. Morphology

b. Growth on Chocolate agar and no growth on blood agar

c. Quad Plate Method: The Haemophilus (Hemo) Identification (ID) Quad plate (with growth factors) is used for the differentiation and presumptive identification of Haemophilus species based on requirements of growth factor X and or V and hemolytic reactions. H. influenzae requires both X factor (component of hemin) and V factor (NAD) for growth. A four quadrant plate that contains Brain Heart Infusion Agar in quadrants I, II, and III and Blood Agar Base in quadrant IV will be used. Quadrant I: Enriched with hemin to supply X factor. Quadrant II: Enriched with Isovitalex Enrichment to supply V factor and other nutrients, such as thiamine and cysteine, which stimulate the growth of Haemophilus species. Quadrant III: Contains both hemin (X factor) and Isovitalex enrichment (V factor). Quadrant IV: Supplemented with NAD to supply V factor; horse blood to provide X factor and demonstrate hemolytic reactions. If the isolate is H. influenzae, it should grow in quadrants III and IV**.**

2. *H. influenzae*: capsular serotyping

H. influenzae serotyping will be performed by Qualitative slide Agglutination test (FDA approved REMEL kit). Capsule production has been shown to be an important virulence factor for H. influenzae, and encapsulated strains are classified serologically into six types according to the chemical structure of the capsular antigen. Strains possessing these antigens are specifically agglutinated by the homologous antiserum, and a culture of an encapsulated strain may therefore be typed by slide agglutination tests. Haemophilus influenzae Agglutinating Sera are intended for use in the qualitative slide agglutination test to identify serologically the type antigen of pathogenic strains of H. influenzae (types a to f) for epidemiological and diagnostic purposes.

3. Determination of beta-lactamase production

Beta-lactamase production by H. influenzae will be assessed using a DrySlide Nitrocefin test. In brief, colonies of a H. influenzae isolate will be smeared onto a slide containing the nitrocefin reagent. Nitrocefin as a substrate contains a beta-lactam ring, which when hydrolyzed (in the presence of beta-lactamases) produces cephalosporanic acid causing a colorimetric change. A change from yellow-pink indicates the production of beta-lactamase.

4. Susceptibility testing through the broth micro dilution test

Haemophilus influenzae							CMP2ADAI				
Levoflox 0.004	Levoflox 0.008	Levoflox 0.015	Levoflox 0.03	Levoflox 0.06	Levoflox 0.12	Levoflox 0.25	Levoflox 0.05	Levoflox 1	Levoflox 2	Levoflox 4	Levoflox 8
Ampicillin 0.12	Ampicillin 0.25	Ampicillin 0.5	Ampicillin 1	Ampicillin 2	Ampicillin 4	Ampicillin 8	Amox/cl 0.015/0.008	Amox/cl 0.03/0.015	Amox/cl 0.06/0.03	Amox/cl 0.12/0.06	Amox/cl 0.25/0.12
Amox/cl 0.5/0.25	Amox/cl 1/0.5	Amox/cl 2/1	Amox/cl 4/2	Amox/cl 8/4	Amox/cl 16/8	Ceftriax 0.015	Ceftriax 0.03	Ceftriax 0.06	Ceftriax 0.12	Ceftriax 0.25	Ceftriax 0.5
Ceftriax 1	Ceftriax 2	Ceftriax 4	Ceftriax 8	Ceftriax 16	Cefurox ax 0.06	Cefurox ax 0.12	Cefurox ax 0.25	Cefurox ax 0.5	Cefurox ax 1	Cefurox ax 2	Cefurox ax 4
Cefurox ax 8	Cefurox ax 16	Clarithrom 0.015	Clarithrom 0.03	Clarithrom 0.06	Clarithrom 0.12	Clarithrom 0.25	Clarithrom 0.5	Clarithrom 1	Clarithrom 2	Clarithrom 4	Clarithrom 8
Clarithrom 16	Clarithrom 32	Azithrom 0.015	Azithrom 0.03	Azithrom 0.06	Azithrom 0.12	Azithrom 0.25	Azithrom 0.5	Azithrom 1	Azithrom 2	Azithrom 4	Azithrom 8
Azithrom 16	Azithrom 32	Erythrom 0.015	Erythrom 0.03	Erythrom 0.06	Erythrom 0.12	Erythrom 0.25	Erythrom 0.5	Erythrom 1	Erythrom 2	Erythrom 4	Erythrom 8
Erythrom 16	Erythrom 32	SXT 0.015	SXT 0.03	SXT 0.06	SXT 0.12	SXT 0.25	SXT 0.5	SXT 1	SXT 2	SXT 4	Pos Control

Antimicrobial Agent	MIC Range (mg/mL)	# of wells
Levofloxacin	0.004-8	12
Ampicillin	0.12-8	7
Amoxicillin/clavulanate	0.015/0.008	11
Ceftraixone	0.015-16	11
Cefuroxime axetil	0.06-16	9

Antimicrobial Agent	MIC Range (mg/mL)	# of wells
Clarithromycin	0.015-32	12
Azithromycin	0.015-32	12
Erythromycin	0.015-32	12
Trimeth/Sulfa (SXT)	0.015/0.3-4/76	9
Pos Control		1

5. H. influenzae strains storage

Each strain of H. influenzae completely processed as described in this protocol will be stored frozen at -70°C (Brucella broth with 20% glycerol) at the Central Laboratory Eurofins Medinet Inc.

Appendix I AOM severity scales used in COMPAS

Acute Otitis Media (AOM) is a common problem that creates a burden on parents and children in any health care system. Nearly all children experience at least 1 episode of AOM by the time they are 3 years old [Teele et al, 1989]. AOM, even if treated correctly, may have a recurrent or persistent nature. Around 10 – 20 % of children will experience subsequent episodes or have persistent mild infections that last up to 12 weeks [Teele et al, 1989].

In many countries, antibiotics are used initially for treatment of AOM. However, the appropriateness of antibiotic prescription at the initial visit remains controversial. Some clinicians favour withholding antibiotics in part because some fraction of AOM is caused by virus and in part because they are concerned that unnecessary antibiotics encourage the emergence of mutidrug-resistant bacterial stains.

The emergence of a 10 valent pneumococcal vaccine that could also induce protection against non-encapsulated Haemophilus influenzae may be one of the most important new developments on immunization in the coming decade. For this reason, the COMPAS study has as ***first secondary*** ~~a primary~~ objective to demonstrate vaccine efficacy against clinically confirmed AOM caused by vaccine serotype pneumococci and *H. influenzae*. **(Amended 09 September2010)**

In order to better describe and understand the impact of 10Pn-PD-DiT vaccination on the AOM burden of disease, the severity of each AOM case will be measured and reported during the study. Unfortunately, there are no universally validated scales in the literature to measure AOM severity in vaccine clinical trials performed in infants . Previous experiences shown excellent results on AOM severity measurement by combining subjectives and objective parameters: parent perception of severity and otoscopy findings [Friedman et al, 2006]:

1. Dagan Scale

- Based on objective and subjective parameters
- Scoring varies between Mild (0-4 points), Moderate (5-7 points) and Severe (8-15 points)

Acute otitis media clinical/otologic score based on temperature, irritability and ear tugging on the day of the visit as reported by parents and redness and bulging of the tympanic membrane at the otologic examination					
Score	Temperature (°C)	Irritability	Tugging	Redness	Bulging
0	<38.0	Absent	Absent	Absent	Absent
1	38.0-38.5	Mild	Mild	Mild	Mild
2	38.6-39.0	Moderate	Moderate	Moderate	Moderate
3	>39.0	Severe	Severe	Severe	Severe*

*Including drainage pus

2. Friedman Scale (also known as McCormick) [Friedman et al, 2006]

- Very complex, combines 4 scales (ETG-5, OM-3, FACES and OS-8/Tympanic Pictures
  - ETG-5 is based on 4 subjective parameters (ear pain, irritability, feeding and sleeping) and 1 objective parameter (fever).

	Score = 0	Score = 4	Score = 7
Fever	< 100.4 °F	100.4 – 102,2	> 102.2
Ear ache	Tugging	Occasional	Frequent
Irritability	None	Occasional	Frequent
Feeding	Feeds well	Mild decrease in appetite	Very poor appetite
Sleeping	Normal	Some what restless sleep	Very poor sleep

  - OM-3 consists of a parent oriented questionnaire to indirectly assess the subject´s health. Parent involvement and feedback is required, leading to the generation of subjective data.
  - FACES is not useful in clinical trials where toddlers or very young children are involved, since the scale is based on the child's disease perception.

Which face describes your child's symptoms during the past 24 hours?

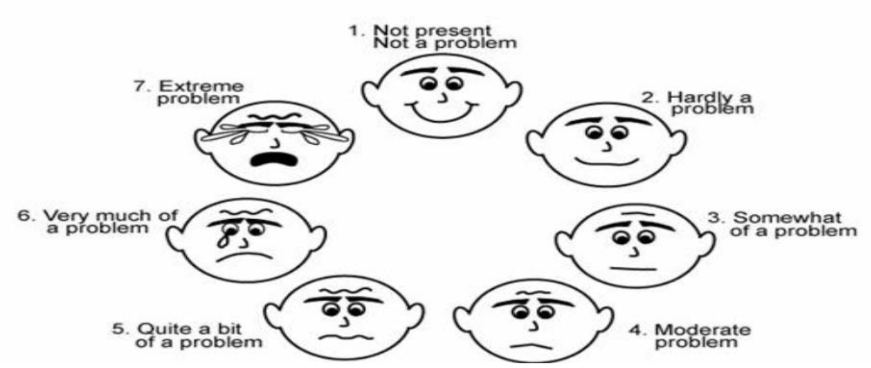

© David P.- McCormick M.D., 2006

  - OS-8/Tympanic pictograms: This is an objective scale, which allows the ENT specialist to grade the disease, based on 8 different pictograms, according to the image seen at the moment of visualising the tympanic membrane. Very easy to use.

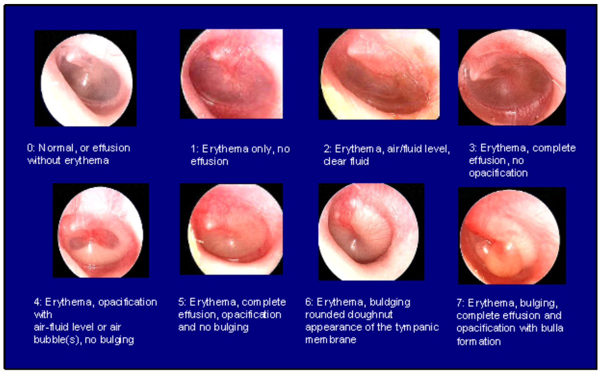

No standardized methods exist to measure AOM severity. There are however scales that have been used in previous clinical trials combining subjective and objective AOM parameters to perform assessment on AOM cases. The association of the Dagan and McCormick scales demonstrated excellent performance with easy implementation. However, OM-3 and FACES included in McCormick scale are found not to be relevant for the COMPAS study, since involvement of parents on the assessment of each case is too subjective and subjects are too young to express their discomfort.

Therefore, AOM severity assessment will combine “objective elements”, as described in the Friedman scale (using only ETG-5 and OS-8 scales), with subjective elements required by the Dagan scale. Collection of clinical data will be performed by the pediatrician /general physician who refers the case; pictograms assessment and other objective elements related to the direct observation of the ear drum will be performed by the ENT specialist.

Appendix J Mathematical details about sample size determination

1. Monotonic transformation between p and VE

The VE can be estimated by

$$VE = 1 - \frac{n1/N1}{n2/N2} \approx 1 - \frac{n1}{n2}$$ since N1/N2 ≈ 1 in a 1:1 randomization ratio

where ni=number of first episodes in group i (1=vaccine group and 2=control group)

Ni=number of subjects in group i

Conditionally to the total number of first episodes n=n1+n2, let denote p the proportion of first episodes in group 1,

$$VE = 1 - \frac{n1}{n} * \frac{n}{n - n1} = 1 - p * \frac{1}{1-p} = 1 - \frac{p}{1-p} \qquad (1)$$

where *p=n1/n* is binomial distributed

There is therefore a monotonic link between VE, the true vaccine efficacy, and p, the true proportion of subjects in group 1, among the first episodes.

2. Null hypothesis expressed in terms of a proportion and derived sample size

Considering a 1:1 randomization ratio, the null hypothesis for VE, that is:

H_O: VE vs control ≤ 0%

can be reformulated in terms of the proportion p of 10Pn-PD-DiT vaccinated subjects among the number of subjects with episodes in either the 10Pn-PD-DiT or the control group, as:

H_O: VE ≤ 0

Using definition (1) of the VE,

H_O: $1 - \frac{p}{1-p} \leq 0$

H_O: $1 - p - p \leq 0$

H_O: $p \geq \frac{1}{2}$ (inversion of the sign because division by a number < 0)

Thus, in terms of p, the null hypothesis can be written as

H_O: p ≥ 0.5

This reformulated hypothesis was used to derive the sample size.

A total number of ***535*** ~~1045~~ subjects with evaluable episodes will provide ~~at least~~ 90% power to demonstrate that VE is at least equal to 0%, assuming that the true VE is equal to ***25%*** ~~20%~~ or that p is equal to ***0.4286*** ~~0.444~~ (PASS 2005 – One Proportion Power Analysis, one-sided test, nominal type I error of ***2.5%*** ~~1.19%~~). **(Amended 09 September2010)**

Appendix K Amendments and Administrative Changes to the Protocol

**GlaxoSmithKline Biologicals**<br><br>Clinical Research & Development<br>**Protocol Amendment Approval**	
**eTrack study number**	109563
**eTrack abbreviated title**	10PN-PD-DIT-028
**Protocol title:**	Clinical Otitis Media and Pneumonia Study (**COMPAS**): a phase III , double-blind, randomized, controlled, multicentre study to demonstrate the efficacy of GlaxoSmithKline (GSK) Biologicals' 10-valent pneumococcal conjugate vaccine (GSK1024850A) against Community Acquired Pneumonia (CAP) and Acute Otitis Media (AOM)
**Amendment number:**	Amendment 1
**Amendment date:**	November 2007
**Co-ordinating author:**	Thomas Moens, Scientific Writer
**Rationale/background for changes: Development of the Amendment 1 of the COMPAS protocol was due to: (1) the addition of study centres in Colombia, (2) the implementation of a new subset ('Additional immuno' subset, from which 2 blood samples will be taken at Visit 4 and 7) of subjects in Panama for further exploration after study end of the correlation between protection against AOM episodes caused by (Non-typeable) Haemophilus influenzae and results of the measurement and characterization of responses induced by the PD carrier protein, (3) the change of Reference Lab from Instituto Malbrán in Argentina to Eurofins in US, which implied adaptations to the lab procedures, especially in Appendix H, and (4) the addition of an Appendix (Appendix I) about the background of Acute Otitis Media severity scales and their application in COMPAS.**	

Amended text which has been included or changed was written in *bold italics* in the following sections:

Synopsis: secondary objectives

- **To assess the immune response to the 10Pn-PD-DiT vaccine, one month after dose 3, before, one month and approximately 8 months after the booster dose in terms of serotype specific ELISA antibody concentrations, serotype specific opsonophagocytic activity and anti-PD antibody responses (in a subset of 500 children in ~~each participating country~~*Argentina and Panama*, totaling 1,000)**
- **To assess the reactogenicity of the 10Pn-PD-DiT vaccine in terms of solicited general and local symptoms occurring within the 4-day period after each study vaccine dose (in a subset of 500 children in ~~each participating country~~*Argentina and Panama*, totaling 1,000)**

Synopsis: secondary endpoints
Reactogenicity (in the 'Immuno and reacto' subset of 1,000 subjects (500 ~~per country~~ *subjects respectively in Argentina and Panama*)):
Immunogencity (in the 'Immuno and reacto' subset of 1,000 subjects (500 ~~per country~~ *subjects respectively in Argentina and Panama*)):

Synopsis: study design

- **T*hree*~~wo~~ subsets of subjects will be defined:**
  - **'Immuno and reacto' subset: 500 subjects ~~per country~~*in Panama and Argentina,* totaling 1,000,**
  - **'Carriage' subset: a subset of 2,000 subjects of all subjects enrolled in Panama**
  - ***'Additional immuno' subset: a subset of 3,500 subjects (not participating in the 'Carriage' subset) of all subjects enrolled in Panama***

List of abbreviations:
CHMP Committee for Human Medicinal Products
LPP Local Protocol Procedures

1.2 Haemophilus influenzae
Haemophilus influenzae plays also an important role in the colonization of the nasopharynx. In countries worldwide, between 30 and 50% of children around 2 years of age are colonized with this pathogen (García-Rodríguez et al, 2002). The reported rates of bacterial acquisition and carriage vary extensively between different studies and geographical areas. These differences have been related to genetic background variables and socio-economic conditions including housing, access to health care, poor hygiene, family size, overcrowded living conditions, day-care contact, number of siblings, etc..

1.7 Immunological correlates of protection

At present, widely accepted immunological correlates of protection exist for certain antigens only and consist of defined humoral antibody responses above which there is a high likelihood of protection in the absence of any host factors that might increase susceptibility to the infectious agent (CHMP, 2005).

In POET GSK Biologicals' 11-valent pneumococcal PD-conjugate vaccine was demonstrated to provide 35.6% [3.8-57.0] protection against AOM episodes caused by Non-typeable Haemophilus influenzae (Prymula, 2006). In addition, a statistically significant reduction of 42.6% [1.3-66.6] was observed in nasopharyngeal carriage of H. influenzae. These findings are of clinical significance in view of the importance of this organism as a major cause of AOM and lower respiratory tract infections, and given the predominant role taken by NTHi as cause of recurrent or refractory AOM in the US since introduction of Prevenar.

Based on these observations and following the CHMP recommendations (CHMP, 2005),an effort should be made to describe the correlation between the immune response against Protein D and the protective efficacy against H. influenzae. In POET, a post-primary vaccination blood sample was taken from all 4,968 subject enrolled in the study, for the purpose of exploring such a correlation. Apart from the protocol defined randomly selected immunogenicity subset of approximately 300 subjects, serum samples were not tested, but stored for further evaluation in the context of the assessment of the correlate of protection

After the POET study end and database unblinding, blood samples from the approximately 100 subjects that developed an AOM episode caused by NTHi, were further evaluated to characterize the immune response against protein D. No correlate could however so far be identified that would predict protection against NTHi AOM espisodes or H. influenzae carriage based on evaluation of anti-PD immune responses following primary vaccination only.

Ideally, confirmation of an immunological correlate for protection (at least in the short-term) should be based on exploration of immune responses in at least a subset of vaccinees during clinical studies of protective efficacy. Evaluation and characterization of the responses induced by the PD carrier protein before and after booster vaccination are considered necessary to further attempt fully understanding the relationship with the protection provided against NTHi. Therefore, the study design foresees a subset of subjects in which pre- and post-booster vaccination blood samples will be drawn and stored for further exploration after study end of the correlation between protection against AOM episodes caused by (Non-typeable) Haemophilus influenzae and results of the measurement and characterization of responses induced by the PD carrier protein.

1.5 Rationale for the study

In addition to pneumonia, the pneumococcus causes a variety of other conditions including sometimes lifethreatening invasive pneumococcal diseases such as meningitis and severe bacteraemia, or usually less serious but extremely frequent acute otitis media. For these reasons, COMPAS is designed to demonstrate the efficacy of GSK Biological's 10Pn-PD-DiT pneumococcal conjugate vaccine against "likely bacterial" CAP (B-CAP) in Argentina, *Colombia* and Panama or against clinically confirmed AOM cases (C-AOM) in subjects enrolled in Panama. In addition, impact on CAP with alveolar consolidation or pleural effusion on chest x-ray (C-CAP), bacteriologically confirmed AOM and ID caused by *S. pneumoniae* and *H. influenzae* will be evaluated. . *Finally the correlation will be explored between protection against AOM caused by (Non-typeable) Haemophilus influenzae and the measurement and characterization of responses induced by the PD carrier protein.*

1.6 Likely bacterial CAP definition

From these analyses, the CRP criterion (≥40 mg/L) appears to be the most promising in terms of providing a high sensitivity (high VAR and therefore increased power to demonstrate efficacy in a vaccine study) and maintaining a high specificity (high vaccine efficacy (VE)). It would also reduce the chance of underestimating the burden of pneumococcal pneumonia that is preventable with a pneumococcal conjugate vaccine [Madhi, 2007]. Therefore, an endpoint called "likely bacterial CAP" (defined as radiologically confirmed CAP cases with either alveolar consolidation/pleural effusion or with non-alveolar infiltrates but with CRP ≥ 40mg/L), was chosen to measure the efficacy of the 10Pn-PD-DiT vaccine in this study.

The extension of the alveolar consolidated CAP case definition by the addition of X-ray confirmed CAP cases without alveolar consolidation but with increased CRP was evaluated by Madhi [2007]. Results confirm that the "likely bacterial CAP" endpoint allows better description of the overall reduction of lower respiratory tract infections following pneumococcal vaccination, while maintaining specificity and therefore vaccine efficacy (Table 3).

Procalcitonin (PCT, at a low threshold (≥ 0.25 ng/mL) has shown to be useful in directing the use of antibiotics in adults with pneumonia [Christ-Crain et al, 2004], and in a recent meta-analysis it showed to offer some advantages over CRP for discriminating bacterial from non-bacterial infections [Simon et al, 2004]. Recent observations [Madhi et al, 2005a] demonstrated that many children with viral associated pneumonia have a bacterial superinfection; however high levels of CRP and PCT may be associated with bacterial co-infection. Therefore, although not taken into account for the Likely bacterial CAP definition, serum from each suspected CAP case will be analysed to measure procalcitonin levels.

2.2 Secondary objectives

- **To assess the immune response to the 10Pn-PD-DiT vaccine, one month after dose 3, before, one month and approximately 8 months after the booster dose in terms of serotype specific ELISA antibody concentrations, serotype specific opsonophagocytic activity and anti-PD antibody responses (in a subset of 500 children in ~~*each participating country*~~*Argentina and Panama*, totaling 1,000)**
- **To assess the reactogenicity of the 10Pn-PD-DiT vaccine in terms of solicited general and local symptoms occurring within the 4-day period after each study vaccine dose (in a subset of 500 children in ~~*each participating country*~~*Argentina and Panama*, totaling 1,000)**

3.1 Study design

Figure 3 General study design

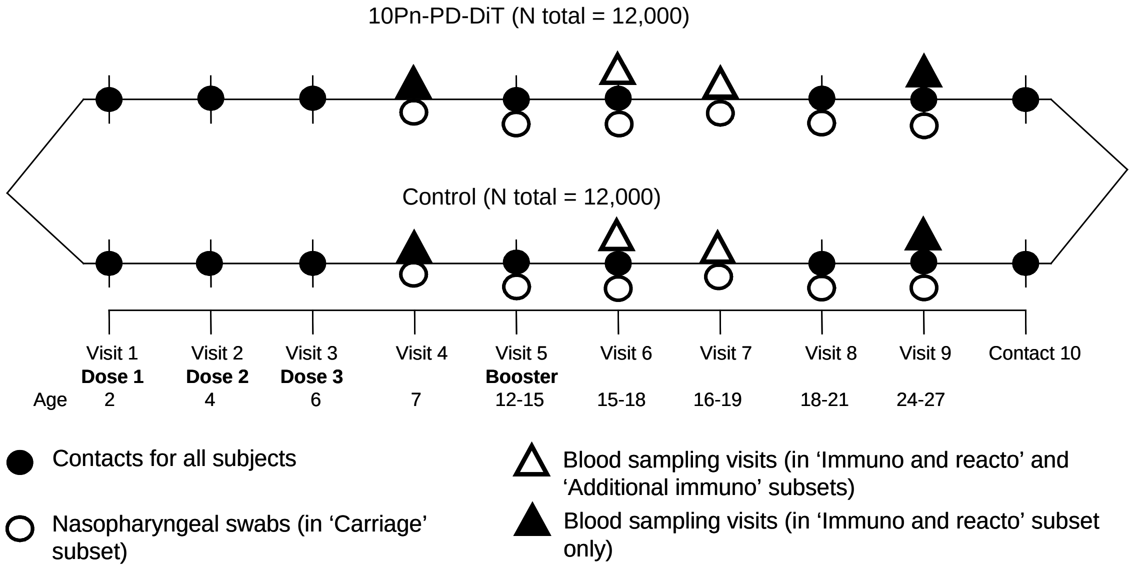

- **The trial will be conducted in Argentina, *Colombia* and Panama.**
- **A MMR vaccine will be administered as a concomitant vaccine to all subjects in *the three*~~both~~ countries at Visit 5 (12-15 months of age), as per local recommendations and country availability.**
- **HAV vaccine: ~~*to comply with local recommendations (one dose of Hep A vaccine by the age of 12 months in Argentina and two doses of Hep A vaccine at 12 and 18 months in Panama) a*~~All subjects will receive 2 doses of HAV vaccine, the first dose at 12 months of age, the second dose at 18-21 months of age. The last HAV dose, given at 18-21 months of age, should be given at least 28 days after the study vaccine booster dose, because the control group also receives a HAV dose at 15-18 months of age as part of the blinded study vaccines. *These vaccine administrations cover the local hepatitis A vaccine recommendations (one dose of Hep A vaccine by the age of 12 months in Argentina and two doses of hepatitis A vaccine at 12 and 18 months in Colombia and Panama).***
- **In *Colombia and* Panama, licensed Varicella vaccine will be offered to all subjects (also subjects in the 10Pn-PD-DiT vaccine group) at 12 months of age in order to provide a benefit to the control group.**
- **A nested study population of approximately 1,000 subjects (500 in *respectively Argentina and Panama*~~*each country*~~) ('Immuno and reacto' subset) will contribute to the assessment of solicited symptoms and the assessment of vaccine immunogenicity.**
- ***'Additional immuno' subset: a subset of approximately 3,500 children (not participating in the 'Carriage' subset) in Panama will have two blood samples taken: just before the booster injection (Visit 6 at 15-18 months of age) and one month post booster (Visit 7 at 16-19 months of age), to be stored and used after study end for exploring the correlation of protein D related responses with occurrence of AOM caused by non-typable Haemophilus influenzae (NTHi).***

3.2 Detailed vaccination scheme

In addition to these blinded study vaccines, the following vaccines will be administered or are recommended in Agentina, *Colombia* and Panama:

Hepatitis B vaccination at birth is recommended in *the three*~~both~~ countries

MMR vaccine is recommended in *the three*~~both~~ countries at 12 months of age. According to these recommendations MMR vaccine provided by the local Expanded Program on Immunization (EPI) will be administered to all subjects at 12 months of age.

To comply with local recommendations for hepatitis A vaccination (one dose of HAV vaccine by the age of 12 months in Argentina and two doses of HAV vaccine at 12 and 18 months of age in *Colombia and* Panama , all subjects will receive 2 doses of HAV vaccine, the first dose at 12 months of age, and the second dose at 18-21 months of age. The last HAV dose should be given at least 28 days after the study vaccine booster dose, because the control group also receives a HAV dose at 15-18 months of age as part of the blinded study vaccines.

In *Colombia and* Panama, licensed Varicella vaccine will be offered to all subjects (also subjects in the 10Pn-PD-DiT vaccine group) at 12 months of age in order to provide a benefit to the control group**.**

4.1 Number of subjects/centres

For all study centers together, the target enrolment will be 24,000 healthy male and female infants between 6 and 16 weeks of age *(between 42 and 118 days)* to be enrolled over a 12 month period in order to obtain 21,600 evaluable subjects (10,800 in both the 10Pn-PD-DiT group and the control group) for the ATP efficacy analysis.

4.2 Inclusion criteria for enrolment

- **A male or female between, and including, 6 and 16 weeks of age *(between 42 and 118 days)* at the time of the first vaccination. Pre-term born infants can be included in the study starting from 8 weeks of chronological age at the time of first vaccination *and up to 16 weeks of chronological age (between 56 and 118 days)*.**
- **In each site, subjects should be living in the area covered by the surveillance system for CAP, ID and AOM (see country specific ~~IO~~*LP*Ps).**

5.4.1 Scheduled visits and study procedures for all subjects

Table 4 List of study procedures applicable for all subjects

Study visit<br><br>Age of subject<br><br>Study timing	VISIT 1<br><br>6-16 weeks<br>Month 0	VISIT 2<br><br>± 4 months<br>Month 2	VISIT 3<br><br>± 6 months<br>Month 4	VISIT 4[1]<br><br>± 7 months<br>Month 5	VISIT 5<br><br>12-15 months<br>Month 10-13	VISIT 6<br><br>15-18 months<br>Month 13-16	VISIT 7[1]<br><br>16-19 months<br>Month 14-17	VISIT 8<br><br>18-21 months<br>Month 16-19	VISIT 9<br><br>24-27 months<br>Month 22-25	CONTACT 10<br><br><br>Study end
Informed consent	●									
Check inclusion criteria	●									
Check exclusion criteria	●									

Study visit<br>Age of subject<br>Study timing	VISIT 1<br>6-16 weeks<br>Month 0	VISIT 2<br>± 4 months<br>Month 2	VISIT 3<br>± 6 months<br>Month 4	VISIT 4[1]<br>± 7 months<br>Month 5	VISIT 5<br>12-15 months<br>Month 10-13	VISIT 6<br>15-18 months<br>Month 13-16	VISIT 7[1]<br>16-19 months<br>Month 14-17	VISIT 8<br>18-21 months<br>Month 16-19	VISIT 9<br>24-27 months<br>Month 22-25	CONTACT 10<br><br>Study end
Demography, including gestational age	●									
Medical history	●									
Pre-vaccination history	●									
Check elimination criteria		●	●		●	●		●	●	
Check risk factors	●				●				●	
Check warnings, precautions and contraindications	O	O	O		O	O		O		
Physical examination	●	●	●		●	●		●	●	
Weight/height recording	●	●	●			●			●	
Pre-vaccination body temperature	●	●	●			●				
Randomization	●									
Vaccination (study and co-administered vaccines)	●	●	●			●				
Vaccination (concomitant vaccines)[2]					●			●		
Record any concomitant vaccination	●	●	●		●	●		●	●	
Reporting of unsolicited Adverse Events[3]		●	●		●	●		●	●	
Reporting of convulsions[4]								●		
Reporting of Serious Adverse Events		●	●		●	●		●	●	●
Study Conclusion										●

Note:
● is used to indicate a study procedure that requires documentation in the individual CRF (eCRF).
O is used to indicate a study procedure that does not require documentation in the individual eCRF.
[1]Study activities during grey-coloured visits are only applicable for subjects in the 'Immuno and reacto' and/or the 'Carriage' subsets (see Table 5 and 7, respectively)
[2]Concomitant vaccines: in addition to the blinded study vaccines, subjects will be vaccinated with *(1)* MMR at the age of 12-15 months (according to local EPI), and *(2)* with HAV vaccine at the age of 12-15 months (visit 5) and at 18-21 months of age (since subjects in the control group receive a dose of HAV vaccine at visit 6, the last HAV dose should be administered at least 28 days after the stuy vaccine booster dose) *(study benefit= vaccination against HAV at 18-21 months of age in Argentina and at 12-15 and 18-21 months of age in Panama and Colombia)*. *(3)* In Argentina all subjects will be vaccinated at 12-15 months of age against *Neisseria meningitidis* group C and in *Colombia and* Panama all subjects will be vaccinated against varicella at 12-15 months of age (study benefit= *vaccination against Neisseria in Argentina and against varicella in Colombia and Panama*).
[3]Only applicable for subjects enrolled in Panama
[4]*Applicable for subjects enrolled in Argentina, Colombia and Panama*

Some children will be allocated to one of *threetwo* subsets (see Table 6,*Table 8* and Table 10). The subsets will differ by the number and nature of the study assessments made during the course of the study as described below.

5.4.2 Scheduled visits and study procedures for 'Immuno and reacto' subset
Subjects enrolled in the 'Immuno and reacto' subset will have the same seven scheduled visits and final study conclusion contact as all subjects plus two additional scheduled visits (see Figure 3 and Table 6). A group of 1,000 subjects (500 *respectively in Argentina and Panama*~~per country~~ will be part of this subset.

Table 6 List of study procedures for subjects participating in the 'Immuno and reacto' subset of the study

Study visit<br>Age of subject<br>Study timing<br>Blood sampling timepoints	VISIT 1<br>6-16 weeks<br>Month 0	VISIT 2<br>± 4 months<br>Month 2	VISIT 3<br>± 6 months<br>Month 4	VISIT 4<br>± 7 months<br>Month 5<br>Post III (M5)	VISIT 5<br>12-15 months<br>Month 10-13	VISIT 6<br>15-18 months<br>Month 13-16<br>Post III (M13)	VISIT 7<br>16-19 months<br>Month 14-17<br>Post IV (M14)	VISIT 8<br>18-21 months<br>Month 16-19	VISIT 9<br>24-27 months<br>Month 22-25<br>Post IV (M22)	CONTACT 10<br>Study end
Informed consent	●									
Check inclusion criteria	●									
Check exclusion criteria	●									
Demography, including gestational age	●									
Medical history	●									
Pre-vaccination history	●									
Check elimination criteria		●	●	●	●	●	●	●	●	
Check risk factors	●				●				●	
Check warnings, precautions and contraindications	O	O	O		O	O		O		
Physical examination	●	●	●	●	●	●	●	●	●	
Weight/height recording	●	●	●			●			●	
Pre-vaccination body temperature	●	●	●			●				
Randomization	●									
Blood sampling: for antibody determination (3.5 ml)				●		●	●		●	
Vaccination (study and co-administered vaccines)	●	●	●			●				
Vaccination (concomitant vaccines)[1]					●			●		
Return of diary cards		O	O	O			O			
Diary card transcription		●	●	●			●			

Study visit<br>Age of subject<br>Study timing<br>Blood sampling timepoints	VISIT 1<br>6-16 weeks<br>Month 0	VISIT 2<br>± 4 months<br>Month 2	VISIT 3<br>± 6 months<br>Month 4	VISIT 4<br>± 7 months<br>Month 5<br>Post III (M5)	VISIT 5<br>12-15 months<br>Month 10-13	VISIT 6<br>15-18 months<br>Month 13-16<br>Post III (M13)	VISIT 7<br>16-19 months<br>Month 14-17<br>Post IV (M14)	VISIT 8<br>18-21 months<br>Month 16-19	VISIT 9<br>24-27 months<br>Month 22-25<br>Post IV (M22)	CONTACT 10<br><br>Study end
Daily post-vaccination recording of solicited symptoms (Days 0-3) by subjects' parents/guardians	●	●	●			●				
Record any concomitant vaccination	●	●	●	●	●	●	●	●	●	
Reporting of unsolicited Adverse Events[2]		●	●	●	●	●	●	●	●	
Reporting of convulsions								●		
Reporting of Serious Adverse Events		●	●	●	●	●	●	●	●	●
Study Conclusion										●

Note:
● is used to indicate a study procedure that requires documentation in the individual CRF (eCRF).
O is used to indicate a study procedure that does not require documentation in the individual eCRF.
[1]Concomitant vaccines: in addition to the blinded study vaccines, subjects will be vaccinated with *(1)* MMR at the age of 12-15 months (according to local EPI), and *(2)* with HAV vaccine at the age of 12-15 months (visit 5) and at 18-21 months of age (since subjects in the control group receive a dose of HAV vaccine at visit 6, the last HAV dose should be administered at least 28 days after the stuy vaccine booster dose) *(study benefit= vaccination against HAV at 18-21 months of age in Argentina and at 12-15 and 18-21 months of age in Colombia and Panama). (3)* In Argentina all subjects will be vaccinated at 12-15 months of age against *Neisseria meningitidis* group C and in *Colombia and* Panama all subjects will be vaccinated against varicella at 12-15 months of age (study benefit= *vaccination against Neisseria in Argentina and against varicella in Colombia and Panama*).
[2]Only applicable for subjects enrolled in Panama

5.4.3 Scheduled visits and study procedures for 'Additional immuno' subset

Subjects enrolled in the 'Immuno' subset will have the same seven scheduled visits and final study conclusion contact as all subjects plus two additional scheduled visits (see Figure 4 and Table 8). A group of 3,500 subjects in Panama will be part of this subset. A first blood sample will be obtained at Visit 6, just before the booster injection, while the second blood sample will be taken one month after the administration of the booster vaccination (at visit 7).

Study design of the 'Additional immuno' subset

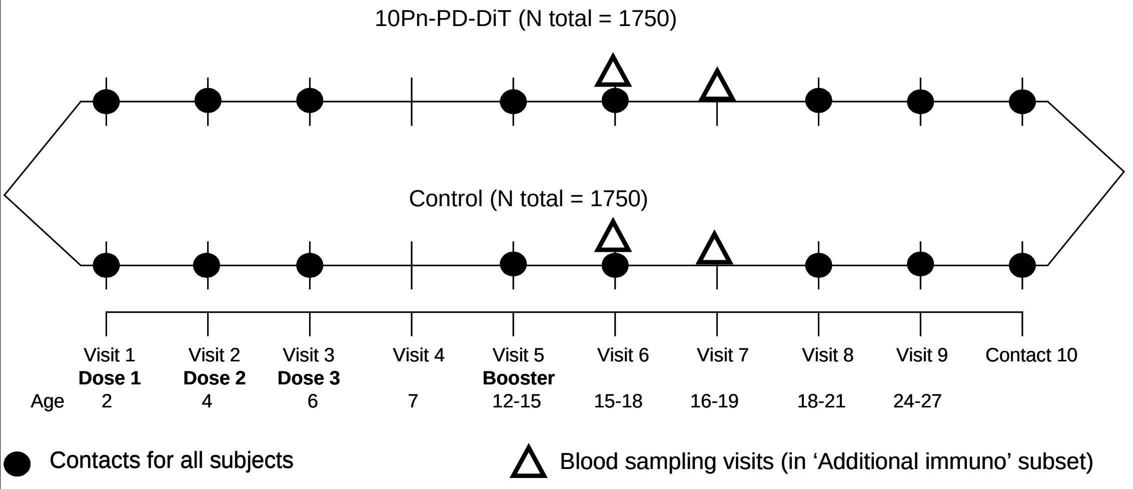

Note: although age windows for different visits sometimes overlap, study procedures for different visits can not be grouped at one visit, and intervals between visits as defined in Table 9 must be respected.

Table 8 List of study procedures for subjects participating in the 'Additional immuno' subset of the study

Study visit	VISIT 1	VISIT 2	VISIT 3	VISIT 4	VISIT 5	VISIT 6	VISIT 7	VISIT 8	VISIT 9	CONTACT 10
Age of subject	6-16 weeks	± 4 months	± 6 months	± 7 months	12-15 months	15-18 months	16-19 months	18-21 month s	24-27 months	
Study timing	Month 0	Month 2	Month 4	Month 5	Month 10-13	Month 13-16	Month 14-17	Month 16-19	Month 22-25	Study end
Blood sampling timepoints				Post III (M5)		Post III (M13)	Post IV (M14)		Post IV (M22)	
Informed consent	●									
Check inclusion criteria	●									
Check exclusion criteria	●									
Demography, including gestational age	●									
Medical history	●									
Pre-vaccination history	●									
Check elimination criteria		●	●	●	●	●	●	●	●	

Study visit	VISIT 1	VISIT 2	VISIT 3	VISIT 4	VISIT 5	VISIT 6	VISIT 7	VISIT 8	VISIT 9	CONTACT 10
Age of subject	6-16 weeks	± 4 months	± 6 months	± 7 months	12-15 months	15-18 months	16-19 months	18-21 month s	24-27 months	
Study timing	Month 0	Month 2	Month 4	Month 5	Month 10-13	Month 13-16	Month 14-17	Month 16-19	Month 22-25	Study end
Blood sampling timepoints				Post III (M5)		Post III (M13)	Post IV (M14)		Post IV (M22)	
Check risk factors (Amended 26 November 2007)	●				●				●	
Check warnings, precautions and contraindications	O	O	O		O	O		O		
Physical examination	●	●	●	●	●	●	●	●	●	
Weight/height recording	●	●	●			●			●	
Pre-vaccination body temperature	●	●	●			●				
Randomization	●									
Blood sampling: for antibody determination (3.5 ml)						●	●			
Vaccination (study and co-administered vaccines)	●	●	●			●				
Vaccination (concomitant vaccines)[1]					●			●		
Record any concomitant vaccination	●	●	●	●	●	●	●	●	●	
Reporting of unsolicited Adverse Events		●	●	●	●	●	●	●	●	
Reporting of convulsions								●		
Reporting of Serious Adverse Events		●	●	●	●	●	●	●	●	●
Study Conclusion										●

Note:
● is used to indicate a study procedure that requires documentation in the individual CRF (eCRF).
O is used to indicate a study procedure that does not require documentation in the individual eCRF.
[1]Concomitant vaccines: in addition to the blinded study vaccines, subjects will be vaccinated with (1) MMR at the age of 12-15 months (according to local EPI, and (2) with HAV vaccine at the age of 12-15 months (visit 5) and at 18-21 months of age (since subjects in the control group receive a dose of HAV vaccine at visit 6, the last HAV dose should be administered at least 28 days after the stuy vaccine booster dose) (study benefit= vaccination against HAV at 18-21 months of age in Argentina and at 12-15 and 18-21 months of age in Colombia and Panama). (3) In Argentina all subjects will be vaccinated at 12-15 months of age against Neisseria meningitidis group C and in Colombia and Panama all subjects will be vaccinated against varicella at 12-15 months of age (study benefit= vaccination against Neisseria in Argentina and against varicella in Colombia and Panama).

It is the investigator's responsibility to ensure that the intervals between visits/contacts are strictly followed. These intervals determine each subject's evaluability in the according to protocol analyses (see Sections 9 and 9.4 for details of criteria for evaluability and cohorts to be analyzed).

Table 9 Intervals between study visits for the 'Additional immuno' subset

Interval	*Size of interval*
Visit 1→Visit 2	*49-83 days*
Visit 2→Visit 3	*49-83 days*
Visit 3→Visit 4	*28-42 days*
Visit 5	*12-15 months of age*
Visit 5→Visit 6	*≥ 28 days*
Visit 6	*15-18 months of age*
Visit 6→Visit 7	*28-42 days*

5.4.4 Scheduled visits and study procedures for 'Carriage' subset

Table 10 List of study procedures for subjects participating in the 'Carriage' subset

Study visit<br>Age of subject<br>Study timing<br>Swab sampling timepoint	VISIT 1<br>6-16 weeks<br>Month 0	VISIT 2<br>± 4 months<br>Month 2	VISIT 3<br>± 6 months<br>Month 4	VISIT 4<br>± 7 months<br>Month 5<br>Post III (M5)	VISIT 5<br>12-15 months<br>Month 10-13<br>Post III (M10)	VISIT 6<br>15-18 months<br>Month 13-16<br>Post III (M13)	VISIT 7<br>16-19 months<br>Month 14-17<br>Post IV (M14)	VISIT 8<br>18-21 months<br>Month 16-19<br>Post IV (M16)	VISIT 9<br>24-27 months<br>Month 22-25<br>Post IV (M22)	CONTACT 10<br>Study end
Informed consent	●									
Check inclusion criteria	●									
Check exclusion criteria	●									
Demography, including gestational age	●									
Medical history	●									
Pre-vaccination history	●									
Check elimination criteria		●	●	●	●	●	●	●	●	
Check risk factors	●				●				●	
Check warnings, precautions and contraindications	O	O	O		O	O		O		
Physical examination	●	●	●	●	●	●	●	●	●	
Weight/height recording	●	●	●			●			●	
Pre-vaccination body temperature	●	●	●			●				
Randomization	●									
Nasopharyngeal swab samples				●	●	●	●	●	●	
Vaccination (study and co-administered vaccines)	●	●	●			●				
Vaccination (concomitant vaccines)[1]					●			●		

Study visit<br>Age of subject<br>Study timing<br>Swab sampling timepoint	VISIT 1<br>6-16 weeks<br>Month 0	VISIT 2<br>± 4 months<br>Month 2	VISIT 3<br>± 6 months<br>Month 4	VISIT 4<br>± 7 months<br>Month 5<br>Post III (M5)	VISIT 5<br>12-15 months<br>Month 10-13<br>Post III (M10)	VISIT 6<br>15-18 months<br>Month 13-16<br>Post III (M13)	VISIT 7<br>16-19 months<br>Month 14-17<br>Post IV (M14)	VISIT 8<br>18-21 months<br>Month 16-19<br>Post IV (M16)	VISIT 9<br>24-27 months<br>Month 22-25<br>Post IV (M22)	CONTACT 10<br><br>Study end
Record any concomitant vaccination	●	●	●	●	●	●	●	●	●	
Record any antibiotic treatment (diary card)		●	●	●	●	●	●	●	●	
Reporting of unsolicited Adverse Events		●	●	●	●	●	●	●	●	
Reporting of convulsions								●		
Reporting of Serious Adverse Events		●	●	●	●	●	●	●	●	●
Study Conclusion										●

Note:
● is used to indicate a study procedure that requires documentation in the individual CRF (eCRF).
O is used to indicate a study procedure that does not require documentation in the individual eCRF.
[1]Concomitant vaccines: in addition to the blinded study vaccines, subjects will be vaccinated with *(1)* MMR at the age of 12-15 months (according to local EPI), and *(2)* with HAV vaccine at the age of 12-15 months (visit 5) and at 18-21 months of age (since subjects in the control group receive a dose of HAV vaccine at visit 6, the last HAV dose should be administered at least 28 days after the stuy vaccine booster dose) *(study benefit= vaccination against HAV at 18-21 months of age in Argentina and at 12-15 and 18-21 months of age in Colombia and Panama)*. *(3)* In Argentina all subjects will be vaccinated at 12-15 months of age against *Neisseria meningitidis* group C and in *Colombia and* Panama all subjects will be vaccinated against varicella at 12-15 months of age (study benefit= *vaccination against Neisseria in Argentina and against varicella in Colombia and Panama*).

5.5.1.1 Scheduled visits to be performed
Visit 1: At 6-16 weeks of age *(between 42 and 118 days)*
Study procedures for all subjects
- ***Check risk factors***

Visit 4: at 7 months of age; Study month 5
'IMMUNO AND REACTO' or 'CARRIAGE' SUBSETS ONLY
Study procedures for 'IMMUNO and REACTO' SUBSET
- **Collection of a blood sample for serology: ~~*4.0 ml (*~~at least 3.5 ml*)* of whole blood.**

Visit 5: at 12-15 months of age; Study Month 10-13
Study procedures for all subjects
- ***Check risk factors***

Visit 6: at 15-18 months of age; Study Month 13-16
Additional study procedures for SUBJECTS IN PANAMA
The completed diary card will be given to the investigator at Visit 7 if the subject is also part of the 'Immuno and reacto', ~~*or*~~ 'Carriage' *and 'Additional immuno'* subsets in Panama and at Visit 8 otherwise.

Additional study procedures for 'IMMUNO AND REACTO' SUBSET
Collection of a blood sample for serology: ~~*4.0 ml (*~~at least 3.5 ml*)* of whole blood.

Additional study procedures for subjects part of the 'Additional immuno' subset in Panama
- ***Check elimination criteria***
- ***Physical examination***
- ***Collection of a blood sample for serology: at least 3.5 ml of whole blood. The sample will be centrifuged and the serum collected (see Section 5.6). The serum will be stored at -20°c before transfer to GSK Biologicals for the laboratory assays (see Section 5.7).***
  When materials are provided by GSK Biologicals, it is MANDATORY that all clinical samples (including serum samples) will be collected and stored using exclusively those materials in the appropriate manner. The use of other materials could result in the exclusion of the subject from the ATP analysis (See Section 9.4 for definition of study cohorts to be evaluated). The investigator must ensure that his/her personnel and the laboratory(ies) under his/her supervision comply with this requirement. However, when GSK Biologicals does not provide material for collecting and storing clinical samples, then appropriate materials from the investigator's site are to be used. Refer to Appendix C and Appendix D.

Visit 7: at 16-19 months of age; Study Month 14-17
IMMUNO AND REACTO', *'ADDITIONAL IMMUNO' AND* ~~or~~ 'CARRIAGE' SUBSETS ONLY

Study procedures for 'IMMUNO AND REACTO' SUBSET

- **Collection of a blood sample for serology: ~~*4.0 ml (*~~at least 3.5 ml~~*)*~~ of whole blood.**

Study procedures for 'ADDITIONAL IMMUNO' subset

- ***Check elimination criteria***
- ***Physical examination***
- ***Collection of a blood sample for serology: at least 3.5 ml of whole blood. The sample will be centrifuged and the serum collected (see Section 5.6). The serum will be stored at -20°c before transfer to GSK Biologicals for the laboratory assays (see Section 5.7).***
  When materials are provided by GSK Biologicals, it is MANDATORY that all clinical samples (including serum samples) will be collected and stored using exclusively those materials in the appropriate manner. The use of other materials could result in the exclusion of the subject from the ATP analysis (See Section 9.4 for definition of study cohorts to be evaluated). The investigator must ensure that his/her personnel and the laboratory(ies) under his/her supervision comply with this requirement. However, when GSK Biologicals does not provide material for collecting and storing clinical samples, then appropriate materials from the investigator's site are to be used. Refer to Appendix C and Appendix D.
- ***Record any concomitant vaccination (see Section 6.11)***
- ***Record any serious adverse event that may have occurred since previous visit.***
- ***The subject's parents/guardians will be instructed to contact the investigator immediately should their child manifest any signs or symptoms they perceive as serious.***

Additional study procedures for SUBJECTS PART OF THE 'IMMUNO AND REACTO'*, 'ADDITIONAL IMMUNO' AND* ~~*or*~~*'*CARRIAGE' SUBSETS IN PANAMA

Visit 8: at 18-21 months of age; Study Month 16-19
Study procedures for all subjects

- ***Reporting of convulsions***

Visit 9: at 24-27 months of age; Study Month 22-25
Study procedures for all subjects

- ***Check risk factors***

Additional study procedures for 'IMMUNO AND REACTO' SUBSET

- **Collection of a blood sample for serology: ~~*4.0 ml (*~~at least 3.5 ml~~*)*~~ of whole blood.**

5.5.2.1.2 Surveillance procedure
For the purpose of the study, suspected pneumonia cases (see definition in Section 5.5.2.1.1) will be captured by qualified study personnel either at the physician's office, the emergency room or the X-ray department. Clinical management of suspected CAP cases will be done according to the local routine practices, including prescription of local laboratory tests and chest X-ray (CXR). *Wheezing diseases (such as bronchiolitis or hyperreactive bronchial syndromes) should not follow the study procedures, unless a suspicion of pneumonia is diagnosed, based on the suspected CAP case definition (refer to Section 5.5.2.1.1.).*

***Digital CXR images from a*~~All~~ ~~*CXR for*~~ suspected CAP cases in study participants will be ~~*digitalized and*~~ sent to the Central Reading Panel for further classification *(refer to X Ray Workbook)*. ~~*For quality appreciation, images will be classified as (1) uninterpretable, (2) suboptimal or (3) adequate.*~~ The reading of the image will be classified as (1) consolidated CAP, (2) ~~*abnormal CXR*~~ *non-consolidated CAP,* ~~*or*~~ (3) normal CXR *or (4) uninterpretable CXR*. For more details about the~~*se*~~ definitions *of radiographic outcomes,* please refer to ~~*Annex 4 [WHO, 2001]*~~ *Appendix F* . All suspected CAP cases occurring in subjects participating in the study will be reported in the eCRF, regardless of the confirmation of the diagnosis by the local radiologist, the treating physician/pediatrician or the Central X-ray Reading Panel of the study.**

- **In addition, whenever possible *and within 5 days after initiation of symptoms,* efforts will be made to collect a nasopharyngeal aspirate sample in any suspected CAP case. Samples will be shipped to the Central Lab to analyse the presence of specific respiratory viruses: adenovirus, RSV, Flu A & B, and para-influenza 1, 2 and 3.**
- **The WBC count and formula and the CRP value of the local lab analysis will be entered in the CAP section of the eCRF if available.**

Please refer to country-specific ~~*IO*~~*LP*Ps for more details.

5.5.2.1.3 Sample handling at local laboratories

Serum samples

Blood sample will be obtained from all suspected CAP cases. The serum sample will be split into two aliquots: one aliquot for local CRP determination (according to local practices) and a second aliquot to be kept at –20°C or *at -70°C* ~~lower~~ until shipment to the Central Lab for quantitative CRP and PCT determination.

Nasopharyngeal aspirate

Whenever possible *and within 5 days after initiation of symptoms*, nasopharyngeal aspirate samples will be obtained from all suspected CAP cases before the initiation of any antibiotic treatment. ~~*The sample will be split into two aliquots: one for local viral diagnosis (according to local practices) and a second one to be prepared for further shipment to the Central Lab*~~ .

***Ideally, a*~~*A*~~ll naso*pharynge*al aspirate samples ~~*will*~~ *should* be stored at -70°C as soon as possible after collection because a loss of infectivity occurs over time, resulting in the diminished likeliehood of a positive result. *However, if immediate delivery to the laboratory is not possible, specimens will be stored in a refrigerator (2-8°C) or placed on wet ice or a cold pack for a maximum of 6 hours.* Loss of viability is slower at refrigerating temperatures than at ambient temperatures; samples containing labile viruses (e.g. respiratory syncytial virus (RSV) at low titers are those most likely to show loss of infectivity with delayed transport. ~~*If immediate delivery to the laboratory is not possible, specimens will be stored in a refrigerator (2-8°C) or placed on wet ice or a cold pack.*~~.**

***The sample will be split into two aliquots and stored at -70°C: one to be prepared for further shipment to the Central Lab, and a second one for future vaccine related testing (refer to Investigator Manual Part I - Biochemistry and Virology for further details on sample collection, handling and storage).* ~~*The aliquot will be stored at –70°C and sent to the Central Lab for further respiratory viral diagnostic testing.*~~**

Freezing at higher temperatures and freeze-thaw cycles ~~*will*~~ *must* be avoided. ~~*Frozen specimens will be transported on sufficient dry ice to ensure that specimens remain frozen until receipt by the laboratory*~~ [Hoberman et al, 1997; Isenberg, 1998].

5.5.2.1.5 Data flow

***Digital CXR images of* ~~*All CXR related to*~~ suspected CAP cases will be ~~*digitalized and*~~ sent to the Central Reading Panel. Results of the interpretations by the Central Reading Panel will be transferred directly to GSK, and will not be sent back to the study investigators, since only the local reading of the CXR according to local routine practices will guide diagnostic and therapeutic decisions *(Refer to COMPAS X Ray Workbook)*.**

5.5.2.1.6 Case accountability

For CAP, the start date of a clinical episode ~~*begins at the time the date the CXR is taken if the first date is not known*~~ *corresponds to the date when symptoms first were observed. In case this date is not known or defined, the start date will be the date when the CXR was taken.*

5.5.2.2.1 Case definitions

A case of clinically confirmed AOM (C-AOM) is defined as a child who meets the above mentioned AOM case definition, as per the judgment of a*n* ~~*pediatric*~~ *ENT* specialist.

5.5.2.2.2 Surveillance procedures

4) If the specialist clinically confirms the presence of AOM, relevant clinical data will be registered in the CRF. The ENT specialist will *assess the severity of the case (refer to Section 5.5.2.2.4) and, based on the results, he/she will decide the following steps. If tympanocentesis will be performed, the ENT specialist will* explain the technique, benefits and potential risks ~~*of tympanocentesis*~~ in depth.

8) An AOM episode will be considered to be bacteriologically confirmed if any of the following bacterial pathogens is identified in the MEF sample: *S. pneumoniae, H. influenzae, M. catarrhalis* and *S. pyogenes*. If *Streptococcus pneumoniae* or *H. influenzae* is isolated at local laboratory, additional bacteriological testing (serotyping and antiobiotic resistance) will be performed at the *Central*~~*Reference*~~ Laboratory ~~*in Argentina*~~. Results from the *Central*~~*Reference*~~ Laboratory will not be sent back to the investigator. Details of the lab procedures performed at the *Central*~~*Reference*~~ lab can be found in Appendix H *and in the Lab Workbook*.

Figure 5 Procedures for follow-up of AOM cases

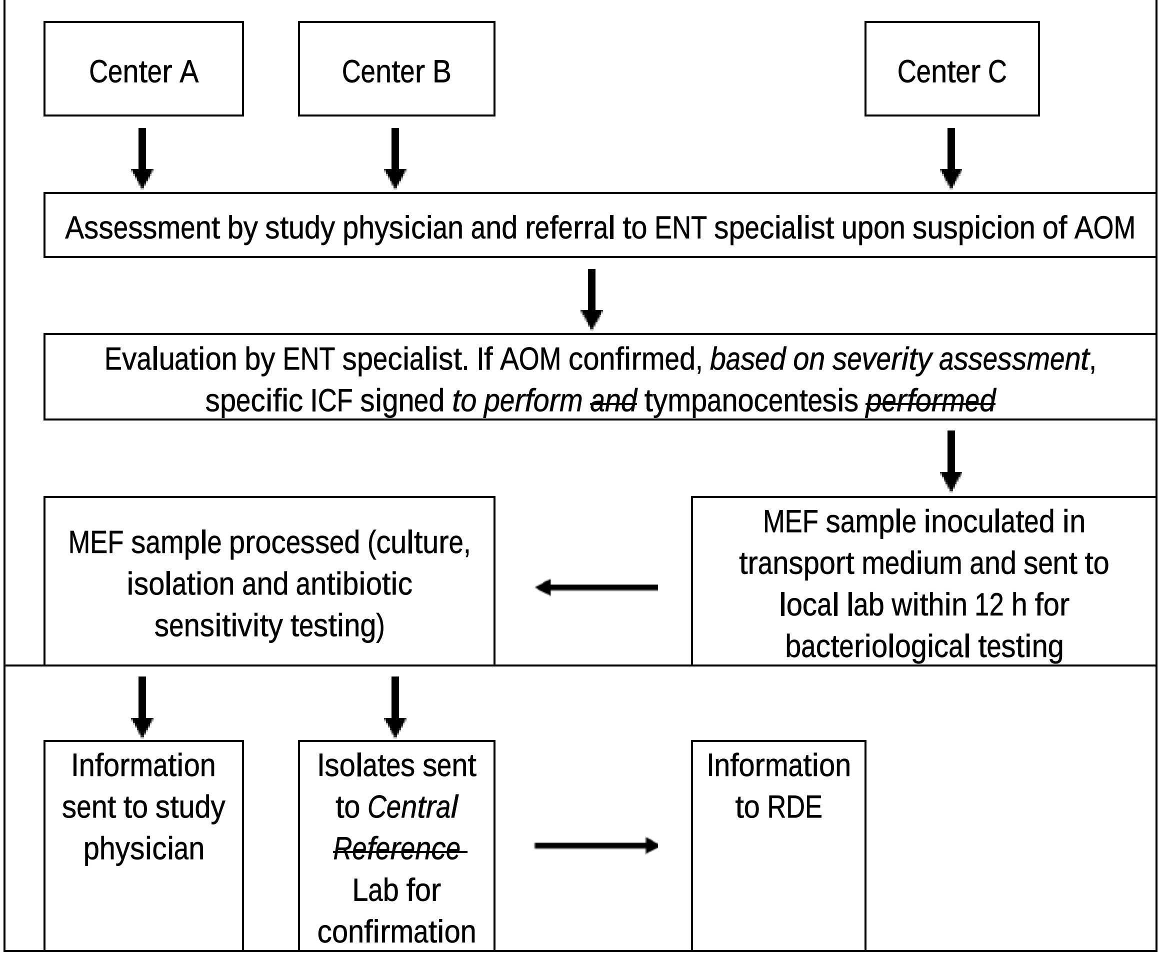

Each unscheduled visit during which acute otitis media is diagnosed will be recorded in the electronic CRF. See Section 5.5.2.2.1 for case definition.

Please refer to country-specific ~~*IO*~~*LP*Ps for more details.

5.5.2.2.3 Sample handling

Middle ear fluid will be collected by tympanocentesis (when tympanic membrane is intact) ~~*or by aspiration*~~, provided parents/guardians agreed with the performance of tympanocentesis via a specific informed consent form. Tympanocentesis can be easily and safely performed using a Channel Directed Tympanocentesis (CTD) speculum (see Appendix G). *In case of spontaneous drainage, MEF will be collected by aspiration using the same CDT device and technique described above.*

***In case of an intact eardrum, t*~~T~~he method is briefly outlined below.**

- ***The use of mask and gloves is highly recommended to avoid contamination of the sample.***

In case of spontaneous/accidental rupture of the tympanic membrane, MEF should be collected by ~~*syringe*~~ aspiration in the ear canal *using the CTD® Aspirator, attached to the CTD® Speculum*. The ear canal should be thoroughly cleaned before the procedure to avoid masking of NTHi by local flora.

~~*Similarly, spontaneous drainage should be collected for culture, using syringe aspiration or swabs as appropriate*~~.

Disposables are provided by GlaxoSmithKline Biologicals.

To obtain best results in the isolation of *S. pneumoniae* and *H. influenzae*, all samples will be inoculated into transport medium *(Amies agar gel without charcoal)* and sent within 12 hours to the local lab for bacteriological testing [Van Horn et al, 1998].

At the local lab, the most representative colonies will be stored in a ~~*skim milk medium or other*~~ cryo-conservation medium *(refer to COMPAS Lab Workbook)* and kept at -70°C.

Isolates will be kept at these conditions until having confirmation of the date of the shipment to the *Central*~~*Reference*~~ Lab and will be subcultured in blood and chocolate agar 3-5 days prior to the shipment. The most representative bacterial colonies will be selected and transferred to Amies-charcoal transport medium and immediately shipped at room temperature to the *Central*~~*Reference*~~ Lab *(refer to Investigator Laboratory Manual - Part II for further details)*.

~~***MEF which was not used for plating will be transferred to Skim milk medium in a vial, and stored at -70°C at the investigator's site. Refer to the country-specific IOP of Panama for a detailed description of bacteriological methods.***~~

5.5.2.2.4 Assessment of severity of AOM

At the primary or study physician's level, the case will be assessed based on the definition of clinically suspected AOM (see Section 5.5.2.2.1). Additionally, severity of each suspected AOM case will be determined according to the following clinical parameters (refer also to Appendix I), and data will be recorded in the specific pages of the eCRF.

- ***Temperature***
- ***Irritability***
- ***Ear ache***
- ***Tugging***
- ***Feeding***
- ***Sleeping***

Based on otoscopy and clinical data previously taken by the primary or study physician, the ENT specialist will determine whether the AOM case is clinically confirmed or not. The following parameters will be reported in the eCRF.

- ***Side of the affected ear***
- ***Presence of redness of tympanic membrane***
- ***Presence of bulging of tympanic membrane***
- ***Presence of spontaneous perforation***
- ***OS 8 Scoring (Refer to Appendix I)***
- ***Macroscopic aspects of MEF (serous, bloody, purulent, other)***
- ***Additional case information per side (report starting date if spontaneous drainage occurred)***
- ~~***Medical records of all clinically suspected AOM cases reported will be inspected by study personnel, and the following clinical parameters will be recorded in the eCRF:***~~

• ~~***Redness, bulging or loss of light reflex of tympanic membrane***~~<br>• ~~***Presence of middle ear fluid effusion***~~<br>• ~~***Ear pain***~~<br>• ~~***Ear discharge***~~<br>• ~~***Hearing loss***~~<br>• ~~***Fever***~~<br>• ~~***Lethargy***~~<br>• ~~***Irritability***~~<br>• ~~***Anorexia***~~<br>• ~~***Vomiting***~~<br>• ~~***Diarrhea***~~<br>• ~~***Chronic supurative otitis media***~~<br>• ~~***Serous otitis media***~~<br>• ~~***Ventilating tube related ottorhea***~~
**5.5.2.2.5 Data flow**<br>**Results of the identification of the MEF isolates will also be transferred to GSK, as well as serotyping and antibiotic sensitivity testing performed at the *Central*~~*Reference*~~ Laboratory. In order to preserve the blinding of the study, the information from the *Central*~~*Reference*~~ Laboratory will ony be made available to the study investigators after the end of the study.**

5.5.2.3.1 Case definitions

- **as probable when no *S. pneumoniae* or *H. influenzae* are isolated from the above mentioned fluids, but when detection of antigens of *S. pneumoniae* and/or *H. influenzae* is positive when using tests such as Latex agglutination or immunochromatographic Binax™ NOW test *(only when meningitis is suspected)*.**

5.5.2.3.2 Surveillance procedures

Cases of invasive disease occurring in study participants will be further documented in the invasive disease section of the eCRF. For each case, *~~including antipyretics taken within 24 hours and~~* antibiotics taken within 3 days prior to Normally Sterile Body Fluid (NSBF) collection will be recorded. If the subject is hospitalized or if the adverse event is considered to be serious (refer to Section 7.2 for the definition of serious adverse events (SAEs)), the event should also be notified to GSK according to the SAE reporting procedures defined in Section 7.6. Any case of meningitis, regardless of the etiology, should be reported *in the SAE Report Form* as a serious adverse event *~~using the SAE Report Form~~ and in the meningitis report form~~, including the appropriate meningitis section~~*, and should be recorded in a specific eCRF page. *In case of a confirmed meningitis, the corresponding pages in the Invasive Disease section, including the Lumbar Puncture section, should be filled in. In case of a probable meningitis, only the Lumbar Puncture section should be filled in.*

The practical organization of the surveillance for invasive diseases caused by *S. pneumonia* or *H. influenzae* in the study subjects will be described in country specific *~~IO~~L*PPs.

5.5.2.3.3 Sample handling

Blood culture

Therefore *~~a~~ remaining* sample of each positive blood culture bottle should be *divided into 3 cryovials (1.5 mL each) and* kept *at -70°C* for possible further analysis *~~in case the bacterial subculture was negative~~* (see *COMPAS* Lab workbook).

Bacterial isolates will be kept in duplicate at a country level in *~~20% skim milk medium or other~~ a* cryo-conservation medium *(refer to COMPAS Lab Workbook) ~~(i.e. CryoBank)~~* at -70°C. Once having the confirmation of the date of the shipment to the *Central~~Reference~~* Lab, isolates will be subcultered in blood and chocolate agar *media* and prepared for transportation in Amies Charcoal medium at room temperature *(see Investigator Laboratory Manual - Part II)*.

The *Central* ~~*Reference*~~ Lab will confirm viability of the strains (log sheet) to the local lab at a country level *(refer to COMPAS Lab Workbook)*. In addition, local requirements for national surveillance programs will be fulfilled. Back-up samples will be destroyed unless local requirements for relevant national surveillance programs define differently.

Other body fluids
If possible, a *remaining* sample of the *original* body fluids should be kept and stored at -70°C until collection by GSK or a GSK-designated laboratory, *even in case the bacterial subculturing resulted negative (see COMPAS Lab Workbook)*.

Other laboratory procedures
Isolation and ~~*antibiotic sensitivity*~~ testing
Positive blood cultures must be sampled within 10 hours after being detected as positive. ~~*Antibiogram will be also performed. Please refer to local IOPs for details on the different procedures*~~ .

Local storage of isolates and transportation to *Central* ~~*Reference*~~ Laboratory
All *S. pneumoniae* and *H. influenzae* strains isolated in the context of the current study in all participating countries will be kept in ~~*20% skim milk medium or other*~~ *a* cryo-conservation medium ~~*(i.e. CryoBank)*~~ *(refer to COMPAS Lab Workbook)* at -70°C until preparation for transport. For this purpose, strains will be subcultured on chocolate and blood agar *media*, the most representative colonies will be selected, transferred to Amies charcoal transport medium and immediately sent at room temperature to the *Central* ~~*Reference*~~ Laboratory where the strains will be serotyped according to established procedures [Austrian, 1976].

Serotyping at the *Central* ~~*Reference*~~ Lab
For capsular typing of recovered pneumococci, the quellung reaction will be used according to established procedures [Austrian, 1976]. The absence of slide agglutination in the presence of a to f antisera (*Haemophilus influenzae* Agglutinating Sera MUREX ZM 20-25) will be used to identify non-encapsulated (non-typeable) *H. influenzae* strains.
For more details, please refer to Appendix H and the ~~*local lab SOPs and*~~ Lab Work Book.

5.5.2.3.5 Data flow

Investigators will receive relevant microbiological information from the local lab according to local routine practices. Results of the identification, ~~*and*~~ serotyping *and antibiotic sensitivity testing* of the blood culture and other isolates at the *Central*~~*Reference*~~ Lab will be transferred directly to the database of GSK. In order to preserve the blinding of the study, this information will only be made available to the study investigators after the end of the study.

5.6.1 Blood samples taken from the 'Immuno and reacto' *and 'Additional immuno'* subsets

A total of *3.5* ml of blood will be taken per subject at each of the ~~*four*~~ blood sampling visits *(in subjects of the 'Immuno and Reacto' or 'Additional immuno' subsets)* for further immunological analysis.

5.6.2 Nasopharyngeal swabs taken from the 'Carriage' subset

After taking the swabs, all samples should be transferred to *transport*~~*Skim milk-Tryptone-Glucose-Glycerine (STGG)*~~ medium and sent to the local bacteriology laboratory. *Refer to Investigator Lab manual Part II for details (Amended 26 November 2007)*. There, samples will be plated and incubated on blood and chocolate agar. If *S. pneumoniae* or *H. influenzae* colonies are identified, these colonies will be inoculated into ~~*20% skim milk medium or*~~ *a cryo-conservation medium* ~~*(i.e. Cryo-Bank)*~~ *(refer to COMPAS Lab Workbook)*and kept at -70°C. Hereafter, at least 3-5 days before the date of shipment to the *Central*~~*Reference*~~ Laboratory, the bacterial samples kept in ~~*20% skim milk*~~ *a cryo-conservation* medium *(refer to COMPAS Lab Workbook)* will be subcultured and incubated again on chocolate and blood agar. The most representative colonies will be selected and inoculated into Amies-charcoal transport medium and sent to *Central*~~*Reference*~~ Laboratory for confirmation.

5.7 Laboratory assays

As serum represents approximately 50% of whole blood, the approximate blood volumes drawn at Visit 4, Visit 6, Visit 7 and at Visit 9 *('Immuno and Reacto' subset)* will be *3.5* mL respectively in order to obtain 2 ml of serum.

1. *S. pneumoniae* opsonophagocytic activity will be measured by a killing-assay using a HL 60 cell line [Romero-Steiner et al, 1997; Henckaerts et al, 2007]. The results will be presented as the dilution of serum (opsonic titre) able to sustain 50 % killing of live pneumococci under the assay conditions. The cut-off of the assay is an opsonic dilution of 8.

5.7.1 Exploratory laboratory assays

The measure of anti-protein D (PD) antibodies by the anti-PD IgG ELISA has been showed inappropriate to discriminate healthy individuals to those who have developed AOM disease against NTHi. Different test developments have been initiated to develop a clinically pertinent assay; the most promising strategies currently followed to correlate serological response to protein D with protection are outlined below. It is worth noting that the list is not exhaustive and that other tests aiming to detect anti-PD protective antibodies might be developed. Provided technical qualification, these tests will be evaluated for their ability to differentiate protected from unprotected individuals.

5.7.1.1 Avidity anti-PD ELISA

Avidity of antibodies for their target antigen is recognized as a possible measure of their protective functionality. This functional test is based on the dissociation of low affinity antibody-antigen complexes in presence of a chaotropic agent such as sodium thiocyanate. The results are expressed as an avidity index (AI) which is the percentage of anti-PD antibodies remaining bound to PD after treatment with sodium thiocyanate.

5.7.1.2 Protein D enzymatic inactivation assay

In an infant rat infection model, it has been observed that wild-type NTHi strains induce ciliary lost in epithelial cells; this effect is not observed when a strain deleted for the gene encoding PD is used. The protein D is an enzyme with a glycerophosphoryl diester phosphodiesterase (GLPQ) activity producing choline from glycerophosphorylcholine. Choline is known to interact with PAF receptor (Platelet Activating Factor) and may trigger ciliary effacement through protein G transduction pathway. These observations indicate that the GLPQ enzymatic activity might be critical for bacterial colonization. Hence, antibodies with a specific GLPQ inhibitory effect could be good indicator of protective immunity. To detect such antibodies, a coupled enzymatic-optical test to assess the GLPQ activity of PD and its possible inhibition by human immune sera assay has been developed.

Briefly Glycerol-3-phosphate (G3P) produced from L-a-Glycerophosphorylcholine by the GLPQ activity is converted into dihydroxyacetone phosphate by a Glycerol-3-phosphate dehydrogenase (GPDH) added to the system. GPDH activity needs NAD+ as co-factor which is reduced into NADH during the enzymatic reaction. The rate of NADH production is followed by measuring the increase in absorbance at 340 nm. Presence of inhibitory antibodies in a given serum is thus assessed by a low level of NADH production. The test has been adapted to a microtiter plate format and the results are expressed as a percentage of inhibition.

5.7.1 Immunological read-outs

Table 12 Immunological read-outs

Number of subjects	Group	Blood sampling timepoint			Antigen[2]
		Timing	Months of age	Visit no.	
1,000 *subjects in 'Immuno and Reacto' subset*	All	Post III (M 5)<br>Post III (M13)<br>Post IV (M14)<br>Post IV (M22)	7<br>15-18<br>16-19<br>24-27	4<br>6<br>7<br>9	-Pneumococcal serotypes 1, 4, 5, 6B, 7F, 9V, 14, 18C, 19F, and 23F, and pneumococcal cross-reactive serotypes 6A and 19A (ELISA)<br>-Pneumococcal serotypes 1, 4, 5, 6B, 7F, 9V, 14, 18C, 19F, and 23F, and pneumococcal cross-reactive serotypes 6A and 19A (OPA)<br>-Protein D

6.2 Co-administered and concomitant vaccines

Table 15 Vaccines administered in the study

Vaccine	Group	Age of administration (months)	Type of product	Blinding
Study vaccines				
10Pn-PD-DiT	10Pn-PD-DiT	2, 4, 6 and 15-18	Randomized (Investigational product)	Blinded
Engerix-B	Control	2, 4 and 6	Randomized (no commercial lot)	Blinded
Havrix	Control	15-18	Randomized (no commercial lot)	Blinded
Co-administered vaccines				
Infanrix hexa	10Pn-PD-DiT	2, 4 and 6	Randomized (no commercial lot)	Blinded
DTPa-IPV/Hib	10Pn-PD-DiT<br>Control	15-18<br>2, 4, 6 and 15-18	Randomized (no commercial lot)	Blinded
Concomitant vaccines*				
Havrix	All subjects	12 and 18-21	Commercial lot	Unblinded
Neisvac-C	All subjects in Argentina	12	Commercial lot	Unblinded
Varicella	All subjects in *Colombia and* Panama	12	Commercial lot	Unblinded

**These vaccines are benefits from the study*

7.3.1 Documentation of any meningitis and/or invasive disease

Any case of meningitis*, regardless of the etiology,* should be reported *in the SAE Report Form* as a serious adverse event *~~using the SAE Report Form~~ and in the meningitis report form~~, including the appropriate meningitis section~~, and should be recorded in a specific eCRF page. In case of a confirmed meningitis, the corresponding pages in the Invasive Disease section, including the Lumbar Puncture section, should be filled in. In case of a probable meningitis, only the Lumbar Puncture section should be filled in.*

7.5 Time period, frequency, and method of detecting adverse events and serious adverse events

Study procedures for ALL SUBJECTS

For all subjects, even if not part of the subjects enrolled in Panama, AEs have to be documented in case*: 1)* this AE is~~:~~ a reason for withdrawal of the subject from the study or from study vaccination*, or 2) the AE refers to a convulsion episode occurring within 30 days after booster vaccination (Visit 6), and to be reported at Visit 8*. *Any case of convulsions occurring within 30 days after 10Pn-PD-DiT booster vaccination should be recorded in a specific eCRF page.* Otherwise, safety assessment in subjects who are not part of the 'Immuno and reacto' subset is limited to the SAE reporting.

The standard time period for collecting and recording SAEs will begin at the first receipt of study vaccine/ control*/ co-administered vaccine* and will continue during the entire study period (until 24 months of age). See Section 7.8 for instructions for reporting and recording SAEs. *In addition, in order to fulfil international reporting obligations, SAEs that are related to study participation (e.g. protocol-mandated procedures, invasive tests, a change from existing therapy) will be collected and recorded from the time the subject consents to participate in the study until she/he is discharged.*

9.2 Secondary endpoints

Reactogenicity (in the 'Immuno and reacto' subset of 1,000 subjects (500 *~~per country~~ subjects respectively in Argentina and Panama*)):

Immunogencity (in the 'Immuno and reacto' subset of 1,000 subjects (500 *~~per country~~ subjects respectively in Argentina and Panama*)):

9.3.1.1 Primary objective (B-CAP or C-AOM)

AOM endpoints will be assessed in all 7,000 subjects enrolled in Panama City (3,500 subjects per group). Considering that at any point in the study, we will have at least as many C-AOM episodes from the 7,000 subjects enrolled in Panama, as we have B-CAP from the total 24,000 subject cohort (Argentina, *Colombia* and Panama combined), the subjects enrolled in Panama will be large enough to also reach at least 1200 first C-AOM episodes at study end.

11 References

~~Arguedas A, Loiaza C, Perez A et al. Microbiology of acute otitis media in Costa Rican children. Ped Infect Dis J 1998; 17: 680-685.~~.

~~Camargo ME, et al. Immunoenzymatic assay of anti-diphtheric toxin antibodies in human serum. J Clin Microbiol 1984;20 (4): 772-774.~~.

~~Centers for Disease Control and Prevention and Emory University. Streptococcus pneumoniae opsonophagocytosis using differentiated HL-60 cells (Promyelocytic Leukemia Cell Line). Laboratory protocol. Version 1.1 1996.~~

~~Centers for Disease Control. Hepatitis B virus: a comprehensive strategy for eliminating transmission in the United States through universal childhood vaccination: recommendations of the Immunization Practices Advisory Committee (ACIP). MMWR 1991: 40(No.RR-13): 1-25.~~

~~Eskola J, Ward J, Dagan R, Goldblatt D, Zepp F, Siegrist CA. Combined vaccination of Haemophilus influenzae type b conjugate and diphtheria-tetanus-pertussis containing acellular pertussis. Lancet 1999; 354: 2063-8.~~

Friedman NR, McCormick DP, Pittman C, Chonmaitree T et al. Development of a practical tool for assessing the severity of Acute Otitis Media. Pediatr Infect Dis J 2006; 25(2):101-107.

García-Rodríguez JA and Fresnadillo Martínez MJ. Dynamics of nasopharyngeal colonization by potential respiratory pathogens. J of Antimicrobial Chemotherapy 2002; 50, Suppl S2, 59-73.

~~Granström M, et al. Acellular Pertussis Vaccine in Adults: adverse reactions and immune response. Eur J Clin Microbiol 1987;6(1): 18-21.~~

Henckaerts I, Durant N, De Grave D et al. Validation of a routine opsonophagocytosis assay to predict invasive pneumococcal disease efficacy of conjugate vaccine in children. Vaccine 200*~~6: in press~~ 7; 25(13): 2518-2527.*

~~*Karpinsky KF, et al. Statistical considerations in the quantitation of serum immunoglobulin levels using the enzyme-linked immunosorbent assay (ELISA). J Immunol Methods 1987;103: 189-194.*~~

~~*Madhi S, Klugman K. A role for Streptococcus pneumoniae in virus-associated pneumonia. Nature Medicine 2004; 10(8): 811-13.*~~.

Madhi S.A. World Health Organisation definition of "radiologically-confirmed pneumonia" may underestimate the true public health value of conjugate pneumonia vaccines. Vaccine 200~~*6: in press*~~ *7: 25(13): 2413-2419*.

~~*Melville-Smith ME, et al. A comparison of enzyme-linked immunosorbent assay (ELISA) with the toxin neutralization test in mice as a method for the estimation of tetanus antitoxin in human sera. J. Biol. Standard 1983;11: 137-144.*~~.

~~*Sato Y, et al. Role of antibody to filamentous hemagglutinin and to leukocytosis promoting factor-hemagglutinin in immunity to pertussis. In: Robbins JB, Hill JC, Sardoff JC ed. Seminars in Infectious Disease: Bacterial Vaccines. New-York: Thierne-Strattton, Inc. 1982;380-385.*~~

~~*Varon E, Gutman L. Rapport d'activité 2003. Epidémiologie 2002. Centre National de Référnce de Pneumocoques. Institut National de Veille Sanitaire. Available at www.invs.sante.fr 2003.*~~.

~~*WHO Collaborative Study Group on Oral and Inactivated Poliovirus Vaccines. Combined immunization of infants with oral and inactivated poliovirus vaccines: results of a randomized trial in the Gambia, Oman and Thailand. Bulletin of the World Health Organization, 1996, 74 (3): 253-268.*~~

~~*WHO. Progress in the control of viral hepatitis: memorandum for a WHO meeting. Bull WHO 1988; 66:443-445.*~~.

Christ-Crain M, Jaccard-Stolz D et al. Effect of procalcitonin-guided treatment on antibiotic use and outcome in lower respiratory tract infections: Cluster-randomized, single-blinded intervention trial. Lancet 2004, 363: 600-607.

Simon L, Gauvin F, Amre DK et al. Serum procalcitonin and C-reactive protein levels as markers of bacterial infection: A systematic review and meta-analysis. Clinical Infect Dis 2004, 39: 206-217.

CHMP. Note for guidance on clinical evaluation of new vaccines. European Medicine Evaluation Agency, May 2005. 23 p.

Appendix E Vaccine supplies, packaging and accountability ~~*(with CSC)*~~

In Argentina*,* *Colombia* and Panama, *Havrix* will be used for vaccination of subjects against hepatitis A at the age of 12-15 months (visit 5) and at 18-21 months of age.

In *Colombia and* Panama, Varilrix will be administered to all subjects at 12 months of age, supplied in monodose vials and container of diluent.

Appendix F Definitions of radiographic outcomes and guidelines for radiographic techniques (WHO, 2003)

A) Definitions of radiographic outcomes (WHO, 2001)

The reading of the image will be classified as:

- ***Consolidated CAP: a dense opacity that may be a fluffy consolidation of a portion of whole or of a lobe or of the entire lung, often containing air bronchograms and sometimes associated with pleural effusion. Pleural effusion refers to the presence of fluid in the pleural space between the lung and the chest wall. In most cases this will be seen at the costo-phrenic angle or as a layer of fluid adjacent to the lateral chest wall. This does not include liquid seen in the horizontal or oblique fissures. Pleural effusion is considered as primary endpoint if it is in the lateral pleural space (and not just in the minor or oblique fissure) and is spatially associated with a pulmonary parenchymal infiltrate (including other infilatrate), OR if the effusion obliterates enough of the hemithorax to obscure an opacity.***
- ***Non-consolidated CAP: linear and patchy densities (interstitial infiltrate) in a lacy pattern involving both lungs, featuring peribronchal thickening and multiple areas of atelectasis. Lung inflation is normal to increased. It also includes minor patchy infiltrates that are not of suffiecient magnitude to constitute primary end-point consolidation, and small areas of atelectasis which in children can be difficult to distinguish from consolidation.***
- ***Normal CXR: absence of consolidation, pleural effusion and/or abnormal infiltrates on the digital CXR image.***
- ***Uninterpretable: an image is classified as "uninterpretable" if the features of the image are not interpretable in terms of presence or absence of "primary end-point" without additional images. No further reading should be made for other infiltrates on such images.***

B) Guidelines for radiographic techniques (WHO, 2003).

Appendix H Procedures for the detection of *Streptococcus pneumoniae* and *Haemophilus influenzae* in each country and at the Central~~*Reference*~~ Laboratory ~~*(Instituto Malbrán, Argentina)*~~

(2) Pneumococci and *H. influenzae* strains transport to the *Central*~~*Reference*~~ Laboratory *Eurofins Medinet Inc.* ~~*(Instituto Malbrán)*~~*.*

All the strains identified as *S. pneumoniae* and *H. influenzae* must be sent to the *Central Laboratory Eurofins Medinet Inc. in USA*~~*Instituto Malbrán in Argentina*~~*.* For this purpose use the supplied transport media ~~*(Amies- charcoal)*~~*.*

(3) *Eurofins Medinet Inc. Central*~~*Reference*~~ Laboratory ~~*(Instituto Malbrán)*~~: laboratory activities.

***Target pathogens*~~*Positive hemocultures*~~ will be sent to:**

Eurofins Medinet, Inc.

13665 Dulles Technology Drive, #100R

Herndon, VA 20171

Tel: +1 703.480.2500

Fax: +1 703.480.2655

www.eurofinsmedinet.com

~~***Instituto Malbrán***~~

~~***Administración Nacional de Laboratorios e Institutos de Salud***~~

~~***"Dr. Carlos G. Malbrán"***~~

~~***Av. Vélez Sarsfield 563***~~

~~***(1281) Buenos Aires - Argentina***~~

~~***www.anlis.gov.ar***~~

<u>Streptococcus pneumoniae</u>

1. *S. pneumoniae* confirmation

Hemolytic pattern on blood agar (alpha-hemolytic)

d. **Optochin test: This test will differentiate *S. pneumoniae* from other alpha haemolytic streptococci. *This test is used to determine the effect of optochin (ethylhydrocupreine hydrochloride) on a microorganism. Optochin lyses S. pneumoniae (positive test) but alpha streptococci are resistant (negative test). Pneumococci may, therefore, be differentiated from other alpha hemolytic streptococci by the formation of a zone of inhibition around a Taxo P disk (disks impregnated with approximately 5.0 ug of hydrocupreine hydrochloride [optochin]). The disk is placed on a blood agar plate heavily inoculated with a pure culture of S. pneumoniae, and optochin susceptibility is assessed after incubation overnight. ~~It is based on the fact that the growth of S. pneumoniae is inhibited by 5.0 ug of optochin (ethylhydroxycuprine) contained in a paper disk applied to the surface of a blood agar plate.~~.***

e. **Bile solubility test: *The bile solubility test differentiates S. pneumoniae (bile soluble) from other alpha-haemolytic streptococci (bile insoluble).* This test determines the ability of an organism to be lysed in the presence of bile salts (sodium deoxycholate). *~~It is used specifically to differentiate S. pneumoniae (bile soluble) from other alphahemolytic streptococci (bile insoluble).~~* Bile salts decrease surface tension at the medium-membrane interface and also cause derangement of the cell membrane. However, *S. pneumoniae* produces an intracellular autolytic enzyme causing a rapid autolysis of the micro-organism when growing on artificial media. The role of bile salts is to accelerate this natural autolytic process by combining with the pneumococcal cell and activating its autolysin. *This test will be performed by spot testing the colony with sodium-deoxycholate and assessing its solubility (lysis).***

~~c. Latex agglutination test: Slidex pneumo-kit (bioMérieux Nº58821), latex agglutination test that recognises capsular antigens from S. pneumoniae (83 serotypes).~~.

2. *~~Penicillin and cefotaxime s~~*Susceptibility testing through the broth micro dilution test.

The Minimal Inhibitory Concentration (MIC) is a quantitative measurement of in vitro activity of an antimicrobial agent against a given bacterial isolate. The standard test is performed by exposing the organism to doubling dilutions of antibiotic. The MIC is the lowest antibiotic concentration that inhibits completely the growth of the organism as determined with the naked eye. *~~The numeric value is interpreted as susceptible, intermediate or resistant based on tables provided in~~*

~~the NCCLS 2005 for penicillin and cefotaxime~~.

Streptococcus pneumoniae							CMP6ADAI				
Levoflox<br>0.06	Levoflox<br>0.12	Levoflox<br>0.25	Levoflox<br>0.5	Levoflox<br>1	Levoflox<br>2	Levoflox<br>4	Levoflox<br>8	Cipro<br>1	Cipro<br>2	Cipro<br>4	Penicillin<br>0.03
Penicillin<br>0.06	Penicillin<br>0.12	Penicillin<br>0.25	Penicillin<br>0.5	Penicillin<br>1	Penicillin<br>2	Penicillin<br>4	Penicillin<br>8	Amox/clav<br>0.015/0.08	Amox/clav<br>0.03/0.015	Amox/clav<br>0.06/0.03	Amox/clav<br>0.12/0.06
Amox/clav<br>0.25/0.12	Amox/clav<br>0.5/0.25	Amox/clav<br>1/0.5	Amox/clav<br>2/1	Amox/clav<br>4/2	Ceftriax<br>0.015	Ceftriax<br>0.03	Ceftriax<br>0.06	Ceftriax<br>0.12	Ceftriax<br>0.25	Ceftriax<br>0.5	Ceftriax<br>1
Ceftriax<br>2	Ceftriax<br>4	Cefurox ax<br>0.12	Cefurox ax<br>0.25	Cefurox ax<br>0.5	Cefurox ax<br>1	Cefurox ax<br>2	Cefurox ax<br>4	Clarithrom<br>0.015	Clarithrom<br>0.03	Clarithrom<br>0.06	Clarithrom<br>0.12
Clarithrom<br>0.25	Clarithrom<br>0.5	Clarithrom<br>1	Azithrom<br>0.015	Azithrom<br>0.03	Azithrom<br>0.06	Azithrom<br>0.12	Azithrom<br>0.25	Azithrom<br>0.5	Azithrom<br>1	Azithrom<br>2	Azithrom<br>4
Erythrom<br>0.015	Erythrom<br>0.03	Erythrom<br>0.06	Erythrom<br>0.12	Erythrom<br>0.25	Erythrom<br>0.5	Erythrom<br>1	Erythrom<br>2	Erythrom<br>4	Clindamy<br>0.015	Clindamy<br>0.03	Clindamy<br>0.06
Clindamy<br>0.12	Clindamy<br>0.25	Clindamy<br>0.5	Clindamy<br>1	Vanco<br>0.06	Vanco<br>0.12	Vanco<br>0.25	Vanco<br>0.5	Vanco<br>1	SXT<br>0.06/1.2	SXT<br>0.12/2.4	SXT<br>0.25/4.75
SXT<br>0.5/9.5	SXT<br>1/19	SXT<br>2/38	SXT<br>4/76	Tetracyc<br>0.06	Tetracyc<br>0.12	Tetracyc<br>0.25	Tetracyc<br>0.5	Tetracyc<br>1	Tetracyc<br>2	Tetracyc<br>4	Pos<br>Control

Antimicrobial Agent	MIC Range	# of wells
Levofloxacin	0.06-8	8
Ciprofloxacin	1-4`	3
Penicillin	0.03-8	9
Amoxicillin/clavulanate	0.015/0.008-4/2`	9
Ceftriaxone (non-men)	0.015-4	9
Cefuroxime axetil	0.12-4	6
Clarithromycin	0.015-1	7

Antimicrobial Agent	MIC Range	# of wells
Azithromycin	0.015-4	9
Erythromycin	0.015-4	9
Clindamycin	0.015-1	7
Vancomycin	0.06-1	5
Trimeth/Sulfa (SXT)	0.06/1.2-4/76`	7
Tetracycline	0.06-4	7
Pos Control		1

3. *S. pneumoniae* capsular serotyping.

S. pneumoniae capsular serotyping will be subcontracted to University Medical Center Amsterdam, National Pneumococcal Reference Laboratory. S. pneumoniae **capsular serotyping is performed according to the Danish Nomenclature System and it is done by Neufeld-Quellung reaction, using 12 pooled antisera and selected factor antisera (Statens Serum Institut of Denmark).The type or group is established from the reaction pattern. This system allows the identification of the most common 21 serotypes or serogroups worldwide.**

***~~If the serotyping is not complete (group determination only), the use of selected factor antisera will be necessary~~* .**

4. *S. pneumoniae* strains storage

Each strain of *S. pneumoniae* completely processed as described in this protocol will be stored by freezing at –70ºC (*Brucella broth with 20% glycerol*~~*tripticase soy broth/ glycerol*~~) at the *Central*~~*Reference*~~ Laboratory *Eurofins Medinet Inc.* ~~*(Instituto Malbrán)*~~*.*

<u>Haemophilus influenzae</u>

5. *H. influenzae* **confirmation**~~***: factor X and Y growth requirements***~~

~~***Growth in supplemented chocolate agar***~~

~~***Gram stain: morphology***~~	~~***Pleiomorphic, coccus bacillus***~~
~~***stain***~~	~~***Negative***~~
~~***Oxidase test***~~	~~***Positive***~~
~~***Catalase test***~~	~~***Negative***~~
~~***Porphyrins test***~~	~~***Negative***~~
~~***Serotyping***~~	~~***a-f polivalent antisera***~~

f. ***Morphology***

g. ***Growth on Chocolate agar and no growth on blood agar***

h. ***Quad Plate Method: The Haemophilus (Hemo) Identification (ID) Quad plate (with growth factors) is used for the differentiation and presumptive identification of Haemophilus species based on requirements of growth factor X and or V and hemolytic reactions. H. influenzae requires both X factor (component of hemin) and V factor (NAD) for growth. A four quadrant plate that contains Brain Heart Infusion Agar in quadrants I, II, and III and Blood Agar Base in quadrant IV will be used. Quadrant I: Enriched with hemin to supply X factor. Quadrant II: Enriched with Isovitalex Enrichment to supply V factor and other nutrients, such as thiamine and cysteine, which stimulate the growth of Haemophilus species. Quadrant III: Contains both hemin (X factor) and Isovitalex enrichment (V factor). Quadrant IV: Supplemented with NAD to supply V factor; horse blood to provide X factor and demonstrate hemolytic reactions. If the isolate is H. influenzae, it should grow in quadrants III and IV***

6. *H. influenzae*: capsular serotyping

H. influenzae serotyping will be performed by Qualitative slide Agglutination test (FDA approved REMEL kit). Capsule production has been shown to be an important virulence factor for H. influenzae, and encapsulated strains are classified serologically into six types according to the chemical structure of the capsular antigen. Strains possessing these antigens are specifically agglutinated by the homologous antiserum, and a culture of an encapsulated strain may therefore be typed by slide agglutination tests. Haemophilus influenzae Agglutinating Sera are intended for use in the qualitative slide agglutination test to identify serologically the type antigen of pathogenic strains of H. influenzae (types a to f) for epidemiological and diagnostic purposes. ~~***The capsule of several bacteria species is a factor of virulence and allows serotyping, therefore, if the capsule is not available, serotyping cannot be done. The capsulated Haemophilus strains can be identified by serotyping, based on the chemical composition of the capsule polysaccharide. It is important to remind that the capsule is lost in the subcultures and the structure of the capsule is damaged in old cultures. Therefore, the serotyping needs to be done in cultures within less than 24 hours and as soon as possible after the primary culture.***~~

~~***The serotyping can be performed by laminar agglutination, capsule swelling (Quellung reaction), co-agglutination, immunofluorescence, or contra-immunoelectrophoresis.***~~

~~***The laminar agglutination is the most common method for serotyping in reference laboratories. The microorganisms in suspension are agglutinated when they are mixed with antibodies against the surface components. This technique is a simple and fast method for identifying and serotyping H. influenzae.***~~

~~***Refer to Guidelines from Colombia, based on SIREVA II guidelines***~~

7. Determination of beta-lactamase production

Beta-lactamase production by H. influenzae will be assessed using a DrySlide Nitrocefin test. In brief, colonies of a H. influenzae isolate will be smeared onto a slide containing the nitrocefin reagent. Nitrocefin as a substrate contains a beta-lactam ring, which when hydrolyzed (in the presence of beta-lactamases) produces cephalosporanic acid causing a colorimetric change. A change from yellow-pink indicates the production of beta-lactamase.

8. Susceptibility testing through the broth micro dilution test

Haemophilus influenzae CMP2ADAI

Levoflox 0.004	Levoflox 0.008	Levoflox 0.015	Levoflox 0.03	Levoflox 0.06	Levoflox 0.12	Levoflox 0.25	Levoflox 0.05	Levoflox 1	Levoflox 2	Levoflox 4	Levoflox 8
Ampicillin 0.12	Ampicillin 0.25	Ampicillin 0.5	Ampicillin 1	Ampicillin 2	Ampicillin 4	Ampicillin 8	Amox/cl 0.015/0.008	Amox/cl 0.03/0.015	Amox/cl 0.06/0.03	Amox/cl 0.12/0.06	Amox/cl 0.25/0.12
Amox/cl 0.5/0.25	Amox/cl 1/0.5	Amox/cl 2/1	Amox/cl 4/2	Amox/cl 8/4	Amox/cl 16/8	Ceftriax 0.015	Ceftriax 0.03	Ceftriax 0.06	Ceftriax 0.12	Ceftriax 0.25	Ceftriax 0.5
Ceftriax 1	Ceftriax 2	Ceftriax 4	Ceftriax 8	Ceftriax 16	Cefurox ax 0.06	Cefurox ax 0.12	Cefurox ax 0.25	Cefurox ax 0.5	Cefurox ax 1	Cefurox ax 2	Cefurox ax 4
Cefurox ax 8	Cefurox ax 16	Clarithrom 0.015	Clarithrom 0.03	Clarithrom 0.06	Clarithrom 0.12	Clarithrom 0.25	Clarithrom 0.5	Clarithrom 1	Clarithrom 2	Clarithrom 4	Clarithrom 8
Clarithrom 16	Clarithrom 32	Azithrom 0.015	Azithrom 0.03	Azithrom 0.06	Azithrom 0.12	Azithrom 0.25	Azithrom 0.5	Azithrom 1	Azithrom 2	Azithrom 4	Azithrom 8
Azithrom 16	Azithrom 32	Erythrom 0.015	Erythrom 0.03	Erythrom 0.06	Erythrom 0.12	Erythrom 0.25	Erythrom 0.5	Erythrom 1	Erythrom 2	Erythrom 4	Erythrom 8
Erythrom 16	Erythrom 32	SXT 0.015	SXT 0.03	SXT 0.06	SXT 0.12	SXT 0.25	SXT 0.5	SXT 1	SXT 2	SXT 4	Pos Control

Antimicrobial Agent	MIC Range (mg/mL)	# of wells
Levofloxacin	0.004-8	12
Ampicillin	0.12-8	7
Amoxicillin/clavulanate	0.015/0.008	11
Ceftraixone	0.015-16	11
Cefuroxime axetil	0.06-16	9

Antimicrobial Agent	MIC Range (mg/mL)	# of wells
Clarithromycin	0.015-32	12
Azithromycin	0.015-32	12
Erythromycin	0.015-32	12
Trimeth/Sulfa (SXT)	0.015/0.3-4/76	9
Pos Control		1

9. H. influenzae strains storage

Each strain of H. influenzae completely processed as described in this protocol will be stored frozen at -70°C (Brucella broth with 20% glycerol) at the Central Laboratory Eurofins Medinet Inc.

Appendix I AOM severity scales used in COMPAS

Acute Otitis Media (AOM) is a common problem that creates a burden on parents and children in any health care system. Nearly all children experience at least 1 episode of AOM by the time they are 3 years old (Teele et al, 1989). AOM, even if treated correctly, may have a recurrent or persistent nature. Around 10 – 20 % of children will experience subsequent episodes or have persistent mild infections that last up to 12 weeks (Teele et al, 1989).

In many countries, antibiotics are used initially for treatment of AOM. However, the appropriateness of antibiotic prescription at the initial visit remains controversial. Some clinicians favour withholding antibiotics in part because some fraction of

AOM is caused by virus and in part because they are concerned that unnecessary antibiotics encourage the emergence of mutidrug-resistant bacterial stains.

The emergence of a 10 valent pneumococcal vaccine that could also induce protection against non-encapsulated Haemophilus influenzae may be one of the most important new developments on immunization in the coming decade. For this reason, the COMPAS study has as a primary objective to demonstrate vaccine efficacy against clinically confirmed AOM caused by vaccine serotype pneumococci and H. influenzae.

In order to better describe and understand the impact of 10Pn-PD-DiT vaccination on the AOM burden of disease, the severity of each AOM case will be measured and reported during the study. Unfortunately, there are no universally validated scales in the literature to measure AOM severity in vaccine clinical trials performed in infants. Previous experiences shown excellent results on AOM severity measurement by combining subjectives and objective parameters: parent perception of severity and otoscopy findings (Dagan XXX, Friedman et al, 2006):

1. Dagan Scale

- ***Based on objective and subjective parameters***

Scoring varies between Mild (0-4 points), Moderate (5-7 points) and Severe (8-15 points)

Acute otitis media clinical/otologic score based on temperature, irritability and ear tugging on the day of the visit as reported by parents and redness and bulging of the tympanic membrane at the otologic examination

Score	Temperature (°C)	Irritability	Tugging	Redness	Bulging
0	<38.0	Absent	Absent	Absent	Absent
1	38.0-38.5	Mild	Mild	Mild	Mild
2	38.6-39.0	Moderate	Moderate	Moderate	Moderate
3	>39.0	Severe	Severe	Severe	Severe*

*Including drainage pus

2. Friedman Scale (also known as McCormick) [Friedman et al, 200])

- ***Very complex, combines 4 scales (ETG-5, OM-3, FACES and OS-8/Tympanic Pictures***
  - ***ETG-5 is based on 4 subjective parameters (ear pain, irritability, feeding and sleeping) and 1 objective parameter (fever).***

	Score = 0	Score = 4	Score = 7
Fever	< 100.4 °F	100.4 – 102,2	> 102.2
Ear ache	Tugging	Occasional	Frequent
Irritability	None	Occasional	Frequent
Feeding	Feeds well	Mild decrease in appetite	Very poor appetite
Sleeping	Normal	Some what restless sleep	Very poor sleep

  - ***OM-3 consists of a parent oriented questionnaire to indirectly assess the subject´s health. Parent involvement and feedback is required, leading to the generation of subjective data.***
  - ***FACES is not useful in clinical trials where toddlers or very young children are involved, since the scale is based on the child's disease perception.***
  - ***Which face describes your child's symptoms during the past 24 hours?***

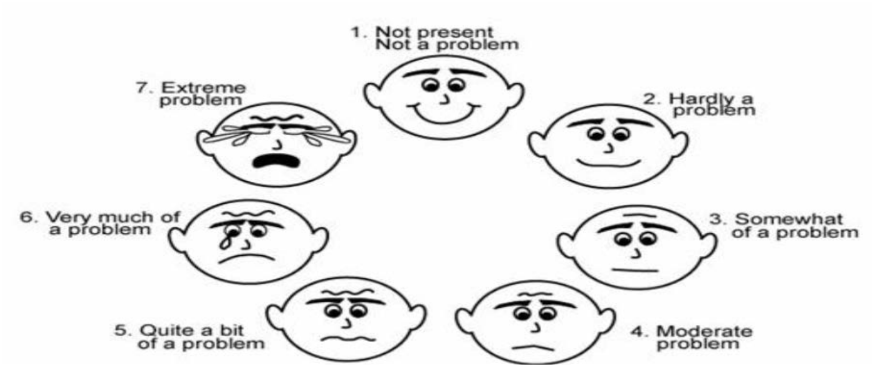

© David P.- McCormick M.D., 2006

– ***OS-8/Tympanic pictograms: This is an objective scale, which allows the ENT specialist to grade the disease, based on 8 different pictograms, according to the image seen at the moment of visualising the tympanic membrane. Very easy to use.***

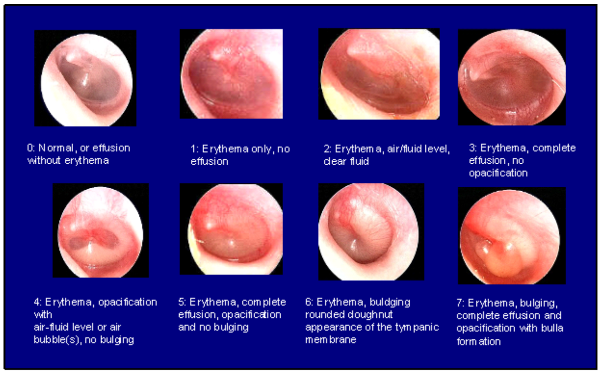

No standardized methods exist to measure AOM severity. There are however scales that have been used in previous clinical trials combining subjective and objective AOM parameters to perform assessment on AOM cases. The association of the Dagan and McCormick scales demonstrated excellent performance with easy implementation. However, OM-3 and FACES included in McCormick scale are found not to be relevant for the COMPAS study, since involvement of parents on the assessment of each case is too subjective and subjects are too young to express their discomfort.

Therefore, AOM severity assessment will combine "objective elements", as described in the Friedman scale (using only ETG-5 and OS-8 scales), with subjective elements required by the Dagan scale. Collection of clinical data will be performed by the pediatrician /general physician who refers the case; pictograms assessment and other objective elements related to the direct observation of the ear drum will be performed by the ENT specialist.

GlaxoSmithKline Biologicals

Clinical Research & Development
Protocol Amendment Approval

**eTrack study number**	109563
**eTrack abbreviated title**	10PN-PD-DIT-028
**Protocol title:**	Clinical Otitis Media and Pneumonia Study (**COMPAS**): a phase III , double-blind, randomized, controlled, multicentre study to demonstrate the efficacy of GlaxoSmithKline (GSK) Biologicals' 10-valent pneumococcal conjugate vaccine (GSK1024850A) against Community Acquired Pneumonia (CAP) and Acute Otitis Media (AOM)
**Amendment number:**	Amendment 2
**Amendment date:**	20 March 2008
**Co-ordinating author:**	Mireille Venken, Scientific Writer

Rationale/background for changes: Amendment 2 of the COMPAS protocol was developed for the following reasons:

- In Colombia, two doses of HRV *(Rotarix)* vaccine will be offered to all subjects within the first six months of life to provide additional benefit.
- In Panama, subjects that are part of the 'Carriage' subset will also have the opportunity to participate in the 'Additional immuno' subset. However, subjects that are part of the 'Immuno and reacto' subset will not be invited to participate in the 'Additional immuno' subset as already four blood samples will be collected from these subjects.
- For pre-term born infants the gestation period has been defined
- In case subjects would by mistake receive a vaccine with antigens common to the antigens contained in the study or co-administered vaccines outside of the context of the study, the investigator would need to evaluate whether the subject could still continue participation in the study.Therefore this criterion was added to the exclusion criteria for further study participation.
- For subjects in the 'Additional Immuno' subset visit 4 was removed from the list of study procedures and the detailed description of study procedures.
- New validated temperature monitoring devices have been provided to the investigational sites. Although the temperature monitoring process described in section 6.4 still covers the use of these new devices, this section was updated with the mandatory text of the current protocol document standard (version 12.5) to avoid confusion.
- The classification of the CXR has been aligned throughout the protocol and with the X-ray workbook.
- Additional minor changes and clarifications have been implemented.

**Amended text has been included in *bold italics* in the following section(s):**	
**Title Page:**	
**Co-ordinating author**	***Mireille Venken, Scientific Writer***<br>Thomas Moens PhD, Scientific Writer
**Contributing authors**	• Juan Pablo Yarzabal MD, MSc, Clinical Development Manager<br>• Leyla Hernandez, ***Raquel Merino*** Central Study Co-ordinator***s***<br>• Isabelle Henckaerts, Scientist, Clinical Immunology<br>• Lode Schuerman MD, Director Clinical Development (Pneumococcal Vaccines)<br>• Patricia Lommel, Biostatistician<br>• William Hausdorff PhD, Director Epidemiology<br>• Ricardo Ruttiman MD, Clinical Director R&D and Medical Affairs Argentina and Southern Cone
**Synopsis:**	
**Study design**	• ***In a***~~A~~ll groups DTPa-IPV/Hib (*Infanrix* IPV/Hib) or DTPa-HBV-IPV/Hib (*Infanrix hexa*) ***will be co-administered according*** to the following immunization scheme:<br>• All subjects will participate in ***at least*** 7 scheduled visits, and a study conclusion contact.<br>• Three subsets of subjects will be defined:<br>– ‘Immuno and reacto’ subset: 500 subjects in Panama and Argentina, totaling 1,000,<br>– ‘Carriage’ subset: a subset of 2,000 subjects of all subjects enrolled in Panama<br>– ‘Additional immuno’ subset: a subset of ***approximately*** 3,500 subjects (not participating in the ***‘Immuno and reacto’*** subset) of all subjects enrolled in Panama<br>• The target recruitment time will be ***approximately*** 12 months ***(depending on the time needed to complete target enrolment),*** starting from first subject enrolled in the study
**List of Abbreviations**	
Rotarix™	***GSK Biologicals’ oral live attenuated HRV vaccine, referred throughout the document as Rotarix***
**Glossary of terms:**	
**Concomitant vaccines**	MMR, Varilrix™, ***Rotarix™*** and Neisvac®-C will be referred to as concomitant vaccines in this study protocol.

Section 3.1 Study design:
Figure 2 General study design

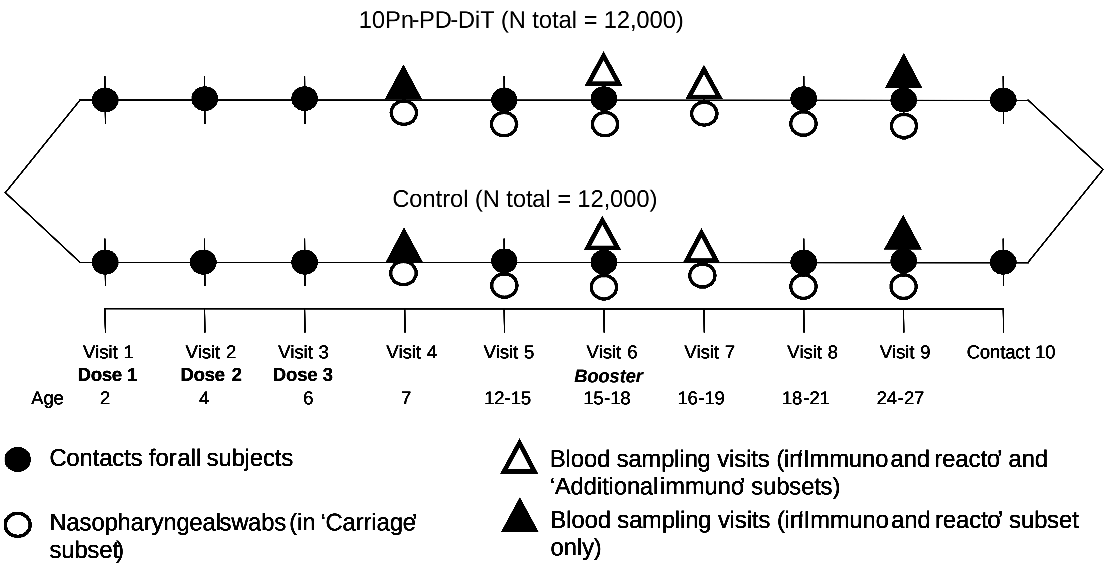

- ***In Colombia, 2 doses of licensed Rotarix vaccine will be offered to all subjects (also subjects in the 10Pn-PD-DiT vaccine group) within the first six months of life.***
- Target recruitment time will be ***approximately*** 12 months ***(depending on the time needed to complete target enrolment),*** starting from first subject enrolled.
- ***Risk factors for pneumococcal and/or H.influenzae infections (agammaglobulinemia, HIV infection, sickle cell disease, nephrotic syndrome, chronic renal failure, organ transplantation, diabetes mellitus, congestive heart failure and CSF leaks), breastsfeeding history and composition of the household will be checked at visits 1, 5 and 9.***
- Additional immuno' subset: a subset of approximately 3,500 children (not participating in the ***'Immuno and reacto'*** subset) in Panama will have two blood samples taken: just before the booster injection (Visit 6 at 15-18 months of age) and one month post booster (Visit 7 at 16-19 months of age), to be stored and used after study end for exploring the correlation of protein D related responses with occurrence of AOM caused by non-typable Haemophilus influenzae (NTHi).

Section 3.2 Detailed vaccination scheme:
In Colombia, 2 doses of licensed Rotarix vaccine will be offered to all subjects (also subjects in the 10Pn-PD-DiT vaccine group) within the first six months of life.

Section 4.2 Inclusion criteria for enrolment:
- A male or female between, and including, 6 and 16 weeks of age (between 42 and 118 days) at the time of the first vaccination. Pre-term born infants ***(born after a gestation period of less than 37 weeks)*** can be included in the study starting from 8 weeks of chronological age at the time of first vaccination and up to 16 weeks of chronological age (between 56 and 118 days).

Section 4.3 Exclusion criteria for enrolment:

- Previous vaccination against diphtheria, tetanus, pertussis, *Haemophilus influenzae* type b, hepatitis A and/or *S. pneumoniae*. Locally recommended EPI vaccines to be given at birth (such as BCG, Hepatitis B, or OPV) are allowed, but should be administered at least one month before the first dose of the study vaccine.Other locally recommended vaccines (such as influenza or rotavirus vaccines, recommended either through the EPI program or through national immunization campaigns) are always allowed, even if ***concomitantly administered*** ~~co-administered~~ with the study vaccines, but should be documented in the eCRF.

Section 4.4 Exclusion criteria for further study participation

The following criteri~~on~~***a*** should be checked for all subjects at each scheduled and unscheduled visit subsequent to the first visit

- Diagnosis of high risk disease for pneumococcal infections (such as HIV positive, sickle cell disease and splenectomized infants) will be evaluated according to medical judgement. Decision on withdrawal of the subject from the study will depend on the medical condition of the subject, the existence of a specific local vaccination program, and the availability of the registered pneumococcal vaccine. If Prevnar is made available through a targeted immunization program for these high risk groups, further participation in the COMPAS study would no longer be possible, and ~~HIV positive~~ ***these*** subjects will need to be excluded from the study and referred to the specific Prevnar immunization program. Once excluded, subjects can not return to receive subsequent study vaccine doses, even if Prevnar would no longer be available.
- ***For each case, where by mistake, vaccines with antigens common to the antigens contained in the study or co-administered vaccines are administered outside of the context of the study, the investigator will need to evaluate whether the subject can still continue participation in the study.***

Section 4.6 Warnings and precautions:

Human rotavirus vaccine (Rotarix)

Rotarix is a vaccine that is administered orally and should under no circumstances be be injected. The administration of Rotarix should be postponed in subjects suffering from diarrhoea or vomiting.

From the side effects commonly reported after administration of Rotarix the following were not listed above: flatulence, abdominal pain, regurgitation of food and fatigue. Uncommonly constipation was reported. Side effects very rarely reported, for Rotarix include: upper respiratory tract infection, hoarseness, rhinorrhoea, dermatitis, rash, muscle cramp and gastroenteritis.

Contacts of recent Rotarix vaccinees should be advised to observe personal hygiene (e.g. wash their hands after changing child's nappies). In a large safety trial in which 63,225 subjects were vaccinated with either Rotarix or with placebo, there was no evidence of an increased risk of intussusception (part of the gut pulls inward like a telescope) in the Rotarix group as compared with the placebo group.

Section 5.4.1 Scheduled visits and study procedures for all subjects:
All study subjects, participating in the study, will have ***at least*** seven scheduled visits and a final study conclusion contact (see Table 4).

Table 4 List of study procedures applicable for all subjects

Study visit<br><br>Age of subject<br><br>Study timing	VISIT 1<br><br>6-16 weeks<br>Month 0	VISIT 2<br><br>± 4 months<br>Month 2	VISIT 3<br><br>± 6 months<br>Month 4	VISIT 4[1]<br><br>± 7 months<br>Month 5	VISIT 5<br><br>12-15 months<br>Month 10-13	VISIT 6<br><br>15-18 months<br>Month 13-16	VISIT 7[1]<br><br>16-19 months<br>Month 14-17	VISIT 8<br><br>18-21 months<br>Month 16-19	VISIT 9<br><br>24-27 months<br>Month 22-25	CONTACT 10<br><br><br>Study end
Informed consent	●									
Check inclusion criteria	●									
Check exclusion criteria	●									
Demography, including gestational age	●									
Medical history	●									
Pre-vaccination history	●									
Check elimination criteria		●	●		●	●		●	●	
Check risk factors	●				●				●	
Check warnings, precautions and contraindications	O	O	O		O	O		O		
Physical examination	●	●	●		●	●		●	●	
Weight/height recording	●	●	●			●			●	
Pre-vaccination body temperature	●	●	●			●				
Randomization	●									
Vaccination (study and co-administered vaccines)	●	●	●			●				
Vaccination (concomitant vaccines)[2]					●			●		
Record any concomitant vaccination	●	●	●		●	●		●	●	
Reporting of unsolicited Adverse Events[3]		●	●		●	●		●	●	
Reporting of convulsions[4]								●		
Reporting of Serious Adverse Events		●	●		●	●		●	●	●
Study Conclusion										●

Note:
● is used to indicate a study procedure that requires documentation in the individual CRF (eCRF).
O is used to indicate a study procedure that does not require documentation in the individual eCRF.
[1]Study activities during grey-coloured visits are only applicable for subjects in the 'Immuno and reacto' and/or the 'Carriage' subsets (see Table 5 and 7, respectively)
[2]Concomitant vaccines: in addition to the blinded study vaccines, subjects will be vaccinated with *(1)* MMR at the age of 12-15 months (according to local EPI), and *(2)* with HAV vaccine at the age of 12-15 months (visit 5) and at 18-21 months of age (since subjects in the control group receive a dose of HAV vaccine at visit 6, the last HAV dose should be administered at least 28 days after the stuy vaccine booster dose) (study benefit= vaccination against HAV at 18-21 months of age in Argentina and at 12-15 and 18-21 months of age in Panama and Colombia). *(3)* In Argentina all subjects will be vaccinated at 12-15 months of age against *Neisseria meningitidis* group C and in Colombia and Panama all subjects will be vaccinated against varicella at 12-15 months of age (study benefit= vaccination against Neisseria in Argentina and against varicella in Colombia and Panama). ***(4) In Colombia all subjects will be vaccinated against HRV at visit 2 and visit 3 (study benefit= vaccination against HRV)***
[3]Only applicable for subjects enrolled in Panama
[4]Applicable for subjects enrolled in Argentina, Colombia and Panama

Section 5.4.2 Scheduled visits and study procedures for 'Immuno and reacto' subset:

Table 6 List of study procedures for subjects participating in the 'Immuno and reacto' subset of the study:

Study visit<br><br>Age of subject<br><br>Study timing<br><br>Blood sampling timepoints	VISIT 1<br><br>6-16 weeks<br>Month 0	VISIT 2<br><br>± 4 months<br>Month 2	VISIT 3<br><br>± 6 months<br>Month 4	VISIT 4<br><br>± 7 months<br>Month 5<br><br>Post III (M5)	VISIT 5<br><br>12-15 months<br>Month 10-13	VISIT 6<br><br>15-18 months<br>Month 13-16<br><br>Post III (M13)	VISIT 7<br><br>16-19 months<br>Month 14-17<br><br>Post IV (M14)	VISIT 8<br><br>18-21 months<br>Month 16-19	VISIT 9<br><br>24-27 months<br>Month 22-25<br><br>Post IV (M22)	CONTACT 10<br><br>Study end
Informed consent	●									
Check inclusion criteria	●									
Check exclusion criteria	●									
Demography, including gestational age	●									
Medical history	●									
Pre-vaccination history	●									
Check elimination criteria		●	●	●	●	●	●	●	●	
Check risk factors	●				●				●	
Check warnings, precautions and contraindications	O	O	O		O	O		O		
Physical examination	●	●	●	●	●	●	●	●	●	
Weight/height recording	●	●	●			●			●	
Pre-vaccination body temperature	●	●	●			●				
Randomization	●									
Blood sampling: for antibody determination (3.5 ml)				●		●	●		●	

Study visit<br>Age of subject<br>Study timing<br>Blood sampling timepoints	VISIT 1<br>6-16 weeks<br>Month 0	VISIT 2<br>± 4 months<br>Month 2	VISIT 3<br>± 6 months<br>Month 4	VISIT 4<br>± 7 months<br>Month 5<br>Post III (M5)	VISIT 5<br>12-15 months<br>Month 10-13	VISIT 6<br>15-18 months<br>Month 13-16<br>Post III (M13)	VISIT 7<br>16-19 months<br>Month 14-17<br>Post IV (M14)	VISIT 8<br>18-21 months<br>Month 16-19	VISIT 9<br>24-27 months<br>Month 22-25<br>Post IV (M22)	CONTACT 10<br>Study end
Vaccination (study and co-administered vaccines)	●	●	●			●				
Vaccination (concomitant vaccines)[1]					●			●		
Return of diary cards		O	O	O			O			
Diary card transcription		●	●	●			●			
Daily post-vaccination recording of solicited symptoms (Days 0-3) by subjects' parents/guardians	●	●	●			●				
Record any concomitant vaccination	●	●	●	●	●	●	●	●	●	
Reporting of unsolicited Adverse Events[2]		●	●	●	●	●	●	●	●	
Reporting of convulsions								●		
Reporting of Serious Adverse Events		●	●	●	●	●	●	●	●	●
Study Conclusion										●

Note:
● is used to indicate a study procedure that requires documentation in the individual CRF (eCRF).
O is used to indicate a study procedure that does not require documentation in the individual eCRF.
[1]Concomitant vaccines: in addition to the blinded study vaccines, subjects will be vaccinated with (1) MMR at the age of 12-15 months (according to local EPI), and (2) with HAV vaccine at the age of 12-15 months (visit 5) and at 18-21 months of age (since subjects in the control group receive a dose of HAV vaccine at visit 6, the last HAV dose should be administered at least 28 days after the stuy vaccine booster dose) (study benefit= vaccination against HAV at 18-21 months of age in Argentina and at 12-15 and 18-21 months of age in ~~Colombia and~~ Panama). (3) In Argentina all subjects will be vaccinated at 12-15 months of age against Neisseria meningitidis group C and in ~~Colombia and~~ Panama all subjects will be vaccinated against varicella at 12-15 months of age (study benefit= vaccination against Neisseria in Argentina and against varicella in ~~Colombia and~~ Panama).
[2]Only applicable for subjects enrolled in Panama

Section 5.4.3 Scheduled visits and study procedures for ‘Additional immuno’ subset:

Figure 4 Study design for the ‘Additional mmuno’ subset

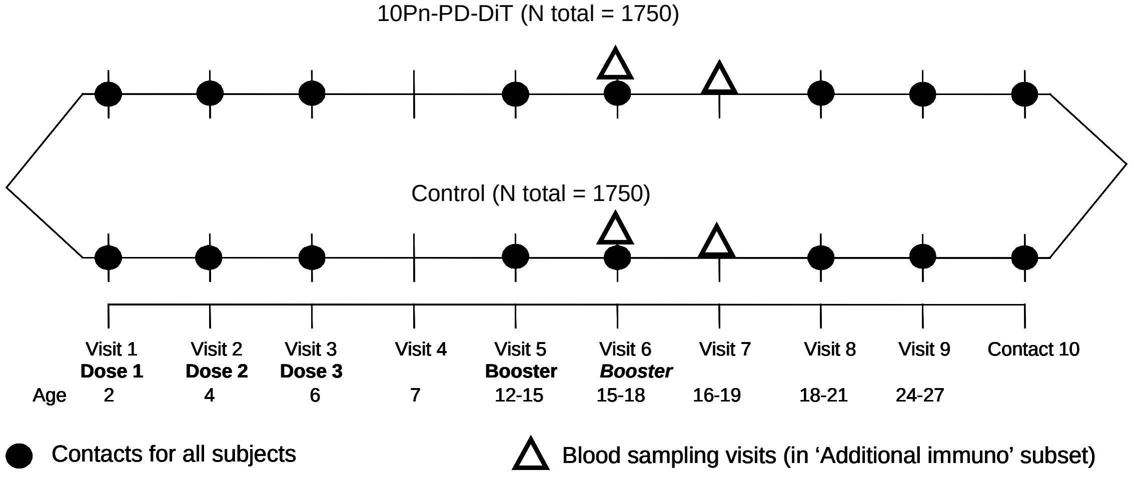

Table 8 List of study procedures for subjects participating in the ‘Additional immuno’ subset of the study

Study visit	VISIT 1	VISIT 2	VISIT 3	VISIT 4	VISIT 5	VISIT 6	VISIT 7	VISIT 8	VISIT 9	CONTACT 10
Age of subject	6-16 weeks	± 4 months	± 6 months	± 7 months	12-15 months	15-18 months	16-19 months	18-21 months	24-27 months	
Study timing	Month 0	Month 2	Month 4	Month 5	Month 10-13	Month 13-16	Month 14-17	Month 16-19	Month 22-25	Study end
Blood sampling timepoints				Post III (M5)		Post III (M13)	Post IV (M14)		Post IV (M22)	
Informed consent	●									
Check inclusion criteria	●									
Check exclusion criteria	●									
Demography, including gestational age	●									
Medical history	●									
Pre-vaccination history	●									
Check elimination criteria		●	●		●	●	●	●	●	
Check risk factors	●				●				●	
Check warnings, precautions and contraindications	O	O	O		O	O		O		
Physical examination	●	●	●		●	●	●	●	●	
Weight/height recording	●	●	●			●			●	
Pre-vaccination body temperature	●	●	●			●				
Randomization	●									

Study visit<br><br>Age of subject<br><br>Study timing<br><br>Blood sampling timepoints	VISIT 1<br><br>6-16 weeks<br>Month 0	VISIT 2<br><br>± 4 months<br>Month 2	VISIT 3<br><br>± 6 months<br>Month 4	VISIT 4<br><br>± 7 months<br>Month 5<br><br>Post III (M5)	VISIT 5<br><br>12-15 months<br>Month 10-13	VISIT 6<br><br>15-18 months<br>Month 13-16<br><br>Post III (M13)	VISIT 7<br><br>16-19 months<br>Month 14-17<br><br>Post IV (M14)	VISIT 8<br><br>18-21 months<br>Month 16-19	VISIT 9<br><br>24-27 months<br>Month 22-25<br><br>Post IV (M22)	CONTACT 10<br><br>Study end
Blood sampling: for antibody determination (3.5 ml)						●	●			
Vaccination (study and co-administered vaccines)	●	●	●			●				
Vaccination (concomitant vaccines)[1]					●			●		
Record any concomitant vaccination	●	●	●		●	●	●	●	●	
Reporting of unsolicited Adverse Events		●	●		●	●	●	●	●	
Reporting of convulsions[2]								●		
Reporting of Serious Adverse Events		●	●		●	●	●	●	●	●
Study Conclusion										●

Note:
● is used to indicate a study procedure that requires documentation in the individual CRF (eCRF).
O is used to indicate a study procedure that does not require documentation in the individual eCRF.
[1]Concomitant vaccines: in addition to the blinded study vaccines, subjects will be vaccinated with (1) MMR at the age of 12-15 months (according to local EPI, and (2) with HAV vaccine at the age of 12-15 months (visit 5) and at 18-21 months of age (since subjects in the control group receive a dose of HAV vaccine at visit 6, the last HAV dose should be administered at least 28 days after the stuy vaccine booster dose) (study benefit= vaccination against HAV ~~at 18-21 months of age in Argentina and~~ at 12-15 and 18-21 months of age in ~~Colombia and~~ Panama). (3) In ~~Argentina all subjects will be vaccinated at 12-15 months of age against Neisseria meningitidis group C and in Colombia and~~ Panama all subjects will be vaccinated against varicella at 12-15 months of age (study benefit= vaccination ~~against Neisseria in Argentina and~~ against varicella in ~~Colombia and~~ Panama).

Section 5.4.4 Scheduled visits and study procedures for 'Carriage' subset

Table 10 List of study procedures for subjects participating in the 'Carriage' subset of the study

Study visit<br><br>Age of subject<br><br>Study timing<br><br>Swab sampling timepoint	VISIT 1<br><br>6-16 weeks<br>Month 0	VISIT 2<br><br>± 4 months<br>Month 2	VISIT 3<br><br>± 6 months<br>Month 4	VISIT 4<br><br>± 7 months<br>Month 5<br><br>Post III (M5)	VISIT 5<br><br>12-15 months<br>Month 10-13<br><br>Post III (M10)	VISIT 6<br><br>15-18 months<br>Month 13-16<br><br>Post III (M13)	VISIT 7<br><br>16-19 months<br>Month 14-17<br><br>Post IV (M14)	VISIT 8<br><br>18-21 months<br>Month 16-19<br><br>Post IV (M16)	VISIT 9<br><br>24-27 months<br>Month 22-25<br><br>Post IV (M22)	CONTACT 10<br><br>Study end
Informed consent	●									
Check inclusion criteria	●									
Check exclusion criteria	●									

Study visit<br>Age of subject<br>Study timing<br>Swab sampling timepoint	VISIT 1<br>6-16 weeks<br>Month 0	VISIT 2<br>± 4 months<br>Month 2	VISIT 3<br>± 6 months<br>Month 4	VISIT 4<br>± 7 months<br>Month 5<br>Post III (M5)	VISIT 5<br>12-15 months<br>Month 10-13<br>Post III (M10)	VISIT 6<br>15-18 months<br>Month 13-16<br>Post III (M13)	VISIT 7<br>16-19 months<br>Month 14-17<br>Post IV (M14)	VISIT 8<br>18-21 months<br>Month 16-19<br>Post IV (M16)	VISIT 9<br>24-27 months<br>Month 22-25<br>Post IV (M22)	CONTACT 10<br><br>Study end
Demography, including gestational age	●									
Medical history	●									
Pre-vaccination history	●									
Check elimination criteria		●	●	●	●	●	●	●	●	
Check risk factors	●				●				●	
Check warnings, precautions and contraindications	O	O	O		O	O		O		
Physical examination	●	●	●	●	●	●	●	●	●	
Weight/height recording	●	●	●			●			●	
Pre-vaccination body temperature	●	●	●			●				
Randomization	●									
Nasopharyngeal swab samples				●	●	●	●	●	●	
Vaccination (study and co-administered vaccines)	●	●	●			●				
Vaccination (concomitant vaccines)[1]					●			●		
Record any concomitant vaccination	●	●	●	●	●	●	●	●	●	
Record any antibiotic treatment (diary card)		●	●	●	●	●	●	●	●	
Reporting of unsolicited Adverse Events		●	●	●	●	●	●	●	●	
Reporting of convulsions								●		
Reporting of Serious Adverse Events		●	●	●	●	●	●	●	●	●
Study Conclusion										●

Note:<br>● is used to indicate a study procedure that requires documentation in the individual CRF (eCRF).<br>O is used to indicate a study procedure that does not require documentation in the individual eCRF.<br>[1]Concomitant vaccines: in addition to the blinded study vaccines, subjects will be vaccinated with (1) MMR at the age of 12-15 months (according to local EPI), and (2) with HAV vaccine at the age of 12-15 months (visit 5) and at 18-21 months of age (since subjects in the control group receive a dose of HAV vaccine at visit 6, the last HAV dose should be administered at least 28 days after the stuy vaccine booster dose) (study benefit= vaccination against HAV ~~at 18-21 months of age in Argentina and~~ at 12-15 and 18-21 months of age in ~~Colombia and~~ Panama). (3) In ~~Argentina all subjects will be vaccinated at 12-15 months of age against *Neisseria meningitidis* group C and in Colombia and~~ Panama all subjects will be vaccinated against varicella at 12-15 months of age (study benefit= vaccination ~~against Neisseria in Argentina and~~ against varicella in ~~Colombia and~~ Panama).
**Section 5.5.1.1 Scheduled visits to be performed**
**Visit 4: at 7 months of age; Study Month 5**<br>'IMMUNO AND REACTO' or 'CARRIAGE' SUBSETS ONLY
~~Additional study procedures for SUBJECTS PART OF THE 'IMMUNO AND REACTO' or 'CARRIAGE' SUBSETS IN PANAMA~~
• ~~Return, verification and transcription of diary card with reporting of any adverse event since last visit.~~<br>• ~~A diary card will be distributed to the parents/guardians. They will be instructed to record any adverse event that may have occurred from the day of visit until the next visit in the appropriate section of the diary card.~~<br>~~The completed diary card will be given to the investigator at Visit 5.~~
**Visit 8: at 18-21 months of age; Study Month 16-19**
Study procedures for ALL SUBJECTS
• Check elimination criteria<br>• Check warning, precautions and contraindications to vaccination<br>• Physical examination<br>• ~~Recording of weight and height~~<br>• Record any concomitant vaccination (see Section 6.10)<br>• Record any serious adverse event that may have occurred since previous visit<br>• Reporting of convulsions<br>• The subject's parents/guardians will be instructed to contact the investigator immediately should their child manifest any signs or symptoms they perceive as serious.<br>• ***Concomitant vaccination: intramuscular administration of one dose of non-study HAV vaccine (Havrix), as set out in Section 6.3.2.***<br>***The vaccinees will be observed closely for at least 30 minutes with the appropriate medical treatment readily available in case of a rare anaphylactic reaction following vaccination.***

Section 5.5.2.1 Community Acquired Pneumonia (CAP) surveillance
Section 5.5.2.1.1 Definitions

The clinical suspicion of pneumonia is left to the judgment of the treating physician.

A case of suspected CAP involves either any subject who is referred to have a CXR performed as part of the clinical assessment of a febrile syndrome or an acute respiratory infection (ARI), or a hospitalized child who has a CXR performed within 2 days prior to, or within the first 3 days after hospital admission, as part of the clinical assessment of a febrile syndrome or an ARI.

Interpretation of the CXRs will be done by an independent panel of readers (see Section 5.5.2.1.2 for procedure and qualifications of images) based on the digitalized images and the concepts and definitions mentioned hereunder.

Chest X-ray (CXR) with **consolidation** is defined as a CXR with a dense, often homogeneous, confluent alveolar infiltrate that may encompass an entire lobe or segment, or a fluffy, mass-like, cloud-like density that erases heart and diaphragm borders (silhouette sign) and that often contains air bronchograms.

Pleural effusion is defined as a fluid collecting in the pleural space around the lung, seen radiologically as a dense rim (the same density as the chest-wall muscles) interposed between the lung and the ribs.

~~A case of **suspected CAP** involves either any subject who is referred to have a CXR performed as part of the clinical assessment of a febrile syndrome or an acute respiratory infection (ARI), or a hospitalized child who has a CXR performed within 2 days prior to, or within the first 3 days after hospital admission, as part of the clinical assessment of a febrile syndrome or an ARI.~~

Confirmed CAP is defined as a suspected CAP case whose CXR reveals the presence of abnormal pulmonary infiltrates as per the judgement of the independent Panel of readers. These abnormal pulmonary infiltrates can be either with or without alveolar consolidation/pleural effusion.

Section 5.5.2.1.2 Surveillance procedures
Digital CXR images from a suspected CAP cases in study participants will be sent to the Central Reading Panel for further classification (refer to X Ray Workbook). The reading of the image will be classified as (1) consolidated CAP, (2) non-consolidated CAP, (3) ~~normal CXR~~ ***no pneumonia*** or (4) uninterpretable CXR. For more details about the definitions of radiographic outcomes, please refer to Appendix F. All suspected CAP cases occurring in subjects participating in the study will be reported in the eCRF, regardless of the confirmation of the diagnosis by the local radiologist, the treating physician/pediatrician **or the Central X-ray Reading Panel of the study**.

For all CAP cases, the following investigations will be performed:

- It is recommended to perform blood culture for all CAP cases with consolidation according to the judgement of the treating physician, and according to the guidelines provided in the invasive disease surveillance section (see Section 5.5.2.3.3).
- A serum sample for CRP and procalcitonin (PCT) determination will be collected from all suspected CAP cases at the time or ~~maximum~~ ***at the latest*** within a maximum of 12 hours after ~~the~~ ***a*** chest X-ray was taken or within 12 hours after hospitalization ***and preferably before the first administration of any antibiotic***. Samples will be analyzed later at the Central Lab to measure CRP and PCT levels.

Section 5.5.2.2.3 Sample handling
In case of spontaneous/accidental rupture of the tympanic membrane, MEF should be collected ***within a maximum of 48 hours*** by aspiration in the ear canal using the CTD® Aspirator, attached to the CTD® Speculum. The ear canal should be thoroughly cleaned before the procedure to avoid masking of NTHi by local flora.

Section 5.5.2.3.3 Sample handling
Once the incubator detects a positive sample, process the sample immediately. Positive automated cultures (or other types of cultures) will be sub-cultured by plating onto chocolate and blood agar plates and incubated aerobically with 5-10% CO_2 at 35°C for 48 hours. In those laboratories where additional automated system is available, (Vytek), differentiation can be made through this system. If the sample became positive during the night and there is no trained person available, wait until the next shift to process the sample. If the blood culture bottle has been incubated for more than 10 hours after it has been detected to be positive, a dramatically low viable cells count has been described for pneumococci [Casetta et al, 1996]. Therefore remaining sample of each positive blood culture bottle should be divided into ~~3~~ ***2*** cryovials (1.5 mL each) and kept at -70°C for possible further analysis (see COMPAS Lab workbook).

Section 6.2 Co-administered and concomitant vaccines

The GSK Biologicals' DTPa-IPV/Hib, *Infanrix hexa*, ***Rotarix*** and *Varilrix* vaccines comply and *Neisvac-C* is assumed to comply with the specifications given in the manufacturer's Summary of Product Characteristics. See Table 15 for details on the study, co-administered and concomitant vaccines

Table 15 Vaccines administered in the study

Vaccine	Group	Age of administration (months)	Type of product	Blinding
Study vaccines				
10Pn-PD-DiT	10Pn-PD-DiT	2, 4, 6 and 15-18	Randomized (Investigational product)	Blinded
Engerix-B	Control	2, 4 and 6	Randomized (no commercial lot)	Blinded
Havrix	Control	15-18	Randomized (no commercial lot)	Blinded
Co-administered vaccines				
Infanrix hexa	10Pn-PD-DiT	2, 4 and 6	Randomized (no commercial lot)	Blinded
DTPa-IPV/Hib	10Pn-PD-DiT<br>Control	15-18<br>2, 4, 6 and 15-18	Randomized (no commercial lot)	Blinded
Concomitant vaccines*				
Havrix	All subjects	12 and 18-21	Commercial lot	Unblinded
Neisvac-C	All subjects in Argentina	12	Commercial lot	Unblinded
Varicella	All subjects in Panama	12	Commercial lot	Unblinded
Rotarix	*All subjects in Colombia*	*2 doses within the first 6 months of life*	*Commercial lot*	*Unblinded*

*These vaccines are benefits from the study

Other locally recommended vaccines (such as influenza or rotavirus vaccines, recommended either through the EPI program or through national immunization campaigns) are always allowed, even if ~~co-administered~~ ***concomitantly administered*** with the study vaccines, but should be documented in the eCRF.

Table 16 Formulation of Vaccines

Vaccine	Formulation (per dose)	Presentation	Vol. (mL)
GSK Biologicals' 10-valent Pn-PD-DIT vaccine	Protein D carrier: 1 μg of each PS for serotypes 1, 5, 6B, 7F, 9V, 14 and 23F and 3μg for serotype 4 conjugated to PD<br>Tetanus toxoid carrier with AH spacer: 3 μg of capsular PS of serotypes 18C conjugated to TT_{AH}<br>Diphtheria toxoid: 3 μg of capsular PS of serotype 19F conjugated to DT<br>Protein carrier content: ~12 μg PD, ~ 4.5 μg DT, ~ 7 μg TT<br>0.5 mg aluminium (Al^{3+}) as aluminium phosphate adjuvant	Whitish liquid in vial or prefilled syringe	0.5
GSK Biologicals' HAV vaccine (*Havrix*)	HAV (strain HM 175) 720 EL.U<br>Aluminium as salt: 0.25 mg	Whitish liquid in vial or prefilled syringe	0.5
GSK Biologicals' HBV vaccine (*Engerix-B*)	10μg of HBsAg, adsorbed on aluminium oxide; 0.25 mg Al^{3+}	Whitish liquid in prefilled syringe	0.5
GSK Biologicals' DTPa-HBV-IPV/Hib vaccine (*Infanrix hexa*)	Diphtheria toxoid ≥ 30IU (25Lf);<br>Tetanus toxoid ≥ 40IU (10Lf);<br>Pertussis antigens: PT 25 μg,<br>FHA 25 μg, PRN 8 μg;<br>Hepatitis B surface antigen (HBsAg): 10 μg;<br>Poliovirus type 1: 40 D antigen units, poliovirus type 2: 8 D antigen units, poliovirus type 3: 32 D antigen units;<br>PRP 10 μg conjugated to 20-40 μg tetanus toxoid<br>Phenoxyethanol ≤ 2.5 mg<br>Aluminium as salts 0.82 mg	DTPa-HBV-IPV component as a whitish liquid in vial or pre-filled syringes<br>+<br>Hib component as a white freeze-dried pellet in a monodose vial. to be reconstituted with DTPa-HBV-IPV vaccine	0.5
GSK Biologicals' DTPa-IPV/Hib vaccine	Diphtheria toxoid ≥ 30IU (25Lf);<br>Tetanus toxoid ≥ 40IU (10Lf);<br>Pertussis antigens: PT 25 μg,<br>FHA 25 μg, PRN 8 μg;<br>Poliovirus type 1: 40 D antigen units, poliovirus type 2: 8 D antigen units, poliovirus type 3: 32 D antigen units;<br>2-phenoxyethanol 2.5 mg<br>PRP 10 μg conjugated to 20-40 μg tetanus toxoid<br>Aluminium as salts 0.5 mg	DTPa-IPV component as a whitish liquid in vial or pre-filled syringes<br>+<br>Hib component as a white freeze-dried pellet in a monodose vial. to be reconstituted with DTPa-IPV vaccine	0.5
MenC vaccine (*Neisvac-C*)	*Neisseria meningitidis* group C (strain C11) polysaccharide (de-O-acetylated) conjugated to 10-20 μg tetanus toxoid adsorbed on 0.5 mg Al^{3+} (aluminium hydroxide)	A semi-opaque white to off-white suspension in vial or prefilled syringe	0.5
GSK Biologicals' varicella (*Varilrix*) vaccine	Live attenuated Oka strain varicella virus (≥ $10^{3.3}$pfu/dose)	Slightly pinkish freeze-dried pellets in vial + pre-filled syringe of diluent	0.5

Vaccine	Formulation (per dose)	Presentation	Vol. (mL)
GSK Biologicals' live oral attenuated HRV (Rotarix) vaccine	*Details on Rotarix vaccine formulation were blinded for Intellectual Property (IP) reasons.*	*Lyophilized vaccine in monodose glass vial. Diluent (composition blinded for IP reasons) supplied separately.*<br><br>*Liquid buffer in pre-filled syringe*	*NA*<br><br>*1.3*

Section 6.4 Storage

All investigational products to be administered to subjects must be stored in a safe and locked place with no access by unauthorized personnel.

Vaccines will be stored at the defined temperature range (i.e. +2 to +8°C).

The storage temperature of vaccines will be monitored daily while using validated temperature monitoring devices and the temperature measurements will be recorded during working days, preferably at the same time of the day (e.g. at the beginning of the day). Freezing indication will be continuously controlled by an appropriate device placed close to the vaccines.

~~The storage temperature of vaccines will be monitored and recorded daily during working days, preferably at the same time of the day (e.g. at the beginning of the day).~~

~~At a minimum, a calibrated/validated min/max thermometer will be placed close to the vaccines and will be used to monitor and record the daily temperature (actual, min and max temperatures will be logged). Additionally, a continuous temperature monitoring device will be used as a back up device and it will be opened in case of any temperature deviation (temperature outside the defined range, i.e. +2 to +8°C) during weekends or holidays. Alternatively, another temperature monitoring system of the storage facility can be used (as a replacement of the GSK continuous temperature monitoring device), if:~~

- ~~proper functioning was demonstrated during the monitor's site evaluation,~~
- ~~if the system continues to work in case of a power failure, and~~
- ~~if the system is maintained regularly (e.g. once/year) as documented in the site files.~~

~~It is also permitted to monitor the storage temperature using a validated temperature continuous recording device, provided it can read the daily actual and min/max temperatures, and that it keeps working after the alarm is activated.~~

~~It is also required to place a validated freezing point indicator close to the vaccines as a back-up device.~~

Any temperature deviation, i.e. temperature outside the defined range (i.e. +2 to +8°C), must be reported within 24 hours to the sponsor (i.e. Study Monitor/ GSK Local Contact/ GSK Biologicals).<br><br>Following exposure to a temperature deviation, vaccines will not be used until written approval is given by the sponsor.<br><br>Storage conditions for transport of vaccines from country medical department or dispatch centre to study sites or between sites are described in Appendix E and according to the local SOP about management of clinical trial supplies.
and according to the local SOP about management of clinical trial supplies.
**Section 6.10 Concomitant medication/treatment/vaccination**
Study procedures for ALL SUBJECTS
Any recommended vaccine and any vaccine not foreseen in the study protocol administered since birth and during the entire study period is to be recorded with trade name ***(if the trade name is not available, the generic name is allowed)***, route of administration and date(s) of administration. Refer to Sections 4.3, 5.4 and 6.11.
Section 6.11.3 GlaxoSmithKline Biologicals' Rotarix vaccine<br><br>***To prepare the oral live attenuated HRV (Rotarix) vaccine for administration, the entire content of the prefilled syringe (diluent) should be injected into the vial of the lyophilized product. The vial should be shaken well to resuspend the vaccine. The entire volume of the resuspended product should be withdrawn into the same syringe and the resuspended product should then be administered promptly as a single ORAL dose. Rotarix should under no circumstances be injected.***<br><br>***Should the subject regurgitate or vomit after the vaccine administration, a new vaccine dose may be administered at that visit.***<br><br>***Section 6.11.3.1 Contraindications***<br><br>***This vaccine is contra-indicated in subjects with hypersensitivity to any component of the vaccine:***<br><br>• ***Known hypersensitivity after previous administration of the HRV vaccine or to any component of the vaccine.***<br>• ***History of chronic gastrointestinal disease including any uncorrected congenital malformation of the gastrointestinal tract.***<br>• ***Previous history of intussusception (IS).***<br><br>***Administration of Rotarix should be postponed for subjects suffering from an acute severe febrile illness or from a gastroenteritis (GE) within 7 days preceding the study vaccine administration (i.e. diarrhoea with or without vomiting).***

Section 7.5 Time period, frequency, and method of detecting adverse events and serious adverse events

Study procedures for ALL SUBJECTS

For all subjects, even if not part of the subjects enrolled in Panama, AEs have to be documented in case***:*** 1) this AE is a reason for withdrawal of the subject from the study or from study vaccination, or 2) the AE refers to a convulsion episode occurring within 30 days after booster vaccination (Visit 6), and to be reported at Visit 8. Any case of convulsions occurring within 30 days after ~~10Pn-PD-DiT~~ ~~booster~~ vaccin~~ation~~***e administration at Visit 6*** should be recorded in a specific eCRF page**.** Otherwise, safety assessment in subjects who are not part of the 'Immuno and reacto' subset is limited to the SAE reporting.

Section 9.3.1.1 Primary objective (B-CAP or C-AOM)

AOM endpoints will be assessed in all 7,000 subjects enrolled in Panama City (3,500 subjects per group). Considering that at any point in the study, we will have at least as many C-AOM episodes from the 7,000 subjects enrolled in Panama, as we have B-CAP from the total 24,000 subject cohort (Argentina**,** Colombia and Panama combined), the ***number of*** subjects enrolled in Panama will be large enough to also reach at least 1200 first C-AOM episodes at study end**.** Based on the same incidence rates, we can anticipate to reach the required number of 628 first episodes for the interim analysis approximately 20 months after study start.

Appendix E Vaccine supplies, packaging and accountability

In Colombia, 2 doses of licensed Rotarix vaccine will be offered to all subjects within the first six months of life, supplied in monodose vials and prefilled syringes.

Labels for sample identification:

The investigator will receive labels from GSK Biologicals to identify samples taken from each subject from the 'Immuno and reacto' subset at each timepoint . Each label will contain the following information: study number, identification number for the subject (e.g. **subject number**), sampling timepoint (e.g., post vacc 3), timing (e.g., study Month 5). ***In addition GSK Biologicals will provide labels to identify samples taken from each subject in the 'Additional Immuno' subset.***

Vaccine packaging

The vaccines will be packed in labelled boxes. Each treatment will consist of ~~2~~ ***one*** box~~es: one~~ containing the study vaccine/control and ~~the other containing~~ the co-administered vaccine. To differentiate between the vaccines, a specifically coloured label will be used on the syringes of the study vaccine/control. The box label will contain, as a minimum, the following information: study number, treatment number, lot number, instructions for vaccine administration and any other local relevant regulatory requirements.

Vaccine accountability

After approval from GSK Biologicals and in accordance with ~~GSK SOP WWD-1102~~ ***GSK SOP WWD-1100***, used and unused vaccine vials/syringes should be destroyed at the study site using locally approved biosafety procedures and documentation unless otherwise described in the protocol. If no adequate biosafety procedures are available at the study site, the used and unused vaccine vials/syringes are to be returned to an appropriate GSK Biologicals site for destruction, also in accordance with current ~~GSK SOP WWD-7054~~ ***GSK SOP WWD-1100***.

Appendix F Defenitions of radiographic outcomes and guidelines for radiographic techniques

- ~~Normal CXR~~ ***No pneumonia***: absence of consolidation, pleural effusion and/or abnormal infiltrates on the digital CXR image.

GlaxoSmithKline Biologicals

Clinical Research & Development
Protocol Amendment Approval

**eTrack study number**	109563
**eTrack abbreviated title**	10PN-PD-DIT-028
**Protocol title:**	Clinical Otitis Media and Pneumonia Study (**COMPAS**): a phase III , double-blind, randomized, controlled, multicentre study to demonstrate the efficacy of GlaxoSmithKline (GSK) Biologicals' 10-valent pneumococcal conjugate vaccine (GSK1024850A) against Community Acquired Pneumonia (CAP) and Acute Otitis Media (AOM)
**Amendment number:**	Amendment 3
**Amendment date:**	25 November 2008
**Co-ordinating author:**	Mireille Venken, Scientific Writer

Rationale/background for changes: Amendment 3 of the COMPAS protocol was developed in reply to questions from local authorithies:

- The recruitment period has been extended to 18 months.
- High risk groups will be excluded from the study in case a specific local vaccination program is available.
- The informed consent process for minors (<18 years of age) has been specified.
- Additional minor changes and clarifications have been implemented.

Amended text has been included in *bold italics* in the following section(s):

Title Page:

**Contributing authors**	• Juan Pablo Yarzabal MD, MSc, Clinical Development Manager<br>• Leyla Hernandez, Raquel Merino, ***Kaat Vandorpe*** Central Study Co-ordinator***s***<br>• Isabelle Henckaerts, Scientist, Clinical Immunology<br>• Lode Schuerman MD, Director Clinical Development (Pneumococcal Vaccines)<br>• Patricia Lommel, Biostatistician<br>• William Hausdorff PhD, Director Epidemiology<br>• Ricardo Ruttiman MD, Clinical Director R&D and Medical Affairs Argentina and Southern Cone

**Synopsis**	
**Study Design**	The target recruitment time will be approximately ~~12~~ ***18*** months (depending on the time needed to complete target enrolment), starting from first subject enrolled in the study.

3.1 Study design:

Target recruitment time will be approximately ~~12~~ ***18*** months (depending on the time needed to complete target enrolment), starting from first subject enrolled**.**

4.2 Exclusion criteria for enrolment:

- ***For Colombia: infants with low birth weight (<2.500g).***

5.1.2 Informed Consent

The Informed Consent Form must be in a language fully comprehensible to the prospective subjects' parents/guardians. Informed consent shall be documented by the use of a written consent form approved by the IRB/IEC and signed and dated by the parents/guardians and by the person who conducted the informed consent discussion. The signature confirms the consent is based on information that has been understood. ***Specific attention should be given to:***

- ***Illiterate individuals***: all illiterate individuals will have the study, and the Informed Consent Form explained to them point by point by the interviewer in the presence of ***at least one*** impartial witness ***following local regulations***. The witness will personally sign and date the consent form. Oral witnessed consent will replace written consent only in countries where the local custom is contrary or if the parents'/guardians' incapacity precludes this and provided that the local legal obligations are fulfilled.
- ***Minors (< 18 years of age): local law should be applied***.

5.4.3 Scheduled visits and study procedures for 'Additional immuno' subset

Subjects enrolled in the 'Additional immuno' subset will have the same seven scheduled visits and final study conclusion contact as all subjects plus ~~two~~ ***one*** additional scheduled visits (see Figure 4 and Table 8). A group of 3,500 subjects in Panama will be part of this subset. A first blood sample will be obtained at Visit 6, just before the booster injection, while the second blood sample will be taken one month after the administration of the booster vaccination (at visit 7).

5.5.2.1.3 Sample handling at local laboratories

Nasopharyngeal aspirate

Whenever possible and within 5 days after initiation of symptoms, nasopharyngeal aspirate samples will be obtained from all suspected CAP cases ~~before the initiation of any antibiotic treatment~~.

5.5.2.1.5 Data flow

Digital CXR images of ***all*** suspected CAP cases will be sent to the Central Reading Panel. Results of the interpretations by the Central Reading Panel will be transferred directly to GSK, and will not be sent back to the study investigators, since only the local reading of the CXR according to local routine practices will guide diagnostic and therapeutic decisions (Refer to COMPAS X Ray Workbook)**.**

5.5.2.1.6 Case accountability

All suspected CAP cases occurring during the entire study must be reported as described in section 5.5.2.1.2.

For CAP, the start date of a clinical episode corresponds to the date when symptoms first were observed. In case this date is not known or defined, the start date will be the date when the CXR was taken. ***From an analysis point of view,*** ~~A~~ ***a*** new CAP episode will be considered to have started if 30 days or more have elapsed since the start date of the previous CAP episode. If less than 30 days have elapsed since the start date of the previous CAP episode, the new CXR will be considered related to the same CAP episode. If for the same CAP episode, the outcome of consecutive CXR readings evolve over time, the most serious diagnosis will be taken into consideration**.**

5.5.2.3 Invasive disease surveillance

5.5.2.3.1 Case definitions

Probable bacterial meningitis due to *S. pneumoniae* or *H. influenzae*:

– Purulent meningitis with negative result of CSF and blood culture but with antigen testing (Latex or Binax) positive for *S. pneumoniae* or *H. influenzae* in CSF.

– ***Meningitis with blood culture positive for S. pneumoniae or H. influenzae, with negative result of CSFculture but antigen testing (Latex or Binax) positive for S. pneumoniae or H. influenzae in CSF.***

5.5.2.2.6 Case accountability

- ***From an analysis point of view,*** ~~I~~***i***f an AOM visit occurs at least 30 days after the date of confirmation by the ENT specialist of a previous AOM episode, it will be considered as a new episode**.**

5.5.2.3.6 Case accountability

From an analysis point of view, ~~I~~***i***f the same pneumococcal or *H. influenzae* serotype is isolated with an interval of less than 30 days, both isolates will be considered related to the same invasive disease episode. If the pneumococcus or *H. influenzae* isolate does not share the same serotype, or if 30 days or more have elapsed since previous isolation, it will be considered as a new invasive disease episode.

7.12 Treatment of adverse events

Treatment of any adverse event is at the sole discretion of the investigator and according to local medical practice. ***Specific medication will be made available by the Sponsor to comply with existing local programs for treatment of the most common adverse events related to vaccination.***

GlaxoSmithKline Biologicals

Clinical Research & Development

Protocol Amendment Approval

**eTrack study number**	109563
**eTrack abbreviated title**	10PN-PD-DIT-028
**Protocol title:**	Clinical Otitis Media and Pneumonia Study (**COMPAS**): a phase III , double-blind, randomized, controlled, multicentre study to demonstrate the efficacy of GlaxoSmithKline (GSK) Biologicals' 10-valent pneumococcal conjugate vaccine (GSK1024850A) against Community Acquired Pneumonia (CAP) and Acute Otitis Media (AOM)
**Amendment number:**	Amendment 4
**Amendment date:**	11 May 2009
**Co-ordinating author:**	Mireille Venken, Scientific Writer

Rationale/background for changes: Amendment 4 of the COMPAS protocol was developed on request of local authorities:

- In the exclusion criteria for further study participation the citeria for high risk for pneumococcal disease have been specified and clarification on how to proceed in case of diagnosis of a high risk condition for pneumococcal infection has been added.

Amended text has been included in *bold italics* in the following section(s):

Title page

**Contributing authors**	• Juan Pablo Yarzabal MD, MSc, Clinical Development Manager<br>• Leyla Hernandez, Raquel Merino, Kaat Vandorpe, ***Frederic Henry***, ***Global Study Managers*** ~~Central Study Co-ordinators~~<br>• Isabelle Henckaerts, Scientist, Clinical Immunology<br>• Lode Schuerman MD, Director Clinical Development (Pneumococcal Vaccines)<br>• Patricia Lommel, Biostatistician<br>• William Hausdorff PhD, Director Epidemiology<br>• Ricardo Ruttiman MD, ***Alejandro Lepetic MD***, Clinical Director R&D and Medical Affairs Argentina and Southern Cone

List of abbreviations

~~CSC~~ ***GSM***	~~Central Study Coordinator~~ ***Global Study Manager***

**Glossary of Terms**	
~~**Central Study Co-ordinator:**~~ ***Global Study Manager***	An individual assigned by and centrally located at GSK Biologicals at Rixensart who is responsible for assuring the co-ordination of the operational aspects and proper conduct of a clinical study, including compliance with International Conference on Harmonization (ICH) Harmonized Tripartite Guideline for Good Clinical Practice (GCP) and GSK policies and standard operating procedures.

Section 4.4 Exclusion criteria for further study participation

- ***High risk disease for pneumococcal infections is defined by the guidelines of the American Academy of Paediatrics [American Academy of Pediatrics, 2000] and also the Argentinean Paediatric Society [Normas Nacionales de Vacunacion, 2008] (see*** *Table 4****).*** Diagnosis of high risk disease for pneumococcal infections ~~(such as HIV positive, sickle cell disease and splenectomized infants)~~ will be evaluated according to medical judgement ***(as established by medical history and clinical examination) and confirmed when needed by the appropriate test(s).*** ~~Decision on withdrawal of the subject from the study will depend on the medical condition of the subject, the existence of a specific local vaccination program, and the availability of the registered pneumococcal vaccine. If Prevnar is made available through a targeted immunization program for these high risk groups, further participation in the COMPAS study would no longer be possible, and these subjects will need to be excluded from the study and referred to the specific Prevnar immunization program. Once excluded, subjects can not return to receive subsequent study vaccine doses, even if Prevnar would no longer be available.~~ ***In case of diagnosis of a high risk condition for pneumococcal infection requiring pneumococcal conjugate vaccination in a subject enrolled in the study, the subject will be unblinded in order to guide appropriate individual management of the case:***

- ***All subjects will continue study vaccination and follow-up according to the procedures planned in the protocol. The date of unblinding for these subjects will be encoded in the e CRF and efficacy follow-up data collected after this date will no longer contribute to the primary efficacy analysis.***
- ***Subjects randomized to the 10Pn-PD-DiT vaccine group will not receive additional vaccines, since they already received pneumococcal conjugate vaccination in addition to the DTPa-HBV-IPV/Hib study vaccine.***
- ***Subjects randomized to the control group will need to receive a licensed pneumococcal conjugate vaccine according to the age-appropriate immunization schedule, in addition to the DTPa-IPV/Hib, HBV and HAV study vaccines.***
- ***If a licensed Pneumococcal conjugate vaccine is made available through a targeted national immunization program for these high risk groups, the investigator will make sure that the subjects belonging to these high risk groups will receive that vaccine. If no locally licensed Pneumococcal conjugate vaccine is available, it will be provided to the investigator by GSK Biologicals.***
- ***Administration of the licensed pneumococcal conjugate vaccine will be documented in the e CRF.***

- For each case, where by mistake, vaccines with antigens common to the antigens contained in the study or co-administered vaccines are administered outside of the context of the study, the investigator will need to evaluate whether the subject can still continue participation in the study.

Table 4	***Children at High Risk of Invasive Pneumococcal Infection****
High risk	
1	*Sickle Cell Disease (SCD), congenital or acquired asplenia, or splenic dysfunction*
2	*Infection with HIV*
Presumed high risk (attack rate not calculated)	
1	*Congenital immune deficiency: some B- (humoral) or T-lymphocyte deficiencies, complement deficiencies (particularly C1, C2, C3, and C4 deficiencies), or phagocytic disorders (excluding chronic granulomatous disease).*
2	*Chronic cardiac disease (particularly cyanotic congenital heart disease and cardiac failure)*
3	*Chronic pulmonary disease (including cystic fibrosis, rheumatic pneumonia, tuberculosis, idiopatic intersticial difuse fibrosis of the lung, pulmonary aspergilosis and asthma treated with high-dose oral corticosteroid therapy defined as more than 14 days of prednisone or* ***equivalent, ≥0.5 mg/kg/day. Inhaled and topical steroids are*** *allowed)*
4	*Cerebrospinal fluid leaks*
5	*Chemotherepy*
6	*Chronic renal insufficiency, including nephrotic syndrome*
7	*Chronic hepatopathy*
8	*Diseases associated with immunosuppressive therapy or radiation therapy (including malignant neoplasms, leukemias, lymphomas, and Hodgkin's disease) and solid organ transplantation***
9	*Diabetes mellitus*
10	*Multiple myeloma*
11	*Preterm infants (born after gestation period ≤ 32 weeks) and/or a birth weight ≤ 1.500g*
** Adapted from [American Academy of Pediatrics, 2000] and [Normas Nacionales de Vacunacion, 2008]*	

Section 5.4.1 Scheduled visits and study procedures

Table 5 List of study procedures applicable for all subjects

Study visit<br><br>Age of subject<br><br>Study timing	VISIT 1<br><br>6-16 weeks<br>Month 0	VISIT 2<br><br>± 4 months<br>Month 2	VISIT 3<br><br>± 6 months<br>Month 4	VISIT 4[1]<br><br>± 7 months<br>Month 5	VISIT 5<br><br>12-15 months<br>Month 10-13	VISIT 6<br><br>15-18 months<br>Month 13-16	VISIT 7[1]<br><br>16-19 months<br>Month 14-17	VISIT 8<br><br>18-21 months<br>Month 16-19	VISIT 9<br><br>24-27 months<br>Month 22-25	CONTACT 10<br><br>Study end
Informed consent	●									
Check inclusion criteria	●									
Check exclusion criteria	●									
Demography, including gestational age	●									
Medical history	●									
Pre-vaccination history	●									
Check elimination criteria		●	●		●	●		●	●	

Study visit<br><br>Age of subject<br><br>Study timing	VISIT 1<br><br>6-16 weeks<br>Month 0	VISIT 2<br><br>± 4 months<br>Month 2	VISIT 3<br><br>± 6 months<br>Month 4	VISIT 4[1]<br><br>± 7 months<br>Month 5	VISIT 5<br><br>12-15 months<br>Month 10-13	VISIT 6<br><br>15-18 months<br>Month 13-16	VISIT 7[1]<br><br>16-19 months<br>Month 14-17	VISIT 8<br><br>18-21 months<br>Month 16-19	VISIT 9<br><br>24-27 months<br>Month 22-25	CONTACT 10<br><br><br>Study end
Check exclusion criteria for further study participation		*O*	*O*		*O*	*O*		*O*	*O*	
Check risk factors	●				●				●	
Check warnings, precautions and contraindications	O	O	O		O	O		O		
Physical examination	●	●	●		●	●		●	●	
Weight/height recording	●	●	●			●			●	
Pre-vaccination body temperature	●	●	●			●				
Randomization	●									
Vaccination (study and co-administered vaccines)	●	●	●			●				
Vaccination (concomitant vaccines)[2]					●			●		
Record any concomitant vaccination	●	●	●		●	●		●	●	
Reporting of unsolicited Adverse Events[3]		●	●		●	●		●	●	
Reporting of convulsions[4]								●		
Reporting of Serious Adverse Events		●	●		●	●		●	●	●
Study Conclusion										●

Note:
● is used to indicate a study procedure that requires documentation in the individual CRF (eCRF).
O is used to indicate a study procedure that does not require documentation in the individual eCRF.
[1]Study activities during grey-coloured visits are only applicable for subjects in the 'Immuno and reacto' and/or the 'Carriage' subsets (see Table 6 and Table 8, respectively)
[2]Concomitant vaccines: in addition to the blinded study vaccines, subjects will be vaccinated with *(1)* MMR at the age of 12-15 months (according to local EPI), and *(2)* with HAV vaccine at the age of 12-15 months (visit 5) and at 18-21 months of age (since subjects in the control group receive a dose of HAV vaccine at visit 6, the last HAV dose should be administered at least 28 days after the stuy vaccine booster dose) (study benefit= vaccination against HAV at 18-21 months of age in Argentina and at 12-15 and 18-21 months of age in Panama and Colombia). *(3)* In Argentina all subjects will be vaccinated at 12-15 months of age against *Neisseria meningitidis* group C and in Colombia and Panama all subjects will be vaccinated against varicella at 12-15 months of age (study benefit= vaccination against Neisseria in Argentina and against varicella in Colombia and Panama). (4) In Colombia all subjects will be vaccinated against HRV at visit 2 and visit 3 (study benefit= vaccination against HRV).
[3]Only applicable for subjects enrolled in Panama
[4]Applicable for subjects enrolled in Argentina, Colombia and Panama

Section 5.4.2 Scheduled visits and study procedures for 'Immuno and reacto' subset

Table 7 List of study procedures for subjects participating in the 'Immuno and reacto' subset of the study

Study visit	VISIT 1	VISIT 2	VISIT 3	VISIT 4	VISIT 5	VISIT 6	VISIT 7	VISIT 8	VISIT 9	CONTACT 10
Age of subject	6-16 weeks	± 4 months	± 6 months	± 7 months	12-15 months	15-18 months	16-19 months	18-21 months	24-27 months	
Study timing	Month 0	Month 2	Month 4	Month 5	Month 10-13	Month 13-16	Month 14-17	Month 16-19	Month 22-25	Study end
Blood sampling timepoints				Post III (M5)		Post III (M13)	Post IV (M14)		Post IV (M22)	
Informed consent	●									
Check inclusion criteria	●									
Check exclusion criteria	●									
Demography, including gestational age	●									
Medical history	●									
Pre-vaccination history	●									
Check elimination criteria		●	●	●	●	●	●	●	●	
Check exclusion criteria for further study participation		*O*	*O*	*O*	*O*	*O*	*O*	*O*	*O*	
Check risk factors	●				●				●	
Check warnings, precautions and contraindications	O	O	O		O	O		O		
Physical examination	●	●	●	●	●	●	●	●	●	
Weight/height recording	●	●	●			●			●	
Pre-vaccination body temperature	●	●	●			●				
Randomization	●									
Blood sampling: for antibody determination (3.5 ml)				●		●	●		●	
Vaccination (study and co-administered vaccines)	●	●	●			●				
Vaccination (concomitant vaccines)[1]					●			●		
Return of diary cards		O	O	O			O			
Diary card transcription		●	●	●			●			
Daily post-vaccination recording of solicited symptoms (Days 0-3) by subjects' parents/guardians	●	●	●			●				

Study visit<br>Age of subject<br>Study timing<br>Blood sampling timepoints	VISIT 1<br>6-16 weeks<br>Month 0	VISIT 2<br>± 4 months<br>Month 2	VISIT 3<br>± 6 months<br>Month 4	VISIT 4<br>± 7 months<br>Month 5<br>Post III (M5)	VISIT 5<br>12-15 months<br>Month 10-13	VISIT 6<br>15-18 months<br>Month 13-16<br>Post III (M13)	VISIT 7<br>16-19 months<br>Month 14-17<br>Post IV (M14)	VISIT 8<br>18-21 months<br>Month 16-19	VISIT 9<br>24-27 months<br>Month 22-25<br>Post IV (M22)	CONTACT 10<br>Study end
Record any concomitant vaccination	●	●	●	●	●	●	●	●	●	
Reporting of unsolicited Adverse Events[2]		●	●	●	●	●	●	●	●	
Reporting of convulsions								●		
Reporting of Serious Adverse Events		●	●	●	●	●	●	●	●	●
Study Conclusion										●

Note:
● is used to indicate a study procedure that requires documentation in the individual CRF (eCRF).
O is used to indicate a study procedure that does not require documentation in the individual eCRF.
[1]Concomitant vaccines: in addition to the blinded study vaccines, subjects will be vaccinated with (1) MMR at the age of 12-15 months (according to local EPI), and (2) with HAV vaccine at the age of 12-15 months (visit 5) and at 18-21 months of age (since subjects in the control group receive a dose of HAV vaccine at visit 6, the last HAV dose should be administered at least 28 days after the stuy vaccine booster dose) (study benefit= vaccination against HAV at 18-21 months of age in Argentina and at 12-15 and 18-21 months of age in Panama). (3) In Argentina all subjects will be vaccinated at 12-15 months of age against Neisseria meningitidis group C and in Panama all subjects will be vaccinated against varicella at 12-15 months of age (study benefit= vaccination against Neisseria in Argentina and against varicella in Panama).
[2]Only applicable for subjects enrolled in Panama

Section 5.4.3 Scheduled visits and study procedures for 'Additional immuno' subset

Table 9 List of study procedures for subjects participating in the 'Additional immuno' subset of the study

Study visit<br>Age of subject<br>Study timing<br>Blood sampling timepoints	VISIT 1<br>6-16 weeks<br>Month 0	VISIT 2<br>± 4 months<br>Month 2	VISIT 3<br>± 6 months<br>Month 4	VISIT 4<br>± 7 months<br>Month 5<br>Post III (M5)	VISIT 5<br>12-15 months<br>Month 10-13	VISIT 6<br>15-18 months<br>Month 13-16<br>Post III (M13)	VISIT 7<br>16-19 months<br>Month 14-17<br>Post IV (M14)	VISIT 8<br>18-21 months<br>Month 16-19	VISIT 9<br>24-27 months<br>Month 22-25<br>Post IV (M22)	CONTACT 10<br>Study end
Informed consent	●									
Check inclusion criteria	●									
Check exclusion criteria	●									
Demography, including gestational age	●									
Medical history	●									
Pre-vaccination history	●									

Study visit<br>Age of subject<br>Study timing<br>Blood sampling timepoints	VISIT 1<br>6-16 weeks<br>Month 0	VISIT 2<br>± 4 months<br>Month 2	VISIT 3<br>± 6 months<br>Month 4	VISIT 4<br>± 7 months<br>Month 5<br>Post III (M5)	VISIT 5<br>12-15 months<br>Month 10-13	VISIT 6<br>15-18 months<br>Month 13-16<br>Post III (M13)	VISIT 7<br>16-19 months<br>Month 14-17<br>Post IV (M14)	VISIT 8<br>18-21 months<br>Month 16-19	VISIT 9<br>24-27 months<br>Month 22-25<br>Post IV (M22)	CONTACT 10<br><br>Study end
Check elimination criteria		●	●		●	●	●	●	●	
Check exclusion criteria for further study participation		*O*	*O*		*O*	*O*	*O*	*O*	*O*	
Check risk factors	●				●				●	
Check warnings, precautions and contraindications	O	O	O		O	O		O		
Physical examination	●	●	●		●	●	●	●	●	
Weight/height recording	●	●	●			●			●	
Pre-vaccination body temperature	●	●	●			●				
Randomization	●									
Blood sampling: for antibody determination (3.5 ml)						●	●			
Vaccination (study and co-administered vaccines)	●	●	●			●				
Vaccination (concomitant vaccines)[1]					●			●		

Study visit<br>Age of subject<br>Study timing<br>Blood sampling timepoints	VISIT 1<br>6-16 weeks<br>Month 0	VISIT 2<br>± 4 months<br>Month 2	VISIT 3<br>± 6 months<br>Month 4	VISIT 4<br>± 7 months<br>Month 5<br>Post III (M5)	VISIT 5<br>12-15 months<br>Month 10-13	VISIT 6<br>15-18 months<br>Month 13-16<br>Post III (M13)	VISIT 7<br>16-19 months<br>Month 14-17<br>Post IV (M14)	VISIT 8<br>18-21 months<br>Month 16-19	VISIT 9<br>24-27 months<br>Month 22-25<br>Post IV (M22)	CONTACT 10<br>Study end
Record any concomitant vaccination	●	●	●		●	●	●	●	●	
Reporting of unsolicited Adverse Events		●	●		●	●	●	●	●	
Reporting of convulsions[2]								●		
Reporting of Serious Adverse Events		●	●		●	●	●	●	●	●
Study Conclusion										●

Note:
● is used to indicate a study procedure that requires documentation in the individual CRF (eCRF).
O is used to indicate a study procedure that does not require documentation in the individual eCRF.
[1]Concomitant vaccines: in addition to the blinded study vaccines, subjects will be vaccinated with (1) MMR at the age of 12-15 months (according to local EPI, and (2) with HAV vaccine at the age of 12-15 months (visit 5) and at 18-21 months of age (since subjects in the control group receive a dose of HAV vaccine at visit 6, the last HAV dose should be administered at least 28 days after the stuy vaccine booster dose) (study benefit= vaccination against HAV at 12-15 and 18-21 months of age in Panama). (3) In Panama all subjects will be vaccinated against varicella at 12-15 months of age (study benefit= vaccination against varicella in Panama).

Section 5.4.4 Scheduled visits and study procedures for 'Carriage' subset

Table 11 List of study procedures for subjects participating in the 'Carriage' subset of the study

Study visit<br>Age of subject<br>Study timing<br>Swab sampling timepoint	VISIT 1<br>6-16 weeks<br>Month 0	VISIT 2<br>± 4 months<br>Month 2	VISIT 3<br>± 6 months<br>Month 4	VISIT 4<br>± 7 months<br>Month 5<br>Post III (M5)	VISIT 5<br>12-15 months<br>Month 10-13<br>Post III (M10)	VISIT 6<br>15-18 months<br>Month 13-16<br>Post III (M13)	VISIT 7<br>16-19 months<br>Month 14-17<br>Post IV (M14)	VISIT 8<br>18-21 months<br>Month 16-19<br>Post IV (M16)	VISIT 9<br>24-27 months<br>Month 22-25<br>Post IV (M22)	CONTACT 10<br>Study end
Informed consent	●									
Check inclusion criteria	●									
Check exclusion criteria	●									
Demography, including gestational age	●									
Medical history	●									
Pre-vaccination history	●									
Check elimination criteria		●	●	●	●	●	●	●	●	

Study visit<br><br>Age of subject<br><br>Study timing<br><br>Swab sampling timepoint	VISIT 1<br><br>6-16 weeks<br><br>Month 0	VISIT 2<br><br>± 4 months<br><br>Month 2	VISIT 3<br><br>± 6 months<br><br>Month 4	VISIT 4<br><br>± 7 months<br><br>Month 5<br><br>Post III (M5)	VISIT 5<br><br>12-15 months<br><br>Month 10-13<br><br>Post III (M10)	VISIT 6<br><br>15-18 months<br><br>Month 13-16<br><br>Post III (M13)	VISIT 7<br><br>16-19 months<br><br>Month 14-17<br><br>Post IV (M14)	VISIT 8<br><br>18-21 months<br><br>Month 16-19<br><br>Post IV (M16)	VISIT 9<br><br>24-27 months<br><br>Month 22-25<br><br>Post IV (M22)	CONTACT 10<br><br><br><br>Study end
Check exclusion criteria for further study participation		*O*	*O*	*O*	*O*	*O*	*O*	*O*	*O*	
Check risk factors	●				●				●	
Check warnings, precautions and contraindications	O	O	O		O	O		O		
Physical examination	●	●	●	●	●	●	●	●	●	
Weight/height recording	●	●	●			●			●	
Pre-vaccination body temperature	●	●	●			●				
Randomization	●									
Nasopharyngeal swab samples				●	●	●	●	●	●	
Vaccination (study and co-administered vaccines)	●	●	●			●				
Vaccination (concomitant vaccines)[1]					●			●		
Record any concomitant vaccination	●	●	●	●	●	●	●	●	●	
Record any antibiotic treatment (diary card)		●	●	●	●	●	●	●	●	
Reporting of unsolicited Adverse Events		●	●	●	●	●	●	●	●	
Reporting of convulsions								●		
Reporting of Serious Adverse Events		●	●	●	●	●	●	●	●	●
Study Conclusion										●

Note:
● is used to indicate a study procedure that requires documentation in the individual CRF (eCRF).
O is used to indicate a study procedure that does not require documentation in the individual eCRF.
[1]Concomitant vaccines: in addition to the blinded study vaccines, subjects will be vaccinated with (1) MMR at the age of 12-15 months (according to local EPI), and (2) with HAV vaccine at the age of 12-15 months (visit 5) and at 18-21 months of age (since subjects in the control group receive a dose of HAV vaccine at visit 6, the last HAV dose should be administered at least 28 days after the stuy vaccine booster dose) (study benefit= vaccination against HAV at 12-15 and 18-21 months of age in Panama). (3) In Panama all subjects will be vaccinated against varicella at 12-15 months of age (study benefit= vaccination against varicella in Panama).

Section 5.5.1.1 Scheduled visits to be performed
**Visit 2: at ±4 months of age; Study Month 2**
Study procedure for ALL SUBJECTS
• Check elimination criteria<br>• ***Check exclusion criteria for further study participation***<br>…
**Visit 3: at ±6 months of age; Study Month 4**
Study procedures for ALL SUBJECTS
• Check elimination criteria<br>• ***Check exclusion criteria for further study participation***<br>...

**Visit 4: at 7 months of age; Study Month 5**<br>'IMMUNO AND REACTO' or 'CARRIAGE' SUBSETS ONLY
Study procedures for 'IMMUNO AND REACTO' SUBSET
• Check elimination criteria<br>• ***Check exclusion criteria for further study participation***<br>**…**
Study procedures for 'CARRIAGE' SUBSET
• Check elimination criteria<br>• ***Check exclusion criteria for further study participation***<br>…
**Visit 5: at 12-15 months of age; Study Month 10-13**
Study procedures for ALL SUBJECTS
• Check elimination criteria<br>• ***Check exclusion criteria for further study participation***<br>…
**Visit 6: at 15-18 months of age; Study Month 13-16**
Study procedures for ALL SUBJECTS
• Check elimination criteria<br>• ***Check exclusion criteria for further study participation***<br>…
**Visit 7: at 16-19 months of age; Study Month 14-17**<br>'IMMUNO AND REACTO', 'ADDITIONAL IMMUNO' and 'CARRIAGE' SUBSETS ONLY
Study procedures for 'IMMUNO AND REACTO' SUBSET
• Check elimination criteria<br>• ***Check exclusion criteria for further study participation***<br>…
Study procedures for 'ADDITIONAL IMMUNO' SUBSET
• Check elimination criteria<br>• ***Check exclusion criteria for further study participation***

Visit 8: at 18-21 months of age; Study Month 16-19
Study procedures for ALL SUBJECTS
• Check elimination criteria<br>• ***Check exclusion criteria for further study participation***<br>…

Visit 9: at 24-27 months of age; Study Month 22-25
Study procedures for ALL SUBJECTS
• Check elimination criteria<br>• ***Check exclusion criteria for further study participation***<br>**…**
**Section 5.5.2.1.6. Case accountability**<br>~~***All suspected CAP cases occurring during the entire study must be reported as described in section 5.5.2.1.2.***~~<br>~~For CAP, the start date of a clinical episode corresponds to the date when symptoms first were observed. In case this date is not known or defined, the start date will be the date when the CXR was taken. From an analysis point of view, a new CAP episode will be considered to have started if 30 days or more have elapsed since the start date of the previous CAP episode. If less than 30 days have elapsed since the start date of the previous CAP episode, the new CXR will be considered related to the same CAP episode. If for the same CAP episode, the outcome of consecutive CXR readings evolve over time, the most serious diagnosis will be taken into consideration.~~

**~~Section 5.5.2.2.6 Case accountability~~**<br><br>• ~~All clinical episodes of OM will be reported, including new episodes in a same subject.~~<br><br>• ~~In children with ventilating tubes, tube-related otorrhea is considered as an episode of OM.~~<br><br>• ~~The start date of an episode is the date of visit during which the diagnosis is confirmed by the ENT specialist.~~<br><br>• ~~If an AOM visit occurs within 30 days after the start date of a previous AOM episode, it will be considered as a part of the same clinical episode but, according to the bacterial culture results, as part of the same or as a new bacterial episode:~~<br>– ~~if the latter visit is associated with the same bacterial pathogen and serotype as the one isolated during the previous AOM visit or if no bacterial pathogen could be isolated, the visit will be considered as part of the same bacterial episode~~<br>– ~~if a different bacterial pathogen or a different serotype is isolated during the latter visit than the one isolated during the previous AOM visit, the visit will be considered as the start of a new bacterial episode~~
• ~~From an analysis point of view, if an AOM visit occurs at least 30 days after the date of confirmation by the ENT specialist of a previous AOM episode, it will be considered as a new episode.~~<br><br>• ~~If a mixed flora is identified in the MEF of a child presenting with AOM (e.g., Streptococcus pneumoniae and NTHi, Streptococcus pneumoniae and Moraxella catarrhalis), it will be considered as a single clinical episode, but as separate bacterial episodes (one per isolated bacteria).~~<br><br>• ~~Bilateral AOM with two different bacterial pathogens or serotypes is considered as one clinical episode and one bacterial episode, but as 2 episodes when looking to each bacterial pathogen.~~

Section 5.5.2.3.6 Case accountability

~~From an analysis point of view, if the same pneumococcal or *H. influenzae* serotype is isolated with an interval of less than 30 days, both isolates will be considered related to the same invasive disease episode. If the pneumococcus or *H. influenzae* isolate does not share the same serotype, or if 30 days or more have elapsed since previous isolation, it will be considered as a new invasive disease episode.~~

Section 9.6.2.1 Case accountability

Preliminary remark:the counting rules specified in this section do not modify the reporting procedures for CAP, AOM and ID cases. These counting rules will be applied at the time of the statistical analysis of the study results. Individual episodes should still be reported by the investigator even if according to the counting rule it appears that the case might not be considered to be a new episode.

9.6.2.1.1 Rules for CAP cases

All suspected CAP cases occurring during the entire study must be reported as described in section 5.5.2.1.2.

For CAP, the start date of a clinical episode corresponds to the date when symptoms first were observed. In case this date is not known or defined, the date when the CXR was taken will be considered to be the start date. From an analysis point of view, a new CAP episode will be considered to have started if 30 days or more have elapsed since the start date of the previous CAP episode. If less than 30 days have elapsed since the start date of the previous CAP episode, the new CXR will be considered related to the same CAP episode. If for the same CAP episode, the outcome of consecutive CXR readings evolve over time, the most serious diagnosis will be taken into consideration.consideration.

9.6.2.1.2 Rules for AOM cases

- ***All clinical episodes of OM will be reported, including new episodes in a same subject.***
- ***In children with ventilating tubes, tube-related otorrhea is considered as an episode of OM.***
- ***The start date of an episode is the date of the visit during which the diagnosis is confirmed by the ENT specialist.***

- *If an AOM visit occurs within 30 days after the start date of a previous AOM episode, it will be considered as a part of the same clinical episode but could eventually, according to the bacterial culture results, be considered as part of the same or as a new bacterial episode:*
  - *if the latter visit is associated with the same bacterial pathogen and serotype as the one isolated during the previous AOM visit or if no bacterial pathogen could be isolated, the visit will be considered as part of the same bacterial episode*
  - *if a different bacterial pathogen or a different serotype is isolated during the latter visit than the one isolated during the previous AOM visit, the visit will be considered as the start of a new bacterial episode*
- *From an analysis point of view, if an AOM visit occurs at least 30 days after the date of confirmation by the ENT specialist of a previous AOM episode, it will be considered as a new episode.*
- *If a mixed flora is identified in the MEF of a child presenting with AOM (e.g., Streptococcus pneumoniae and NTHi, Streptococcus pneumoniae and Moraxella catarrhalis, or two different pneumococcal serotypes) it will be considered as a single clinical episode, but as separate bacterial episodes (one per isolated bacteria).*
- *Bilateral AOM with two different bacterial pathogens or serotypes is considered as one clinical episode and one bacterial episode, but as 2 episodes when looking to each bacterial pathogen.*

9.6.2.1.3 Rules for ID cases

From an analysis point of view, if the same pneumococcal or H. influenzae serotype is isolated with an interval of less than 30 days, both isolates will be considered related to the same invasive disease episode. If the pneumococcus or H. influenzae isolate does not share the same serotype, or if 30 days or more have elapsed since previous isolation, it will be considered as a new invasive disease episode.

Section 11 References

American Academy of Pediatrics, Committee on Infectious Diseases. Policy statement: recommendations for the prevention of pneumococcal infections, including the use of pneumococcal conjugate vaccine (Prevnar), pneumococcal polysaccharide vaccine, and antibiotic prophylaxis.Pediatrics 2000; 106 (2); 362-6.

Ministerio de Salud de la Nación del Argentina. Normas Nacionales de Vacunacion, Edición 2008.

GlaxoSmithKline Biologicals

Clinical Research & Development
Protocol Amendment Approval

**eTrack study number**	109563
**eTrack abbreviated title**	10PN-PD-DIT-028
**Protocol title:**	Clinical Otitis Media and Pneumonia Study (**COMPAS**): a phase III , double-blind, randomized, controlled, multicentre study to demonstrate the efficacy of GlaxoSmithKline (GSK) Biologicals' 10-valent pneumococcal conjugate vaccine (GSK1024850A) against Community Acquired Pneumonia (CAP) and Acute Otitis Media (AOM)
**Amendment number:**	Amendment 5
**Amendment date:**	14 December 2009
**Co-ordinating author:**	Mireille Venken, Scientific Writer

Rationale/background for changes: Amendment 5 of the COMPAS protocol was developed for the following reason:

- The number of AOM cases reported to date is much lower than anticipated. To be able to perform the interim analysis to evaluate the efficacy of the 10Pn-PD-DiT vaccine to prevent the first episode of B-CAP regardless of the number of cases of C-AOM that are reached, it was decided to evaluate the vaccine efficacy to prevent the first episode of B-CAP as the only primary objective, and to evaluate the vaccine efficacy to prevent the first episode of C-AOM as a secondary objective instead of a co-primary objective. The number of B-CAP cases needed to perform the interim analysis has been adjusted accordingly.

Amended text has been included in *bold italics* in the following section(s):

Title page

**Contributing authors**	• Juan Pablo Yarzabal MD, MSc, ***Javier Ruiz Guiñazú, MD***, Clinical Development Manager***s***<br>• Leyla Hernandez, Raquel Merino, Kaat Vandorpe, Frederic Henry, Global Study<br>• Isabelle Henckaerts, Scientist, Clinical Immunology<br>• ***Dorota Borys, MD, Director Clinical Development (Pneumococcal vaccines)***<br>• Lode Schuerman MD, ~~Director Clinical Development (Pneumococcal Vaccines)~~ ***Director***, ***Scientific Advisor (Pneumococcal vaccines)***<br>• Patricia Lommel, Biostatistician<br>• William Hausdorff PhD, Director Epidemiology<br>• Ricardo Ruttiman MD,~~ Alejandro Lepetic MD,~~ Clinical Director R&D and Medical Affairs Argentina and Southern Cone

**Synopsis**	
**Objectives**	**Primary**<br><br>To demonstrate the efficacy of a 3-dose primary course followed by a booster dose in the second year of life with the 10Pn-PD-DiT vaccine against likely bacterial CAP cases (B-CAP) in the entire study cohort~~, or against clinically confirmed Acute Otitis Media (C-AOM) cases in the 7,000 children enrolled in Panama~~. Likely bacterial CAP is defined as radiologically confirmed CAP cases with either alveolar consolidation/pleural effusion on the chest X-ray (CXR), or with non-alveolar infiltrates but with CRP $\geq$ 40 mg/L.<br><br>*Criteria for efficacy:*<br><br>• *Efficacy against likely bacterial CAP ~~or clinically confirmed AOM~~ will be demonstrated if the one-sided p-value calculated for the null hypothesis Ho= [B-CAP vaccine efficacy (VE)$\leq$ 0%] ~~or the null hypothesis Ho= [C-AOM VE $\leq$ 0%]~~ is lower than the alpha level defined in Section 9.3.1.1.*<br>**Secondary**<br><br>• ***To demonstrate the efficacy of the 10Pn-PD-DiT vaccine against clinically confirmed Acute Otitis Media (C-AOM) cases in the 7,000 children enrolled in Panama.***<br><br>***Criteria for efficacy:***<br><br>***Efficacy against clinically confirmed AOM will be demonstrated if the one-sided p-value calculated for the null hypothesis Ho= [C-AOM (VE)$\leq$ 0%] is lower than 2.5%.***
**Study design**	• The study will end when the number of subjects with B-CAP ~~and C-AOM~~ required for the final analysis (see Section 9.3.1.1) will be reached, but not before a minimal efficacy follow-up up to 24-27 months of age (visit 9) for each subject was performed.<br><br>• ***At the end of the study, parents/guardians of each subject in Panama will asked if they would be willing to let their child/ward participate in a follow-up study.***

**Primary endpoints**	Occurrence of likely bacterial CAP cases (B-CAP) defined as radiologically confirmed CAP cases with either alveolar consolidation/pleural effusion on the chest X-ray or with non-alveolar infiltrates but with CRP ≥ 40 mg/L.<br>• ~~Occurrence of clinically confirmed AOM cases (C-AOM) *(in all subjects enrolled in Panama)*~~
**Secondary endpoints**	• ***Occurrence of clinically confirmed AOM cases (C-AOM) (in all subjects enrolled in Panama)***

Section 2.1 Primary objective

To demonstrate the efficacy of a 3-dose primary course followed by a booster dose in the second year of life with the 10Pn-PD-DiT vaccine against likely bacterial CAP cases (B-CAP) in the entire study cohort~~, or against clinically confirmed Acute Otitis Media (C-AOM) cases in the 7,000 children enrolled in Panama~~. Likely bacterial CAP is defined as radiologically confirmed CAP cases with either alveolar consolidation/pleural effusion on the chest X-ray (CXR), or with non-alveolar infiltrates but with CRP ≥ 40 mg/L.

Criteria for efficacy:

Efficacy against likely bacterial CAP ~~or clinically confirmed AOM~~ will be demonstrated if the one-sided p-value calculated for the null hypothesis Ho= [B-CAP vaccine efficacy (VE)≤ 0%] ~~or the null hypothesis Ho= [C-AOM VE ≤ 0%]~~ is lower than the alpha level defined in Section 9.3.1.1.

Refer to Section 9.1 for definition of the primary endpoint.

Section 2.2 Secondary objectives

- ***To demonstrate the efficacy of the 10Pn-PD-DiT vaccine against clinically confirmed Acute Otitis Media (C-AOM) cases in the 7,000 children enrolled in Panama.***

  Criteria for efficacy:

  Efficacy against clinically confirmed AOM will be demonstrated if the one-sided p-value calculated for the null hypothesis Ho= [C-AOM (VE)≤ 0%] is lower than 2.5%.

Section 3.1 Study design

- The study will end when the number of subjects with B-CAP ~~and C-AOM~~ required for the final analysis (see Section 9.3.1.1) will be reached, but not before a minimal efficacy follow-up up to 24-27 months of age (visit 9) for each subject was performed**.**
- ***At the end of the study, the investigator will ask the parents/guardians of each subject in Panama if they would be willing to let their child/ward participate in a follow-up study. If the subject's parents/guardians decline to participate in the follow-up study, refusal will be documented in the subject's eCRF.***

Section 6.6 Method of blinding and breaking the study blind

GSK Biologicals ~~Clinical~~ *Central* Safety Physician
(Study Contact for Emergency Code Break)

Tel: ~~+32 2 656 8850~~ ***+32 10 85 48 43***
Fax: +32 2 656 51 16 or +32 2 656 80 09
Mobile phones for 7/7 day availability:
+32 472 906 600
Back-up mobile phone contact:
+32 474 53 48 68

Section 9.1 ~~Co-p~~*Primary endpoint*~~s~~

Occurrence of likely bacterial CAP cases (B-CAP) defined as radiologically confirmed CAP cases with either alveolar consolidation/pleural effusion on the chest X-ray or with non-alveolar infiltrates but with CRP ≥ 40 mg/L.

- ~~Occurrence of clinically confirmed AOM cases (C-AOM) *(in all subjects enrolled in Panama)*~~

Section 9.2 Secondary endpoints

Efficacy:

- ***Occurrence of clinically confirmed AOM cases (C-AOM) (in all subjects enrolled in Panama)***

Section 9.3.1.1.Primary objective (B-CAP ~~or C-AOM~~)

The primary objective is to demonstrate~~:~~ that ~~either~~ the vaccine efficacy (VE), defined as 1-Hazard ratio (HR), induced by the 10Pn-PD-DiT vaccine (three doses in subjects ≤ 18 months of age or four doses in subjects > 18 months of age) in preventing the first episode of likely bacterial CAP (B-CAP) is greater than 0%, as compared to the control group**.**

> ~~OR~~
>
> ~~that the VE induced by the 10Pn-PD-DiT vaccine (three doses in subjects ≤ 18 months of age or four doses in subjects > 18 months of age) in preventing the first episode of clinically confirmed AOM cases (C-AOM) is greater than 0%, as compared to the control group.~~

~~In addition to the possibility to draw independent conclusion on both co-primary endpoints,~~ COMPAS study design would allow early efficacy conclusion if true vaccine efficacy would be in line with our current estimations (25%) while maintaining the possibility to conclude later if the VE would be slightly lower than anticipated (for example, 20%). This leads to the introduction of a planned interim analysis when we will reach a required number of B-CAP ~~and C-AOM~~ cases.

In order to keep the overall one-sided significance level for ~~each co-~~***the*** primary endpoint at ~~1.25%~~ ***2.5%***~~(alpha adjustment for multiplicity on the two co-primary endpoints; i.e. 2.5% / 2~~), the following alpha adjustment based on the Pocock approach will be applied to take into account this interim analysis:

The group receiving the 10Pn-PD-DiT vaccine will be compared to the Control group using a one-sided test at ~~0.90%~~ ***1.75%*** nominal alpha level (at interim analysis) and at ~~0.59%~~ ***1.19%*** nominal alpha level (at final analysis). The success will be established if the one-sided p-value calculated for the null hypothesis H0=[B-CAP VE ≤ 0%] ~~OR H0=[C-AOM VE ≤ 0%]~~ is lower than ***1.75%*** (at interim analysis) and lower than ***1.19%*** (at final analysis) (EAST Survival Module: One sided test~~s~~ with Early rejection of H0 only).

Considering a 1:1 randomization ratio, a true VE against B-CAP ~~and against C-AOM~~ of 20%, a total number of ***1045*** **first B-CAP episodes** (= subjects with evaluable likely bacterial CAP) ~~and of **1200 first C-AOM episodes** (= subjects with evaluable clinically confirmed AOM)~~ in the study will be needed to demonstrate VE > 0% with 90% power at final analysis (PASS 2005 - one-sided test for one proportion, nominal type I error of ~~0.59%~~ ***1.19%***, see details in Appendix J).

The interim analysis will be done when we will reach ~~**628**~~ ***535*** **first B-CAP episodes** ~~and **628 first C-AOM episodes**~~**, with a minimum study duration of 18 months after study start (see Section 9.7).**

Considering the nominal 1-sided alpha level of ~~0.90%~~ ***1.75%*** to be used at the interim analysis, the power to demonstrate VE > 0% at the time of the interim analysis will be ~~64%~~ ***66%*** based on a true VE of 20%, ~~88%~~ ***87%*** based on a true VE of 25% and ~~98%~~ ***97%*** if the true VE is 30% (PASS 2005 - one-sided test for one proportion, nominal type I error of ~~0.90%~~ ***1.75%***, see details in Appendix J).

To be considered as evaluable for primary analysis, an episode must appear in the ATP cohort for efficacy, more than two weeks after the administration of the third dose of study vaccine, and must fulfil the criteria defined in Section 4.5

Based on the incidence rates as shown in Table 22, an enrolment period of 12 months and 10% withdrawal in the ATP cohort for efficacy, a total number of **24,000 subjects** (**12,000 subjects per group**) will need to be enrolled in order to reach ***the initial planned number of first B-CAP episodes of*** at least 1200 ~~first B-CAP episodes~~ approximately 36 months after study start (study end).

~~AOM endpoints will be assessed in all 7,000 subjects enrolled in Panama City (3,500 subjects per group). Considering that at any point in the study, we will have at least as many C-AOM episodes from the 7,000 subjects enrolled in Panama, as we have B-CAP from the total 24,000 subject cohort (Argentina, Colombia and Panama combined), the number of subjects enrolled in Panama will be large enough to also reach at least 1200 first C-AOM episodes at study end. Based on the same incidence rates, we can anticipate to reach the required number of 628 first episodes for the interim analysis approximately 20 months after study start.~~

Section 9.3.1.2. Secondary objective (Clinical AOM - C-AOM) at study end

The first secondary objective is to demonstrate that the VE induced by the 10Pn-PD-DiT vaccine (three doses in subjects ≤18 months of age or four doses in subjects > 18 months of age) in preventing the first episode of clinically confirmed AOM cases (C-AOM) is greater than 0%, as compared to the control group.

Considering a 1:1 randomization ratio and various true C-AOM VE, Table 23 shows the total number of first evaluable C-AOM episodes needed to demonstrate C-AOM VE > 0% with 80 or 90% power (one-sided test, nominal type I error of 2.5%).

Table 23 Total number of first episodes required to show C-AOM VE > 0% with 80% and 90% power, according to various true VE

True VE	*Required first episodes of C-AOM **	
	80% power	*90% power*
10%	*2,891*	*3,845*
15%	*1,226*	*1,637*
20%	*662*	*880*
25%	*405*	*535*
30%	*270*	*350*
35%	*184*	*244*
40%	*134*	*178*
45%	*99*	*132*
50%	*77*	*99*
55%	*61*	*77*

PASS 2005 - one-sided test for one proportion, nominal type I error of 2.5%.

The group receiving the 10Pn-PD-DiT vaccine will be compared to the Control group using a one-sided test at 2.5% nominal alpha level at final analysis. The success will be established if the primary objective has been reached and if the one-sided p-value calculated for the null hypothesis H0=[C-AOM VE ≤ 0%] is lower than 2.5% (at final analysis) (EAST Survival Module: One sided tests with Early rejection of H0 only).

Sections 9.3.1.3, 9.3.1.4 and 9.3.1.5

Footnote Table 24, Table 26 and Table 27

*PASS 2005 - one-sided test for one proportion, nominal type I error of 2.5%~~, %, see details in Appendix J~~

Section 9.3.1.3 Secondary objective (C-CAP) at study end

Footnote Table 24 Total number of first episodes required to show C-CAP VE > 0% with 80% and 90% power, according to various true VE:

*PASS 2005 - one-sided test for one proportion, nominal type I error of 2.5%~~, %, see details in Appendix J~~

Section 9.6.2.2.1. According-to-protocol (ATP) analysis = primary analysis

For the ATP cohort, the first analysis of efficacy will be based on the occurrence of the primary endpoint (i.e., first episodes of likely bacterial CAP ~~and of clinically confirmed AOM~~) anytime from two weeks after the administration of the third dose of the study vaccine.

The VE as compared to the control group will be evaluated using a Cox regression model on the time starting from two weeks after the administration of the third dose of the study vaccine up to the first B-CAP ~~or C-AOM~~ episode. Censoring will occur at the time of the last scheduled and unscheduled contact. For subjects who have more than 18 months of age without having received the booster dose, an episode of B-CAP ~~or C-AOM~~ will be censored at 18 months of age.

VE= (1 – hazard ratio) x 100

All subjects from the ATP cohort will contribute to the comparison.

The Cox model, including the treatment group as the only regressor, will be used to derive 95% CI for the VE.

In order to check the statistical significance, one-sided p-values for the Wald-Test~~s~~ obtained from the Cox proportional hazard model will be calculated. Nominal type I error for the final analysis will be computed according to the final number of first episodes to maintain the ~~1.25%~~ ***2.5%*** type I error ~~for each co-primary endpoint~~. Assuming this is still ~~0.59%~~ ***1.19%,*** the success of the primary objective will be established if the one-sided p-value calculated for the null hypothesis H0=[B-CAP VE ≤ 0%] is lower than ~~0.59%~~ ***1.19%*** ~~OR if the one-sided p-value calculated for the null hypothesis H0=[C-AOM VE ≤ 0%] is lower than 0.59%~~**.**

Note that cumulative hazard curves will be used to display the distribution of time to B-CAP ~~and C-AOM~~ episode, for each group.

Section 9.6.2.2.2 Total vaccinated cohort analysis

For the total cohort, the analysis of efficacy will be based on the occurrence of the primary endpoint (i.e., first episodes of B-CAP ~~or of C-AOM~~) anytime from the time of first vaccination. Subjects without booster dose will not be censored (i.e. all episodes will be included in the analysis).

VE and 95% CI will be computed the same way as for the primary analysis on ATP cohort.

Section 9.6.2.3 Analysis of secondary efficacy objectives

The number of episodes (first and new episodes as defined in Section 5.5.2), follow-up days, and associated rate will be presented by group, for each endpoint.

Based on the first episode of each endpoint, a 95% CI will also be constructed around the VE, as for the primary endpoint~~s~~.

In order to check the statistical significance of the ***first*** secondary objective~~s~~ (***C-AOM***~~, B-AOM, C-CAP and VT-IPD~~), one-sided p-value for the Wald-Tests obtained from the Cox proportional hazard model will be calculated. The success of ~~a~~ ***the first*** secondary objective will be established if the primary objective has been reached and if the one-sided p-value calculated for the null hypothesis H0=[***C-AOM*** VE ≤ 0%] will be lower than 2.5%.

From an exploratory point of view, in order to check the statistical significance of the next secondary objectives (B-AOM, C-CAP and VT-IPD), one-sided p-value for the Wald-Tests obtained from the Cox proportional hazard model will be calculated. The success of a secondary objective will be established if the primary and first secondary objectives has been reached and if the one-sided p-value calculated for the null hypothesis H0=[VE ≤ 0%] will be lower than 2.5%.

Finally, for AOM and CAP endpoints, VE and its 95% CI for the time to occurrence of any episodes (first and new episodes) of a specific event during the considered efficacy follow-up will also be estimated using the Andersen & Gill model (generalization of the Cox proportional hazard model) taking into account all episodes.

Change in VE over time will be investigated using Spline-smoothed estimates of hazard ratio and 95% confidence bands.

In addition, descriptive analysis to evaluate whether vaccine efficacy is influenced by the severity of the disease could be done to complement the primary endpoint results.

Section 9.6.5 Analysis of immunogenicity

'IMMUNO AND REACTO' SUBSET ONLY

As planned on a subset of first enrolled subjects, according to availability of final and complete laboratory results, immunogenicity analysis as described in this section could be done in a stepwise approach before the availability of data related to other endpoints (efficacy, safety etc.). In order to keep the blinding regarding efficacy endpoints, any analysis of immunogenicity before final analysis will be performed by an independent statistician.

Section 9.7. Planned interim analysis

An interim efficacy analysis will be performed to evaluate the efficacy to prevent the first episode of B-CAP ~~and C-AOM~~ (~~co-~~primary endpoint~~s~~). The interim analysis will be used to determine whether positive VE can be detected earlier than anticipated based on a higher VE (interim analysis for early final conclusion).

The interim analysis will be performed when we will reach at least **~~628~~** ***535*** **first B-CAP episodes** ~~and at least **628 first C-AOM episodes**~~ and will be performed **for subjects in the ATP cohort.** In addition, a minimum study duration of 18 months after study start will be respected in order to allow a clinically meaningful duration of follow-up in a sufficient number of subjects

The interim analysis to evaluate the efficacy to prevent the first episode of B-CAP ~~or the first episode of C-AOM~~ will be analyzed according to the methodology described in Section 9.6.2.2. The interim analysis will be performed by a statistician independent of the project and GSK Biologicals (i.e. from a contract research organization). Project staff within GSK Biologicals and investigator will remain blinded to the randomization codes until final analysis.

In order to check the statistical significance at the time of this interim analysis, one-sided p-values for the Wald-Tests obtained from the Cox proportional hazard model will be calculated. The success of the primary objective will be established if the one-sided nominal p-value calculated for the null hypothesis H0=[B-CAP VE ≤ 0%] is lower than ~~0.90%~~ ***1.75%*** ~~OR if the one-sided p-value calculated for the null hypothesis H0=[C-AOM VE ≤ 0%] is lower than 0.90%.~~

The results of the interim analysis will be sent to the IDMC. The IDMC will review the results and will communicate to GSK ~~which of~~ ***if*** the primary endpoint~~s~~ ~~have~~ ***has*** been met. Upon request by GSK, this conclusive results (nominal p-value < ~~0.90%~~ ***1.75%***) will be shared by IDMC. ***In case the primary endpoint has been met, GSK might request to analyse secondary endpoints related to CAP. This analysis will also be performed by a statistician independent of the project and GSK Biologicals. The results will be sent to the IDMC and communicated to GSK.***

Appendix J Mathematical details about sample size determination

<u>2. Null hypothesis expressed in terms of a proportion and derived sample size</u>

…

Thus, in terms of p, the null hypothesis can be written as

$$\mathbf{H_O: p \geq 0.5}$$

This reformulated hypothesis was used to derive the sample size.

A total number of ~~1200~~ ***1045*** subjects with evaluable episodes will provide at least 90% power to demonstrate that VE is at least equal to 0%, assuming that the true VE is equal to 20% or that p is equal to 0.444 (PASS 2005 – One Proportion Power Analysis, one-sided test, nominal type I error of ~~0.90%~~ ***1.19%***).

GlaxoSmithKline Biologicals

Clinical Research & Development

Protocol Amendment Approval

**eTrack study number**	109563
**eTrack abbreviated title**	10PN-PD-DIT-028
**Protocol title:**	Clinical Otitis Media and Pneumonia Study (**COMPAS**): a phase III , double-blind, randomized, controlled, multicentre study to demonstrate the efficacy of GlaxoSmithKline (GSK) Biologicals' 10-valent pneumococcal conjugate vaccine (GSK1024850A) against Community Acquired Pneumonia (CAP) and Acute Otitis Media (AOM)
**Amendment number:**	Amendment 6
**Amendment date:**	09 September2010
**Co-ordinating author:**	Mireille Venken, Scientific Writer

Rationale/background for changes: Amendment 6 of the COMPAS protocol was developed for the following reason:

As the observed incidence of B-CAP has been lower than the expected incidence, the number of cases needed to perform the final analysis would not be reached in the near future and might never be reached based on extrapolation of the accrual of B-CAP cases. As this study has been designed to determine vaccine efficacy pneumonia, which is a major burden in Latin America and the rest of the world, results should be reported in a timely manner. Therefore the Company has decided to amend the protocol to re-define study end based on the outcome of the planned interim analysis

In addition, clarifications to the objectives and endpoints have been implemented. Finally, wording in Appendix I was corrected to align with the change in the primary objective implemented in previous amendment (amendment 5, dated 14 December 2009).

The contact details for the emergency code break have been clarified. As of now new phone numbers have to be used for the safety contact for code break (emergency unblinding).

Amended text has been included in *bold italics* and deleted text in ~~strikethrough~~ in the following section(s):

Title page

**Contributing authors**	• Juan Pablo Yarzabal MD, MSc, Javier Ruiz Guiñazú, MD, Clinical Development Managers<br>• Leyla Hernandez, Raquel Merino, Kaat Vandorpe, Frederic Henry, ***Mercedes Lojo-Suarez***,Global Study Managers<br>• Isabelle Henckaerts, Scientist, Clinical Immunology<br>• ***Sonia Schoonbroodt**, **Expert Scientist, Clinical***

	Immunology<br>• Dorota Borys, MD, Director Clinical Development (Pneumococcal vaccines)<br>• Lode Schuerman MD, Director, Scientific Advisor (Pneumococcal vaccines)<br>• Patricia Lommel, Biostatistician<br>• William Hausdorff PhD, Director Epidemiology<br>• Ricardo Ruttiman MD, Clinical Director R&D and Medical Affairs Argentina and Southern Cone

Synopsis: Objectives

Secondary

- ~~To assess the efficacy of the 10Pn-PD-DiT vaccine in preventing bacteriologically confirmed AOM cases (B-AOM) caused by *Haemophilus influenzae* and vaccine serotypes, cross-reactive and other *Streptococcus pneumoniae* (in the 7,000 subjects enrolled in Panama)~~
- ***To assess the efficacy of the 10Pn-PD-DiT vaccine in preventing bacteriologically confirmed AOM cases (B-AOM) caused by any bacterial pathogen (in the 7,000 subjects enrolled in Panama)***
- ***To assess the efficacy of the 10Pn-PD-DiT vaccine in preventing bacteriologically confirmed AOM cases (B-AOM) caused by vaccine serotypes, cross-reactive and other Streptococcus pneumoniae serotypes (in the 7,000 subjects enrolled in Panama)***
- ***To assess the efficacy of the 10Pn-PD-DiT vaccine in preventing bacteriologically confirmed AOM cases (B-AOM) caused by Haemophilus influenzae (in the 7,000 subjects enrolled in Panama)***
- ***To assess the efficacy of the 10Pn-PD-DiT vaccine in preventing bacteriologically confirmed AOM cases (B-AOM) caused by non-typeable Haemophilus influenzae (in the 7,000 subjects enrolled in Panama)***
- ***To assess the efficacy of the 10Pn-PD-DiT vaccine in preventing bacteriologically confirmed AOM cases (B-AOM) caused by other AOM pathogens (for example Moraxella catarrhalis, Group A streptococci and Staphylococcus aureus) (in the 7,000 subjects enrolled in Panama)***
- To document the impact of the 10Pn-PD-DiT vaccine against ~~confirmed~~ CAP ***with alveolar consolidation or pleural effusion on chest X-ray (C-CAP)*** cases ~~with respiratory tract infections~~ ***positive respiratory viral test***
- ***To document the impact of the 10Pn-PD-DiT vaccine against CAP cases with any abnormal CXR with positive respiratory viral test***
- To document the impact of the 10Pn-PD-DiT vaccine against likely bacterial CAP (B-CAP) cases with positive respiratory viral ~~diagnostic~~ test

**Study design**	• ~~The study will end when the number of subjects with B-CAP required for the final analysis (see Section 9.3.1.1) will be reached, but not before a minimal efficacy follow-up up to 24-27 months of age (visit 9) for each subject was performed.~~<br>• ***The study end will depend on the outcome of the planned B-CAP interim analysis. If the outcome on the primary endpoint is conclusive, the study end (completion of contact 10 for each subject) will be organized as soon as possible. If the outcome is not conclusive, the study end will be organized approximately between September and December 2011.***<br>• ~~At the end of the study, the investigator will ask the parents/guardians of each subject in Panama if they would be willing to let their child/ward participate in a follow-up study. If the subject's parents/guardians decline to participate in the follow-up study, refusal will be documented in the subject's eCRF.~~
**Secondary endpoints**	***Efficacy:***<br>• ~~Occurrence of bacteriologically confirmed AOM cases (B-AOM) caused by vaccine serotypes, cross-reactive and other pneumococcal serotypes or by *H. influenzae (in all subjects enrolled in Panama)*~~<br>• ***Occurrence of bacteriologically confirmed AOM cases (B-AOM) caused by any bacterial pathogen (in all subjects enrolled in Panama)***<br>• ***Occurrence of bacteriologically confirmed AOM cases (B-AOM) caused by vaccine serotypes, cross-reactive and other pneumococcal serotypes (in all subjects enrolled in Panama)***<br>• ***Occurrence of bacteriologically confirmed AOM cases (B-AOM) caused by H. influenzae (in all subjects enrolled in Panama)***<br>• ***Occurrence of bacteriologically confirmed AOM cases (B-AOM) caused by non-typeable H. influenzae (in all subjects enrolled in Panama)***<br>• ***Occurrence of bacteriologically confirmed AOM cases (B-AOM) caused by other AOM pathogens (for example Moraxella catarrhalis, Group A streptococci and Staphylococcus aureus) (in all subjects enrolled in Panama)***

	• Occurrence of ~~confirmed~~ CAP cases ***with alveolar consolidation or pleural effusion on the Chest X-ray (C-CAP)*** ~~associated with respiratory viral infection~~ ***with positive respiratory viral test***<br>• ***Occurrence of CAP cases with any abnormal CXR with positive respiratory viral test***<br>***Occurrence of likely bacterial CAP (B-CAP) cases with positive respiratory viral test***

Section 2.2 Secondary objectives

- ~~To assess the efficacy of the 10Pn-PD-DiT vaccine in preventing bacteriologically confirmed AOM cases (B-AOM) caused by *Haemophilus influenzae* and vaccine serotypes, cross-reactive and other *Streptococcus pneumoniae* (in the 7,000 subjects enrolled in Panama)~~
- ***To assess the efficacy of the 10Pn-PD-DiT vaccine in preventing bacteriologically confirmed AOM cases (B-AOM) caused by any bacterial pathogen (in the 7,000 subjects enrolled in Panama)***
- ***To assess the efficacy of the 10Pn-PD-DiT vaccine in preventing bacteriologically confirmed AOM cases (B-AOM) caused by vaccine serotypes, cross-reactive and other Streptococcus pneumoniae serotypes (in the 7,000 subjects enrolled in Panama)***
- ***To assess the efficacy of the 10Pn-PD-DiT vaccine in preventing bacteriologically confirmed AOM cases (B-AOM) caused by Haemophilus influenzae (in the 7,000 subjects enrolled in Panama)***
- ***To assess the efficacy of the 10Pn-PD-DiT vaccine in preventing bacteriologically confirmed AOM cases (B-AOM) caused by non-typeable Haemophilus influenzae (in the 7,000 subjects enrolled in Panama)***
- ***To assess the efficacy of the 10Pn-PD-DiT vaccine in preventing bacteriologically confirmed AOM cases (B-AOM) caused by other AOM pathogens (for example Moraxella catarrhalis, Group A streptococci and Staphylococcus aureus) (in the 7,000 subjects enrolled in Panama)***
- To document the impact of the 10Pn-PD-DiT vaccine against ~~confirmed~~ CAP cases ***with alveolar consolidation or pleural effusion on chest X-ray (C-CAP)*** with ~~respiratory tract infections~~ ***positive respiratory viral test***
- ***To document the impact of the 10Pn-PD-DiT vaccine against CAP cases with any abnormal CXR with positive respiratory viral test***
- To document the impact of the 10Pn-PD-DiT vaccine against likely bacterial CAP (B-CAP) cases with positive respiratory viral ~~diagnostic~~ test

Section 3.1 Study design

- The study is designed in order to reach the ~~628~~ 535 B-CAP cases ~~and the 628 C-AOM cases~~ required for the interim analysis (see Section 9.3.1.1) approximately 24 months after study start. The evaluation of ~~these~~ ***this*** primary endpoint~~s~~ can however be delayed in case accumulation of cases is slower than expected. ~~If accumulation of cases is faster, the analysis of one or both of the primary endpoints can be done earlier, but a minimum study duration of 18 months after study start will be respected in order to allow a clinically meaningful duration of follow-up in a sufficient number of subjects. Efficacy follow-up will continue for all subjects until study end.~~
- ~~The study will end when the number of subjects with B-CAP required for the final analysis (see Section 9.3.1.1) will be reached, but not before a minimal efficacy follow-up up to 24-27 months of age (visit 9) for each subject was performed.~~
- ***The study end will depend on the outcome of the planned B-CAP interim analysis. If the outcome on the primary endpoint is conclusive, the study end (completion of contact 10 for each subject) will be organized as soon as possible. If the outcome is not conclusive, the study end will be organized approximately between September and December 2011.***
- ~~At the end of the study, the investigator will ask the parents/guardians of each subject in Panama if they would be willing to let their child/ward participate in a follow-up study. If the subject's parents/guardians decline to participate in the follow-up study, refusal will be documented in the subject's eCRF.~~

Section 5.6.3 *Microbiological assessments*
For each subject, evidence and identification of Haemophilus influenzae, Streptococcus pneumoniae and other bacterial pathogens (for example Moraxella catarrhalis, Group A streptococci and Staphylococcus aureus) in nasopharyngeal specimens will be investigated using bacteriological culture and other bacteriological procedures as described in the COMPAS Lab Workbook at the local bacteriological laboratory.

Identified S. pneumoniae and H. influenzae will undergo further testing for identification of serotypes at the Central Laboratory, GSK Biologicals laboratory or other laboratory designated by GSK Biologicals.

H. influenzae isolates will undergo further subtyping for discrimination of Haemophilus haemolyticus species using new molecular biology tools at GSK Biologicals' laboratory or a validated laboratory designated by GSK Biologicals using standardized and qualified/validated procedures.

Section 6.6 Method of blinding and breaking the study blind

This study will be conducted in a double-blind/observer-blind fashion. Although the 10Pn-PD-DiT, the HBV and the HAV vaccines are whitish liquid in prefilled syringes, *Havrix* and *Engerix-B* have a slightly different visual appearance than 10Pn-PD-DiT. Therefore, in this trial, an observer-blind procedure will be followed to ensure the double-blinding of the study: the person responsible for the preparation and

administration of the vaccine will not be involved in the efficacy or safety/reactogenicity evaluation of the vaccine.

In order to keep the blinding, any analysis conducted before all study data are available and cleaned will be performed by an independent statistician (see section 9.7). In addition ~~The serological and efficacy~~ data, which might lead to the unblinding of the treatment groups, will not be available during the course of the trial to any investigator or any person involved in the clinical conduct of the study (including data cleaning).

GSK Biologicals Central Safety Physician
(Study Contact for Emergency Code Break)

Phones for 7/7 day availability

Tel: ~~+32 10 85 48 43~~ ***+32 10 85 64 00***
~~Fax: +32 2 656 51 16 or +32 2 656 80 09~~
~~Mobile phones for 7/7 day availability:~~
~~+32 472 906 600~~
Back-up ~~mobile~~ phone contact:
~~+32 474 53 48 68~~ ***+32 10 85 6401***

Section 9.2 Secondary endpoints
Efficacy:

- ~~Occurrence of bacteriologically confirmed AOM cases (B-AOM) caused by vaccine serotypes, cross-reactive and other pneumococcal serotypes or by *H. influenzae (in all subjects enrolled in Panama)*~~
- ***Occurrence of bacteriologically confirmed AOM cases (B-AOM) caused by any bacterial pathogen (in all subjects enrolled in Panama)***
- ***Occurrence of bacteriologically confirmed AOM cases (B-AOM) caused by vaccine serotypes, cross-reactive and other pneumococcal serotypes (in all subjects enrolled in Panama)***
- ***Occurrence of bacteriologically confirmed AOM cases (B-AOM) caused by H. influenzae (in all subjects enrolled in Panama)***
- ***Occurrence of bacteriologically confirmed AOM cases (B-AOM) caused by non-typeable H. influenzae (in all subjects enrolled in Panama)***
- ***Occurrence of bacteriologically confirmed AOM cases (B-AOM) caused by other AOM pathogens (for example Moraxella catarrhalis, Group A streptococci and Staphylococcus aureus) (in all subjects enrolled in Panama)***
- Occurrence of ~~confirmed~~ CAP cases ***with alveolar consolidation or pleural effusion on the Chest X-ray (C-CAP)*** ~~associated with respiratory viral infection~~ ***with positive respiratory viral test***
- ***Occurrence of CAP cases with any abnormal CXR with positive respiratory viral test***
- ***Occurrence of likely bacterial CAP (B-CAP) cases with with positive respiratory viral test***

Section 9.3.1.1 Primary objective (B-CAP ~~or C-AOM~~)

The primary objective is to demonstrate that the vaccine efficacy (VE), defined as 1-Hazard ratio (HR), induced by the 10Pn-PD-DiT vaccine (three doses in subjects ≤ 18 months of age or four doses in subjects > 18 months of age) in preventing the first episode of likely bacterial CAP (B-CAP) is greater than 0%, as compared to the control group.

COMPAS study design would allow early efficacy conclusion if true vaccine efficacy would be in line with our current estimation (25%) while maintaining the possibility to conclude later if the VE would be slightly lower than anticipated (for example, 20%). This leads to the introduction of a planned interim analysis when we will reach a required number of B-CAP cases.

In order to keep the overall one-sided significance level for the primary endpoint at 2.5%, the following alpha adjustment based on the Pocock approach will be applied to take into account this interim analysis:

When the number of first B-CAP from 14 days post dose 3 reported in the ATP cohort reaches 535, the ~~The~~ group receiving the 10Pn-PD-DiT vaccine will be compared to the Control group using a one-sided test at 1.75% nominal alpha level ~~(at~~

~~interim analysis)~~ ***i.e.*** ~~and at 1.19% nominal alpha level (at final analysis). The success will be established~~ ***the interim analysis will be considered conclusive*** if the one-sided p-value calculated for the null hypothesis H0=[B-CAP VE ≤ 0%] is lower than 1.75% ~~(at interim analysis) and lower than 1.19% (at final analysis) (EAST Survival Module: One sided test with Early rejection of H0 only)~~.

If the interim analysis is not conclusive the study will continue and study end will be organized approximately between September and December 2011. The final analysis will then take place and the group receiving the 10Pn-PD-DiT vaccine will be compared to the Control group using a one-sided test and an adjusted nominal alpha level. The nominal alpha level will be adjusted according to the number of B-CAP at interim analysis and at the final analysis to ensure the total type I error is maintained to 2.5%.

Table 22 provides the power for B-CAP efficacy at the interim analysis and at the final analysis according to various VE assumptions and first B-CAP cases at final analysis. If the true VE is 25%, the study has 87.6% power to be conclusive at the interim analysis. If the interim is not conclusive, Figure 8 provides projection on the number of evaluable B-CAP expected at study end (=end 2011) and shows that one can consider that the total number of evaluable B-CAP cases at the final analysis will be between 600 and 700.

Therefore if the true VE is 20% and the interim analysis is not conclusive despite a 66% power at the interim there is at least 9.7% chance to conclude at the final analysis with a global power of at least 75.7%

Table 22 Power for B-CAP efficacy according to the number of events at interim and final analyses

Number of events at final analysis	*1-sided alpha at final analysis**	*True VE*	*Power at Interim*	*Power at final***	*Global Power*
600	*2.024016%*	*20.0%<br>22.5%<br>25.0%*	*66.0%<br>78.1%<br>87.6%*	*9.7%<br>7.9%<br>5.4%*	*75.7%<br>86.0%<br>93.0%*
625	*1.894783%*	*20.0%<br>22.5%<br>25.0%*	*66.0%<br>78.1%<br>87.6%*	*11.6%<br>9.4%<br>6.4%*	*77.6%<br>87.5%<br>91.0%*
650	*1.790330%*	*20.0%<br>22.5%<br>25.0%*	*66.0%<br>78.1%<br>87.5%*	*11.6%<br>9.5%<br>6.6%*	*77.6%<br>87.6%<br>94.1%*
675	*1.704119%*	*20.0%<br>22.5%<br>25.0%*	*66.0%<br>78.1%<br>87.6%*	*12.2%<br>10.7%<br>7.3%*	*79.2%<br>88.8%<br>94.9%*
700	*1.631312%*	*20.0%<br>22.5%<br>25.0%*	*66.0%<br>78.1%<br>87.6%*	*14.7%<br>11.8%<br>8.0%*	*80.7%<br>89.9%<br>95.5%*

** nominal alpha at final analysis is adjusted to reach 2.5% global alpha considering an interim analysis with 535 events and an interim nominal 1-sided alpha of 1.75% (EAST, 1-sided test for a binomial distribution, see Appendix J on how testing VE can be expressed as testing one proportion)*
*** probability to succeed at the final analysis and to fail at the interim analysis*

Figure 7 Projection of the number of first B-CAP episodes from 2 weeks after dose 3 (Total vaccinated cohort) (10% drop-out /year)

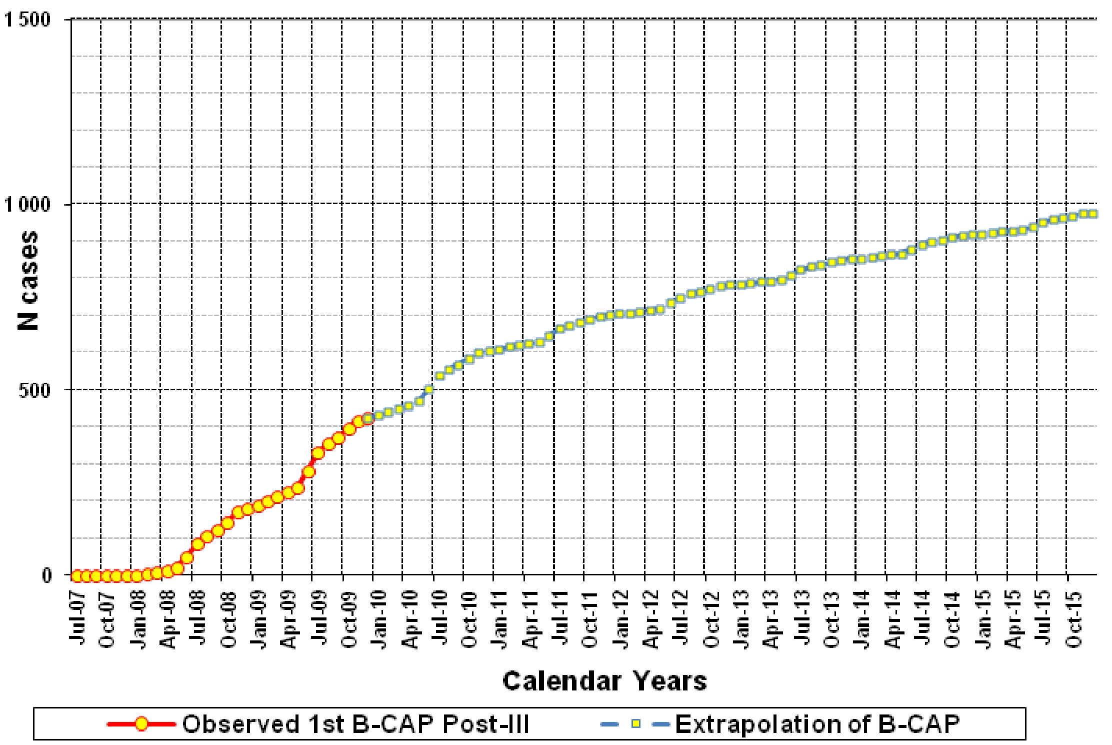

Observed 1st B-CAP Post-dose III = number of observed 1st B-CAP cases up to December 2009.
Extrapolation of B-CAP = extrapolation beyond December 2009 assuming no limit in study duration but taking into account an expected drop of 50% in incidence of B-CAP (compared to the observed incidence in the second year of life) when children reach 2 years of age.

~~Considering a 1:1 randomization ratio, a true VE against B-CAP of 20% a total number of **1045 first B-CAP episodes** (= subjects with evaluable likely bacterial CAP) in the study will be needed to demonstrate VE > 0% with 90% power at final analysis (PASS 2005 - one-sided test for one proportion, nominal type I error of 1.19%, see details in Appendix J).~~

~~The interim analysis will be done when we will reach **535 first B-CAP episodes, with a minimum study duration of 18 months after study start (see Section 9.7).**~~

~~Considering the nominal 1-sided alpha level of 1.75% to be used at the interim analysis, the power to demonstrate VE > 0% at the time of the interim analysis will be 66% based on a true VE of 20%, 87% based on a true VE of 25% and 97% if the true VE is 30% (PASS 2005 - one-sided test for one proportion, nominal type I error of 1.75%, see details in Appendix J).~~

~~To be considered as evaluable for primary analysis, an episode must appear in the ATP cohort for efficacy, more than two weeks after the administration of the third dose of study vaccine, and must fulfil the criteria defined in Section 4.5~~

~~Based on the incidence rates as shown in Table 23, an enrolment period of 12 months~~

~~and 10% withdrawal in the ATP cohort for efficacy, a total number of **24,000 subjects (12,000 subjects per group)** will need to be enrolled in order to reach the initial planned number of first B-CAP episodes of at least 1200 approximately 36 months after study start (study end).~~

~~Table 23 Likely Bacterial CAP incidences~~

~~Endpoint~~	~~1-5 months of age~~	~~6-11 months of age~~	~~12-23 months of age~~	~~24-35 months of age~~
~~B-CAP* (/100,000 child months)~~	~~345.35~~	~~329.40~~	~~245.40~~	~~179.56~~

~~Figure 8 shows the estimated evolution over time of the number of B-CAP cases occurring during the entire study period.~~

~~Figure 8 Estimated evolution of the number of B-CAP cases~~

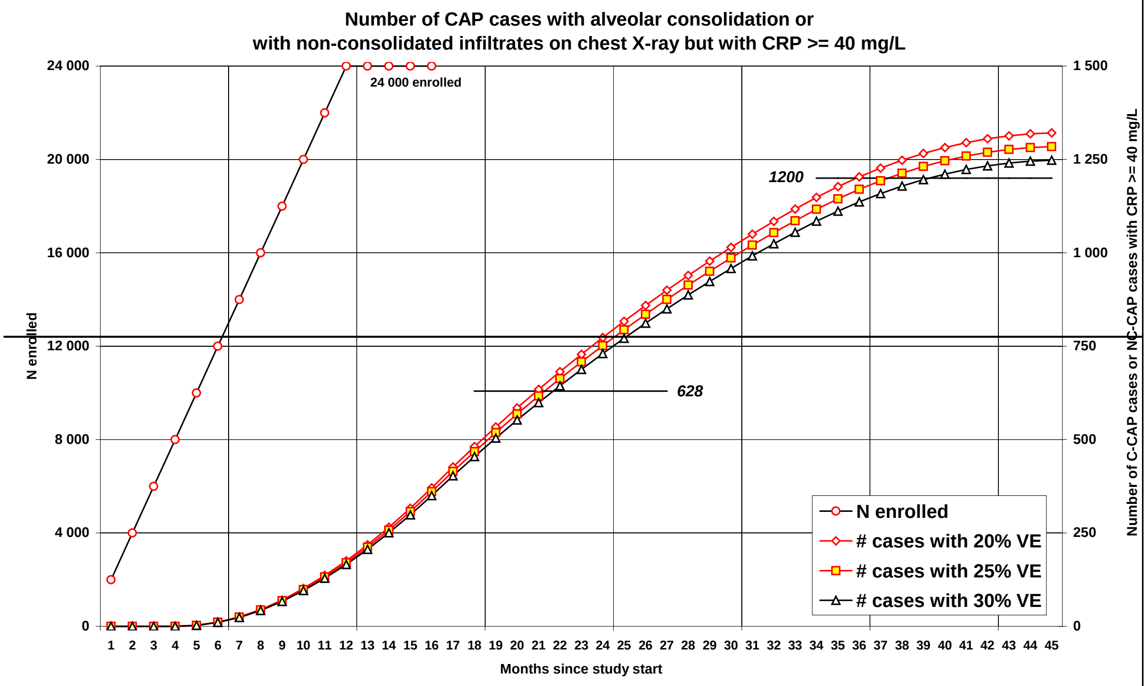

~~Based on a 1:1 randomization ratio, 12 months enrollment, 24,000 enrolled subjects and 10% withdrawal, an estimated true VE against B-CAP of 20%, LL of 95%CI > 0%, power = 90%, ATP efficacy follow-up starting 2 weeks after the third vaccine dose, baseline incidence estimates according to the average of the incidence rates of Santiago de Chile (Jan-2004 to Dec-2005, INE) (see Table 23)~~

Section 9.6.2.2.1 According-to-protocol (ATP) analysis = primary analysis
In order to check the statistical significance, one-sided p-values for the Wald-Test obtained from the Cox proportional hazard model will be calculated. ***Nominal type I error for the interim analysis will be 1.75%.*** Nominal type I error for the final analysis will be computed according to the final number of first episodes to maintain the 2.5% type I error. ~~Assuming this is still 1.19%, the success of the primary objective will be~~

~~established if the one-sided p-value calculated for the null hypothesis H0=[B-CAP VE ≤ 0%] is lower than 1.19%.~~

Section 9.6 Final analysis (at study end: see definition in Section3.1)
In order to keep the blinding, any analysis of immunogenicity or carriage before the final analysis will be performed by an independent statistician.

For sequence of analysis refer to section 9.7.1.

Section 9.6.2.3. Analysis of secondary efficacy objectives
In order to check the statistical significance of the first secondary objective (C-AOM) one-sided p-value for the Wald-Tests obtained from the Cox proportional hazard model will be calculated. The success of the first secondary objective will be established if the primary objective has been reached and if the one-sided p-value calculated for the null hypothesis H0=[C-AOM VE ≤ 0%] will be lower than 2.5%.

From an exploratory point of view, in order to check the statistical significance of the next secondary objectives (B-AOM, C-CAP and VT-IPD), one-sided p-value for the Wald-Tests obtained from the Cox proportional hazard model will be calculated. ***Next secondary objectives will be reached*** ~~The success of a secondary objective will be established~~ if the primary and first secondary objectives ~~has~~ ***have*** been reached and if the one-sided p-value calculated for the null hypothesis H0=[VE ≤ 0%] will be lower than 2.5%.

…
The vaccine-attributable reduction (VAR) per 1000 child-years, defined as the incidence in the control group minus the incidence in the 10Pn group will also be computed with its 95% CI.

Section 9.6.3 Analysis of carriage

‘CARRIAGE’ SUBSET ONLY

As planned on a second subset of enrolled subjects, according to availability of final and complete laboratory results, carriage analysis as described in this section could be done in a stepwise approach before the availability of final data related to other endpoints (efficacy, safety, etc.). In order to keep the blinding, any analysis of carriage before final analysis will be performed by an independent statistician.

Section 9.6.5 Analysis of immunogenicity

‘IMMUNO AND REACTO’ SUBSET ONLY

As planned on a subset of first enrolled subjects, according to availability of final and complete laboratory results, immunogenicity analysis as described in this section could be done in a stepwise approach before the availability of data related to other endpoints (efficacy, safety etc.). In order to keep the blinding ~~regarding efficacy endpoints~~, any analysis of immunogenicity before final analysis will be performed by an independent statistician.

Section 9.7 Planned interim analysis
Section 9.7.1 Sequence of analysis
B-CAP interim analysis will be conducted as described in section 9.7.2.

If this interim analysis is conclusive (=primary objective is met):

- ***Additional analyses will be performed by the Independent Statistician before study end in a stepwise approach according to availability of required data:***
  - ***ATP vaccine efficacy analysis on defined secondary endpoints related to CAP.***
  - ***Partial analysis of demography characteristics (age, gender and race).***
  - ***Final analysis of immunogenicity as described in section 9.6.5.***
  - ***Final analysis of carriage as described in section 9.6.3.***
- ***Remaining analyses will be prepared by GSK at study end on cleaned and final data, including:***
  - ***Complementary analysis of CAP including the additional CAP reported from the interim analysis to study end.***
  - ***Final analysis of AOM and ID.***
  - ***Final analysis of safety.***
  - ***Final analysis of antibiotic treatment.***

If this interim analysis is NOT conclusive:

- ***The study end for a subject will be organized approximately between September and December 2011. All the analyses described in section 9.6 will be generated on cleaned and final data.***

Section 9.7.2 Consideration for the interim analysis
The results of the interim analysis will be sent to the IDMC. The IDMC will review the results and will communicate to GSK if the primary endpoint has been met. Upon request by GSK, this conclusive result (nominal p-value < 1.75%) will be shared by IDMC. In case the primary endpoint has been met, GSK might request to analyse secondary endpoints related to CAP ***and other endpoints as described in the section 9.2***. This analysis will also be performed by a statistician independent of the project and GSK Biologicals. The results will be sent to the IDMC and communicated to GSK.

Appendix I AOM severity scales used in COMPAS

For this reason, the COMPAS study has as ***first secondary*** ~~a primary~~ objective to demonstrate vaccine efficacy against clinically confirmed AOM caused by vaccine serotype pneumococci and *H. influenzae*.

Appendix J Mathematical details about sample size determination

2. Null hypothesis expressed in terms of a proportion and derived sample size

A total number of ***535*** ~~1045~~ subjects with evaluable episodes will provide ~~at least~~ 90% power to demonstrate that VE is at least equal to 0%, assuming that the true VE is equal to ***25%*** ~~20%~~ or that p is equal to ***0.4286*** ~~0.444~~ (PASS 2005 – One Proportion Power Analysis, one-sided test, nominal type I error of ***2.5%*** ~~1.19%~~).

GlaxoSmithKline Biologicals

Clinical Research & Development
Protocol Amendment Approval

**eTrack study number**	109563
**eTrack abbreviated title**	10PN-PD-DIT-028
**Protocol title:**	Clinical Otitis Media and Pneumonia Study (**COMPAS**): a phase III , double-blind, randomized, controlled, multicentre study to demonstrate the efficacy of GlaxoSmithKline (GSK) Biologicals' 10-valent pneumococcal conjugate vaccine (GSK1024850A) against Community Acquired Pneumonia (CAP) and Acute Otitis Media (AOM)
**Amendment number:**	Amendment 6
**Amendment date:**	09 September2010

Approved by:

_________________________ _____________
Javier Ruiz Guiñazú MD, MSc dd-mm-yyyy

Clinical Development Manager
(Pneumococcal vaccines)

GlaxoSmithKline Biologicals

Clinical Research & Development
Protocol Amendment Approval

**eTrack study number**	109563
**eTrack abbreviated title**	10PN-PD-DIT-028
**Protocol title:**	Clinical Otitis Media and Pneumonia Study (**COMPAS**): a phase III , double-blind, randomized, controlled, multicentre study to demonstrate the efficacy of GlaxoSmithKline (GSK) Biologicals' 10-valent pneumococcal conjugate vaccine (GSK1024850A) against Community Acquired Pneumonia (CAP) and Acute Otitis Media (AOM)
**Amendment number:**	Amendment 6
**Amendment date:**	09 September2010
**Agreed by:**<br>**Investigator:**<br><br>**Investigator signature:**<br><br><br>**Date:**	 <br><br><br>________________________________<br><br>dd-mm-yyyy<br>________________________________
