# Supplementary material for: Efficacy of Pneumococcal Nontypable Haemophilus influenzae Protein D Conjugate Vaccine (PHiD-CV) in Young Latin American Children: A Double-Blind Randomized Controlled Trial
Source: PLoS Med. 2014 Jun 3;11(6):e1001657. doi: 10.1371/journal.pmed.1001657 (PMC4043495; doi:10.1371/journal.pmed.1001657)
Supplement: Text S3 — Major protocol changes. (DOCX) [file pmed.1001657.s014.docx]

**Text S3 Major protocol changes**

In protocol amendment 1, dated November 26, 2007, the following changes were implemented:

- Study centers in Colombia were added.
- A new subset of children in Panama was added for further exploration after study end of the correlation between protection against acute otitis media (AOM) episodes caused by (nontypable) *Haemophilus influenzae* and results of the measurement and characterization of responses induced by the protein D carrier protein.
- The Reference Laboratory was changed from Instituto Malbrán in Argentina to Eurofins in the USA, which implied adaptations to the laboratory procedures.

In protocol amendment 2, dated March 20, 2008, the following changes were implemented:

- In Colombia, two doses of human rotavirus vaccine (Rotarix) would be offered to all children within the first six months of life to provide additional benefit.
- The definition of preterm infants was clarified.
- In case children would by mistake receive a vaccine with antigens common to the antigens contained in the study or co-administered vaccines outside of the context of the study, the investigator would need to evaluate whether the child could still continue participation in the study. Therefore this criterion was added to the exclusion criteria for further study participation.

Protocol amendment 3, dated November 25, 2008, was developed in reply to questions from local health authorities:

- Children diagnosed to be at high risk for invasive pneumococcal disease (IPD) were excluded from the study in case a specific local vaccination program was available (further clarification was provided in protocol amendment 4).
- The informed consent process for minor parents (<18 years of age) was specified.
- Additional minor changes and clarifications were implemented.

In addition, GlaxoSmithKline decided to extend the recruitment period to 18 months.

Protocol amendment 4, dated May 11, 2009, was developed on request of local health authorities:

In the exclusion criteria for further study participation the criteria for high risk for pneumococcal disease were specified and clarification on how to proceed in case of diagnosis of a high risk condition for pneumococcal infection was added (see main text).

Protocol amendment 5, dated December 14, 2009, was developed for the following reason:

The number of AOM cases reported to that date was much lower than anticipated. To be able to perform the interim analysis to evaluate the efficacy of the PHiD-CV vaccine to prevent the first episode of likely bacterial community-acquired pneumonia (B-CAP) regardless of the number of cases of clinically-confirmed AOM (C-AOM) that would be reached, it was decided to evaluate the vaccine efficacy to prevent the first episode of B-CAP as the only primary objective, and to evaluate the vaccine efficacy to prevent the first episode of C-AOM as a secondary objective instead of a co-primary objective. The number of B-CAP cases needed to perform the interim analysis was adjusted accordingly.

Protocol amendment 6, dated September 9, 2010, was developed for the following reason:

As the observed incidence of B-CAP was lower than the expected incidence, the number of cases needed to perform the final analysis would not be reached in the near future and might never be reached based on extrapolation of the accrual of B-CAP cases. Therefore GlaxoSmithKline decided to amend the protocol to re-define the study end based on the outcome of the planned interim analysis for the primary objective.

Note that assessment of CAP in study participants took several months to confirm the classification of each CAP case through X-ray and laboratory analyses. Therefore the data lock point for the interim analysis (when at least 535 B-CAP episodes were identified, which was August 31, 2010) was only defined retrospectively in February 2011, well after the date of protocol amendment 6 (September 9, 2010).
