# Supplementary material for: Efficacy of Pneumococcal Nontypable Haemophilus influenzae Protein D Conjugate Vaccine (PHiD-CV) in Young Latin American Children: A Double-Blind Randomized Controlled Trial
Source: PLoS Med. 2014 Jun 3;11(6):e1001657. doi: 10.1371/journal.pmed.1001657 (PMC4043495; doi:10.1371/journal.pmed.1001657)
Supplement: Text S4 — Laboratory analyses. (DOCX) [file pmed.1001657.s015.docx]

**Text S4 Laboratory analyses**

*Serum samples from suspected community-acquired pneumonia (CAP) cases*:

Blood samples were obtained from all suspected CAP cases. The serum samples were split into two aliquots: one aliquot for local C-reactive protein (CRP) determination (according to local practices) and a second aliquot was stored at -20°C until shipment to the central laboratory for quantitative CRP and procalcitonin determination.

*Middle ear fluid and spontaneous ear discharge samples:*

Middle ear fluid and spontaneous ear discharge samples were inoculated into transport medium (Amies agar gel without charcoal) and sent within 12 hours to the local laboratory for culture, identification of bacterial etiology, and antibiotic sensitivity testing. The most representative bacterial colonies were sent to the central laboratory for confirmation of identity, serotype, and antibiotic sensitivity.

*Blood culture:*

Blood samples for hemoculture were collected as soon as possible after a spike of fever and before the start of antimicrobial therapy, where possible. If blood samples for other tests were taken at the same venipuncture, the blood culture bottles were inoculated first to avoid contamination. It was preferable to take samples for blood culture separately.

The blood culture bottles were inspected for damage or defect before use. They were stored according to the manufacturers’ specifications and expiry dates were not exceeded.

To avoid contamination of the blood culture, it was required to disinfect adequately the site of venipuncture: cleansing with water and soap if necessary and disinfecting with 70% isopropyl or ethyl alcohol. Hereafter, the area was treated with iodine-based solution and allowed to dry completely. The septum of the blood culture bottle was disinfected with ethanol, methanol, or isopropyl alcohol and allowed to dry. Blood from a peripheral vein was withdrawn. Samples were not taken through an intravenous catheter or other access device unless no other access was available.

After taking a blood sample, blood culture was performed using a licensed fully automated continuously monitoring blood culture system (such as BacT/ALERT^®^). Blood culturing was started as soon as possible after sampling. In the meantime, blood was stored at room temperature (15–25°C). A single pediatric bottle appropriate for small volumes of blood was used (recommended volume, 3 ml).

Once the incubator detected a positive sample, the sample was processed immediately. Positive automated cultures (or other types of cultures) were sub-cultured by plating onto chocolate and blood agar plates and incubated aerobically with 5–10% CO_2_ at 35°C for 48 hours. In those laboratories where an additional automated system was available (such as Vitek^®^), differentiation between samples could be made through this system. If the sample became positive during the night and there was no trained person available, the next shift was awaited to process the sample. Dramatically low viable cells count has been described for pneumococci if blood culture bottles had been incubated for more than 10 hours after being detected to be positive [1]. Therefore the remaining sample of each positive blood culture bottle was divided into two cryovials (1.5 ml each) and kept at -70°C for possible further analysis.

Bacterial isolates were kept in duplicate in cryo-conservation medium at -70°C. After confirmation of the date of shipment to the central laboratory, isolates were sub-cultured in blood and chocolate agar media and prepared for transportation in Amies Charcoal medium at room temperature. The central laboratory confirmed viability of the strains to the local laboratory. In addition, local requirements for national surveillance programs were fulfilled. Back-up samples were destroyed unless local requirements for relevant national surveillance programs defined differently.

*Other body fluids:*

Normally sterile body fluids such as cerebrospinal fluid (CSF), pleural effusion, synovial fluid, peritoneal fluid, or pericardial fluid were examined by direct smears and cultured in parallel on appropriate chocolate and blood agar plates. It was also recommended that these fluids were inoculated in special broth for automated culture as was used for blood culture. Sub-culturing was performed as described for blood culture.

In case of a suspicion of meningitis with collection of CSF, in addition to the local laboratory procedures and bacteriological culture, performance of BinaxNOW^®^ test on CSF was mandatory. For this purpose GlaxoSmithKline ensured the availability of this test at each local laboratory. The BinaxNOW^®^ *Streptococcus pneumoniae* test is a rapid immunochromatographic (ICT) *in vitro* assay for the detection of *S. pneumoniae* C-polysaccharide and was used, in conjunction with culture and other methods, to aid the diagnosis of pneumococcal meningitis. CSF was collected according to standard procedures and stored at room temperature (15–25°C) for up to 24 hours before testing. Alternatively, CSF could be refrigerated (2–8°C) or frozen (-20°C) for up to 1 week before testing. As a negative BinaxNOW^®^ test did not exclude infection with *S. pneumoniae*, the results of this test as well as culture results, serology or other antigen detection methods were used in conjunction with clinical findings to make an accurate diagnosis.

*Other laboratory procedures:*

In the participating countries, the following laboratory procedures were performed:

- Identification of colonies of gram-positive cocci as *S. pneumoniae* was based on inhibition by optochin and bile solubility [2].
- Identification of colonies of *Haemophilus influenzae* was done by incubation on chocolate agar plates by Factors X and V growth requirements and biochemical tests [3]. Positive blood cultures were sampled within 10 hours after being detected as positive.

All *S. pneumoniae* and *H. influenzae* strains isolated in the context of the current study in all participating countries were kept in cryo-conservation medium at -70°C until preparation for transport. For this purpose, strains were subcultured on chocolate and blood agar media, the most representative colonies were selected, transferred to Amies charcoal transport medium and immediately sent at room temperature to the central laboratory where the strains were serotyped according to established procedures [3]. The absence of slide agglutination in the presence of a to f antisera (*H. influenzae* Agglutinating Sera MUREX ZM 20-25) was used to identify non-encapsulated (nontypable) *H. influenzae* strains.

**References**

1. Casetta A, Derouin V, Boussougant Y. (1996) Absence of spontaneous autolysis of *Streptococcus pneumoniae* in aerobic fan culture bottles in a commercial blood culture system. Eur J Clin Microbiol Infect Dis 15: 616-617.

2. Murray PR, Baron EJ, Pfaller MA. (1995) Manual of Clinical Microbiology. 6th edition ed. Washington DC: American Society of Microbiology.

3. Austrian R. (1976) The quellung reaction, a neglected microbiologic technique. Mt Sinai J Med 43: 699-709.
